# Varicella-zoster virus reactivation and the risk of dementia

**DOI:** 10.1038/s41591-025-03972-5

**Published:** 2025-10-06

**Authors:** Vitaly Polisky, Maria Littmann, Aleksei Triastcyn, Max Horn, Andreas Georgiou, Robyn Widenmaier, Bruno Anspach, Halima Tahrat, Sanjay Kumar, Carolyn Buser-Doepner, Pascal Geldsetzer, Cornelia M. Van Duijn, Patrick Schwab

**Affiliations:** 1 GSK, Heidelberg, Germany; 2 GSK, Baar, Switzerland; 3 GSK, Toronto, Ontario Canada; 4 GSK, Rockville, MD USA; 5 GSK, Collegeville, PA USA; 6 Division of Primary Care and Population Health, Department of Medicine, Stanford University, Stanford, CA USA; 7 Chan Zuckerberg Biohub, San Francisco, CA USA; 8 Nuffield Department of Population Health, University of Oxford, Oxford, UK

**Keywords:** Neurological disorders, Risk factors

## Abstract

Varicella-zoster virus (VZV) is a neurotropic virus that establishes lifelong latency in humans. VZV reactivation is associated with a wide range of symptoms, including herpes zoster (HZ; also known as shingles), and has been implicated in the development of dementia, although to an unknown extent. Here we present a large-scale longitudinal analysis of health records from more than 100 million individuals in the United States that demonstrates a consistent relationship of VZV reactivation with dementia after controlling for nearly 400 measured characteristics (covering demographics, socioeconomic factors, comorbidities, medications, proxies for healthcare-seeking behavior, shifts in clinical guidelines and completeness of records). We found that recurrent HZ was associated with an increased risk of dementia compared to a single HZ episode. Additionally, exposure to HZ vaccines was associated with a reduced risk of dementia compared to the control 23-valent pneumococcal polysaccharide vaccine. Furthermore, the reduced risk of dementia after administration of the live-attenuated zoster vaccine waned over time and was highly correlated with a waning of the vaccine-mediated protection against HZ. The dementia risk reduction at 3 and 5 years postexposure was also stronger in individuals who received multiple as opposed to only one dose of the recombinant HZ vaccine and those at greater risk of HZ. Our findings strongly implicate VZV reactivation as a modifiable risk factor for dementia.

## Abstract (web summary)

Large-scale longitudinal health records reveal consistent association of varicella-zoster virus reactivation with dementia.

## Main

Dementia is associated with a decline in cognitive function that considerably interferes with daily activities and mostly affects older adults^1^. Due to the globally rising average age and the lack of effective preventive and therapeutic measures, the number of people living with dementia is expected to reach 153 million in 2050 (ref. ^2^). The underlying causes of dementia are complex and incompletely understood. Genetics, vascular and metabolic diseases, and several lifestyle factors have been linked to an increased risk of being diagnosed with dementia^1,3,4^.

Evidence for a potential role of neurotropic human herpes viruses in dementia and Alzheimer’s disease has been mounting, particularly for herpes simplex virus 1 (HSV-1) and varicella-zoster virus (VZV)^5–8^, which are carried by most adults^9,10^. Molecular studies suggest that VZV may contribute to dementia or Alzheimer’s disease-like pathology (deposition of neurotoxic amyloid peptides) directly^11,12^ or indirectly through reactivation of quiescent HSV-1 (ref. ^13^). Cerebrospinal fluid from individuals infected with VZV was shown to contain increased levels of amyloid and amylin compared to controls^11^. Increased levels of intracellular amylin, β-amyloid peptides and amyloid in VZV-infected (but not mock-infected or HSV-1-infected) quiescent primary human perineurial cells and spinal astrocytes were also observed^11,12^. Other studies in cell and animal models have shown that infection with HSV-1 is followed by increased levels and accumulation of β-amyloid 1–40 and 1–42, as well as hyperphosphorylated tau^14–16^. Finally, ref. ^13^ showed that VZV infection of naive human neural stem cells led to gliosis and increased the levels of pro-inflammatory cytokines, but did not directly induce a neurotoxic phenotype. However, VZV infection caused HSV-1 reactivation in quiescently infected cells, resulting in dementia-like cellular changes^13^.

In line with the proposed roles of herpes viruses in the development of dementia, antiherpetic antiviral treatments^17–19^ and vaccinations protecting against clinical VZV reactivation (that is, herpes zoster (HZ))^20–27^ have been associated with a lower risk of dementia and/or Alzheimer’s disease. While these findings are of great interest to public health, a mechanistic understanding of these observations is lacking. Our work addresses this gap by investigating and establishing continuous VZV reactivation as the likely mechanistic path by which HZ contributes to the development of dementia.

To confirm the hypothesis that VZV reactivation contributes to the risk of dementia, we study several assumptions, all of which would have to be true if the hypothesis were true.

The first assumption—that a greater burden of VZV reactivation leads to an increased dementia risk—is tested by comparing individuals with multiple episodes versus a single episode of HZ.

The second assumption—that a lower burden of reactivation leads to a decreased dementia risk—is tested by investigating future dementia risk in individuals who received an HZ vaccine (the live-attenuated zoster vaccine (ZVL; Zostavax, Merck) or the recombinant zoster vaccine (RZV; Shingrix, GSK)).

The third assumption—that the strength of modulation of VZV reactivation is proportional to dementia risk reduction—is tested by evaluating the varying levels of HZ protection in individuals receiving different types and dosages of HZ vaccines (at least one dose of ZVL, one dose of RZV or at least two doses of RZV).

The fourth assumption—that the impact of VZV reactivation on dementia risk is proportional to protection against VZV reactivation over time—is tested by leveraging the waning protection against HZ over ~10 years in individuals vaccinated with ZVL.

Finally, the fifth assumption—that individuals at higher risk of VZV reactivation benefit more from vaccination against HZ—is tested by evaluating dementia risk after ZVL or RZV vaccination in individuals at increased risk for VZV reactivation (that is, females and the elderly).

As we test the above assumptions, we consistently demonstrate that reduced VZV reactivation is the mechanism by which HZ vaccination protects against dementia, thereby lending mechanistic support to existing observational findings.

The above is achieved by leveraging large-scale longitudinal electronic health records (EHRs) of diverse cohorts of individuals aged 50 years or older who were exposed to various factors linked to VZV reactivation. To advance the confidence in findings beyond prior work, we (1) increase the degree to which unmeasured confounders are controlled for by including and adjusting for almost 400 EHR-derived covariates and using active controls, (2) quantify the risk of residual confounding (and demonstrate that residual confounding was addressed) and (3) replicate existing observations from distinct studies using this high standard of confounder control in a single, large dataset across different settings. The entirety of our findings under comprehensive confounder control strongly implicates VZV reactivation as a modifiable risk factor in progression to dementia.

## Results

**Fig. 1:**
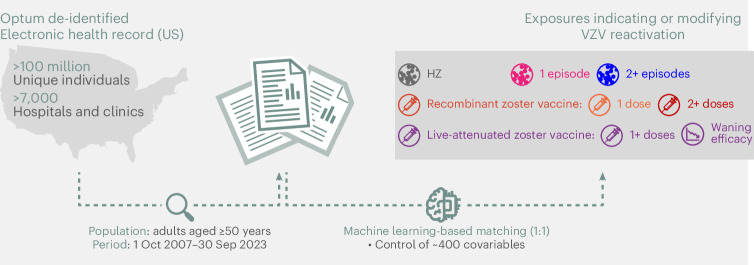
Study design. We evaluated the effects of multiple exposures that indicate or influence VZV reactivation on progression to dementia in a large, representative US cohort after controlling for nearly 400 covariates.

We used the multi-center Optum EHR database, which comprises de-identified longitudinal health records from over 100 million unique individuals seen at more than 7,000 hospitals and clinics in the United States between 2007 and 2023. As part of routine clinical care, some of these individuals were subjected to various exposures that indicate or impact the level of VZV reactivation, including recurrent HZ and different VZV vaccines (ZVL and RZV; Fig. 1). We used the comprehensive EHR data to construct a covariate set of nearly 400 pre-exposure characteristics, including demographics, comorbidities/lifestyle factors (for example, smoking status, cardiovascular conditions and diabetes), healthcare utilization and healthcare-seeking behaviors (Supplementary Tables 1–18). Using these covariates, we created 1:1 propensity-score-matched cohorts of individuals aged 50 years or older using a machine-learning-based methodology (Methods). After constructing the matched cohorts, we used recorded diagnoses of dementia and exposures of interest to evaluate the relationship between VZV reactivation and progression to dementia. Dementia diagnosis and exposures of interest were identified based on the presence of at least one related code (Supplementary Tables 19 and 20) in the Optum EHR (International Classification of Diseases codes for dementia and HZ, Current Procedural Terminology and National Drug codes for vaccinations). HZ, a form of clinically manifested VZV reactivation that presents as a painful vesicular rash^28^, was the only VZV reactivation form investigated in this study. Other exposures of interest were HZ vaccines (RZV and ZVL) and the 23-valent pneumococcal polysaccharide vaccine (PPSV23). PPSV23 was chosen as an active control because it is an elective vaccine indicated for a largely similar population as HZ vaccines^29^, but not conferring protection against HZ.

### HZ recurrence is associated with increased risk of dementia

**Fig. 2:**
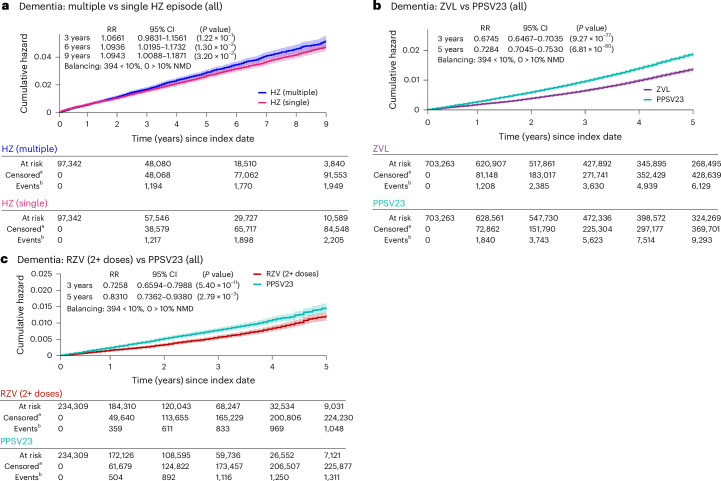
Exposures indicating or impacting VZV reactivation are associated with a modified risk of dementia. **a**, Experiencing two or more episodes of HZ is associated with a substantially increased dementia risk (cumulative hazard, *y* axis) up to 9 years after the second HZ event (*x* axis), compared to experiencing a single episode of HZ. **b**,**c**, Suppression of VZV reactivation through exposure to the (**b**) ZVL and (**c**) RZV is associated with a substantially reduced risk of dementia compared with exposure to the elective PPSV23 used as control at 3 and 5 years postindex date (PPSV23 is used by a similar population as HZ vaccines^29^ but does not confer protection against HZ). Results presented here are consistent with results obtained using IPTW and OW (Supplementary Figs. 7 and 8 and Supplementary Tables 21 and 22); see Methods for more details. The postmatching cohort characteristics for comparisons shown in this figure are presented in Supplementary Table 23. The curves show the Nelson–Aalen estimates of the cumulative hazard function (*y* axis) over the follow-up period (*x* axis) with a 95% CI band (shaded areas around each curve) for each cohort being compared. Cumulative hazards at specific time points are compared between the cohorts based on RR using a two-sided chi-squared statistic with 1 degree of freedom without adjusting for multiple comparisons. Superscript letter ‘a’ indicates censored, cumulative number of individuals who were lost to follow-up or had died until each time point; superscript letter ‘b’ indicates cumulative numbers of individuals with the event of interest (dementia diagnosis) until each time point; those numbers are to be interpreted relative to the cohort size under observation as indicated by ‘At risk’. NMD, normalized mean difference; RR, relative risk. Source data

We found that, compared to a single HZ episode, experiencing multiple HZ episodes was associated with a 7–9% higher risk of dementia at 3–9 years after index date (that is, the second HZ episode; Fig. 2a).

### Vaccine-mediated prevention of HZ episodes is associated with reduced risk of dementia

Vaccination against HZ was associated with reductions in dementia risk of 33% (95% confidence interval (CI) = 30–35%) for ZVL and 27% (95% CI = 20–34%) for at least 2 doses of RZV (RZV (2+ doses)) at 3 years after index date compared to those exposed to PPSV23 (Fig. 2b,c). The lower risk of dementia in the ZVL and RZV cohorts compared to the PPSV23 cohort persisted until 5 years postindex date (Fig. 2b,c).

### Higher vaccine-mediated protection against HZ is associated with greater reductions in dementia risk

**Fig. 3:**
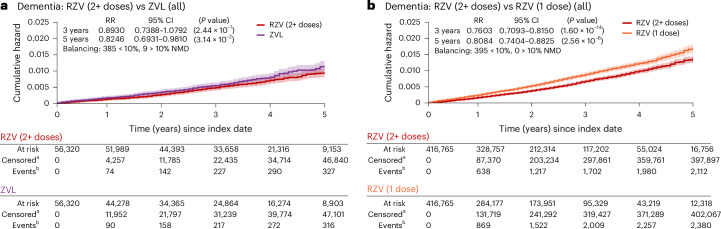
Consistent relationship between VZV reactivation and the risk of dementia diagnosis across exposures, vaccine dosage and time. **a**, When directly comparing the recombinant (RZV) to the ZVL, RZV was associated with a lower dementia risk (cumulative hazard, *y* axis) at 5 years after index date (*x* axis). **b**, Receiving two or more doses of RZV was associated with a substantially lower risk of dementia at 3 and 5 years after index date compared to having received only a single dose of RZV. The postmatching cohort characteristics for comparisons shown in this figure are presented in Supplementary Table 24. The curves show the Nelson–Aalen estimates of the cumulative hazard function (*y* axis) over the follow-up period (*x* axis) with a 95% CI band (shaded areas around each curve) for each cohort being compared. Cumulative hazards at specific time points are compared between the cohorts based on RR using a two-sided chi-squared statistic with 1 degree of freedom without adjusting for multiple comparisons. Superscript letter ‘a’ indicates censored, cumulative number of individuals who were lost to follow-up or had died until each time point; superscript letter ‘b’ indicates cumulative numbers of individuals with the event of interest (dementia diagnosis) until each time point. NMD, normalized mean difference; RR, relative risk. Source data

Separate clinical trials reported on a two-dose RZV efficacy of 97.2% (95% CI = 94–99%) in adults aged 50 years and older and 91.3% (95% CI = 87–95%) in those aged 70 years and older^30,31^, as well as a ZVL efficacy of 70% (54–81%) in adults aged 50–59 years and 38% (25–48%) in those aged 70 years and older^32–34^. In real-world studies, two RZV doses were more effective against HZ than one dose^35,36^. When comparing RZV (2+ doses) with ZVL in the present study, dementia risk was 18% (95% CI = 2–31%) lower for RZV at 5 years after index date (Fig. 3a). Additionally, at 3 and 5 years after index date, the risk of dementia was 24% (95% CI = 18–29%) and 19% (12–26%) lower in those who received multiple compared to one RZV dose (Fig. 3b).

### Waning of vaccine-mediated suppression of HZ correlates with a return to baseline dementia risk

**Fig. 4:**
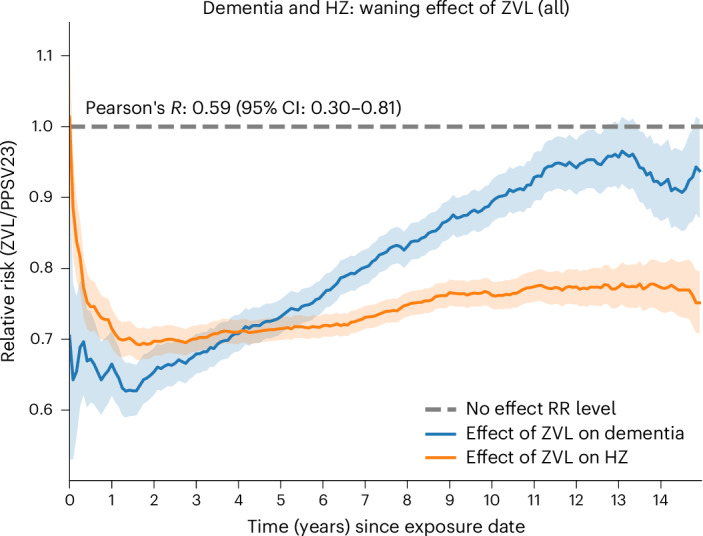
Correlation between the waning effects of the ZVL immunization on HZ and the risk of dementia. As the effectiveness of ZVL in protecting against HZ waned over up to 15 years after index date (*x* axis), the magnitude of protection against dementia similarly diminished over the same time frame. Please note that (1) the *y* axis indicates RR of an outcome (HZ or dementia) in the ZVL cohort, calculated relative to the effect observed in recipients of the PPSV23, which does not confer protection against HZ; (2) *y*-axis values <1 indicate a lower risk due to exposure; (3) the near horizontal line for HZ protection after ~10 years postindex date indicates that HZ cases were prevented, whereas the return of the dementia risk to an RR of ~1 suggests delayed progression to rather than prevention of dementia diagnoses. The curves represent RR (*y* axis) for HZ (orange line) and dementia (blue line) in the ZVL cohort calculated relative to the effect observed in PPSV23 cohort, for each month during the follow-up period along with their respective 95% CI bands (shaded areas around each curve) based on the ratio of the Nelson–Aalen estimates of the corresponding cumulative hazard functions. The Person’s *R* coefficient with a 95% CI shows the level of linear correlation between the curves; RR values <1 indicate a lower risk due to exposure.

The protection against clinical VZV reactivation (that is, HZ) conferred by ZVL was previously found to wane over time, with a near-complete loss of effectiveness around 10 years after vaccination^37^. We therefore studied the relationship between the waning of protection against HZ and the reduced risk of dementia for up to 15 years of follow-up (Fig. 4). The effect of ZVL was evaluated relative to the PPSV23 control cohort, in which the cumulative numbers of HZ and dementia cases were expected to increase during the 15-year follow-up. Among those who received ZVL, the protective association of this vaccination with dementia diagnoses was correlated with the protective association with HZ episodes (Pearson’s *R* = 0.59; Fig. 4). This finding implies a consistent relationship between the waning ZVL effects observed for HZ and dementia.

In Fig. 4, we observe that the relative risk (RR) of HZ remains relatively stable while the RR of dementia continually regresses toward the value of 1. We attribute this observation to HZ cases being prevented and dementia cases being merely delayed (that is, in those developing dementia, progression is slower but eventually resumes as ZVL-associated protection wanes). During the strong vaccine-mediated control of VZV reactivation, VZV reactivation may contribute less to progression to dementia (for example, using an inflammatory cascade eventually leading to cell death). However, as protection against VZV reactivation wanes, the dementia-triggering processes may resume, eventually leading to dementia being diagnosed later than it may have been without a protective intervention. We note that this observation also matches our understanding of neurodegeneration as a continuous, progressive process, rather than a single discrete event.

A similar long-term analysis for RZV was not possible at the time of this publication, due to the relatively recent US market entry of RZV in 2017 (ref. ^38^) and the resulting limited number of individuals with 4–5 years of follow-up after receipt of RZV.

### Dementia-related benefits of reducing clinical VZV reactivation are greater in those at higher risk of HZ

**Fig. 5:**
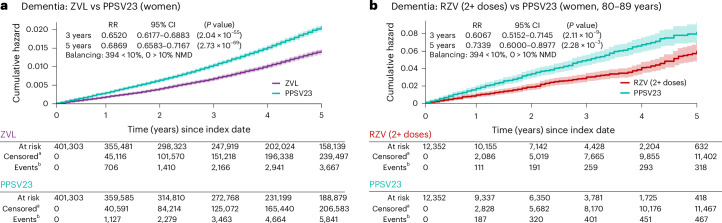
More prominent reduction in the risk of dementia due to vaccination observed in cohorts at higher risk of HZ. **a**,**b**, Comparing dementia risk (cumulative hazard, *y* axis) over time (*x* axis) after (**a**) exposures of female individuals to the ZVL and (**b**) exposure of female individuals aged 80–89 years to the RZV showed a substantial association with a decreased dementia risk at 3 and 5 years after index date compared to the PPSV23. The postmatching cohort characteristics for comparisons shown in this figure are presented in Supplementary Table 25. The curves show the Nelson–Aalen estimates of the cumulative hazard function (*y* axis) over the follow-up period (*x* axis) with a 95% CI band (shaded areas around each curve) for each cohort being compared. Cumulative hazards at specific time points are compared between the cohorts based on RR using a two-sided chi-squared statistic with 1 degree of freedom without adjusting for multiple comparisons. Superscript letter ‘a’ indicates censored, cumulative numbers of individuals who were lost to follow-up or had died until each time point; superscript letter ‘b’ indicates cumulative numbers of individuals with the event of interest (dementia diagnosis) until each time point. NMD, normalized mean difference; RR, relative risk. Source data

Some individual characteristics, such as older age and female sex, are known to be associated with a higher risk of HZ^28,39,40^. If reduction of VZV reactivation is a mechanism by which progression to dementia is delayed after HZ vaccination, and assuming greater rate/magnitude of reactivations in those at higher risk^28,39^, one would expect a greater impact of ZVL or RZV immunization on progression to dementia in those at higher risk of HZ compared to those at lower risk. We thus investigated whether the prevention of HZ in populations at higher risk also conveyed a greater reduction in progression to dementia. When stratifying by sex and age, we found greater relative reductions in the risk of dementia for older and/or female individuals than the overall cohorts exposed to HZ vaccines compared to PPSV23 at 3 and 5 years after index date (Fig. 5). Female individuals aged ≥50 years and who were exposed to ZVL had a 35% (95% CI = 31–38%) and 31% (28–34%) lower risk of being diagnosed with dementia at 3 and 5 years after index date (compared to 33% and 27% for all individuals; Fig. 5a). Additionally, female individuals aged 80–89 years who were exposed to multiple doses of RZV had a 39% (29–48%) and 27% (10–40%) lower risk of dementia diagnoses at 3 and 5 years after index date (compared to 27% and 17% for all individuals; Fig. 5b).

### Machine-learning control of nearly 400 covariates and use of active exposure comparison groups effectively reduce the risk of residual confounding

**Extended Data Fig. 1:**
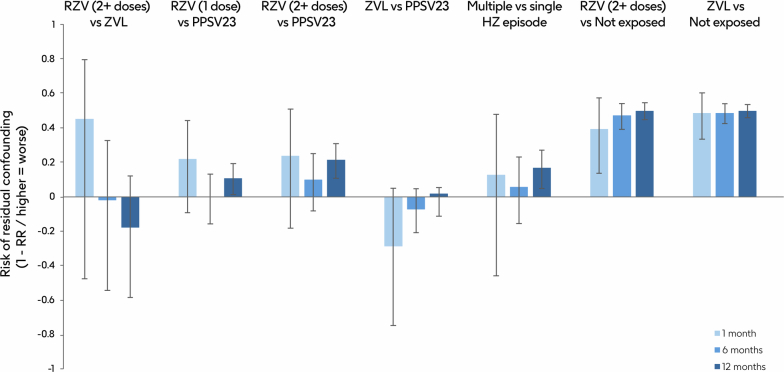
Evaluation of residual confounding. We quantified the risk of residual confounding in primary comparisons between several cohorts (individuals exposed to the live-attenuated zoster vaccine [ZVL], recombinant zoster vaccine [RZV], the 23-valent pneumococcal polysaccharide vaccine [PPSV23], not exposed [Not exposed] to any herpes zoster [HZ] vaccine and individuals with only a single versus multiple HZ episodes) using a negative control via time shift (NCTS) analysis. Briefly, to conduct the NCTS analysis, we artificially shifted the exposure date of the exposed groups to 1 year prior to their actual recorded exposure while keeping other elements of the study design constant. Since this new exposure date is before any biological effect could have taken place, any differences in cohorts already present before exposure indicate residual confounding not accounted for via matching. The estimated risk of residual confounding is represented as a per-comparison group of bars for each of the primary comparisons with height reflecting an estimate of (1 – relative risk [RR]) at 3, 6, and 9 months after the shifted index date based on the Nelson-Aalen estimates of cumulative hazards along with error bars indicating a 95% confidence interval (CI). The respective sample sizes in terms of the number of matched pairs of individuals at the shifted index date are: 77,331 (RZV [2+ doses] vs ZVL), 276,987 (RVZ [1 dose] vs PPSV23), 191,073 (RZV [2+ doses] vs PPSV23), 344,159 (ZVL vs PPSV23), 85,650 (HZ [multiple] vs HZ [single]), 481,124 (RZV [2+ doses] vs Not exposed), 441,740 (ZVL vs Not exposed). The higher the y-axis value, the more residual confounding was present in a comparison. We found that comparisons between elective vaccines (ZVL, RZV, PPSV23) were generally at a lower risk of residual confounding than comparisons to Not exposed, a finding suggesting that using another elective vaccine (PPSV23) as control is effective at reducing residual confounding. Error bars represent 95% CIs. Source data

**Extended Data Fig. 2:**
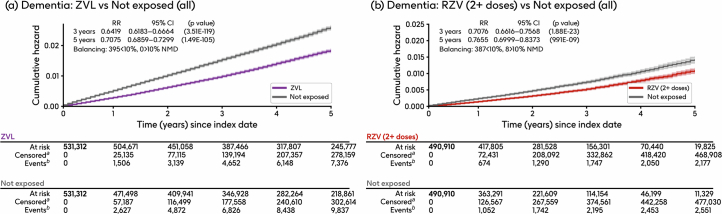
Comparison to cohorts not exposed to herpes zoster (HZ) vaccines (Not exposed). We evaluated the risk of dementia (cumulative hazard, y*-*axis) over time (x*-*axis, years since exposure) following exposure to (**a**) the live-attenuated zoster vaccine (ZVL) and (**b**) at least 2 doses of the recombinant zoster vaccine (RZV). The curves show the Nelson-Aalen estimates of the cumulative hazard function (y-axis) over the follow-up period (x-axis) with a 95% confidence interval (CI) band (shaded areas around each curve) for each cohort being compared. Cumulative hazards at specific time points are compared between the cohorts based on relative risk (RR) using a two-sided chi-squared statistic with 1 degree of freedom without adjustments for multiple comparisons. The sample size (n) for each cohort is indicated as the counts of individuals at risk in the ‘At risk’ row in the event table. Both ZVL and RZV are associated with a significantly decreased risk of dementia 3 and 5 years after exposure when compared to Not exposed. Post-matching cohort characteristics are listed in Supplementary Table 26. Superscript letter ‘a’ indicates censored, cumulative numbers of individuals who were lost to follow-up or had died until each time point; superscript letter ‘b’ indicates cumulative numbers of individuals with the event of interest (dementia diagnosis) until each time point. NMD, normalized mean difference. Source data

**Extended Data Fig. 3:**
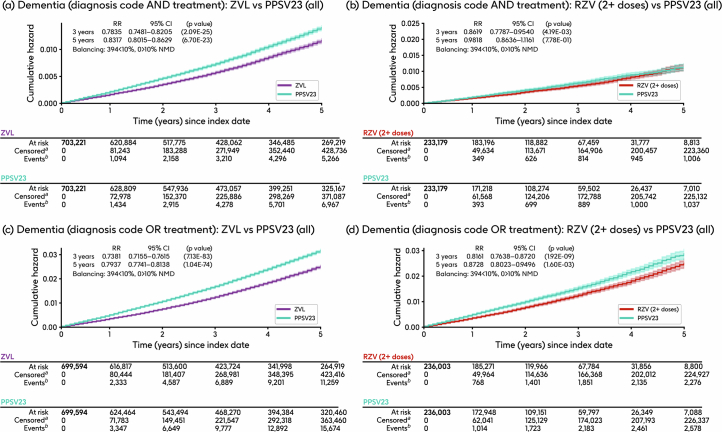
Herpes zoster (HZ) vaccine comparisons using different dementia definitions. We evaluated the risk of dementia (cumulative hazard, y-axis) over time (x-axis, years since exposure) using a more precise but less sensitive dementia definition (requiring diagnosis code and dementia treatment; **a** and **b**) and a more sensitive but less precise dementia definition (requiring diagnosis code or dementia treatment; **c** and **d**) following exposure to the live-attenuated zoster vaccine (ZVL) (**a** and **c**) and at least 2 doses of the recombinant zoster vaccine (RZV) (**b** and **d**). The curves show the Nelson-Aalen estimates of the cumulative hazard function (y-axis) over the follow-up period (x-axis) with a 95% confidence interval (CI) band (shaded areas around each curve) for each cohort being compared. Cumulative hazards at specific time points are compared between the cohorts based on relative risk (RR) using a two-sided chi-squared statistic with 1 degree of freedom without adjustments for multiple comparisons. The sample size (n) for each cohort is indicated as the counts of individuals at risk in the ‘At risk’ row in the event table. Post-matching cohort characteristics are listed in Supplementary Table 27. Superscript letter ‘a’ indicates censored, cumulative numbers of individuals who were lost to follow-up or had died until each time point; superscript letter ‘b’ indicates cumulative numbers of individuals with the event of interest (dementia diagnosis) until each time point. NMD, normalized mean difference; PPSV23, 23-valent pneumococcal polysaccharide vaccine. Source data

**Extended Data Fig. 4:**
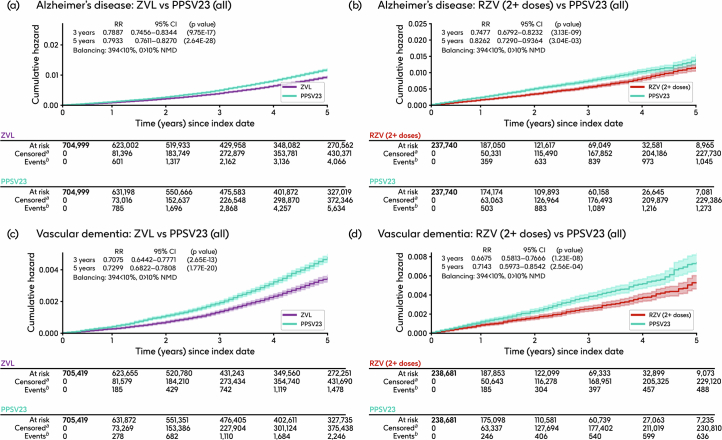
Comparisons for different dementia subtypes. We evaluated the risk of dementia (cumulative hazard, y-axis) over time (x-axis, years since exposure) for the main subtypes of dementia following exposure to the live-attenuated zoster vaccine (ZVL) (**a** and **c**) and at least 2 doses of the recombinant zoster vaccine (RZV) (**b** and **d**). The curves show the Nelson-Aalen estimates of the cumulative hazard function (y-axis) over the follow-up period (x-axis) with a 95% confidence interval (CI) band (shaded areas around each curve) for each cohort being compared. Cumulative hazards at specific time points are compared between the cohorts based on relative risk (RR) using a two-sided chi-squared statistic with 1 degree of freedom without adjustments for multiple comparisons. The sample size (n) for each cohort is indicated as the counts of individuals at risk in the ‘At risk’ row in the event table. Both ZVL and RZV are associated with a significantly decreased risk 3 and 5 years after exposure when compared to those exposed to the 23-valent pneumococcal polysaccharide vaccine (PPSV23) for both Alzheimer’s disease (**a** and **b**) and vascular dementia (**c** and **d**). Post-matching cohort characteristics are listed in Supplementary Table 28. Superscript letter ‘a’ indicates censored, cumulative numbers of individuals who were lost to follow-up or had died until each time point; superscript letter ‘b’ indicates cumulative numbers of individuals with the event of interest (dementia diagnosis) until each time point. NMD, normalized mean difference. Source data

To quantify the risk of residual confounding, we conducted negative control by temporal shift analyses where we synthetically shifted exposure dates by one year before the actual exposures. The index dates were shifted to a point before any biological effect on dementia from an intervention could have occurred, leaving only inherent differences in cohorts as a possible explanation. Using these temporal shift analyses, we found little evidence for residual confounding for active comparisons to other elective vaccines for most pairs of compared exposures (Extended Data Fig. 1). We also consistently observed greater delays in progression to dementia when repeating the main comparisons using a control group of individuals not exposed to HZ vaccines instead of a control group exposed to PPSV23 (Extended Data Fig. 2). However, the temporal shift analyses revealed a higher risk of confounding when comparing vaccine-exposed to the not-exposed cohorts (Extended Data Fig. 1). This finding highlights the importance of extensive confounder control, which was achieved in this study by accounting for nearly 400 covariates and by using comparable control groups. The compared cohorts were generally large, ranging from 56,320 (RZV (2+ doses) versus ZVL) to 703,263 (ZVL versus PPSV23) individuals included, and were well-balanced across the considered covariates after matching (Supplementary Tables 2–18 and 21–28 and Supplementary Figs. 1–6). The robustness of the presented findings was also further validated with respect to different definitions (Extended Data Fig. 3 and Supplementary Table 27) and subtypes of dementia (Extended Data Fig. 4 and Supplementary Table 28).

## Discussion

Our study demonstrated a consistent epidemiological link between VZV reactivation and dementia. This opens the question of a potential causal link between neurotropic human herpes viruses and dementia risk, and the possibility that interventions that reduce VZV reactivation may also delay progression to dementia. Here we leveraged the large multi-center Optum EHR database to demonstrate that a lower VZV reactivation burden (in RZV/ZVL-vaccinated individuals or those with only a single HZ episode) corresponds to a substantially reduced risk of dementia over up to 9 years of follow-up. Conversely, and consistent with the implication of VZV in the development of dementia-like pathology^11–13^, we found an increased risk of dementia in cohorts with an increased clinical VZV reactivation burden (that is, individuals with multiple HZ episodes). The observations were consistent across different degrees of VZV reactivation control, as illustrated, for example, by greater observed benefits in those receiving multiple RZV doses compared to only a single dose. Furthermore, individuals at a higher risk of VZV reactivation (that is, female individuals aged ≥50 and ≥80 years) were found to benefit more from a reduced dementia risk at 3 and 5 years of follow-up through vaccine-suppressed VZV reactivation. This is consistent with the previously reported greater risk reduction in female individuals than in males upon receipt of ZVL^20^ and RZV^25^. Furthermore, we used the naturally waning effectiveness of ZVL^37^ to demonstrate that decreasing protection against VZV reactivation coincides with a loss of dementia-delaying benefits. Together, the associations observed in this study indicate VZV reactivation as a contributing factor in progression to dementia in adults aged ≥50 years.

Although the present data indicate that a relationship between VZV reactivation and dementia is the leading explanation for the findings, it is not yet clear precisely how VZV infection and the subsequent (clinical and subclinical) reactivation of this virus may influence progression to dementia. Molecular studies have reported on the possibility of VZV contributing to dementia or Alzheimer’s disease-like pathology either directly by inducing amyloid deposition^11,12^ or indirectly through reactivation of quiescent HSV-1 (ref. ^13^). Also, VZV infection was identified as a cause of vasculopathy and has been linked to cerebrovascular events (for example, infarction, ischemia and hemorrhage) resembling those observed in Alzheimer’s disease^41^. Our data do not allow us to pinpoint whether the observed effect of VZV reactivation on dementia is HSV-1-mediated or a direct consequence of VZV-mediated pathologies.

Our study examined the link between dementia and clinically manifested VZV reactivation recorded in the EHR as HZ episodes while not investigating other, non-HZ, clinical presentations of VZV reactivation because they are considerably rarer than HZ. However, it is possible that other clinical forms of VZV reactivation or even subclinical VZV reactivation exert effects on dementia progression. Data from saliva, blood and cerebrospinal fluid samples indicate frequent subclinical reactivation of VZV, also in healthy individuals^42–46^, and substantial increases in subclinical VZV reactivation were, for example, reported for astronauts exposed to space flight-associated stress^47^. It is possible that subclinical VZV reactivation without HZ symptoms could be contributing to dementia risk, and the correlation with HZ demonstrated in this study may reflect HZ as a proxy for a higher VZV reactivation burden in general. Further studies evaluating molecular markers of low-level VZV reactivation are necessary to elucidate whether VZV reactivation without clinical presentations or with non-HZ clinical presentations may also be associated with progression to dementia.

The primary limitation of the present study is that causality of the evaluated exposures with the measured dementia outcomes cannot be conclusively established without a prospective, randomized controlled design. However, the consistency of findings across multiple exposures and over time reasonably limits the explanations for the observed findings to one of two probable mechanisms. The first is that the observed differences between exposures were driven by the presence of an unknown or uncontrolled residual confounding factor. An alternative explanation is a potentially causal mechanism related to the VZV-targeting activity of HZ vaccines.

Confounding is a major concern in any retrospective analysis, and we cannot exclude the possibility of residual confounding in our study. However, this risk is lower than in most other studies on the association of dementia with HZ vaccinations and HZ episodes because any bias or confounding would have to affect the results consistently across exposures with the expected effect and over similar time frames. The existence of such systematic confounding factors is not plausibly explained by chance, given the large number of tested exposures and the multi-year time spans evaluated in this study.

To address the risk of unmeasured confounding, we leveraged a combination of extensive data from the Optum EHR and nonlinear machine-learning estimators to control for almost 400 covariates covering demographics, comorbidities, healthcare utilization and healthcare-seeking behaviors. Additionally, by using the PPSV23 active control, we accounted for potential unmeasured confounders related to healthcare-seeking behaviors in the matched populations. In the populations more likely to benefit from diminished VZV reactivation (elderly and female), the observed effect size indicates that a potential unaccounted-for confounder would have to increase dementia risk by 2.66-fold over 3 years after vaccination in the control PPSV23 cohort to explain the observed difference in dementia outcomes between groups (*E* value = 2.66)^48^. Among the most predictive nonmodifiable dementia risk factors, age and sex were controlled for. Genetic variants are known to contribute to the risk of dementia^49^ and were not controlled for in this study. Nevertheless, a large systematic selection bias for those genetically at risk would be expected to be visible in the negative control by temporal shift analyses for residual confounding, as genetics do not change over time. Although several risk factors have been identified as substantial contributors to dementia progression^50^, none of these risk factors would likely provide the required effect magnitude reported to be associated with dementia in the present study. Among the modifiable risk factors of dementia, educational attainment, cognitive reserve and physical activity were not directly controlled for. However, these factors are known to be correlated with healthcare utilization and comorbidities^51^, as well as body mass index and obesity status^52^, all of which were controlled for. We also controlled for other notable modifiable risk factors of dementia, including diabetes and smoking status.

Our sensitivity analyses indicated a minimal risk of residual confounding for the active comparisons with PPSV23. Other potential explanations that could affect both dementia and HZ, such as reverse causality (that is, dementia causing VZV reactivation) or a systematic bias that could affect dementia and HZ jointly, can be ruled out since the protective effects of RZV and ZVL against HZ have been proven in randomized clinical studies. A systemic confounder would therefore have to be specific to dementia and should not affect the impact of the exposures on HZ.

Additionally, the findings of this study regarding HZ vaccination are concordant with those presented for ZVL, as discussed in the ref. ^20^, and for RZV, as discussed in ref. ^25^. By relying on a natural experiment caused by vaccination eligibility based on fixed cutoff dates in Wales, United Kingdom, ref. ^20^ also showed a stronger association between HZ vaccination and reduced dementia risk in female individuals, despite the use of a study design based on regression discontinuity and a dataset from a different healthcare system. Using a similar quasi-experimental approach, ref. ^25^ showed that immunization with RZV was associated with a 17% increase in dementia-free time during 6 years postexposure, and with a substantially lower risk of dementia compared to influenza and tetanus–diphtheria–pertussis control vaccines.

Finally, limitations inherent to EHR-derived data, such as incomplete pharmacy claims and misclassification or absence of exposures and outcomes, were likely present to some extent in this analysis. The restricted length of follow-up for RZV prevented us from comparing its effects on HZ and progression to dementia over a 15-year period (as done for ZVL). While the long-term data are still pending, the effect of RZV on delaying dementia over the same follow-up period may be different, due to the high efficacy and durable protection against HZ after RZV immunization^30,31,53^.

In conclusion, we found a consistent relationship between VZV reactivation and progression to dementia in a large, diverse US database that allowed us to control for nearly 400 covariates. We observed a correlation between protection against VZV reactivation and dementia, as well as between an increased clinical VZV reactivation burden and an increased 3–9-year risk of dementia diagnosis. Establishing VZV reactivation as a modifiable risk factor for progression to dementia could have wide implications for public health, prevention and potential future development of dementia-targeting therapies. While we have taken extensive steps to reduce the risk of confounding, and the findings supporting a relationship between diminished VZV reactivation and a reduced risk of dementia are consistent across different settings and over time, unmeasured confounding cannot be fully ruled out as a potential explanation for the observed results.

## Methods

### Study design and ethics

This was a retrospective, observational, matched-cohort study with pairwise comparisons. No ethical approval from ethics committees or institutional review boards was necessary because no personally identifiable data were collected or analyzed. The study was registered in the HMA–EMA catalogs of real-world data sources and studies (formerly known as EU Post-Authorization Studies (EU PAS) Register) with the number EUPAS107206.

### Population and follow-up

De-identified individual data from the Optum EHR database, which comprises records of >100 million individuals from >2,000 hospitals and >5,000 clinics in the United States, were used in this study. The data used in this study spanned from 1 October 2007 to 30 September 2023. We excluded individuals who:

- were younger than 50 years at the index date,
- did not have a documented birth year,
- had an invalid death status (that is, an individual marked as deceased but without a documented death date or a participant marked as alive but with a death date entered),
- had the index date before birth or after death,
- had the index date before 1 October 2007,
- for the not-exposed and comparator cohorts: fewer than 25 recorded encounters,
- for the single and multiple HZ episodes cohorts: received an HZ vaccination before the exposure date (first HZ episode for the single HZ and second HZ episode for the multiple HZ episodes cohort),
- had a dementia diagnosis before the index date, or
- did not have an index date.

Study participants were included in a cohort if they had a documented respective cohort-defining event and fulfilled the cohort-specific inclusion criteria (Extended Data Table 1). We observed a short-term clustering of PPSV23 exposure and recorded dementia diagnoses (within 10 days of the index date), likely due to participants being vaccinated with PPSV23 soon after being diagnosed with dementia. Additionally, a small fraction of diagnostic records may have been delayed, resulting in some diagnoses being recorded after the PPSV23 exposure. To avoid including individuals with a delayed diagnosis, we excluded all persons with a dementia diagnosis within 10 days after PPSV23 vaccination from the analyses.

**Extended Data Fig. 5:**
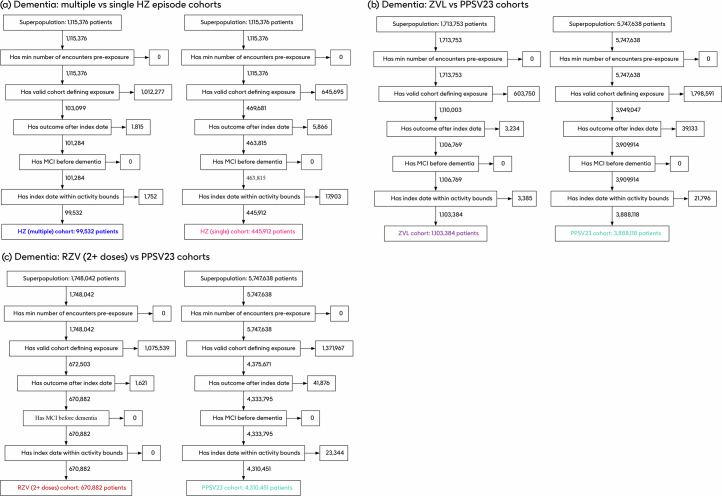
Cohort flow diagrams for the primary comparisons. Comparison of dementia outcomes for cohorts that experienced multiple vs a single HZ episode (**a**), that were exposed to ZVL vs PPSV23 (**b**) and that were exposed to RZV (2+ doses) vs PPSV23 (**c**). Multiple and single HZ, individuals experiencing at least 2 (multiple) and 1 (single) episodes of herpes zoster (HZ); MCI, mild cognitive impairment; OW, overlap weighting; PPSV23, recipients of at least 1 dose of a 23-valent pneumococcal polysaccharide vaccine; RZV (2+ doses), recipients of at least 2 doses of the recombinant zoster vaccine (Shingrix, GSK); ZVL, recipients of at least 1 dose of the live-attenuated zoster vaccine (Zostavax, Merck).

Individuals without a specific cohort-defining event were included in the respective not-exposed cohorts. The not-exposed cohort for comparisons against individuals vaccinated with either RZV or ZVL only included people who did not receive either of the two HZ vaccines but could receive PPSV23 (receipt of PPSV23 was accounted for during matching). For comparisons between vaccinated individuals, we also excluded individuals from one cohort who received the vaccine from the other cohort before the index date. To ensure minimal data availability, only individuals with at least 25 recorded healthcare encounters over the entire observation period were included in the respective not-exposed and comparison cohorts. Cohort flow diagrams for primary comparisons are provided in Extended Data Fig. 5. The index date for each cohort was defined as the date of the first HZ diagnosis for individuals with a single episode, or the second diagnosis for recurrent HZ, or the first immunization with RZV, ZVL or PPSV23 (Extended Data Table 1 and Supplementary Tables 19 and 20). For individuals in the not-exposed cohorts, the possible index dates were sampled randomly from all documented healthcare encounters. During matching, we ensured that the index date of each matched unexposed individual fell within approximately 3 months before or after the index date of the matched exposed individual.

Individuals included in a cohort were followed up until experiencing one of the following censoring events:

- end of their records (that is, last observed record) in the Optum EHR dataset
- death
- diagnosis of interest
- end of study (30 September 2023)
- experiencing the comparator cohort-defining event (for example, record of ZVL immunization in an individual who previously received RZV)
- for comparisons including HZ episodes: record of an RZV or ZVL vaccination after the HZ diagnosis
- for vaccination comparisons: receiving any HZ vaccination other than the cohort-defining vaccine or receiving another dose of the cohort-defining vaccine beyond the number of doses required for the cohort.

The earliest of the above-listed events was taken as the censoring date. To mitigate the impact of data errors and to make the censoring approach more robust, we applied a safety margin or a shift for certain censoring events (Extended Data Table 2). The safety margin defines the minimal period to count the censoring event as valid. Censoring was not applied if the time elapsed between the index date and the censoring event was below this safety margin, allowing to control for short periods between events. In the case of a shift, the censoring event date was shifted forward by a certain period. Those two adjustments of censoring events allowed us to reduce the impact of certain data errors, that is, RZV vaccination recorded as ZVL, multiple records of the same vaccination and delayed records of dementia diagnosis that were associated with a PPSV23 vaccination.

Furthermore, in the negative control by temporal shift comparisons with the PPSV23 control, to mitigate the effect of PPSV23 exposures administered in response to dementia diagnoses, no censoring on the cohort-defining vaccines was applied. Instead, we included only individuals for whom the other cohort-defining vaccine could not have affected the outcome of interest (that is, only those who either did not receive the other cohort-defining vaccine or received it after the outcome of interest).

### Machine-learning matching methodology

We followed a propensity-score matching methodology to build comparable populations for each pairwise comparison. A machine-learning approach was used to compute propensity scores based on key covariates (demographic (age at index date, sex, ethnicity, race and region), healthcare utilization (duration of records in months pre-exposure, median number of recorded visits per year, number of recorded medication prescriptions and immunizations), index date time frame (month of exposure), comorbidities (for example, depression, diabetes, stroke and hypertension) and lifestyle factors (for example, smoking)). In total, we considered 395 covariates in matching, some of which were excluded for the following specific comparisons:

- For comparisons with vaccinated individuals (that is, immunization with RZV, ZVL or PPSV23), the cohort-defining vaccine was excluded, resulting in 394 covariates for comparisons among 2 cohorts vaccinated with different vaccines, 395 covariates for cohorts vaccinated with RZV (2+ doses versus 1 dose) and 395 covariates for comparisons between vaccinated and not-exposed cohorts.
- For comparisons of individuals with an HZ diagnosis, both HZ vaccines (RZV and ZVL) were excluded, resulting in 393 covariates.

Removal of these covariates did not introduce any bias, as, due to the way the cohorts were defined, none of the included individuals had received the vaccinations removed from matching or had been diagnosed with HZ before exposure.

Covariates were selected to maximize the use of information contained in the EHR data and to thoroughly address possible sources of confounding. We worked with clinical and neurological experts to identify the relevant covariate groups (for example, demographics, socioeconomic factors, dementia risk factors, comorbidities, medications and healthcare utilization) and then derived all available covariates and proxies as they were available in the EHR data.

**Extended Data Fig. 6:**
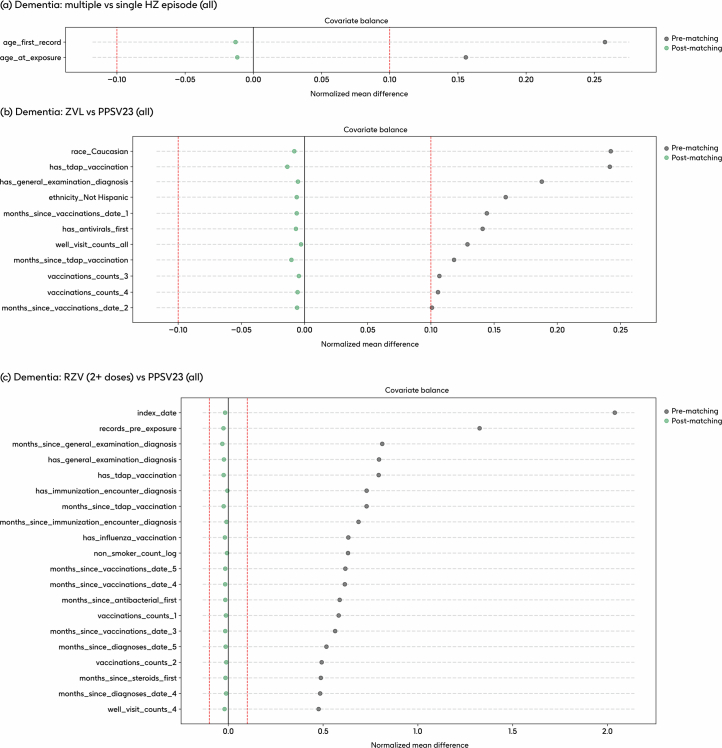
Select pre- and post-matching covariate balances (based on normalized mean difference values) in the primary comparisons. Comparisons of dementia outcomes for cohorts that experienced multiple vs a single HZ episode (**a**), that were exposed to ZVL vs PPSV23 (**b**), and that were exposed to RZV (2+ doses) vs PPSV23 (**c**). The values on the y-axis are the names of covariates. The dots indicate pre- and post-matching normalized mean difference (NMD) for the corresponding covariate. The red vertical lines indicate the bounds of what is considered a good balance (−0.1 < NMD < 0.1). Post-matching dots within these bounds indicate that the corresponding covariate’s mean is well balanced after matching. The covariates are sorted in descending order of their pre-matching NMD. Note for interpretation of covariate names: For covariates named ‘months_since_i’ (where i = 1, 2, 3, 4, 5), the name indicates months between i^th^ last intervention pre-exposure and the exposure date. For ‘months_since_vaccination’, the term ‘vaccination’ refers to any vaccination except the exposure vaccination. For ‘months_since_diagnosis’ covariate, the term ‘diagnosis’ refers to any diagnosis in the Diagnosis table with a certain status. For covariates named ‘intervention_counts_i’, the name indicates the number of interventions in the i^th^ pre-exposure year. The covariate named ‘well_visits_counts_all’ indicates the total count of routine medical and gynecological examinations. Multiple and single HZ, individuals experiencing at least 2 (multiple) and 1 (single) episodes of herpes zoster (HZ); PPSV23, recipients of at least 1 dose of a 23-valent pneumococcal polysaccharide vaccine; RZV (2+ doses), recipients of at least 2 doses of the recombinant zoster vaccine (Shingrix, GSK); tdap, receipt of tetanus-diphtheria-acellular pertussis vaccine; ZVL, recipients of at least 1 dose of the live-attenuated zoster vaccine (Zostavax, Merck).

To compare two cohorts, a machine-learning model was trained to predict assignment probabilities for each individual in the respective cohorts (propensity scores), allowing for building strata with close probabilities of inclusion in cohort 1. Subsequently, new matched cohorts were formed by selecting for each cohort 1 individual a single nearest neighbor (with respect to the predicted propensity scores, 1:1 matching) from cohort 2 within a predefined window of ±0.005 around the propensity score of the cohort 1 individual. In case there were no such cohort 2 individuals, the affected individual from cohort 1 was not included in the matched population. A matched cohort 2 individual was excluded from further matching (matching without replacement). After this matching step, the created cohorts were close to indistinguishable based on the normalized mean differences, meaning that the distribution of their characteristics at index date was nearly identical, thus increasing the probability of an unconfounded comparison between cohorts (see Extended Data Fig. 6 for prematching and postmatching balance diagnostics for the primary comparisons and Supplementary Figs. 1–6 for all comparisons). We acknowledge that such a comparison can only account for measured confounders.

The above matching methodology was used in all instances^54^. Both XGBoost (for exposed cohorts) and LightGBM (for not-exposed cohorts) were used as the propensity-score estimator^55,56^.

For primary comparisons between RZV and ZVL, we reduced the strictness in index date matching compared to other comparisons through injection of Gaussian noise to index dates to increase the pool of individuals available for matching. We did this because the number of RZV recipients was much lower, due to the more recent licensure date of RZV (2017)^38^ compared to ZVL (2006)^57^ in the United States.

We additionally tested alternative analysis approaches, including inverse probability of treatment weighting (IPTW)^58^ and overlap weights (OW)^59^ where samples are not matched but weighted based on their propensity scores. We included the IPTW and OW methodologies to show consistency of our results. For OW, individuals included in the treatment cohort were weighted by *w* = (1 − ps) and individuals in the control cohort were weighted by *w* = ps, where ps refers to the propensity score obtained by the strategy outlined above. For IPTW, propensity scores were trimmed to the range (0.1, 0.9) by removing individuals with smaller or larger propensity scores following common practices^60^. Weights were then calculated as $w=\frac{1}{\mathrm{ps}}$ for the treatment and $w=\frac{1}{1-{\mathrm{ps}}}$ for the control cohort.

### Effect estimation

Cumulative hazard functions for each cohort were evaluated at defined time points postindex date using the Nelson–Aalen estimator. Equivalence was determined using a chi-squared test (*α* = 0.05) between the pairs of cohorts in terms of the difference of their cumulative hazard distributions at each evaluated time point^61^. Missing covariates were imputed using the multiple imputation by chained equations method^62^.

The choice of the Nelson–Aalen estimation strategy was motivated by the prior knowledge that efficacy of some of the evaluated exposures (in particular ZVL) substantially wanes over time. Hazard ratio estimation using a Cox proportional hazards model is made assuming that the hazard ratio remains constant over time, which is violated in our setting given the known waning of efficacy. The Nelson–Aalen-based methodology does not require a proportional hazards assumption and is therefore suited for this setting. Furthermore, we provide effect estimates at various time points, to capture the time-varying effect of vaccination on dementia and HZ risk.

The study design ensures that there are no systematic differences in follow-up times between the compared cohorts through censoring. Individuals who are no longer under observation (for example, have no later EHR records) are censored from the analysis, as defined in the Population and follow-up. In other words, these individuals are no longer considered ‘at risk’ and do not contribute to the result after their censoring event. An important consideration in real-world datasets is that systematic differences between censored individuals in the two cohorts may point to residual confounding. We addressed this risk by using the matching methodology outlined above, as well as additional analyses to quantify residual confounding (such as time shift validation).

### Software

The source code used for cohort definition, matching and data analysis was created using Python (version 3.9) and standard scientific computing and machine-learning libraries (scikit learn, polars, pandas, numpy, scipy). Dependencies were managed using Poetry. Data were plotted using matplotlib and seaborn in Python.

### Trademark statement

Shingrix is a trademark owned by or licensed to GSK. Zostavax is a trademark of Merck. Prevenar 13 is a trademark of Pfizer. Optum EHR is a registered trademark of Optum, Inc.

### Reporting summary

Further information on research design is available in the Nature Portfolio Reporting Summary linked to this article.

## Online content

Any methods, additional references, Nature Portfolio reporting summaries, source data, extended data, supplementary information, acknowledgements, peer review information; details of author contributions and competing interests; and statements of data and code availability are available at 10.1038/s41591-025-03972-5.

## Supplementary information

Supplementary Information Supplementary Tables 1–28 and Supplementary Figs. 1–8.

Reporting Summary

## Source data

Source Data Fig. 2 Prematching and postmatching characteristics of the cohorts.

Source Data Fig. 3 Prematching and postmatching characteristics of the cohorts.

Source Data Fig. 5 Prematching and postmatching characteristics of the cohorts.

Source Data Extended Data Fig. 1 Pre- and post-matching characteristics of the cohorts.

Source Data Extended Data Fig. 2 Pre- and post-matching characteristics of the cohorts.

Source Data Extended Data Fig. 3 Pre- and post-matching characteristics of the cohorts.

Source Data Extended Data Fig. 4 Pre- and post-matching characteristics of the cohorts.

## Figures and Tables

**Extended Data Table 1:**
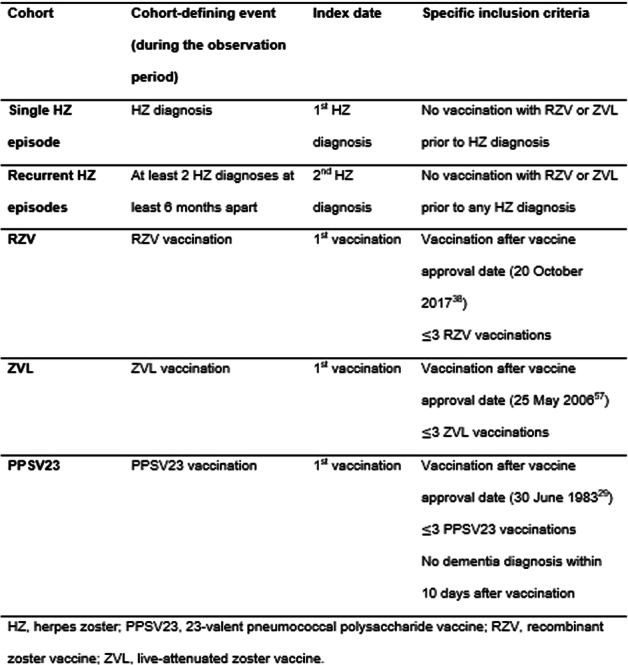
Cohort definitions

Individuals were included in a specific cohort if they had the respective cohort-defining event in the records and fulfilled the specific inclusion criteria. Index date was defined as the first record of the cohort-defining event except for recurrent HZ, for which the date of the second HZ episode was used.

HZ, herpes zoster; PPSV23, 23-valent pneumococcal polysaccharide vaccine; RZV, recombinant zoster vaccine; ZVL, live-attenuated zoster vaccine.

**Extended Data Table 2:**
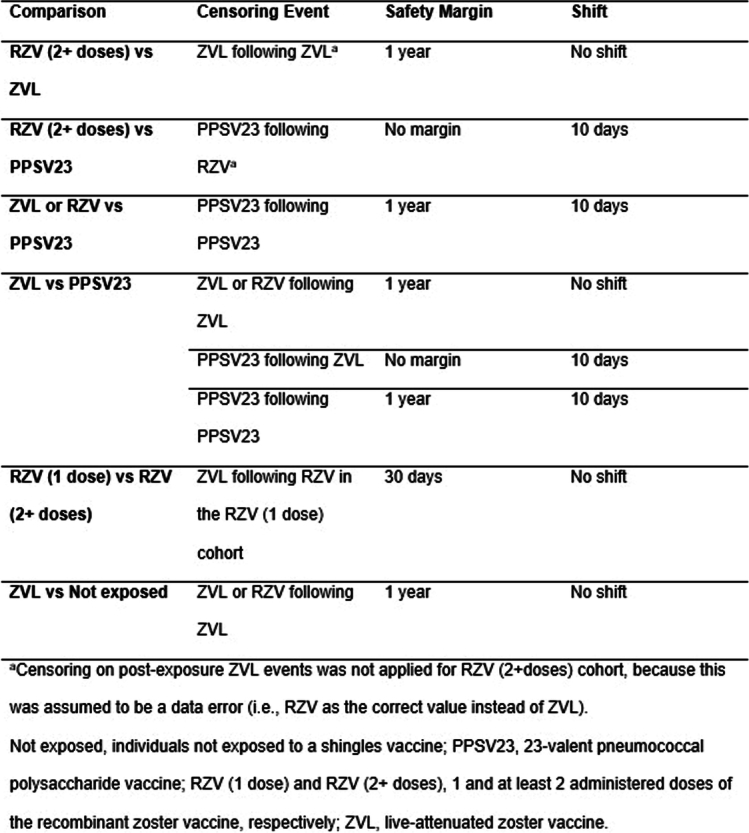
Safety margins and shifts applied for certain censoring events and the affected comparisons

For comparisons and censoring events not listed in the table, no safety margin and no shift were applied.

^a^Censoring on post-exposure ZVL events was not applied for RZV (2+ doses) cohort, because this was assumed to be a data error (that is, RZV as the correct value instead of ZVL).

NE, not exposed; PPSV23, 23-valent pneumococcal polysaccharide vaccine; RZV (1 dose) and RZV (2+ doses), 1 and at least 2 administered doses of the recombinant zoster vaccine, respectively; ZVL, live-attenuated zoster vaccine.

## Data Availability

The source data used for the present study were licensed from the Optum de-identified EHR database (https://www.optum.com/), with restrictions that do not allow for the data to be redistributed or made publicly available. However, for accredited researchers, the Optum de-identified EHR database is available for licensing at Optum, Inc. Data access may require a data-sharing agreement and may incur data access fees. Source data are provided with this paper.
