# Supplementary material for: Varicella-zoster virus reactivation and the risk of dementia
Source: Nat Med. 2025 Oct 6;31(12):4172–9. doi: 10.1038/s41591-025-03972-5 (PMC12705433; doi:10.1038/s41591-025-03972-5)
Supplement: Supplementary file 1 — Supplementary Tables 1–28 and Supplementary Figs. 1–8. [file 41591_2025_3972_MOESM1_ESM.pdf]

nature medicine

Supplementary information https://doi.org/10.1038/s41591-025-03972-5

Varicella-zoster virus reactivation and the risk of dementia

In the format provided by the authors and unedited

Supplementary Table 1 | Prescribed medications, identified by either National Drug Codes (NDC) or by their membership to a class of prescriptions as standardized in the Optum® EHR

Prescription	NDC or drug classes
Donepezil	00093073805, 00093073856, 00093073898, 00093073905, 00093073956, 00093073998, 00093540765, 00093540865, 00143974709, 00143974730, 00143974809, 00143974830, 00228452903, 00228452909, 00378102577, 00378102593, 00378514405, 00378514477, 00378514493, 00378514505, 00378514577, 00378514593, 00781527410, 00781527413, 00781527431, 00781527492, 00781527510, 00781527513, 00781527531, 00781527592, 00781527664, 00781527764, 00904624261, 00904624361, 00904635461, 00904635561, 00904640846, 00904640861, 00904640880, 00904640889, 00904640946, 00904640961, 00904640980, 00904640989, 00904647761, 00904647861, 12280029230, 12280029290, 12280036715, 12280036730, 12280036790, 13668010205, 13668010210, 13668010230, 13668010240, 13668010271, 13668010274, 13668010290, 13668010305, 13668010310, 13668010326, 13668010330, 13668010371, 13668010374, 13668010390, 16571077803, 16571077809, 16571077810, 16571077850, 16571077903, 16571077909, 16571077910, 16571077950, 24979000406, 24979000407, 29300025011,

	29300025087, 29300025111, 29300025187, 31722013910, 31722014010, 31722073705, 31722073730, 31722073790, 31722073805, 31722073830, 31722073890, 33342002707, 33342002710, 33342002715, 33342002744, 33342002807, 33342002810, 33342002815, 33342002844, 33342002907, 33342002960, 33342003007, 33342003060, 33342006107, 33342006110, 42254030430, 42254030530, 42254030590, 42291024690, 42291025490, 42291025590, 42543070201, 42543070205, 42543070210, 42543070230, 42543070290, 42543070301, 42543070305, 42543070310, 42543070330, 42543070390, 42582031109, 42582031130, 42582031209, 42582031230, 43063050090, 43063065990, 43547027503, 43547027509, 43547027511, 43547027603, 43547027609, 43547027611, 43547038203, 43547038209, 45963056004, 45963056008, 45963056030, 45963056104, 45963056108, 45963056130, 49848000590, 49848000690, 49884023209, 49884023211, 49999075330, 49999075430, 49999075490, 50090262700, 50090291000, 50090632900, 50436997401, 50436997501, 51079013830, 51079013856, 51079013930, 51079013956, 51407032030, 52343008930, 52343008990, 52343008999, 52343009030, 52343009090, 52343009099, 54569631000, 54569631100, 54868395200, 54868424500,

	54868620700, 54868620701, 54868620800, 55111030230, 55111030290, 55111035605, 55111035610, 55111035630, 55111035690, 55111035705, 55111035710, 55111035730, 55111035790, 55289015121, 55289015130, 58864088630, 58864089530, 59746032930, 59746032990, 59746033001, 59746033030, 59746033090, 59762024501, 59762024502, 59762024503, 59762024504, 59762024601, 59762024602, 59762024603, 59762024604, 59762025001, 59762025201, 60429032110, 60429032130, 60429032190, 60429032210, 60429032230, 60429032290, 60687017101, 60687017111, 60687018201, 60687018211, 60687029201, 60687029211, 60687030301, 60687030311, 62332009230, 62332009290, 62332009291, 62332009330, 62332009390, 62332009391, 62756044018, 62756044081, 62756044083, 62756044518, 62756044581, 62756044583, 62856024511, 62856024530, 62856024541, 62856024590, 62856024611, 62856024630, 62856024641, 62856024690, 62856024730, 62856024790, 62856083130, 62856083230, 63304012810, 63304012830, 63304012877, 63304012890, 63304012910, 63304012930, 63304012977, 63304012990, 63629111701, 63629111801, 63629363201, 63629848301, 63739064610, 63739065210, 63739065310, 63739066710, 63739066810, 63739067810,

	64380090704, 64380090705, 64380090707, 64380090804, 64380090807, 64679031101, 64679031103, 64679031105, 64679031201, 64679031203, 64679031205, 65038005501, 65038005503, 65038005601, 65038005603, 65862032530, 65862032590, 65862032599, 65862032630, 65862032690, 65862032699, 67544009215, 67544009217, 67544009288, 67544009289, 68084047701, 68084047711, 68084047801, 68084047811, 68084072501, 68084072511, 68084073401, 68084073411, 68180052706, 68180052709, 68382034606, 68382034706, 69150041503, 69150041509, 69150041510, 69150041603, 69150041609, 69150041610, 69452010813, 69452010819, 69452010830, 69452010913, 69452010919, 69452010930, 71093012701, 71093012703, 71093012705, 71093012706, 71093012801, 71093012803, 71093012805, 71093012806, 71205094800, 71205094830, 71205094855, 71205094860, 71205094890, 72189003130, 72189003160, 72189003190, 65038005510, 65038005610, 65038005504, 65038005604, 75929008603, 75929008703, 71335079201, 31722073801, 31722073701, 31722073731, 31722073831, 71335079202, 68071319809, 82009011905, 82009011910, 82009012005, 82009012010, 71335079203, 71209002004, 71335087602, 71209001904, 46708029510, 46708029530,

	46708029590, 46708029610, 68382034601, 68382034677, 68382034716, 65841072106, 65841072116, 65841072201, 65841074916, 65841075016, 62756019483, 00615831305, 00615831330, 00615831339, 65841072101, 68071152303, 62756019418, 68382034605, 68382034710, 68382034777, 29300024801, 50228013930, 70771132000, 70771132003, 68788820803, 68788820806, 68788820809, 55111035678, 46708029591, 65841072206, 65841075001, 65841075010, 71335206501, 71335206502, 71335206503, 33342002712, 33342002731, 33342002812, 33342002831, 33342002906, 33342002912, 33342003006, 33342003012, 33342006112, 71335041601, 62332009210, 71335058203, 62756019481, 68382034701, 46708029691, 65841072210, 65841072230, 65841074901, 65841074906, 70518045200, 65862032501, 63187040130, 71335209301, 71335209302, 71335209303, 69844003701, 69844003702, 69844003703, 69844003801, 69844003802, 69844003803, 71335087603, 65862032505, 46708029690, 65862032614, 71335041603, 46708029630, 65841072105, 65841072130, 65841072216, 70518166600, 65841074910, 65841074930, 71335202201, 71335202202, 71335202203, 71335090001, 71335090003, 00615795105, 00615795130, 00615795139, 50090353700, 55111035778,

	29300024813, 29300024913, 55154788200, 55154788300, 70771132005, 70771132009, 71209001901, 71209001911, 71335090002, 61919069290, 70771132004, 29300024819, 29300024910, 29300024810, 29300024919, 29300024901, 70771132001, 50228014030, 71335041602, 71335058201, 71335087601, 64380090805, 68382052105, 68382052177, 71209002001, 65841075006, 65862032601, 68382052101, 43063065930, 65841072205, 65862032510, 65862032605, 72189026590, 50228014010, 43547038210, 43547038250, 55111030205, 55111030210, 71209002011, 72162213600, 72162213603, 72162213605, 72162213609, 72162213700, 72162213703, 72162213705, 72162213709, 68382034610, 68382052106, 68382052110, 50228013910, 62332009310, 71335058202, 68382034616, 68382034705, 68382052116, 65841072110, 65841074905, 65841075005, 65841075030
Memantine	00121085005, 00121085040, 00378110391, 00378110491, 00378543593, 00378543677, 00378543693, 00378543793, 00378543877, 00378543893, 00456320014, 00456320212, 00456320511, 00456320560, 00456320563, 00456321011, 00456321060, 00456321063, 00456340029, 00456340733, 00456341411, 00456341433, 00456341463, 00456341490, 00456342133, 00456342811, 00456342833, 00456342863,

	00456342890, 00527122106, 00527122205, 00527122206, 00527194313, 00591387044, 00591387045, 00591387060, 00591387544, 00591387545, 00591387560, 00591390087, 00832111260, 00832111360, 00904650506, 00904650561, 00904650606, 00904650661, 00904673461, 00904673561, 00904673661, 00904673761, 10370034611, 10370034709, 10370034711, 10370034811, 10370034909, 10370034911, 12280028460, 12280038160, 13668022260, 13668022360, 13668057309, 13925054012, 16590076915, 16590076930, 16590076960, 16714095901, 16714096001, 16714096002, 16714096101, 16714096201, 16714096202, 21695016930, 21695016960, 21695023215, 21695023260, 27241007006, 27241007105, 27241007106, 29300017105, 29300017116, 29300017205, 29300017216, 31722080760, 31722080860, 33342006628, 33342029709, 33342029809, 33342029815, 35356010560, 39328055112, 42291055160, 42291055260, 42292000501, 42292000506, 42292000601, 42292000606, 43353016518, 43353016553, 43353017018, 43353089718, 43975024203, 43975024303, 43975026403, 43975026409, 43975026603, 43975026609, 47335032186, 47335032213, 47335032286, 49848000360, 49848000460, 49999080430, 49999080460, 50090443400, 50090443401, 50090443402,

	50090583200, 50090583201, 50090583202, 50090592600, 50090592601, 50090629101, 51407005430, 51407005530, 51407005590, 51407005630, 51407005730, 51407005790, 51407028460, 51407028560, 53746016930, 53746016960, 53746017360, 54569588500, 54868516100, 54868516101, 54868565400, 55111059660, 55111059705, 55111059760, 55289093730, 55289093760, 58864088730, 59651040430, 59651040530, 59651040590, 59651040630, 59651040730, 59651040790, 60505616205, 60505620803, 60505620903, 60505620909, 60505621003, 60505621103, 60505621109, 60687017311, 60687017357, 60687018411, 60687018457, 62135089560, 62135089660, 62135094337, 62332007531, 62332007560, 62332007591, 62332007631, 62332007642, 62332007660, 62332007671, 63629198201, 63629198301, 63629198401, 63629198501, 63629214201, 63629214301, 63629222201, 63629222301, 63629251101, 63629251201, 63629251301, 63629251401, 63629338301, 63629338302, 63629338303, 63629338304, 63629739701, 63629928601, 64679012102, 64679012103, 64679012202, 64679012203, 65162016906, 65162017306, 65162078203, 65162078303, 65162078309, 65162078403, 65162078503, 65162078509, 65862065260, 65862065360, 65862065399, 66105065003,

	66105065103, 68180022907, 68180023007, 68180024606, 68180024706, 68180024709, 68180024806, 68180024902, 68180024906, 68180024909, 68382054606, 68382054706, 68382054716, 68382054806, 68382054906, 68382054916, 70436005404, 70436005504, 70436005506, 70436005604, 70436005704, 70436005706, 71335173202, 71335190801, 71335190802, 71610001118, 72189029430, 72189029490, 72578000305, 72578000314, 72578000405, 72578000414, 72603011801, 72603011901, 72603011902, 72606051402, 72606051404, 72606051502, 72606051504, 75839042501, 55111059679, 55111059730, 55111059630, 00615831905, 00615831939, 00615819239, 70518329700, 55111059605, 55111059701, 55111059779, 55154415100, 55111059678, 55111059778, 55111059601, 55154763700, 71335160301, 71335160302, 47335003383, 47335032108, 47335032208, 47335032283, 62332007530, 63629195701, 47335032218, 68382054616, 68382054816, 68382054977, 47335003283, 47335003381, 46708045142, 46708045271, 00615826439, 62332007542, 46708045230, 70771111900, 68382054777, 71335160303, 71335160304, 70771132404, 71335034602, 59651040490, 59651040690, 59651040828, 62332007510, 46708045120, 46708045231, 27241007005, 65162078250,

	46708045160, 46708045260, 50090640000, 50090640001, 50090640002, 62332007520, 71335189901, 71335189902, 71335189903, 71335189904, 70771111906, 65862065299, 65862065303, 70771112004, 70518333800, 71335186301, 71335186302, 71335186303, 72578000301, 72578000310, 72578000377, 72578000401, 72578000410, 72578000477, 47335003181, 47335003281, 53746017330, 65862065278, 65862065378, 65862065203, 47335003118, 47335003318, 71335201301, 71335201302, 70771112001, 70771132203, 53746017310, 64380077430, 55154266600, 55154266700, 63629195601, 46708045191, 46708045242, 00527122105, 00527122110, 00527122210, 31722080702, 53746016910, 70771132103, 70771132209, 70771132409, 71335186304, 64380077401, 70771132303, 70771132403, 63629739702, 63629739703, 63629739704, 70771112000, 70771132104, 70771111901, 70771112005, 70771112006, 64380077403, 70771111904, 70771111905, 50090629100, 71335034601, 70771132304, 68788773003, 68788773006, 68788773009, 71335179801, 71335179802, 71335173201, 47335032188, 47335032288, 62332007620, 65162078350, 65162078450, 70771132109, 70771132309, 71034000730, 71034000830, 71034000930, 31722080732, 31722080802, 31722080832,

	71335190803, 71335190804, 47335003218, 47335003481, 64380074503, 64380074530, 68382054677, 33342029712, 33342029715, 33342029812, 47335003418, 52605007110, 52605007113, 52605007116, 52605007210, 62332007610, 52605007213, 52605007216, 65162078409, 65162078550, 46708045110, 46708045130, 46708045210, 46708045220, 65162078209, 62332007630, 46708045131, 68382054877, 47335003183, 70771132204, 47335003483, 47335032118
Donepezil_memantine	00456120730, 00456121430, 00456122130, 00456122830, 00456122929, 69238124709, 69238124703, 69238124803, 69238124809, 69238155203, 69238155209, 69238155303, 69238155309, 00456121411, 00456122811
Galantamine	00054009021, 00054009121, 00054009221, 00054013749, 00115112008, 00115112108, 00115112208, 00378272191, 00378272291, 00378272391, 00378810491, 00378810593, 00378810693, 00378810793, 00378810891, 00378811291, 00555013809, 00555013909, 00555014009, 00555102001, 00555102101, 00555102201, 00591349630, 00591349730, 00591349830, 00904710404, 10147088106, 10147088206, 10147088306, 10147089103, 10147089203, 10147089303, 12280029160, 21695018430, 21695059130, 21695078730, 43353090505, 43353098405, 47335083583, 47335083683,

	47335083783, 50458038730, 50458038830, 50458038930, 50458039060, 50458039160, 50458039260, 50458039660, 50458039760, 50458039860, 50458039910, 50458049010, 51079046901, 51079046903, 51079047001, 51079047003, 51079047101, 51079047103, 51079085201, 51079085203, 51079085301, 51079085303, 51079085401, 51079085403, 54868503200, 54868511100, 54868545300, 55111040760, 55111040860, 55111040960, 57237004960, 57237005060, 57237005160, 59762000801, 59762000901, 59762001001, 60505254206, 60505254306, 60505254406, 63629236301, 63629236401, 63629236501, 63739070833, 63739099933, 65862045860, 65862045960, 65862046060, 65862074430, 65862074530, 65862074630, 68084049211, 68084049221, 68084072911, 68084072921, 68382017714, 68382017814, 68382017914, 70436000406, 70436000506, 70436000606, 68382017910, 68382017701, 68382017777, 68382017901, 65841075501, 65841075514, 65862045899, 65862045971, 65862074599, 68382017877, 68382017810, 65862074405, 65862074501, 65862074505, 65862074590, 65862045999, 65841075610, 65862074601, 65862074690, 65862074699, 65841075714, 65862045819, 65862046049, 65862046099, 65862074605, 65862074401, 65862074499, 65841075577,

	65841075677, 65841075777, 65841075701, 65841075510, 65841075601, 68382017977, 68382017710, 65841075614, 65841075710, 65862074490, 68382017801, 47335083508, 47335083688, 47335083608, 47335083618, 47335083718, 72162144603, 72162144703, 72162144803, 47335083708, 47335083518, 47335083588, 47335083788
Rivastigmine	00078032306, 00078032315, 00078032344, 00078032361, 00078032406, 00078032415, 00078032444, 00078032461, 00078032506, 00078032515, 00078032544, 00078032561, 00078032606, 00078032615, 00078032644, 00078032661, 00078033931, 00078050115, 00078050161, 00078050215, 00078050261, 00078050315, 00078050361, 00378907016, 00378907093, 00378907116, 00378907193, 00378907216, 00378907293, 00591320860, 00591320960, 00591321060, 00591321160, 00781261406, 00781261413, 00781261460, 00781261506, 00781261513, 00781261560, 00781261606, 00781261613, 00781261660, 00781261706, 00781261713, 00781261760, 00781730431, 00781730458, 00781730931, 00781730958, 00781731331, 00781731358, 00904658761, 00904710761, 12280038960, 16714011501, 16714011502, 16714011601, 16714011602, 16714011701, 16714011702, 21695035730, 33342008909, 33342008915, 33342009009,

	33342009015, 33342009109, 33342009115, 33342009209, 33342009215, 35356039430, 47781030403, 47781030411, 47781030503, 47781030511, 47781040503, 47781040511, 51991079306, 51991079406, 51991079506, 51991079606, 51991089730, 51991089799, 51991089830, 51991089899, 51991089930, 51991089999, 54868451200, 54868451201, 54868524000, 54868533900, 54868583900, 54868595400, 54868607000, 54868614500, 55111035205, 55111035260, 55111035305, 55111035360, 55111035405, 55111035460, 55111035505, 55111035560, 60429039360, 60429039460, 60429039560, 60429039660, 60505322006, 60505322106, 60505322206, 60505322306, 60687057401, 60687057411, 62135090060, 62135090160, 62135090260, 62135090360, 62332006360, 62332006460, 62332006560, 62332006660, 62756014513, 62756014586, 62756014613, 62756014686, 62756014713, 62756014786, 62756014813, 62756014886, 63629206401, 63629206501, 63629206601, 63629880701, 63629884601, 63739057610, 63739057710, 63739057810, 63739057910, 65162074934, 65162082534, 65162082634, 65862064860, 65862064960, 65862065060, 65862065160, 68084055001, 68084055011, 70710119601, 70710119607, 70710119701, 70710119707, 70710119801, 70710119807,

	72241001103, 72241001203, 72241001303, 72241001403, 75834013305, 75834013360, 75834013405, 75834013460, 75834013505, 75834013560, 75834013605, 75834013660, 51956000107, 51956000207, 51956000307, 62756014761, 71209001403, 71209001303, 71209001310, 55111035201, 46708006471, 46708006671, 65862065005, 55111035501, 33342008906, 33342008912, 33342009006, 33342009012, 33342009106, 33342009112, 33342009206, 33342009212, 62756014561, 55111035230, 46708006310, 46708006410, 46708006491, 46708006630, 65862065078, 65862064978, 46708006360, 46708006391, 55111035401, 65862064905, 65862064878, 65862065105, 65862065178, 46708006510, 62332006310, 62332006330, 62332006371, 62332006391, 62332006410, 62332006430, 62332006471, 62332006491, 62332006510, 62332006530, 62332006571, 62332006591, 62332006610, 62332006630, 62332006671, 62332006691, 55111035330, 55111035430, 55111035530, 71209001503, 46708006560, 46708006660, 72241001110, 71209001510, 55111035301, 62756014661, 62756014861, 71209001210, 46708006371, 46708006430, 46708006591, 46708006691, 65862064805, 46708006610, 46708006330, 46708006460, 46708006530, 46708006571, 71209001203, 71209001410,

	72241001210, 72241001310, 72241001410, 72162160803, 72855010001, 72855010101, 72855010201
Antidepressants	Selective serotonin reuptake inhibitor antidepressants, Tricyclic antidepressants, Antidepressants; miscellaneous, Serotonin and norepinephrine reuptake inhibitors
Antipsychotics	Antipsychotics, Psychotherapeutic combinations
Angiotensin_antagonist	Angiotensin II receptor antagonists (ARB), Angiotensinconverting enzyme (ACE) inhibitors
Adrenergics	Centrally-acting antiadrenergic agents, Alpha-1 adrenergic antagonists, Beta-adrenergic antagonists (beta blockers) without intrinsic sympathomimetic activity (ISA), Beta-adrenergic antagonists (beta blockers) with ISA, Beta-adrenergic antagonists; Glaucoma treatment, Peripherally-acting antiadrenergics, Alpha- and beta-adrenergic antagonists, Alpha-1 adrenergic antagonists for benign prostatic hyperplasia
Non-steroidal anti-inflammatory drugs (NSAIDs)	NSAIDs; cyclooxygenase-2 (COX-2) inhibitors, NSAID agents, NSAID combinations; various, Ophthalmic non-steroidal anti-inflammatory agents, Ophthalmic steroid and non-steroidal anti-inflammatory agents
Antidiabetics	Antidiabetic agents; thiazolidinediones, Antidiabetic agents; biguanides, Glucagon-like peptide 1 receptor agonists; and other miscellaneous diabetes agents, Insulin and other

	antidiabetic medication, Antidiabetic combination agents, Diabetic supplies, Antidiabetic agents; dipeptidyl peptidase inhibitors, Antidiabetic agents; sulfonylurea, Antidiabetic agents; alpha-glucosidase inhibitors, Antidiabetic agents; amylin analogs, Antidiabetic agents; meglitinides, Insulin
Antihypertensives	Antihypertensive agents; miscellaneous, Antihypertensive combinations; ARB and thiazide diuretic, Antihypertensive combinations; beta blocker and thiazide diuretic, Antihypertensive combinations; ARB and calcium channel blocker; with or without diuretic, Antihypertensive combinations; direct renin inhibitor and various, Hypertensive emergency agents, Antihypertensive combinations; ACE inhibitor & thiazide diuretic, Antihypertensive combinations; miscellaneous, Antihypertensive combinations; ACE inhibitor and calcium channel blocker, Calcium channel antagonists (calcium channel blockers), Nitrates
Anticholinergics	Urinary anticholinergics, Gastrointestinal anticholinergics, Upper respiratory combinations; decongestant; antihistamine and anticholinergic, Gastrointestinal anticholinergic and antispasmodic combinations, Antivertigo agents; anticholinergics, Inhaled anticholinergic agents

Antivirals	Hepatitis B antivirals; reverse transcriptase inhibitors, Ophthalmic antiviral agents, human immunodeficiency virus (HIV) antiviral combinations; or systemic exposure boosters, Hepatitis C antivirals, HIV antivirals; cellular chemokine receptor antagonists, Antivirals; topical, HIV antivirals; monoclonal antibodies, HIV antivirals; reverse transcriptase inhibitors, HIV antivirals; integrase inhibitors, HIV antivirals; miscellaneous, Non-HIV antivirals, HIV antivirals; protease inhibitors, Viral vaccines, HIV antivirals; fusion inhibitors
Glucocorticoids	Systemic glucocorticoids
Statins	Hydroxymethylglutaryl-CoA (HMG-CoA) reductase inhibitor combinations; statin & various, HMG-CoA reductase inhibitors (statins)
Nutrition	Vitamin B-12 products, Nutritional products; miscellaneous, Nutritional supplement supplies, Vitamin K products, Minerals and electrolytes; oral, Vitamin B and derivatives, Multivitamin and mineral combinations, Vitamin A analogs, Vitamin C and derivatives, Vitamin D analogs, Vitamin E analogs, Herbal nutritional products, Intravenous nutritional therapy; electrolyte; trace element; metal; vitamin; alone or combinations, Intravenous nutritional therapy; protein or energy substrates,

	Calcium supplements; oral, Iron, Iron combination products; miscellaneous
Cancer_treatment	Cytoprotective agents; chemotherapeutic, Biological response modifiers; chemotherapeutic, Antiandrogens; chemotherapeutic, Vaccines; chemotherapeutic, Antibody drug conjugates; chemotherapeutic, Antiestrogens; chemotherapeutic, Chemotherapy adjuncts, Gonadotropin releasing hormones; chemotherapeutic, Progestins; chemotherapeutic, Photochemotherapeutic agents, Monoclonal antibodies; chemotherapeutic, Aromatase inhibitors; chemotherapeutic, Kinase inhibitors; chemotherapeutic, Enzymes; chemotherapeutic, Antineoplastic and chemotherapy adjunct combinations, Alkylating agents; miscellaneous, Alkylating agents; nitrogen mustards, Alkylating agents; nitrosoureas, Mitotic inhibitors; epothilones, Mitotic inhibitors; halichondrin b analogs, Mitotic inhibitors; podophyllotoxin derivatives, Mitotic inhibitors; taxanes, Mitotic inhibitors; vinca alkaloids, Antimetabolites, Oncolytic virotherapies, Topoisomerase inhibitors
Steroids	Corticosteroid combinations; topical, Corticosteroids; topical, Inhaled corticosteroids, Ophthalmic corticosteroids, Ophthalmic steroid & antibiotic combinations, Otic steroid & antibiotic

	combinations, Intranasal steroids, Anabolic steroids, Ophthalmic steroid & non-steroidal anti-inflammatory agent, Estrogen and androgen combinations, Estrogen and nitrogen mustard combinations, Estrogen and progestin combinations, Estrogens, Natural androgens, Synthetic androgens, Vaginal estrogens, Progestins, Vaginal progesterones, Mineralocorticoids
Antibacterial	Anthracycline antibiotics, Antibiotics; miscellaneous, Antineoplastic antibiotics; miscellaneous, Beta-lactam antibiotics; miscellaneous, Ophthalmic antibiotic combinations, Ophthalmic antibiotics; miscellaneous, Ophthalmic steroid and antibiotic combinations, Otic antibiotics, Otic steroid and antibiotic combinations, Penicillins, Antibacterial acne products, Antibacterial combinations; topical, Antibacterials; topical, Bacterial vaccines, Cephalosporins, Macrolides, Ophthalmic quinolones, Quinolones, Sulfonamide combinations, Sulfonamides, Tetracyclines, Ophthalmic aminoglycosides, Aminoglycosides
Contraceptives	Contraceptive hormones, Contraceptive implants or injections or systems, Contraceptive supplies
Antiallergics	Ophthalmic antiallergy agents, Antihistamine combination products; non-sedating, Antihistamines; non-sedating, Antihistamines; sedating, Antihistamines; topical, Ophthalmic

	antihistamines, Ophthalmic decongestant and antihistamine combinations, Upper respiratory combinations; antihistamine and analgesic, Upper respiratory combinations; decongestant; antihistamine and anticholinergic, Upper respiratory combinations; decongestant and antihistamine, Allergenic extracts
Analgesics	Analgesics; miscellaneous, Analgesics; topical, Centrally-acting analgesics, Narcotic and analgesic combinations, Narcotic agonist analgesics, Non-narcotic analgesic combinations, Otic anesthetic and analgesic combinations, Oral cavity topical analgesic or protectant products, Skeletal muscle relaxant and analgesic combinations, Upper respiratory combinations; antihistamine and analgesic, Upper respiratory combinations; decongestant and analgesic, Upper respiratory combinations; decongestant; antihistamine and analgesic, Acetaminophen
Antiarrhythmics	Antiarrhythmics; class I, Antiarrhythmics; class III, Antiarrhythmics; class IV, Antiarrhythmics; no Vaughan Williams Class distinction
Antifungal	Antifungal combinations; topical, Antifungals, Antifungals; topical, Ophthalmic antifungal agents, Oral cavity antifungals, Vaginal antifungals

Diuretic	Diuretic combinations, Diuretics; miscellaneous, Thiazides and related agents, Loop diuretics, Osmotic diuretics, Potassium-sparing diuretics
Vassopressin	Vasopressin derivatives, Vasopressin-receptor antagonists
Antihypotensives	Vasopressors; circulatory shock
Diagnostics	In vivo diagnostic; adrenal cortical function, In vivo diagnostic; cardiovascular function, In vivo diagnostic; drug hypersensitivity, In vivo diagnostic; gall bladder function, In vivo diagnostic; gastroenterology, In vivo diagnostic; hepatobiliary function, In vivo diagnostic; infectious disease, In vivo diagnostic; miscellaneous, In vivo diagnostic; neurology, In vivo diagnostic; pancreatic function, In vivo diagnostic; pituitary function, In vivo diagnostic; renal function, In vivo diagnostic; roentgenography, In vivo diagnostic; skeletal imaging, In vivo diagnostic; thyroid function, In vivo diagnostics; biological, Miscellaneous diagnostics or supplies or devices
Antiplatelets	Antiplatelet agents; glycoprotein llb/IIIa inhibitors, Antiplatelet combinations, Antiplatelets or aggregation inhibitors, Factor Xa inhibitors, Heparins or glycosaminoglycans, Anticoagulants; coumarin derivatives
Sympathomimetics	Sympathomimetics, Sympathomimetics; glaucoma treatment

Antiemetics	Antiemetic agents or combinations; miscellaneous, Antiemetic agents; 5-hydroxytryptamine-3 (5HT-3) antagonists, Antiemetic agents; antidopaminergics
Muscle_relaxants	Skeletal muscle relaxants; centrally-acting, Skeletal muscle relaxants; direct-acting
Laxatives	Bulk-producing laxatives, Hyperosmolar laxatives, Irritant or stimulant laxatives, Laxative & stool softener combinations, Laxative products; miscellaneous, Saline laxatives, Fecal softeners
Anxiolytics	Anxiolytics; benzodiazepine, Anxiolytics; miscellaneous
Antiepileptics	Anticonvulsants; hydantoin, Anticonvulsants; miscellaneous, Anticonvulsants; succinimide
Salicylates	Salicylates
Antitussives	Antitussives, Cough combinations; expectorant, Cough combinations; narcotic antitussive, Cough combinations; nonnarcotic antitussive
Ulcer_treatment	Histamine H2 antagonists
Amebicides	Amebicides
Antiasthmatics	Antiasthmatic combinations; xanthine and expectorant, Leukotrienereceptor antagonists
Migraine	Migraine agents; ergot derivatives, Migraine agents; serotonin receptor agonists, Migraine combinations

Proton pump inhibitors	Proton pump inhibitors
Bowel_evacuants	Bowel evacuant combinations, Bowel evacuants
Glaucoma	Carbonic anhydrase inhibitors; glaucoma treatment, Beta-adrenergic antagonists; glaucoma treatment, Cholinesterase inhibitors; glaucoma treatment, Glaucoma drug therapy; miscellaneous, Miotics; glaucoma treatment, Prostaglandin agonists; glaucoma treatment, Sympathomimetics; glaucoma treatment
Skin_care	Retinoid combinations, Retinoids and derivatives, Tar-containing dermatologicals, Therapeutic skin cleansers
Osteoporosis_treatment	Bone resorption inhibitors; bisphosphonate, Bone resorption inhibitors; hormone or monoclonal antibody, Bone resorption inhibitors; miscellaneous

Supplementary Table 2 | Key pre- and post-matching characteristics of HZ multiple episodes and HZ single episode cohorts (dementia, all)

	Pre-Matching						Post-Matching				
	HZ multiple episodes		HZ single episode				HZ multiple episodes	HZ single episode			
**Covariate**	% missing	Mean	% missing	Mean	Difference in Mean	NMD	Mean	Mean	Difference in Mean	NMD	p
Age at exposure	0.0	66.7062	0.0	65.1826	1.5237	0.1558	66.69	66.8037	-0.1137	-0.0117	0.0038
Body mass index	23.1	29.2552	15.1	29.5415	-0.2863	-0.0428	29.2612	29.3127	-0.0444	-0.0077	0.3793
gender_female	0.0	0.6677	0.1	0.6326	0.0351	0.0737	0.667	0.6626	0.0044	0.0094	0.0385
race_Caucasian	0.0	0.871	0.0	0.8502	0.0208	0.0602	0.8706	0.8591	0.0115	0.0337	0.0
race_Other/Unknown	0.0	0.0465	0.0	0.0649	-0.0184	-0.0804	0.0462	0.055	-0.0088	-0.0401	0.0
race_African American	0.0	0.0653	0.0	0.0657	-0.0004	-0.0016	0.066	0.0668	-0.0009	-0.0035	0.4388
race_Asian	0.0	0.0172	0.0	0.0192	-0.002	-0.0152	0.0172	0.0191	-0.0019	-0.014	0.0019
has_chickenpox_diagnosis	0.0	0.002	0.0	0.0017	0.0003	0.0065	0.002	0.0017	0.0003	0.0075	0.0999
has_diabetes_diagnosis	0.0	0.1907	0.0	0.1973	-0.0066	-0.0167	0.1927	0.1972	-0.0045	-0.0113	0.0125
has_depression_diagnosis	0.0	0.16	0.0	0.1721	-0.0121	-0.0326	0.1624	0.1644	-0.002	-0.0053	0.239
has_stroke_diagnosis	0.0	0.0263	0.0	0.0304	-0.0041	-0.0248	0.0267	0.0292	-0.0025	-0.0153	0.0008

Covariate	Pre-Matching						Post-Matching				
	HZ multiple episodes		HZ single episode		Difference in Mean	NMD	HZ multiple episodes	HZ single episode	Difference in Mean	NMD	p
	% missing	Mean	% missing	Mean			Mean	Mean			
has_hypertension_diagnosis	0.0	0.4986	0.0	0.5015	-0.0029	-0.0057	0.5037	0.5076	-0.0039	-0.0078	0.0868
has_atherosclerosis_diagnosis	0.0	0.0447	0.0	0.0452	-0.0005	-0.0023	0.0451	0.047	-0.0019	-0.0093	0.0408
has_ischemic heart disease_diagnosis	0.0	0.1629	0.0	0.1607	0.0023	0.0062	0.1648	0.1668	-0.002	-0.0053	0.2397
has_chronic obstructive pulmonary disease_diagnosis	0.0	0.1147	0.0	0.1066	0.0081	0.0257	0.1161	0.1198	-0.0038	-0.0117	0.0101
has_obesity_diagnosis	0.0	0.1491	0.0	0.1911	-0.0421	-0.1121	0.1517	0.1522	-0.0005	-0.0014	0.7494
has_herpes_simplex_diagnosis	0.0	0.03	0.0	0.0261	0.0039	0.0237	0.0305	0.0274	0.0031	0.0183	0.0001
has_non-steroidal anti-inflammatory drugs_first	0.0	0.2512	0.0	0.2974	-0.0462	-0.1037	0.2553	0.2588	-0.0035	-0.0079	0.0755
has_antivirals_first	0.0	0.1778	0.0	0.1773	0.0005	0.0014	0.18	0.1823	-0.0023	-0.0061	0.1817
has_glucocorticoids_first	0.0	0.2644	0.0	0.2967	-0.0322	-0.0718	0.2688	0.2634	0.0054	0.0122	0.0069

A normalized mean difference (NMD) greater than 0.1 indicates a potential imbalance between the cohorts. Covariates with NMD > 0.1 pre-matching are highlighted by red color of their respective pre-matching NMD.

Supplementary Table 3 | Key pre- and post-matching characteristics of ZVL and PPSV23 cohorts (dementia, all)

	Pre-Matching						Post-Matching				
	ZVL		PPSV23		Difference		ZVL	PPSV23	Difference		
**Covariate**	% missing	Mean	% missing	Mean	in Mean	NMD	Mean	Mean	in Mean	NMD	p
Age at exposure	0.0	65.1409	0.0	66.271	-1.1301	-0.1489	66.4604	66.309	0.1514	0.0212	0.0
Body mass index	36.7	29.2739	32.3	30.124	-0.8501	-0.1278	29.7741	29.7465	0.0286	0.0043	0.1961
gender_Female	0.0	0.5825	0.0	0.5445	0.038	0.0767	0.5707	0.5712	-0.0005	-0.001	0.5439
race_Caucasian	0.0	0.9123	0.0	0.832	0.0803	0.2423	0.9011	0.9035	-0.0024	-0.0081	0.0
race_Other/Unknown	0.0	0.0409	0.0	0.0623	-0.0214	-0.097	0.0462	0.0457	0.0005	0.0023	0.1801
race_African American	0.0	0.033	0.0	0.0873	-0.0543	-0.23	0.039	0.0371	0.0019	0.01	0.0
race_Asian	0.0	0.0138	0.0	0.0184	-0.0046	-0.0364	0.0137	0.0137	0.0	0.0001	0.9421
has_chickenpox_diagnosis	0.0	0.0009	0.0	0.0006	0.0003	0.0111	0.0006	0.0006	0.0	0.0017	0.323
has_diabetes_diagnosis	0.0	0.1041	0.0	0.2146	-0.1105	-0.3054	0.1269	0.1231	0.0038	0.0116	0.0
has_depression_diagnosis	0.0	0.0985	0.0	0.1337	-0.0351	-0.1098	0.087	0.0857	0.0012	0.0044	0.0095
has_stroke_diagnosis	0.0	0.0104	0.0	0.0275	-0.0172	-0.1263	0.0117	0.0113	0.0004	0.0038	0.0246
has_hypertension_diagnosis	0.0	0.3639	0.0	0.4644	-0.1005	-0.2051	0.3461	0.3405	0.0056	0.0119	0.0
has_atherosclerosis_diagnosis	0.0	0.0163	0.0	0.0357	-0.0194	-0.122	0.018	0.0174	0.0006	0.0042	0.0128

Covariate	Pre-Matching						Post-Matching				
	ZVL		PPSV23		Difference in Mean	NMD	ZVL	PPSV23	Difference in Mean	NMD	p
	% missing	Mean	% missing	Mean			Mean	Mean			
has_ischemic heart disease_diagnosis	0.0	0.0814	0.0	0.1497	-0.0684	-0.2151	0.0915	0.0889	0.0026	0.0091	0.0
has_chronic obstructive pulmonary disease_diagnosis	0.0	0.0349	0.0	0.1049	-0.07	-0.277	0.045	0.043	0.002	0.0097	0.0
has_obesity_diagnosis	0.0	0.1034	0.0	0.1593	-0.0559	-0.1661	0.0929	0.0917	0.0012	0.004	0.0177
has_herpes_simplex_diagnosis	0.0	0.0173	0.0	0.013	0.0043	0.035	0.0112	0.0115	-0.0003	-0.0028	0.099
has_herpes_zoster_diagnosis	0.0	0.0253	0.0	0.0214	0.0039	0.0257	0.0197	0.0199	-0.0002	-0.0011	0.501
has_non-steroidal anti-inflammatory drugs_first	0.0	0.2001	0.0	0.2057	-0.0055	-0.0138	0.1602	0.1597	0.0006	0.0015	0.3658
has_antivirals_first	0.0	0.155	0.0	0.1075	0.0475	0.1409	0.0954	0.0974	-0.002	-0.0068	0.0
has_glucocorticoids_first	0.0	0.1537	0.0	0.1885	-0.0348	-0.0925	0.1306	0.1302	0.0005	0.0014	0.4207

A normalized mean difference (NMD) greater than 0.1 indicates a potential imbalance between the cohorts. Covariates with NMD > 0.1 pre-matching are highlighted by red color of their respective pre-matching NMD. Covariates with NMD > 0.1 post-matching are highlighted by red background color of the corresponding row.

PPSV23, recipients of at least 1 dose of the 23-valent pneumococcal polysaccharide vaccine; ZVL, recipients of at least 1 dose of the live-attenuated zoster vaccine (*Zostavax*, Merck).

Supplementary Table 4 | Key pre- and post-matching characteristics of RZV (2+ doses) and PPSV23 cohorts (dementia, all)

Covariate	Pre-Matching						Post-Matching				
	RZV (2+ doses)		PPSV23		Difference in Mean	NMD	RZV (2+ doses)	PPSV23	Difference in Mean	NMD	P-value
	% missing	Mean	% missing	Mean			Mean	Mean			
Age at exposure	0.0	62.0799	0.0	66.5783	-4.4985	-0.5504	65.5469	65.1898	0.3571	0.0404	0.0
Body mass index	13.0	29.2553	31.9	30.0363	-0.781	-0.1178	30.0797	30.0595	0.0376	0.003	0.1471
gender_Female	0.0	0.5761	0.0	0.5482	0.0279	0.0562	0.5547	0.5567	-0.002	-0.004	0.1716
race_Caucasian	0.0	0.8597	0.0	0.8432	0.0165	0.0465	0.8399	0.8409	-0.001	-0.0027	0.3493
race_African American	0.0	0.0466	0.0	0.0809	-0.0343	-0.1405	0.0624	0.0615	0.001	0.004	0.1678
race_Other/Unknown	0.0	0.0604	0.0	0.0585	0.0018	0.0077	0.0703	0.069	0.0013	0.0053	0.0711
race_Asian	0.0	0.0333	0.0	0.0174	0.0159	0.1013	0.0273	0.0286	-0.0013	-0.0079	0.0065
has_chickenpox_diagnosis	0.0	0.0018	0.0	0.0007	0.0011	0.0304	0.0013	0.0012	0.0001	0.0016	0.5916
has_diabetes_diagnosis	0.0	0.1168	0.0	0.2086	-0.0918	-0.2506	0.2153	0.2081	0.0072	0.0177	0.0
has_depression_diagnosis	0.0	0.17	0.0	0.133	0.037	0.1034	0.1778	0.1784	-0.0006	-0.0016	0.5929
has_stroke_diagnosis	0.0	0.0173	0.0	0.0266	-0.0093	-0.0636	0.0264	0.0255	0.0009	0.0056	0.0564

Covariate	Pre-Matching						Post-Matching				
	RZV (2+ doses)		PPSV23		Difference in Mean	NMD	RZV (2+ doses)	PPSV23	Difference in Mean	NMD	P-value
	% missing	Mean	% missing	Mean			Mean	Mean			
has_hypertension_diagnosis	0.0	0.4499	0.0	0.4677	-0.0178	-0.0358	0.529	0.5229	0.006	0.0121	0.0
has_atherosclerosis_diagnosis	0.0	0.026	0.0	0.0355	-0.0095	-0.0548	0.0396	0.0379	0.0017	0.0088	0.0025
has_ischemic heart disease_diagnosis	0.0	0.0984	0.0	0.1482	-0.0499	-0.1521	0.1484	0.1436	0.0048	0.0135	0.0
has_chronic obstructive pulmonary disease_diagnosis	0.0	0.04	0.0	0.1006	-0.0606	-0.2387	0.0778	0.0743	0.0035	0.0133	0.0
has_obesity_diagnosis	0.0	0.2684	0.0	0.1564	0.1121	0.2766	0.2828	0.2825	0.0002	0.0005	0.8584
has_herpes_simplex_diagnosis	0.0	0.0427	0.0	0.0137	0.0291	0.1763	0.0294	0.0301	-0.0008	-0.0046	0.1151
has_herpes_zoster_diagnosis	0.0	0.0452	0.0	0.0222	0.023	0.1277	0.0386	0.039	-0.0003	-0.0018	0.5454
has_non-steroidal anti-inflammatory drugs_first	0.0	0.3531	0.0	0.2102	0.1429	0.3217	0.3282	0.3306	-0.0024	-0.005	0.0872

Covariate	Pre-Matching						Post-Matching				
	RZV (2+ doses)		PPSV23				RZV (2+ doses)	PPSV23			
	% missing	Mean	% missing	Mean	Difference in Mean	NMD	Mean	Mean	Difference in Mean	NMD	P-value
has_antivirals_first	0.0	0.2705	0.0	0.117	0.1535	0.396	0.2217	0.2219	-0.0002	-0.0005	0.8741
has_glucocorticoids_first	0.0	0.3214	0.0	0.1909	0.1305	0.3023	0.3109	0.3145	-0.0036	-0.0078	0.0076

A normalized mean difference (NMD) greater than 0.1 indicates a potential imbalance between the cohorts. Covariates with NMD > 0.1 pre-matching are highlighted by red color of their respective pre-matching NMD. Covariates with NMD > 0.1 post-matching are highlighted by red background color of the corresponding row.

PPSV23, recipients of at least 1 dose of the 23-valent pneumococcal polysaccharide vaccine; RZV (2+ doses), recipients of at least 2 doses of the recombinant zoster vaccine (*Shingrix*, GSK).

Supplementary Table 5 | Key pre- and post-matching characteristics of RZV (2+ doses) and ZVL cohorts (dementia, all)

	Pre-Matching						Post-Matching				
	RZV (2+ doses)		ZVL				RZV (2+ doses)	ZVL			
**Covariate**	% missing	Mean	% missing	Mean	Difference in Mean	NMD	Mean	Mean	Difference in Mean	NMD	p
Age at exposure	0.0	63.6279	0.0	66.1757	-2.5479	-0.3235	65.0251	64.7587	0.2665	0.0342	0.0
Body mass index	12.1	29.7542	33.8	29.6345	0.1197	0.0186	29.7261	29.6597	0.041	0.0104	0.3052
gender_Female	0.0	0.5674	0.0	0.5731	-0.0057	-0.0115	0.5606	0.5606	0.0	0.0	0.9952
gender_Male	0.0	0.4323	0.0	0.4267	0.0056	0.0114	0.4392	0.4391	0.0001	0.0002	0.9713
race_Caucasian	0.0	0.8501	0.0	0.9093	-0.0592	-0.1827	0.8585	0.8236	0.0349	0.0956	0.0
race_African American	0.0	0.0567	0.0	0.0374	0.0194	0.0915	0.0516	0.0561	-0.0045	-0.0199	0.0008
race_Other/Unknown	0.0	0.0606	0.0	0.0392	0.0214	0.0982	0.07	0.0951	-0.0251	-0.0915	0.0
race_Asian	0.0	0.0326	0.0	0.0141	0.0185	0.1227	0.0199	0.0252	-0.0053	-0.0355	0.0
has_chickenpox_diagnosis	0.0	0.0018	0.0	0.0011	0.0006	0.0163	0.001	0.0008	0.0002	0.0067	0.264
has_diabetes_diagnosis	0.0	0.1938	0.0	0.1485	0.0453	0.1204	0.163	0.17	-0.007	-0.0187	0.0017
has_depression_diagnosis	0.0	0.1954	0.0	0.1143	0.0811	0.2257	0.1405	0.1498	-0.0093	-0.0263	0.0
has_stroke_diagnosis	0.0	0.0252	0.0	0.0148	0.0104	0.0746	0.0188	0.02	-0.0012	-0.009	0.1302
has_hypertension_diagnosis	0.0	0.5236	0.0	0.4206	0.1029	0.2073	0.4539	0.4542	-0.0003	-0.0006	0.9185

Covariate	Pre-Matching						Post-Matching				
	RZV (2+ doses)		ZVL				RZV (2+ doses)	ZVL			
	% missing	Mean	% missing	Mean	Difference in Mean	NMD	Mean	Mean	Difference in Mean	NMD	p
has_atherosclerosis_diagnosis	0.0	0.0415	0.0	0.0241	0.0174	0.098	0.0286	0.0326	-0.0039	-0.0228	0.0001
has_ischemic heart disease_diagnosis	0.0	0.1386	0.0	0.1087	0.0299	0.0909	0.1109	0.1131	-0.0021	-0.0068	0.2518
has_chronic obstructive pulmonary disease_diagnosis	0.0	0.0752	0.0	0.0559	0.0193	0.0778	0.0672	0.0696	-0.0024	-0.0096	0.1081
has_obesity_diagnosis	0.0	0.3109	0.0	0.1231	0.1878	0.468	0.2063	0.2324	-0.0261	-0.0632	0.0
has_herpes_simplex_diagnosis	0.0	0.0408	0.0	0.0169	0.0239	0.1432	0.0246	0.0268	-0.0021	-0.0136	0.0231
has_herpes_zoster_diagnosis	0.0	0.0502	0.0	0.0279	0.0223	0.1156	0.0367	0.039	-0.0023	-0.0119	0.0457
has_non-steroidal anti-inflammatory drugs_first	0.0	0.3744	0.0	0.2167	0.1577	0.3509	0.2679	0.2755	-0.0076	-0.0171	0.004
has_antivirals_first	0.0	0.2888	0.0	0.1789	0.1098	0.2617	0.2199	0.2427	-0.0227	-0.054	0.0
has_glucocorticoids_first	0.0	0.3491	0.0	0.174	0.1751	0.4066	0.2428	0.262	-0.0192	-0.0443	0.0

A normalized mean difference (NMD) greater than 0.1 indicates a potential imbalance between the cohorts. Covariates with NMD > 0.1 pre-matching are highlighted by red color of their respective pre-matching NMD. Covariates with NMD > 0.1 post-matching are highlighted by red background color of the corresponding row.

RZV (2+ doses), recipients of at least 2 doses of the recombinant zoster vaccine (*Shingrix*, GSK); ZVL, recipients of at least 1 dose of the live-attenuated zoster vaccine (*Zostavax*, Merck).

Supplementary Table 6 | Key pre- and post-matching characteristics of RZV (2+ doses) and RZV (1 dose) cohorts (dementia, all)

	Pre-Matching						Post-Matching				
	RZV (2+ doses)		RZV (1 dose)				RZV (2+ doses)	RZV (1 dose)			
**Covariate**	% missing	Mean	% missing	Mean	Difference in Mean	NMD	Mean	Mean	Difference in Mean	NMD	p
Age at exposure	0.0	65.2418	0.0	65.0683	0.1735	0.019	65.0837	65.0607	0.023	0.0025	0.3806
Body mass index	11.1	29.6391	10.1	29.9607	-0.3217	-0.0494	29.8867	29.8628	0.0189	0.0036	0.1834
gender_Female	0.0	0.5687	0.0	0.5646	0.0041	0.0083	0.5638	0.5636	0.0003	0.0005	0.808
gender_Male	0.0	0.431	0.0	0.4351	-0.0041	-0.0082	0.4359	0.4362	-0.0002	-0.0005	0.8286
race_Caucasian	0.0	0.8665	0.0	0.8198	0.0467	0.1286	0.8284	0.8314	-0.003	-0.008	0.0002
race_African American	0.0	0.0512	0.0	0.0831	-0.0319	-0.1277	0.0767	0.0736	0.0031	0.0116	0.0
race_Other/Unknown	0.0	0.0532	0.0	0.0693	-0.0161	-0.067	0.0672	0.0665	0.0008	0.003	0.1657
race_Asian	0.0	0.0291	0.0	0.0278	0.0013	0.0077	0.0277	0.0285	-0.0008	-0.005	0.0219
has_chickenpox_diagnosis	0.0	0.002	0.0	0.0016	0.0005	0.0107	0.0015	0.0016	-0.0001	-0.0015	0.4879
has_diabetes_diagnosis	0.0	0.2029	0.0	0.2276	-0.0247	-0.0601	0.2162	0.2157	0.0006	0.0013	0.5374
has_depression_diagnosis	0.0	0.1993	0.0	0.2305	-0.0312	-0.0761	0.2186	0.2174	0.0012	0.0029	0.1886
has_stroke_diagnosis	0.0	0.0276	0.0	0.0354	-0.0077	-0.0442	0.0327	0.0321	0.0006	0.0033	0.1294

Covariate	Pre-Matching						Post-Matching				
	RZV (2+ doses)		RZV (1 dose)		Difference in Mean	NMD	RZV (2+ doses)	RZV (1 dose)	Difference in Mean	NMD	p
	% missing	Mean	% missing	Mean			Mean	Mean			
has_hypertension_diagnosis	0.0	0.5548	0.0	0.5734	-0.0186	-0.0374	0.5589	0.5581	0.0008	0.0017	0.4492
has_atherosclerosis_diagnosis	0.0	0.0487	0.0	0.0565	-0.0078	-0.035	0.0527	0.052	0.0007	0.0029	0.1807
has_ischemic heart disease_diagnosis	0.0	0.1554	0.0	0.175	-0.0195	-0.0526	0.1664	0.1663	0.0001	0.0003	0.8991
has_chronic obstructive pulmonary disease_diagnosis	0.0	0.081	0.0	0.1047	-0.0236	-0.0815	0.0988	0.096	0.0028	0.0095	0.0
has_obesity_diagnosis	0.0	0.3138	0.0	0.3369	-0.0231	-0.0492	0.3238	0.3232	0.0006	0.0013	0.5406
has_herpes_simplex_diagnosis	0.0	0.0424	0.0	0.0405	0.0019	0.0094	0.0394	0.0395	-0.0001	-0.0007	0.7441
has_herpes_zoster_diagnosis	0.0	0.0523	0.0	0.0535	-0.0012	-0.0054	0.0519	0.0513	0.0006	0.0028	0.1998
has_non-steroidal anti-inflammatory drugs_first	0.0	0.3879	0.0	0.4178	-0.0299	-0.0611	0.3995	0.3986	0.0009	0.0019	0.3843
has_antivirals_first	0.0	0.3183	0.0	0.3209	-0.0026	-0.0056	0.3134	0.3139	-0.0005	-0.0011	0.6046

Covariate	Pre-Matching						Post-Matching				
	RZV (2+ doses)		RZV (1 dose)		Difference in Mean	NMD	RZV (2+ doses)	RZV (1 dose)	Difference in Mean	NMD	p
	% missing	Mean	% missing	Mean			Mean	Mean			
has_glucocorticoids_first	0.0	0.3648	0.0	0.3919	-0.0271	-0.0558	0.3755	0.3746	0.0009	0.0019	0.3885

A normalized mean difference (NMD) greater than 0.1 indicates a potential imbalance between the cohorts. Covariates with NMD > 0.1 pre-matching are highlighted by red color of their respective pre-matching NMD. Covariates with NMD > 0.1 post-matching are highlighted by red background color of the corresponding row.

RZV (2+ doses) and RZV (1 dose), recipients of at least 2 doses or 1 dose of the recombinant zoster vaccine (*Shingrix*, GSK).

Supplementary Table 7 | Key pre- and post-matching characteristics of ZVL and PPSV23 cohorts (dementia, women ≥50 years)

	Pre-Matching						Post-Matching				
	ZVL		PPSV23				ZVL	PPSV23			
**Covariate**	% missing	Mean	% missing	Mean	Difference in Mean	NMD	Mean	Mean	Difference in Mean	NMD	p
Age at exposure	0.0	65.0776	0.0	66.5881	-1.5105	-0.197	66.5	66.4207	0.0793	0.0109	0.0
Body mass index	37.1	28.9769	32.8	30.2571	-1.2801	-0.1767	29.63	29.5909	0.0486	0.0056	0.3494
gender_Female	0.0	1.0	0.0	1.0	0.0	nan	1.0	1.0	0.0	nan	1.0
gender_Male	0.0	0.0	0.0	0.0	0.0	nan	0.0	0.0	0.0	nan	1.0
race_Caucasian	0.0	0.9094	0.0	0.8265	0.0829	0.2468	0.8969	0.8999	-0.003	-0.0098	0.0
race_Other/Unknown	0.0	0.0387	0.0	0.0606	-0.0218	-0.1007	0.0441	0.0443	-0.0002	-0.0012	0.5865
race_African American	0.0	0.0372	0.0	0.0941	-0.0569	-0.2314	0.0446	0.0415	0.0031	0.0152	0.0
race_Asian	0.0	0.0146	0.0	0.0188	-0.0042	-0.0324	0.0145	0.0143	0.0001	0.0011	0.6127
has_chickenpox_diagnosis	0.0	0.001	0.0	0.0007	0.0003	0.011	0.0007	0.0006	0.0	0.0018	0.4079
has_diabetes_diagnosis	0.0	0.0912	0.0	0.1966	-0.1054	-0.3037	0.1131	0.1107	0.0024	0.0077	0.0006
has_depression_diagnosis	0.0	0.1239	0.0	0.1668	-0.0429	-0.122	0.109	0.1075	0.0015	0.005	0.0255
has_stroke_diagnosis	0.0	0.0089	0.0	0.025	-0.016	-0.1244	0.0104	0.01	0.0003	0.0031	0.1621
has_hypertension_diagnosis	0.0	0.342	0.0	0.4513	-0.1093	-0.2248	0.3298	0.3258	0.004	0.0086	0.0001

Covariate	Pre-Matching						Post-Matching				
	ZVL		PPSV23				ZVL	PPSV23			
	% missing	Mean	% missing	Mean	Difference in Mean	NMD	Mean	Mean	Difference in Mean	NMD	p
has_atherosclerosis_diagnosis	0.0	0.0135	0.0	0.0316	-0.0181	-0.1219	0.0151	0.0151	-0.0	-0.0003	0.8835
has_ischemic heart disease_diagnosis	0.0	0.0525	0.0	0.1102	-0.0577	-0.2124	0.0606	0.0591	0.0015	0.0064	0.004
has_chronic obstructive pulmonary disease_diagnosis	0.0	0.0331	0.0	0.1014	-0.0683	-0.2753	0.0431	0.0409	0.0022	0.011	0.0
has_obesity_diagnosis	0.0	0.1043	0.0	0.1652	-0.0609	-0.1791	0.094	0.0921	0.0019	0.0065	0.0037
has_herpes_simplex_diagnosis	0.0	0.0216	0.0	0.0169	0.0047	0.0339	0.0138	0.0142	-0.0004	-0.0036	0.1082
has_herpes_zoster_diagnosis	0.0	0.0288	0.0	0.0251	0.0037	0.023	0.0219	0.0226	-0.0007	-0.0045	0.0455
has_non-steroidal anti-inflammatory drugs_first	0.0	0.2091	0.0	0.2206	-0.0114	-0.0279	0.1674	0.1684	-0.0009	-0.0025	0.2601
has_antivirals_first	0.0	0.1652	0.0	0.1171	0.0481	0.1384	0.1014	0.1027	-0.0012	-0.0041	0.0657
has_glucocorticoids_first	0.0	0.1621	0.0	0.2049	-0.0428	-0.1107	0.1381	0.1386	-0.0005	-0.0014	0.5203

A normalized mean difference (NMD) greater than 0.1 indicates a potential imbalance between the cohorts. Covariates with NMD > 0.1 pre-matching are highlighted by red color of their respective pre-matching NMD. Covariates with NMD > 0.1 post-matching are highlighted by red background color of the corresponding row.

PPSV23, recipients of at least 1 dose of the 23-valent pneumococcal polysaccharide vaccine; ZVL, recipients of at least 1 dose of the live-attenuated zoster vaccine (*Zostavax*, Merck).

Supplementary Table 8 | Key pre- and post-matching characteristics of RZV (2+ doses) and PPSV23 cohorts (dementia, women 80–89 years)

	Pre-Matching						Post-Matching				
	RZV (2+ doses)		PPSV23				RZV (2+ doses)	PPSV23			
**Covariate**	% missing	Mean	% missing	Mean	Difference in Mean	NMD	Mean	Mean	Difference in Mean	NMD	p
Age at exposure	0.0	83.2678	0.0	81.9663	1.3015	0.6469	83.2843	83.2905	-0.0062	-0.0029	0.7791
Body mass index	16.6	27.0805	16.8	27.0561	0.0244	0.0045	27.174	27.0467	0.0862	0.0238	0.5333
gender_Female	0.0	1.0	0.0	1.0	0.0	nan	1.0	1.0	0.0	nan	1.0
gender_Male	0.0	0.0	0.0	0.0	0.0	nan	0.0	0.0	0.0	nan	1.0
race_Caucasian	0.0	0.9071	0.0	0.864	0.0431	0.1357	0.8866	0.8901	-0.0035	-0.0111	0.3837
race_African American	0.0	0.0337	0.0	0.0701	-0.0363	-0.1644	0.0424	0.0393	0.0031	0.0155	0.22
race_Other/Unknown	0.0	0.0396	0.0	0.0497	-0.0101	-0.049	0.0502	0.0511	-0.0009	-0.0041	0.7489
race_Asian	0.0	0.0195	0.0	0.0162	0.0034	0.0255	0.0208	0.0195	0.0013	0.0092	0.468
has_chickenpox_diagnosis	0.0	0.0012	0.0	0.0014	-0.0002	-0.0059	0.0013	0.0013	0.0	0.0	1.0
has_diabetes_diagnosis	0.0	0.2054	0.0	0.232	-0.0266	-0.0643	0.2015	0.209	-0.0075	-0.0186	0.1436
has_depression_diagnosis	0.0	0.1756	0.0	0.1726	0.0029	0.0077	0.1699	0.1667	0.0032	0.0084	0.5049
has_stroke_diagnosis	0.0	0.0602	0.0	0.069	-0.0088	-0.0359	0.0614	0.061	0.0004	0.0017	0.8945
has_hypertension_diagnosis	0.0	0.7196	0.0	0.7239	-0.0043	-0.0096	0.6912	0.6991	-0.0079	-0.0171	0.1787
has_atherosclerosis_diagnosis	0.0	0.1133	0.0	0.0944	0.0189	0.0618	0.1007	0.0969	0.0038	0.0128	0.3127

Covariate	Pre-Matching						Post-Matching				
	RZV (2+ doses)		PPSV23				RZV (2+ doses)	PPSV23			
	% missing	Mean	% missing	Mean	Difference in Mean	NMD	Mean	Mean	Difference in Mean	NMD	p
has_ischemic heart disease_diagnosis	0.0	0.2313	0.0	0.2517	-0.0205	-0.0479	0.2269	0.2323	-0.0053	-0.0127	0.3163
has_chronic obstructive pulmonary disease_diagnosis	0.0	0.1221	0.0	0.1504	-0.0284	-0.0828	0.1271	0.1253	0.0018	0.0054	0.6746
has_obesity_diagnosis	0.0	0.1845	0.0	0.135	0.0494	0.1353	0.1715	0.1679	0.0036	0.0095	0.4533
has_herpes_simplex_diagnosis	0.0	0.0212	0.0	0.0118	0.0093	0.0734	0.0166	0.0163	0.0003	0.0025	0.8407
has_herpes_zoster_diagnosis	0.0	0.0825	0.0	0.0488	0.0337	0.1365	0.069	0.0653	0.0036	0.0146	0.2544
has_non-steroidal anti-inflammatory drugs_first	0.0	0.3115	0.0	0.2343	0.0772	0.1739	0.2751	0.2706	0.0045	0.01	0.4321
has_antivirals_first	0.0	0.334	0.0	0.1722	0.1618	0.3788	0.2536	0.2346	0.0189	0.0441	0.0006
has_glucocorticoids_first	0.0	0.329	0.0	0.2496	0.0794	0.1758	0.2954	0.2831	0.0123	0.0271	0.0322

A normalized mean difference (NMD) greater than 0.1 indicates a potential imbalance between the cohorts. Covariates with NMD > 0.1 pre-matching are highlighted by red color of their respective pre-matching NMD. Covariates with NMD > 0.1 post-matching are highlighted by red background color of the corresponding row.

PPSV23, recipients of at least 1 dose of the 23-valent pneumococcal polysaccharide vaccine; RZV (2+ doses), recipients of at least 2 doses of the recombinant zoster vaccine (*Shingrix*, GSK).

Supplementary Table 9 | Key pre- and post-matching characteristics of ZVL and Not exposed cohorts (dementia, all)

Covariate	Pre-Matching						Post-Matching				
	ZVL		Not exposed				ZVL	Not exposed			
	% missing	Mean	% missing	Mean	Difference in Mean	NMD	Mean	Mean	Difference in Mean	NMD	p
Age at exposure	0.0	66.471	0.0	64.2057	2.2653	0.2477	66.5982	66.7806	-0.1823	-0.0244	0.0
Body mass index	5.0	30.1149	7.8	30.2046	-0.0898	-0.013	30.124	30.3289	-0.2084	-0.0305	0.0
gender_Female	0.0	0.5982	0.0	0.5881	0.0102	0.0207	0.5957	0.5975	-0.0019	-0.0038	0.0513
race_Caucasian	0.0	0.9127	0.0	0.8245	0.0882	0.2633	0.9089	0.8984	0.0105	0.0354	0.0
race_Other/Unknown	0.0	0.0246	0.0	0.0592	-0.0346	-0.1732	0.0255	0.0263	-0.0008	-0.005	0.0093
race_African American	0.0	0.0497	0.0	0.1003	-0.0506	-0.1929	0.0525	0.0627	-0.0101	-0.0435	0.0
race_Asian	0.0	0.0129	0.0	0.0159	-0.003	-0.0253	0.0131	0.0126	0.0005	0.0043	0.0256
has_chickenpox_diagnosis	0.0	0.002	0.0	0.0012	0.0007	0.0182	0.0019	0.0015	0.0004	0.0105	0.0
has_diabetes_diagnosis	0.0	0.268	0.0	0.2758	-0.0078	-0.0176	0.2722	0.295	-0.0228	-0.0506	0.0
has_depression_diagnosis	0.0	0.2224	0.0	0.243	-0.0205	-0.0486	0.2225	0.2408	-0.0183	-0.0434	0.0
has_stroke_diagnosis	0.0	0.0304	0.0	0.0521	-0.0217	-0.1094	0.0318	0.0366	-0.0048	-0.0262	0.0
has_hypertension_diagnosis	0.0	0.6794	0.0	0.629	0.0503	0.1059	0.6817	0.7012	-0.0195	-0.0423	0.0

Covariate	Pre-Matching						Post-Matching				
	ZVL		Not exposed		Difference in Mean	NMD	ZVL	Not exposed	Difference in Mean	NMD	p
	% missing	Mean	% missing	Mean			Mean	Mean			
has_atherosclerosis_diagnosis	0.0	0.0529	0.0	0.0719	-0.019	-0.0787	0.0546	0.0643	-0.0097	-0.0411	0.0
has_ischemic heart disease_diagnosis	0.0	0.2038	0.0	0.2395	-0.0357	-0.0861	0.2088	0.2322	-0.0235	-0.0567	0.0
has_chronic obstructive pulmonary disease_diagnosis	0.0	0.1099	0.0	0.1542	-0.0443	-0.1312	0.1153	0.1313	-0.016	-0.0486	0.0
has_obesity_diagnosis	0.0	0.2381	0.0	0.2948	-0.0568	-0.1287	0.2392	0.2446	-0.0054	-0.0125	0.0
has_herpes_simplex_diagnosis	0.0	0.0315	0.0	0.0275	0.004	0.0234	0.0302	0.0345	-0.0043	-0.0241	0.0
has_herpes_zoster_diagnosis	0.0	0.0538	0.0	0.036	0.0178	0.0862	0.0536	0.0578	-0.0041	-0.0181	0.0
has_non-steroidal anti-inflammatory drugs_first	0.0	0.424	0.0	0.3987	0.0253	0.0515	0.42	0.4259	-0.0059	-0.0119	0.0
has_antivirals_first	0.0	0.3328	0.0	0.1889	0.144	0.3324	0.3033	0.3065	-0.0032	-0.007	0.0002
has_glucocorticoids_first	0.0	0.361	0.0	0.3758	-0.0147	-0.0306	0.3606	0.3872	-0.0266	-0.0549	0.0

A normalized mean difference (NMD) greater than 0.1 indicates a potential imbalance between the cohorts. Covariates with NMD > 0.1 pre-matching are highlighted by red color of their respective pre-matching NMD. Covariates with NMD > 0.1 post-matching are highlighted by red background color of the corresponding row.

Covariate	Pre-Matching						Post-Matching				
	ZVL		Not exposed				ZVL	Not exposed			
	% missing	Mean	% missing	Mean	Difference in Mean	NMD	Mean	Mean	Difference in Mean	NMD	p

Not exposed, individuals not exposed to a herpes zoster vaccine; ZVL, recipients of at least 1 dose of the live-attenuated zoster vaccine (*Zostavax*, Merck).

Supplementary Table 10 | Key pre- and post-matching characteristics of RZV (2+ doses) and Not exposed cohorts (dementia, all)

	Pre-Matching						Post-Matching				
	RZV (2+ doses)		Not exposed				RZV (2+ doses)	Not exposed			
**Covariate**	% missing	Mean	% missing	Mean	Difference in Mean	NMD	Mean	Mean	Difference in Mean	NMD	p
Age at exposure	0.0	63.9303	0.0	64.2057	-0.2754	-0.0277	63.388	63.0799	0.3081	0.0334	0.0
BMI	2.0	30.0749	7.8	30.2046	-0.1298	-0.0187	30.1893	30.4608	-0.272	-0.0398	0.0
gender_Female	0.0	0.5971	0.0	0.5881	0.009	0.0183	0.5942	0.6073	-0.0131	-0.0268	0.0
gender_Male	0.0	0.4027	0.0	0.4116	-0.0089	-0.0181	0.4056	0.3924	0.0131	0.0268	0.0
race_Caucasian	0.0	0.8663	0.0	0.8245	0.0418	0.1158	0.861	0.8528	0.0082	0.0234	0.0
race_African American	0.0	0.0643	0.0	0.1003	-0.036	-0.1312	0.0686	0.0785	-0.0099	-0.0379	0.0
race_Other/Unknown	0.0	0.0417	0.0	0.0592	-0.0175	-0.0801	0.0432	0.0455	-0.0024	-0.0115	0.0
race_Asian	0.0	0.0277	0.0	0.0159	0.0117	0.0803	0.0273	0.0232	0.0041	0.0261	0.0
has_chickenpox_diagnosis	0.0	0.0024	0.0	0.0012	0.0012	0.0275	0.0025	0.0024	0.0001	0.0018	0.3653
has_diabetes_diagnosis	0.0	0.2491	0.0	0.2758	-0.0267	-0.0607	0.2517	0.2722	-0.0205	-0.0466	0.0
has_depression_diagnosis	0.0	0.2613	0.0	0.243	0.0184	0.0423	0.2646	0.2906	-0.026	-0.058	0.0
has_stroke_diagnosis	0.0	0.035	0.0	0.0521	-0.0172	-0.0842	0.0357	0.0427	-0.007	-0.0362	0.0

Covariate	Pre-Matching						Post-Matching				
	RZV (2+ doses)		Not exposed				RZV (2+ doses)	Not exposed			
	% missing	Mean	% missing	Mean	Difference in Mean	NMD	Mean	Mean	Difference in Mean	NMD	p
has_hypertension_diagnosis	0.0	0.6322	0.0	0.629	0.0031	0.0065	0.6295	0.6462	-0.0168	-0.0349	0.0
has_atherosclerosis_diagnosis	0.0	0.0599	0.0	0.0719	-0.012	-0.0485	0.0599	0.0684	-0.0085	-0.0346	0.0
has_ischemic heart disease_diagnosis	0.0	0.1876	0.0	0.2395	-0.052	-0.1271	0.1874	0.2059	-0.0184	-0.0464	0.0
has_chronic obstructive pulmonary disease_diagnosis	0.0	0.1036	0.0	0.1542	-0.0506	-0.1514	0.1071	0.1197	-0.0127	-0.0399	0.0
has_obesity_diagnosis	0.0	0.4015	0.0	0.2948	0.1066	0.2252	0.4068	0.4257	-0.0189	-0.0383	0.0
has_herpes_simplex_diagnosis	0.0	0.0537	0.0	0.0275	0.0262	0.1332	0.0523	0.0559	-0.0036	-0.0159	0.0
has_herpes_zoster_diagnosis	0.0	0.0679	0.0	0.036	0.0319	0.1441	0.0657	0.0697	-0.004	-0.0161	0.0
has_non-steroidal anti-inflammatory drugs_first	0.0	0.5064	0.0	0.3987	0.1077	0.2177	0.5069	0.5408	-0.0339	-0.0679	0.0
has_antivirals_first	0.0	0.3778	0.0	0.1889	0.189	0.4288	0.3569	0.3692	-0.0123	-0.0256	0.0
has_glucocorticoids_first	0.0	0.4781	0.0	0.3758	0.1023	0.2079	0.4815	0.5173	-0.0358	-0.0716	0.0

A normalized mean difference (NMD) greater than 0.1 indicates a potential imbalance between the cohorts. Covariates with NMD > 0.1 pre-matching are highlighted by red color of their respective pre-matching NMD. Covariates with NMD > 0.1 post-matching are highlighted by red background color of the corresponding row.

Covariate	Pre-Matching						Post-Matching				
	RZV (2+ doses)		Not exposed		Difference in Mean	NMD	RZV (2+ doses)	Not exposed	Difference in Mean	NMD	p
	% missing	Mean	% missing	Mean			Mean	Mean			

Not exposed, individuals not exposed to a herpes zoster vaccine; RZV (2+ doses), recipients of at least 2 doses of the recombinant zoster vaccine (*Shingrix*, GSK).

Supplementary Table 11 | Key pre- and post-matching characteristics of ZVL and PPSV23 cohorts (dementia defined by code AND prescribed medication, all)

	Pre-Matching						Post-Matching				
	ZVL		PPSV23				ZVL	PPSV23			
**Covariate**	% missing	Mean	% missing	Mean	Difference in Mean	NMD	Mean	Mean	Difference in Mean	NMD	p
Age at exposure	0.0	65.1409	0.0	66.3055	-1.1646	-0.1533	66.4826	66.3639	0.1187	0.0166	0.0
Body mass index	36.7	29.2746	32.3	30.1059	-0.8313	-0.1249	29.7648	29.7504	0.0328	0.0022	0.1026
gender_Female	0.0	0.5825	0.0	0.5447	0.0378	0.0763	0.5703	0.5717	-0.0014	-0.0029	0.0881
race_Caucasian	0.0	0.9123	0.0	0.8318	0.0805	0.2428	0.9012	0.9039	-0.0027	-0.0091	0.0
race_Other/Unknown	0.0	0.0409	0.0	0.0623	-0.0215	-0.0973	0.0461	0.0454	0.0006	0.003	0.0702
race_African American	0.0	0.033	0.0	0.0875	-0.0545	-0.2304	0.039	0.037	0.002	0.0104	0.0
race_Asian	0.0	0.0138	0.0	0.0184	-0.0046	-0.0362	0.0138	0.0137	0.0001	0.0006	0.7281
has_chickenpox_diagnosis	0.0	0.0009	0.0	0.0006	0.0003	0.011	0.0006	0.0006	0.0	0.0008	0.6335
has_diabetes_diagnosis	0.0	0.1041	0.0	0.2149	-0.1108	-0.306	0.1271	0.1243	0.0028	0.0086	0.0
has_depression_diagnosis	0.0	0.0986	0.0	0.1343	-0.0357	-0.1114	0.0873	0.0867	0.0006	0.0022	0.1947
has_stroke_diagnosis	0.0	0.0104	0.0	0.0278	-0.0174	-0.1278	0.0118	0.0114	0.0004	0.0034	0.0423
has_hypertension_diagnosis	0.0	0.3639	0.0	0.4654	-0.1014	-0.2069	0.3463	0.3431	0.0031	0.0066	0.0001

	Pre-Matching						Post-Matching				
	ZVL		PPSV23				ZVL	PPSV23			
Covariate	% missing	Mean	% missing	Mean	Difference in Mean	NMD	Mean	Mean	Difference in Mean	NMD	p
has_atherosclerosis_diagnosis	0.0	0.0163	0.0	0.0358	-0.0195	-0.1225	0.0182	0.0179	0.0003	0.0024	0.156
has_ischemic heart disease_diagnosis	0.0	0.0814	0.0	0.1504	-0.069	-0.2167	0.0919	0.0898	0.0021	0.0074	0.0
has_chronic obstructive pulmonary disease_diagnosis	0.0	0.035	0.0	0.1053	-0.0704	-0.2782	0.045	0.0436	0.0014	0.0069	0.0
has_obesity_diagnosis	0.0	0.1035	0.0	0.1591	-0.0556	-0.1653	0.0927	0.0923	0.0005	0.0016	0.3525
has_herpes_simplex_diagnosis	0.0	0.0173	0.0	0.013	0.0043	0.0353	0.0112	0.0113	-0.0001	-0.0007	0.6888
has_herpes_zoster_diagnosis	0.0	0.0253	0.0	0.0214	0.0039	0.0257	0.0196	0.0202	-0.0005	-0.0039	0.022
has_non-steroidal anti-inflammatory drugs_first	0.0	0.2001	0.0	0.2051	-0.005	-0.0124	0.1602	0.1603	-0.0001	-0.0003	0.846
has_antivirals_first	0.0	0.1549	0.0	0.1071	0.0478	0.1419	0.0956	0.0972	-0.0016	-0.0054	0.0012
has_glucocorticoids_first	0.0	0.1537	0.0	0.188	-0.0343	-0.0913	0.1304	0.1309	-0.0005	-0.0014	0.4197

A normalized mean difference (NMD) greater than 0.1 indicates a potential imbalance between the cohorts. Covariates with NMD > 0.1 pre-matching are highlighted by red color of their respective pre-matching NMD. Covariates with NMD > 0.1 post-matching are highlighted by red background color of the corresponding row.

PPSV23, recipients of at least 1 dose of the 23-valent pneumococcal polysaccharide vaccine; ZVL, recipients of at least 1 dose of the live-attenuated zoster vaccine (*Zostavax*, Merck).

Supplementary Table 12 | Key pre- and post-matching characteristics of RZV (2+ doses) and PPSV23 cohorts (dementia defined by code AND prescribed medication, all)

Covariate	Pre-Matching						Post-Matching				
	RZV (2+ doses)		PPSV23				RZV (2+ doses)	PPSV23			
	% missing	Mean	% missing	Mean	Difference in Mean	NMD	Mean	Mean	Difference in Mean	NMD	p
Age at exposure	0.0	62.0827	0.0	66.6088	-4.5261	-0.5533	65.4769	65.2258	0.2511	0.0284	0.0
Body mass index	13.0	29.2545	31.8	30.0202	-0.7657	-0.1155	30.0831	30.0377	0.041	0.0068	0.1586
gender_Female	0.0	0.5761	0.0	0.5484	0.0277	0.0559	0.5555	0.5552	0.0003	0.0006	0.8365
race_Caucasian	0.0	0.8597	0.0	0.843	0.0167	0.047	0.8405	0.843	-0.0025	-0.0068	0.0201
race_African American	0.0	0.0466	0.0	0.0811	-0.0344	-0.1412	0.0616	0.0602	0.0014	0.0059	0.0449
race_Other/Unknown	0.0	0.0604	0.0	0.0586	0.0018	0.0075	0.0701	0.0683	0.0018	0.0072	0.0137
race_Asian	0.0	0.0333	0.0	0.0174	0.0159	0.1015	0.0278	0.0285	-0.0008	-0.0045	0.1205
has_chickenpox_diagnosis	0.0	0.0018	0.0	0.0007	0.0011	0.0304	0.0013	0.0013	0.0	0.0004	0.9018
has_diabetes_diagnosis	0.0	0.1168	0.0	0.2089	-0.0921	-0.2513	0.2146	0.2061	0.0085	0.0208	0.0
has_depression_diagnosis	0.0	0.1701	0.0	0.1336	0.0365	0.1019	0.178	0.1768	0.0012	0.0031	0.2964
has_stroke_diagnosis	0.0	0.0172	0.0	0.0268	-0.0096	-0.0654	0.0263	0.0252	0.0011	0.0071	0.0146

Covariate	Pre-Matching						Post-Matching				
	RZV (2+ doses)		PPSV23				RZV (2+ doses)	PPSV23			
	% missing	Mean	% missing	Mean	Difference in Mean	NMD	Mean	Mean	Difference in Mean	NMD	p
has_hypertension_diagnosis	0.0	0.4499	0.0	0.4686	-0.0187	-0.0376	0.5278	0.5211	0.0067	0.0134	0.0
has_atherosclerosis_diagnosis	0.0	0.026	0.0	0.0356	-0.0096	-0.0553	0.0395	0.0379	0.0016	0.0082	0.0051
has_ischemic heart disease_diagnosis	0.0	0.0984	0.0	0.1488	-0.0504	-0.1535	0.1476	0.1444	0.0032	0.0091	0.0019
has_chronic obstructive pulmonary disease_diagnosis	0.0	0.04	0.0	0.101	-0.061	-0.2399	0.077	0.0743	0.0027	0.0102	0.0004
has_obesity_diagnosis	0.0	0.2684	0.0	0.1562	0.1122	0.2771	0.2809	0.2805	0.0004	0.0009	0.7491
has_herpes_simplex_diagnosis	0.0	0.0427	0.0	0.0136	0.0291	0.1765	0.0289	0.0304	-0.0016	-0.0092	0.0018
has_herpes_zoster_diagnosis	0.0	0.0451	0.0	0.0222	0.023	0.1276	0.039	0.0389	0.0001	0.0007	0.82
has_non-steroidal anti-inflammatory drugs_first	0.0	0.353	0.0	0.2096	0.1434	0.323	0.3276	0.3277	-0.0001	-0.0002	0.9576
has_antivirals_first	0.0	0.2705	0.0	0.1166	0.1539	0.397	0.2184	0.2206	-0.0022	-0.0052	0.0746
has_glucocorticoids_first	0.0	0.3213	0.0	0.1904	0.1309	0.3033	0.3106	0.3126	-0.002	-0.0044	0.1372

	Pre-Matching						Post-Matching				
	RZV (2+ doses)		PPSV23				RZV (2+ doses)	PPSV23			
**Covariate**	% missing	Mean	% missing	Mean	Difference in Mean	NMD	Mean	Mean	Difference in Mean	NMD	p

A normalized mean difference (NMD) greater than 0.1 indicates a potential imbalance between the cohorts. Covariates with NMD > 0.1 pre-matching are highlighted by red color of their respective pre-matching NMD. Covariates with NMD > 0.1 post-matching are highlighted by red background color of the corresponding row.

PPSV23, recipients of at least 1 dose of the 23-valent pneumococcal polysaccharide vaccine; RZV (2+ doses), recipients of at least 2 doses of the recombinant zoster vaccine (*Shingrix*, GSK).

Supplementary Table 13 | Key pre- and post-matching characteristics of ZVL and PPSV23 cohorts (dementia defined by code OR prescribed medication, all)

	Pre-Matching						Post-Matching				
	ZVL		PPSV23				ZVL	PPSV23			
**Covariate**	% missing	Mean	% missing	Mean	Difference in Mean	NMD	Mean	Mean	Difference in Mean	NMD	p
Age at exposure	0.0	65.1069	0.0	66.1979	-1.091	-0.1443	66.4206	66.3199	0.1007	0.0142	0.0
Body mass index	36.8	29.2826	32.5	30.1549	-0.8723	-0.131	29.7946	29.7769	0.0022	0.0027	0.7908
gender_Female	0.0	0.5825	0.0	0.544	0.0384	0.0776	0.5689	0.572	-0.0031	-0.0063	0.0002
race_Caucasian	0.0	0.9124	0.0	0.8321	0.0803	0.2423	0.9009	0.9046	-0.0037	-0.0127	0.0
race_Other/Unknown	0.0	0.0409	0.0	0.0623	-0.0215	-0.0972	0.0461	0.045	0.0011	0.0055	0.0012
race_African American	0.0	0.0329	0.0	0.0872	-0.0542	-0.2297	0.0391	0.0369	0.0021	0.0111	0.0
race_Asian	0.0	0.0138	0.0	0.0184	-0.0046	-0.0365	0.014	0.0135	0.0005	0.0041	0.0146
has_chickenpox_diagnosis	0.0	0.0009	0.0	0.0006	0.0003	0.0111	0.0006	0.0006	-0.0	-0.0014	0.4104
has_diabetes_diagnosis	0.0	0.1035	0.0	0.2138	-0.1104	-0.3056	0.1266	0.1232	0.0034	0.0104	0.0
has_depression_diagnosis	0.0	0.0977	0.0	0.132	-0.0343	-0.1078	0.0859	0.0855	0.0003	0.0011	0.4957
has_stroke_diagnosis	0.0	0.0101	0.0	0.0268	-0.0168	-0.1249	0.0115	0.011	0.0005	0.0048	0.0041
has_hypertension_diagnosis	0.0	0.3626	0.0	0.4622	-0.0996	-0.2034	0.3451	0.341	0.004	0.0085	0.0
has_atherosclerosis_diagnosis	0.0	0.0161	0.0	0.0353	-0.0191	-0.1212	0.0179	0.0174	0.0005	0.0038	0.0254

	Pre-Matching						Post-Matching				
	ZVL		PPSV23				ZVL	PPSV23			
**Covariate**	% missing	Mean	% missing	Mean	Difference in Mean	NMD	Mean	Mean	Difference in Mean	NMD	p
has_ischemic heart disease_diagnosis	0.0	0.0807	0.0	0.1486	-0.0679	-0.2142	0.0911	0.089	0.0021	0.0073	0.0
has_chronic obstructive pulmonary disease_diagnosis	0.0	0.0346	0.0	0.1043	-0.0697	-0.2769	0.0447	0.043	0.0018	0.0086	0.0
has_obesity_diagnosis	0.0	0.1033	0.0	0.1594	-0.0561	-0.1665	0.0928	0.0916	0.0013	0.0043	0.01
has_herpes_simplex_diagnosis	0.0	0.0173	0.0	0.013	0.0043	0.0351	0.0112	0.0115	-0.0003	-0.0032	0.0618
has_herpes_zoster_diagnosis	0.0	0.0252	0.0	0.0213	0.0039	0.026	0.0195	0.02	-0.0004	-0.0031	0.0673
has_non-steroidal anti-inflammatory drugs_first	0.0	0.1996	0.0	0.2048	-0.0052	-0.013	0.1594	0.158	0.0015	0.004	0.0181
has_antivirals_first	0.0	0.1544	0.0	0.1068	0.0475	0.1415	0.0948	0.0969	-0.0021	-0.0072	0.0
has_glucocorticoids_first	0.0	0.1532	0.0	0.1878	-0.0346	-0.092	0.1301	0.1292	0.0009	0.0027	0.1121

A normalized mean difference (NMD) greater than 0.1 indicates a potential imbalance between the cohorts. Covariates with NMD > 0.1 pre-matching are highlighted by red color of their respective pre-matching NMD. Covariates with NMD > 0.1 post-matching are highlighted by red background color of the corresponding row.

PPSV23, recipients of at least 1 dose of the 23-valent pneumococcal polysaccharide vaccine; ZVL, recipients of at least 1 dose of the live-attenuated zoster vaccine (*Zostavax*, Merck).

Supplementary Table 14 | Key pre- and post-matching characteristics of RZV (2+ doses) and PPSV23 cohorts (dementia defined by code OR prescribed medication, all)

	Pre-Matching						Post-Matching				
	RZV (2+ doses)		PPSV23				RZV (2+ doses)	PPSV23			
**Covariate**	% missing	Mean	% missing	Mean	Difference in Mean	NMD	Mean	Mean	Difference in Mean	NMD	p
Age at exposure	0.0	62.0385	0.0	66.5086	-4.4701	-0.5492	65.4332	65.1729	0.2603	0.0296	0.0
Body mass index	13.1	29.2594	32.0	30.0652	-0.8058	-0.1215	30.1072	30.0628	0.037	0.0067	0.1085
gender_Female	0.0	0.5761	0.0	0.5479	0.0283	0.057	0.5543	0.5544	-0.0001	-0.0001	0.9696
race_Caucasian	0.0	0.8596	0.0	0.8433	0.0164	0.046	0.8408	0.842	-0.0013	-0.0035	0.2279
race_African American	0.0	0.0466	0.0	0.0807	-0.0341	-0.14	0.0623	0.0607	0.0016	0.0067	0.0209
race_Other/Unknown	0.0	0.0604	0.0	0.0586	0.0018	0.0078	0.0692	0.0687	0.0005	0.0019	0.5163
race_Asian	0.0	0.0334	0.0	0.0174	0.0159	0.1013	0.0277	0.0285	-0.0008	-0.0049	0.0935
has_chickenpox_diagnosis	0.0	0.0018	0.0	0.0007	0.0011	0.0304	0.0012	0.0013	-0.0001	-0.0028	0.3416
has_diabetes_diagnosis	0.0	0.1163	0.0	0.2078	-0.0916	-0.2504	0.2145	0.2077	0.0068	0.0166	0.0
has_depression_diagnosis	0.0	0.1691	0.0	0.1313	0.0377	0.1058	0.177	0.1763	0.0007	0.0018	0.5248
has_stroke_diagnosis	0.0	0.0169	0.0	0.0259	-0.009	-0.0625	0.0255	0.0251	0.0005	0.003	0.298
has_hypertension_diagnosis	0.0	0.4488	0.0	0.4656	-0.0167	-0.0336	0.5282	0.5226	0.0056	0.0113	0.0001
has_atherosclerosis_diagnosis	0.0	0.0257	0.0	0.0351	-0.0093	-0.0545	0.0394	0.0376	0.0018	0.0094	0.0012

Covariate	Pre-Matching						Post-Matching				
	RZV (2+ doses)		PPSV23				RZV (2+ doses)	PPSV23			
	% missing	Mean	% missing	Mean	Difference in Mean	NMD	Mean	Mean	Difference in Mean	NMD	p
has_ischemic heart disease_diagnosis	0.0	0.0976	0.0	0.147	-0.0494	-0.1511	0.147	0.1444	0.0025	0.0072	0.0136
has_chronic obstructive pulmonary disease_diagnosis	0.0	0.0396	0.0	0.1	-0.0604	-0.2386	0.0767	0.0738	0.003	0.0113	0.0001
has_obesity_diagnosis	0.0	0.2683	0.0	0.1564	0.1119	0.2763	0.2844	0.2811	0.0033	0.0073	0.0122
has_herpes_simplex_diagnosis	0.0	0.0428	0.0	0.0137	0.0291	0.1765	0.0297	0.0307	-0.0009	-0.0054	0.0629
has_herpes_zoster_diagnosis	0.0	0.045	0.0	0.022	0.023	0.128	0.0391	0.0388	0.0003	0.0014	0.6255
has_non-steroidal anti-inflammatory drugs_first	0.0	0.3526	0.0	0.2093	0.1433	0.3229	0.329	0.3287	0.0003	0.0007	0.811
has_antivirals_first	0.0	0.2699	0.0	0.1163	0.1536	0.3968	0.221	0.2208	0.0002	0.0004	0.8826
has_glucocorticoids_first	0.0	0.3208	0.0	0.1901	0.1307	0.3032	0.3121	0.3148	-0.0027	-0.0058	0.0458

A normalized mean difference (NMD) greater than 0.1 indicates a potential imbalance between the cohorts. Covariates with NMD > 0.1 pre-matching are highlighted by red color of their respective pre-matching NMD. Covariates with NMD > 0.1 post-matching are highlighted by red background color of the corresponding row.

PPSV23, recipients of at least 1 dose of the 23-valent pneumococcal polysaccharide vaccine; RZV (2+ doses), recipients of at least 2 doses of the recombinant zoster vaccine (*Shingrix*, GSK).

Supplementary Table 15 | Key pre- and post-matching characteristics of ZVL and PPSV23 cohorts (Alzheimer's disease, all)

	Pre-Matching						Post-Matching				
	ZVL		PPSV23				ZVL	PPSV23			
**Covariate**	% missing	Mean	% missing	Mean	Difference in Mean	NMD	Mean	Mean	Difference in Mean	NMD	p
Age at exposure	0.0	65.1595	0.0	66.3266	-1.1671	-0.1535	66.4884	66.3472	0.1412	0.0197	0.0
Body mass index	36.7	29.2684	32.2	30.0945	-0.8261	-0.1242	29.7623	29.7447	0.0067	0.0027	0.8751
gender_Female	0.0	0.5824	0.0	0.5448	0.0376	0.0759	0.5711	0.5717	-0.0006	-0.0012	0.4631
race_Caucasian	0.0	0.9123	0.0	0.8319	0.0805	0.2427	0.9012	0.903	-0.0018	-0.006	0.0004
race_Other/Unknown	0.0	0.0409	0.0	0.0623	-0.0215	-0.0971	0.046	0.0462	-0.0002	-0.001	0.5681
race_African American	0.0	0.033	0.0	0.0875	-0.0545	-0.2304	0.039	0.0372	0.0018	0.0095	0.0
race_Asian	0.0	0.0138	0.0	0.0183	-0.0045	-0.036	0.0138	0.0136	0.0002	0.0013	0.4428
has_chickenpox_diagnosis	0.0	0.0009	0.0	0.0006	0.0003	0.011	0.0006	0.0006	0.0	0.0007	0.6763
has_diabetes_diagnosis	0.0	0.1044	0.0	0.215	-0.1106	-0.3054	0.128	0.1238	0.0042	0.0127	0.0
has_depression_diagnosis	0.0	0.099	0.0	0.1348	-0.0358	-0.1116	0.0875	0.0862	0.0012	0.0043	0.0103
has_stroke_diagnosis	0.0	0.0105	0.0	0.028	-0.0175	-0.1274	0.0118	0.0116	0.0002	0.0023	0.1802
has_hypertension_diagnosis	0.0	0.3646	0.0	0.466	-0.1014	-0.2069	0.348	0.3418	0.0062	0.013	0.0
has_atherosclerosis_diagnosis	0.0	0.0164	0.0	0.0359	-0.0195	-0.1223	0.0184	0.0172	0.0011	0.0085	0.0
has_ischemic heart disease_diagnosis	0.0	0.0818	0.0	0.1507	-0.0689	-0.2163	0.0919	0.0893	0.0026	0.0091	0.0

Covariate	Pre-Matching						Post-Matching				
	ZVL		PPSV23				ZVL	PPSV23			
	% missing	Mean	% missing	Mean	Difference in Mean	NMD	Mean	Mean	Difference in Mean	NMD	p
has_chronic obstructive pulmonary disease_diagnosis	0.0	0.0351	0.0	0.1054	-0.0703	-0.2777	0.0454	0.0433	0.0021	0.01	0.0
has_obesity_diagnosis	0.0	0.1034	0.0	0.1589	-0.0555	-0.1649	0.0931	0.0917	0.0014	0.0048	0.004
has_herpes_simplex_diagnosis	0.0	0.0172	0.0	0.0129	0.0043	0.0353	0.0111	0.0116	-0.0005	-0.0046	0.0064
has_herpes_zoster_diagnosis	0.0	0.0253	0.0	0.0215	0.0039	0.0256	0.0198	0.0199	-0.0001	-0.0008	0.6157
has_non-steroidal anti-inflammatory drugs_first	0.0	0.2003	0.0	0.2054	-0.0051	-0.0126	0.1605	0.1591	0.0014	0.0038	0.0242
has_antivirals_first	0.0	0.1552	0.0	0.1073	0.0478	0.142	0.0953	0.0971	-0.0018	-0.0061	0.0003
has_glucocorticoids_first	0.0	0.1539	0.0	0.1882	-0.0343	-0.0913	0.131	0.1299	0.001	0.003	0.0715

A normalized mean difference (NMD) greater than 0.1 indicates a potential imbalance between the cohorts. Covariates with NMD > 0.1 pre-matching are highlighted by red color of their respective pre-matching NMD. Covariates with NMD > 0.1 post-matching are highlighted by red background color of the corresponding row.

PPSV23, recipients of at least 1 dose of the 23-valent pneumococcal polysaccharide vaccine; ZVL, recipients of at least 1 dose of the live-attenuated zoster vaccine (*Zostavax*, Merck).

Supplementary Table 16 | Key pre- and post-matching characteristics of RZV (2+ doses) and PPSV23 cohorts (Alzheimer's disease, all)

	Pre-Matching						Post-Matching				
	RZV (2+ doses)		PPSV23				RZV (2+ doses)	PPSV23			
Covariate	% missing	Mean	% missing	Mean	Difference in Mean	NMD	Mean	Mean	Difference in Mean	NMD	p
Age at exposure	0.0	62.0844	0.0	66.63	-4.5456	-0.5555	65.5431	65.1788	0.3643	0.0412	0.0
Body mass index	13.0	29.2549	31.8	30.009	-0.7541	-0.1138	30.0944	30.0682	0.0472	0.0039	0.0517
gender_Female	0.0	0.5761	0.0	0.5485	0.0276	0.0556	0.5536	0.5553	-0.0017	-0.0034	0.2401
race_Caucasian	0.0	0.8597	0.0	0.843	0.0167	0.047	0.8415	0.8421	-0.0006	-0.0016	0.5831
race_African American	0.0	0.0466	0.0	0.0811	-0.0345	-0.1413	0.0623	0.0615	0.0008	0.0035	0.2232
race_Other/Unknown	0.0	0.0603	0.0	0.0586	0.0018	0.0076	0.0694	0.068	0.0013	0.0053	0.0675
race_Asian	0.0	0.0333	0.0	0.0173	0.016	0.1017	0.0268	0.0284	-0.0016	-0.0098	0.0007
has_chickenpox_diagnosis	0.0	0.0018	0.0	0.0007	0.0011	0.0304	0.0012	0.0013	-0.0001	-0.002	0.488
has_diabetes_diagnosis	0.0	0.1169	0.0	0.2091	-0.0922	-0.2515	0.216	0.208	0.008	0.0196	0.0
has_depression_diagnosis	0.0	0.1701	0.0	0.1341	0.036	0.1004	0.1785	0.179	-0.0004	-0.0012	0.6908
has_stroke_diagnosis	0.0	0.0173	0.0	0.027	-0.0097	-0.0658	0.0267	0.0259	0.0008	0.005	0.0828
has_hypertension_diagnosis	0.0	0.45	0.0	0.4693	-0.0193	-0.0388	0.5306	0.5242	0.0064	0.0128	0.0
has_atherosclerosis_diagnosis	0.0	0.0261	0.0	0.0357	-0.0096	-0.0558	0.0397	0.0393	0.0004	0.002	0.4975
has_ischemic heart disease_diagnosis	0.0	0.0984	0.0	0.1492	-0.0507	-0.1545	0.1483	0.1459	0.0023	0.0065	0.0234

Covariate	Pre-Matching						Post-Matching				
	RZV (2+ doses)		PPSV23				RZV (2+ doses)	PPSV23			
	% missing	Mean	% missing	Mean	Difference in Mean	NMD	Mean	Mean	Difference in Mean	NMD	p
has_chronic obstructive pulmonary disease_diagnosis	0.0	0.0401	0.0	0.1012	-0.0611	-0.2401	0.0776	0.0743	0.0033	0.0123	0.0
has_obesity_diagnosis	0.0	0.2684	0.0	0.156	0.1124	0.2776	0.2833	0.2826	0.0007	0.0016	0.5714
has_herpes_simplex_diagnosis	0.0	0.0427	0.0	0.0136	0.0291	0.1766	0.0294	0.0305	-0.0011	-0.0065	0.0253
has_herpes_zoster_diagnosis	0.0	0.0452	0.0	0.0222	0.023	0.1276	0.0388	0.0393	-0.0005	-0.0026	0.3737
has_non-steroidal anti-inflammatory drugs_first	0.0	0.3532	0.0	0.21	0.1432	0.3225	0.3306	0.3314	-0.0008	-0.0017	0.5661
has_antivirals_first	0.0	0.2706	0.0	0.1168	0.1537	0.3965	0.2214	0.2238	-0.0024	-0.0057	0.0504
has_glucocorticoids_first	0.0	0.3214	0.0	0.1906	0.1308	0.3031	0.3142	0.3146	-0.0004	-0.0009	0.7496

A normalized mean difference (NMD) greater than 0.1 indicates a potential imbalance between the cohorts. Covariates with NMD > 0.1 pre-matching are highlighted by red color of their respective pre-matching NMD. Covariates with NMD > 0.1 post-matching are highlighted by red background color of the corresponding row.

PPSV23, recipients of at least 1 dose of the 23-valent pneumococcal polysaccharide vaccine; RZV (2+ doses), recipients of at least 2 doses of the recombinant zoster vaccine (*Shingrix*, GSK).

Supplementary Table 17 | Key pre- and post-matching characteristics of ZVL and PPSV23 cohorts (Vascular dementia, all)

	Pre-Matching						Post-Matching				
	ZVL		PPSV23				ZVL	PPSV23			
**Covariate**	% missing	Mean	% missing	Mean	Difference in Mean	NMD	Mean	Mean	Difference in Mean	NMD	p
Age at exposure	0.0	65.1666	0.0	66.3569	-1.1903	-0.1563	66.484	66.374	0.11	0.0154	0.0
Body mass index	36.7	29.2657	32.2	30.0814	-0.8157	-0.1227	29.7608	29.7513	0.0167	0.0015	0.9788
gender_Female	0.0	0.5824	0.0	0.5451	0.0373	0.0753	0.5721	0.5714	0.0007	0.0014	0.4125
gender_Male	0.0	0.4173	0.0	0.4545	-0.0372	-0.075	0.4276	0.4283	-0.0007	-0.0014	0.4057
race_Caucasian	0.0	0.9123	0.0	0.8319	0.0804	0.2426	0.9009	0.902	-0.0011	-0.0038	0.0231
race_Other/Unknown	0.0	0.0409	0.0	0.0623	-0.0215	-0.0971	0.0463	0.0462	0.0001	0.0003	0.847
race_African American	0.0	0.033	0.0	0.0875	-0.0545	-0.2303	0.0391	0.0378	0.0013	0.0065	0.0001
race_Asian	0.0	0.0138	0.0	0.0183	-0.0045	-0.036	0.0138	0.014	-0.0002	-0.0017	0.3237
has_chickenpox_diagnosis	0.0	0.0009	0.0	0.0006	0.0003	0.0111	0.0006	0.0006	0.0	0.0002	0.8916
has_diabetes_diagnosis	0.0	0.1045	0.0	0.215	-0.1106	-0.3052	0.127	0.1248	0.0022	0.0066	0.0001
has_depression_diagnosis	0.0	0.0992	0.0	0.1352	-0.036	-0.1122	0.0874	0.0867	0.0007	0.0026	0.1195
has_stroke_diagnosis	0.0	0.0105	0.0	0.0279	-0.0174	-0.1269	0.0118	0.0114	0.0004	0.004	0.0172
has_hypertension_diagnosis	0.0	0.3648	0.0	0.4666	-0.1018	-0.2077	0.3464	0.3446	0.0018	0.0037	0.0272
has_atherosclerosis_diagnosis	0.0	0.0165	0.0	0.036	-0.0195	-0.1225	0.0181	0.0177	0.0004	0.0028	0.101

	Pre-Matching						Post-Matching				
	ZVL		PPSV23				ZVL	PPSV23			
Covariate	% missing	Mean	% missing	Mean	Difference in Mean	NMD	Mean	Mean	Difference in Mean	NMD	p
has_ischemic heart disease_diagnosis	0.0	0.0819	0.0	0.1509	-0.069	-0.2166	0.0916	0.0911	0.0005	0.0017	0.3064
has_chronic obstructive pulmonary disease_diagnosis	0.0	0.0352	0.0	0.1055	-0.0703	-0.2776	0.0454	0.0439	0.0014	0.007	0.0
has_obesity_diagnosis	0.0	0.1034	0.0	0.1588	-0.0554	-0.1646	0.0924	0.0923	0.0001	0.0004	0.7908
has_herpes_simplex_diagnosis	0.0	0.0172	0.0	0.0129	0.0043	0.0353	0.0112	0.0117	-0.0005	-0.0043	0.0101
has_herpes_zoster_diagnosis	0.0	0.0254	0.0	0.0215	0.0039	0.0255	0.0197	0.0201	-0.0003	-0.0022	0.1825
has_non-steroidal anti-inflammatory drugs_first	0.0	0.2004	0.0	0.2055	-0.0051	-0.0126	0.1594	0.1602	-0.0008	-0.0021	0.2004
has_antivirals_first	0.0	0.1552	0.0	0.1075	0.0478	0.1418	0.095	0.0978	-0.0028	-0.0094	0.0
has_glucocorticoids_first	0.0	0.1539	0.0	0.1883	-0.0343	-0.0913	0.1304	0.1298	0.0006	0.0018	0.2788

A normalized mean difference (NMD) greater than 0.1 indicates a potential imbalance between the cohorts. Covariates with NMD > 0.1 pre-matching are highlighted by red color of their respective pre-matching NMD. Covariates with NMD > 0.1 post-matching are highlighted by red background color of the corresponding row.

PPSV23, recipients of at least 1 dose of the 23-valent pneumococcal polysaccharide vaccine; ZVL, recipients of at least 1 dose of the live-attenuated zoster vaccine (*Zostavax*, Merck).

Supplementary Table 18 | Key pre- and post-matching characteristics of RZV (2+ doses) and PPSV23 cohorts (Vascular dementia, all)

	Pre-Matching						Post-Matching				
	RZV (2+doses)		PPSV23				RZV (2+ doses)	PPSV23			
**Covariate**	% missing	Mean	% missing	Mean	Difference in Mean	NMD	Mean	Mean	Difference in Mean	NMD	p
Age at exposure	0.0	62.1044	0.0	66.6586	-4.5542	-0.5553	65.5192	65.2244	0.2948	0.0332	0.0
Body mass index	13.0	29.2508	31.7	29.9966	-0.7458	-0.1125	30.0686	30.0398	0.0314	0.0043	0.2197
gender_Female	0.0	0.5761	0.0	0.5488	0.0272	0.0549	0.556	0.5576	-0.0016	-0.0032	0.2662
race_Caucasian	0.0	0.8598	0.0	0.843	0.0167	0.0471	0.8409	0.8424	-0.0014	-0.0039	0.1784
race_African American	0.0	0.0466	0.0	0.0811	-0.0345	-0.1413	0.0623	0.0605	0.0017	0.0072	0.0127
race_Other/Unknown	0.0	0.0603	0.0	0.0586	0.0018	0.0075	0.0694	0.0687	0.0007	0.0027	0.3549
race_Asian	0.0	0.0333	0.0	0.0173	0.016	0.1017	0.0274	0.0284	-0.001	-0.006	0.0383
has_chickenpox_diagnosis	0.0	0.0018	0.0	0.0007	0.0011	0.0304	0.0013	0.0013	-0.0	-0.0006	0.842
has_diabetes_diagnosis	0.0	0.117	0.0	0.2091	-0.0921	-0.2513	0.2159	0.2073	0.0085	0.0209	0.0
has_depression_diagnosis	0.0	0.1703	0.0	0.1345	0.0357	0.0996	0.1794	0.1786	0.0008	0.0021	0.4607
has_stroke_diagnosis	0.0	0.0173	0.0	0.0269	-0.0097	-0.0657	0.0266	0.0254	0.0011	0.007	0.0158
has_hypertension_diagnosis	0.0	0.4503	0.0	0.4699	-0.0196	-0.0393	0.5301	0.5239	0.0062	0.0123	0.0
has_atherosclerosis_diagnosis	0.0	0.0261	0.0	0.0358	-0.0097	-0.0558	0.0402	0.0382	0.0019	0.01	0.0006
has_ischemic heart disease_diagnosis	0.0	0.0986	0.0	0.1494	-0.0507	-0.1544	0.1486	0.1448	0.0038	0.0108	0.0002

	Pre-Matching						Post-Matching				
	RZV (2+doses)		PPSV23				RZV (2+ doses)	PPSV23			
**Covariate**	% missing	Mean	% missing	Mean	Difference in Mean	NMD	Mean	Mean	Difference in Mean	NMD	p
has_chronic obstructive pulmonary disease_diagnosis	0.0	0.0402	0.0	0.1012	-0.0611	-0.2399	0.0776	0.0748	0.0028	0.0104	0.0003
has_obesity_diagnosis	0.0	0.2683	0.0	0.1559	0.1125	0.2777	0.2841	0.2814	0.0027	0.0059	0.0416
has_herpes_simplex_diagnosis	0.0	0.0427	0.0	0.0136	0.0291	0.1766	0.0301	0.0304	-0.0003	-0.0016	0.5829
has_herpes_zoster_diagnosis	0.0	0.0452	0.0	0.0222	0.0229	0.1273	0.0402	0.0391	0.001	0.0053	0.0672
has_non-steroidal anti-inflammatory drugs_first	0.0	0.3531	0.0	0.2101	0.143	0.3221	0.3303	0.3309	-0.0006	-0.0012	0.6853
has_antivirals_first	0.0	0.2706	0.0	0.117	0.1537	0.3963	0.2225	0.2215	0.001	0.0024	0.405
has_glucocorticoids_first	0.0	0.3214	0.0	0.1907	0.1307	0.3029	0.3146	0.3137	0.0009	0.002	0.4882

A normalized mean difference (NMD) greater than 0.1 indicates a potential imbalance between the cohorts. Covariates with NMD > 0.1 pre-matching are highlighted by red color of their respective pre-matching NMD. Covariates with NMD > 0.1 post-matching are highlighted by red background color of the corresponding row.

PPSV23, recipients of at least 1 dose of the 23-valent pneumococcal polysaccharide vaccine; RZV (2+ doses), recipients of at least 2 doses of the recombinant zoster vaccine (*Shingrix*, GSK).

Supplementary Table 19 | Variable definitions and codes

Disease/comorbidity	ICD-9 codes	ICD-10 codes
**Alzheimer's disease**	294.1	F00.*, G30.*
**Dementia**	290.2, 290.3, 290.4, 291.2, 294.1, 331.0, 331.1, 331.2, 331.5	F00, F00.0, F00.1, F00.2, F00.9, F01, F01.1, F01.2, F01.3, F01.8, F01.9, F02.8, F03, F05.1, G30, G30.0, G30.1, G30.8, G30.9, G31.1, I67.3, A81.0, F01.0, F02, F02.0, F02.1, F02.2, F02.3, F02.4, F10.6, G31.0, G31.8, I67.3
**Dementia (reduced)**	290.2, 290.3, 290.4, 291.2, 294.1, 331.0, 331.1, 331.2, 331.5	F00, F00.0, F00.1, F00.2, F00.9, F01, F01.1, F01.2, F01.3, F01.8, F01.9, F02.8, F03, F05.1, G30, G30.0, G30.1, G30.8, G30.9, G31.1, I67.3
**Dementia (extended)**	290.2, 290.3, 290.4, 291.2, 294.1, 331.0, 331.1, 331.2, 331.5	A81.0, F00, F00.0, F00.1, F00.2, F00.9, F01.*, F02.*, F03.*, F05, F05.1, F10.6, G30.*, G31.0*, G31.1, G31.8, I67.3
**Vascular dementia (extended)**	290.4	F01.*
**Dementia diseases (extended)**	294.1	F02.*

**Mild cognitive impairment**	331.83	331.83
**Mild cognitive impairment (extended)**	331.83, 330.9, 331.9, 780.93	G31.84, G31.9, R41, R41.3
**Cardiovascular condition**	272.0, 272.4, 427.3*, 398.91, 402.11, 402.91, 404.11, 404.13, 404.91, 428.*, 410.*, 411.*, 412.*, 413.*, 414.*, V45.81, V45.82, 440.*	E78.*, I48.*, I09.81, I11.0, I13.0, I13.2, I50.*, I20.*, I21.*, I22.*, I23.*, I24.*, I25.*, I70.*
**Chickenpox**	052.*	B01.*
**Cognitive decline**	797.*	R41.81
**Dependence care**	V60.4*, V63.*	Z74.*, Z75.*
**Depression**	296.20, 296.22, 296.23, 296.30, 296.32, 296.33, 311	F32.*, F33.*
**Diabetes**	250.00, 250.02	E11.*
**Falls/fractures**	805.4*, 805.5*, 806.4*, 806.5*, 805.6*, 805.7*, 806.6*, 806.7*, 808.*, 809.*, 839.2*, 839.3*, 839.41*, 839.42*, 839.43*, 839.44*, 839.45*, 839.46*, 839.47*, 839.48*, 839.49*, 839.50*, 839.51*, 839.52*, 846.*, 847.2*, 847.3*, 847.4*, 848.5*, 810.*, 811.*, 812.*, 831.*, 832.*, 833.*, 834.*, 835.*, 840.*,	S32.*, S33.*, S42.*, S43.*, S52.*, S53.*, S62.*, S63.*, S72.*, S73.*, W0%, W1%, M80.*, M81.*, R29.6*, R55.*, R54.*, M96.6*

	841.*, 842.*, 843.*, 839.61*, 839.71*, 848.4*, 813.*, 814.*, 815.*, 816.*, 817.*, 820.*, 821.*, E88.*, 733.0*, 733.1*, V15.88*, 780.2*, 797.*, 996.4*	
**Herpes simplex**	054.*	B00.*
**Herpes zoster**	053.*	B02.*
**Hyperlipidemia**	272.0, 272.4	E78.*
**Hypertension**	401.*	I10.*
**Incontinence**	788.3*, 787.6*	R32.*, R15.*
**Mobility problems**	781.2*, 781.99*, 728.3*	R26.*, R29.8*, M62.84*
**Stroke**	434.91	I61.*, I62.*, I63.*, I64.*
**Ulcers weight loss**	707.2*, 707.0*, 783.2*, V69.1*	L89.*, R63.4*, R63.6*, Z72.4*
**General examination**	V70.0	Z00.00
**Immunization encounter**	V05.9	Z23
**Drug therapy**	V58.6*	Z79*
**Viral infection**	070.*, 071, 072.*, 074.*, 075, 077.*, 078.*, 079.*, 372.*, 420.*, 422.*, 460.*, 464.*, 465.*, 466.*, 480.*, 487.*, 647.*, 711.*, 045.*, 046.*, 047.*, 048, 049.*, 050.*, 051.*, 052.*, 053.*, 054.*, 055.*, 056.*, 057.*, 060.*, 061, 062.*, 063.*, 064, 065.*, 066.*	A08.*, A60.*, A8%, A9%, B0%, B15.*, B16.*, B17.*, B18.*, B19.*, B20, B25.*, B26.*, B27.*, B30.*, B33.*, B34.*, B97.*, H10.*, I30.*, I40.*, J00, J04.*, J05.*, J06.*, J10.*, J12.*, J20.*, J21.*, O98.*, P35.*, Z21

**Bacterial infection**	001.*, 002.*, 003.*, 004.*, 005.*, 073.*, 076.*, 077.*, 078.*, 079.*, 080, 081.*, 082.*, 083.*, 320.*, 381.*, 382.*, 383.*, 421.*, 461.*, 475, 481, 482.*, 510.*, 567.*, 590.*, 595.*, 597.*, 670.*, 730.*, 01%, 02%, 03%, 040.*, 041.*, 09%, 100.*, 101, 102.*, 103.*, 104.*, 390, 391.*, 392.*, 614.*, 615.*, 616.*, 680.*, 681.*, 682.*, 683, 684, 685.*, 686.*	A00.*, A01.*, A02.*, A03.*, A04.*, A05.*, A1%, A2%, A3%, A4%, A50.*, A51.*, A52.*, A53.*, A54.*, A55, A56.*, A57, A58, A65, A66.*, A67.*, A68.*, A69.*, A7%, B95.*, B96.*, G00.*, G01, H60.*, H70.*, I00, I01.*, I02.*, I33.*, J01.*, J13, J14, J15.*, J36, J86.*, K65.*, L0%, M00.*, M86.*, N30.*, N34.*, N7%, O23.*, O85, O86.*, P36.*
**Other infection**	084.*, 085.*, 086.*, 087.*, 088.*, 112.*, 114.*, 117.*, 118, 120.*, 121.*, 122.*, 124, 125.*, 126.*, 128.*, 130.*, 131.*, 132.*, 133.*, 134.*, 135, 136.*, 370.*, 675.*, 771.*, 137.*, 138, 139.*	B37.*, B38.*, B45.*, B46.*, B47.*, B48.*, B49, B50.*, B51.*, B52.*, B53.*, B54, B55.*, B56.*, B57.*, B58.*, B60.*, B64, B65.*, B66.*, B67.*, B68.*, B69.*, B70.*, B72, B73.*, B74.*, B75, B76.*, B77.*, B78.*, B79, B80, B83.*, B85.*, B86, B87.*, B88.*, B89, B90.*, B91, B92, B94, B99.*, H16.*, H32, M01.*, M02.*, O91.*, P37.*, P38.*, P39.*, Z22.*, A59.*, A63.*, A64
**Atherosclerosis**	440.*	I70.*

**Chronic obstructive pulmonary disease**	491.*, 492.*, 496	J41.*, J42, J43.2, J43.8, J43.9, J44.*
**Ischemic heart disease**	410.*, 411.*, 412.*, 413.*, 414.*, V45.81, V45.82	I20.*, I21.*, I22.*, I23.*, I24.*, I25.*
**Heart failure**	398.91, 402.11, 402.91, 404.11, 404.13, 404.91, 428.*	I09.81, I11.0, I13.0, I13.2, I50.*
**Atrial fibrillation**	427.3*	I48.*
**Asthma**	493.*	J45.*
**Vitamin B12 deficiency**	266.2, 281.1	E53.8, D51.*
**Anxiety**	309.81, 300.00, 300.01, 300.02, 300.23, 300.3	F40.1*, F41.0*, F41.1*, F41.9*, F42, F34.1*
**Brain injury**	800.*, 801.*, 802.*, 803.*, 804.*, 850.*, 851.*, 852.*, 853.*, 854.*, 905.0, 907.0, 959.01, V15.52	S01.90*, S02.*, S04.*, S06.*, S07.*, S09.8*, S09.9*, Z87.820
**Obesity**	278.00, 278.0127803, V85.3*, V85.4*	E66.01, E66.09, E66.1, E66.2, E66.8, E66.9, Z68.3*, Z68.4*
**Alcohol disorder**	303.9*, 305.0*	F10.*
**Tobacco use**	V15.82, 305.1	Z87.891, Z72.0, F17.20*, F17.21*
**Substance use disorder**	304.0*, 304.1*, 304.2*, 304.3*, 304.4*, 304.5*, 304.6*, 304.7*, 304.8*, 304.9*, 305.2*, 305.3*, 305.4*, 305.5*, 305.6*, 305.7*, 305.9*	F11.*, F12.*, F13.*, F14.*, F15.*, F16.*, F18.*, F19.*

Note: For diagnoses, unless specified otherwise, presence of at least 1 code in the individual's records indicates presence

of the disease/comorbidity. Diagnoses are Boolean (0 = no diagnosis, 1 = diagnosis present). *Super-group and all its child codes.

ICD, International Classification of Diseases.

Supplementary Table 20 | Vaccine codes

Vaccine	NDC code	CPT code
**RZV**	58160081912, 58160082311	90750
**ZVL**	00006496341	90736
**PPSV23**	00006494300	90732
**PCV13**	00005197101, 00005197102, 00005197105	90670
**BCG**	00052060301, 00052060302	90586
**Tdap**	58160084211, 49281028601, 58160081052, 49281040015, 49281028605, 49281028610, 58160084252, 49281040005, 49281040020, 58160084234, 58160081011, 49281040010, 58160084251, 49281029810, 50090288300	90702, 90714, 90715
**Influenza**	19515080852, 19515081452, 19515081652, 19515081852, 19515084511, 19515085052, 19515088907, 19515089007, 19515089111, 19515089307, 19515089452, 19515089511, 19515089611, 19515089711, 19515089811, 19515090011, 19515090152, 19515090311,	90662, 90672, 90674, 90682, 90686, 90687, 90688, 90694, 90756

	19515090652, 19515090852,<br>19515090952, 19515091252,<br>33332001001, 33332001301,<br>33332001401, 33332001501,<br>33332001601, 33332001701,<br>33332001801, 33332011010,<br>33332011310, 33332011410,<br>33332011510, 33332011610,<br>33332011710, 33332011810,<br>33332021920, 33332022020,<br>33332022120, 33332031601,<br>33332031701, 33332031801,<br>33332031901, 33332032001,<br>33332032101, 33332032203,<br>33332032303, 33332041610,<br>33332041710, 33332041810,<br>33332041910, 33332042010,<br>33332042110, 33332042210,<br>33332042310, 42874001210,<br>42874001310, 42874001410, 42874001510,<br>42874001610,<br>42874001710, 42874011710,<br>49281001010, 49281001025,<br>49281001050, 49281001110,<br>49281001150, 49281001210,<br>49281001250, 49281001310,<br>49281001350, 49281001450,<br>49281011125, 49281011225,	

	49281011325, 49281012065,<br>49281012165, 49281012265,<br>49281012365, 49281032050,<br>49281032150, 49281032250,<br>49281032350, 49281033615,<br>49281033715, 49281033915,<br>49281035515, 49281037950,<br>49281038615, 49281038765,<br>49281038815, 49281038965,<br>49281039015, 49281039165,<br>49281039215, 49281039365,<br>49281039415, 49281039565,<br>49281039615, 49281039765,<br>49281039965, 49281040165,<br>49281040365, 49281040565,<br>49281041310, 49281041350,<br>49281041410, 49281041450,<br>49281041510, 49281041610,<br>49281041650, 49281041710,<br>49281041750, 49281041810,<br>49281041850, 49281041910,<br>49281041950, 49281042010,<br>49281042050, 49281042110,<br>49281042150, 49281042210,<br>49281042250, 49281042350,<br>49281051325, 49281051425,<br>49281051625, 49281051725,<br>49281051825, 49281051925,	

	49281052025, 49281052125,<br>49281062115, 49281062515,<br>49281062715, 49281062915,<br>49281063115, 49281063315,<br>49281063515, 49281063715,<br>49281063915, 49281070355,<br>49281070555, 49281070755,<br>49281070840, 49281070955,<br>49281071040, 49281071240,<br>49281071810, 49281071910,<br>49281072010, 49281072110,<br>49281072210, 49281072310, 54868617700,<br>54868618000, 58160087952, 58160088052,<br>58160088152, 58160088352,<br>58160088552, 58160088752,<br>58160089052, 58160089652,<br>58160089852, 58160090052,<br>58160090152, 58160090352,<br>58160090552, 58160090752,<br>58160090952, 62577061301,<br>62577061401, 63851061201,<br>63851061301, 66019010701,<br>66019010810, 66019010910,<br>66019011010, 66019030010,<br>66019030110, 66019030210,<br>66019030310, 66019030410,<br>66019030510, 66019030610,<br>66019030710, 66019030810,	

	66019030910, 66019031010, 66521000001, 66521011202, 66521011210, 66521011302, 66521011310, 66521011402, 66521011410, 66521011502, 66521011510, 66521011602, 66521011610, 66521011702, 66521011710, 66521011802, 66521011810, 70461000101, 70461000201, 70461001803, 70461001903, 70461002003, 70461011902, 70461011910, 70461012002, 70461012003, 70461012010, 70461012103, 70461012203, 70461012303, 70461020001, 70461020101, 70461030110, 70461031803, 70461031903, 70461032003, 70461032103, 70461032203, 70461032303, 70461041810, 70461041910, 70461042010, 70461042110, 70461042210, 70461042310, 76420048201, 76420048301	

BCG, Bacillus Calmette-Guérin vaccine; CPT, Current Procedural Terminology; NDC, National Drug Code; PCV13, 13-valent pneumococcal conjugate vaccine (*Prevenar 13*, manufactured by Pfizer); PPSV23, 23-valent pneumococcal polysaccharide vaccine; RZV, recombinant zoster vaccine (*Shingrix*, manufactured by GSK); Tdap, tetanus-diphtheria-acellular pertussis vaccine; ZVL, live-attenuated zoster vaccine (*Zostavax*, Merck).

Supplementary table 21 | Post-matching cohort characteristics for main comparisons, Inverse Probability of Treatment Weighting (IPTW)

Covariate	Multiple vs single HZ episode			ZVL vs PPSV23			RZV (2+ doses) vs PPSV23		
	HZ multiple N=391,437	HZ single N=393,406	p	ZVL N=2,628,740	PPSV23 N=2,584,519	p	RZV (2+ doses) N=1,087,804	PPSV23 N=1,051,869	p
**Mean age at exposure, years**	66.6	66.4	<0.001	66.4	66.4	<0.001	63.7	63.9	<0.001
**Mean BMI, kg/m^2**	31.1	29.0	NR	26.2	31.8	NR	29.0	30.2	NR
**Female gender, %**	66.4	66.0	<0.001	57.1	57.3	<0.001	56.6	56.9	<0.001
**Race, %**									
Caucasian	87.0	86.0	<0.001	90.5	90.7	<0.001	85.1	84.9	<0.001
African American	6.5	6.5	0.331	3.6	3.4	<0.001	5.5	5.6	<0.001
Asian	1.7	1.8	0.011	1.4	1.4	0.296	3.1	3.1	<0.001
Other/Unknown	4.8	5.8	<0.001	4.6	4.5	<0.001	6.4	6.4	<0.001
**Comorbidity diagnosis, %**									
Chickenpox	0.2	0.2	0.049	0.1	0.1	0.563	0.2	0.1	<0.001
Diabetes	19.4	19.9	<0.001	11.7	11.5	<0.001	16.5	17.1	<0.001

Depression	16.7	16.6	<0.001	8.8	8.6	<0.001	17.5	17.6	<0.001
Stroke	2.7	3.0	<0.001	1.1	1.1	0.005	2.2	2.3	<0.001
Hypertension	50.8	50.4	0.066	34.6	34.2	<0.001	49.0	50.0	<0.001
Atherosclerosis	4.7	4.6	0.088	1.7	1.7	<0.001	3.3	3.4	<0.001
Ischemic heart disease	16.7	16.6	0.775	8.7	8.7	<0.001	12.2	13.0	<0.001
Chronic obstructive pulmonary disease	11.8	11.8	0.141	4.1	4.0	<0.001	5.9	6.2	<0.001
Obesity	15.4	15.5	<0.001	9.2	9.0	<0.001	27.7	28.0	<0.001
Herpes simplex	3.1	2.7	<0.001	1.3	1.3	<0.001	3.7	3.6	<0.001
Herpes zoster	NA	NA	NA	2.1	2.1	<0.001	4.3	4.3	<0.001
**Prescribed medication, %**									
Non-steroidal anti-inflammatory drugs	26.0	26.3	<0.001	16.7	16.4	<0.001	34.4	34.2	<0.001
Antivirals	18.3	17.8	0.733	11.0	11.0	<0.001	24.9	24.7	<0.001
Glucocorticoids	27.6	26.6	<0.001	13.3	13.1	<0.001	31.8	32.2	0.152

%, percentage of individuals with a given characteristic; BMI, body mass index; Multiple and single HZ, individuals experiencing at least 2 (multiple) and 1 (single) episodes of herpes zoster (HZ); IPTW, Inverse Probability of Treatment Weighting; N, number of individuals included in the cohorts; NA, not applicable; NR, not reported; PPSV23, recipients of at least 1 dose of a 23-valent pneumococcal polysaccharide vaccine; RZV (2+ doses), recipients of at least 2 doses of the recombinant zoster vaccine (*Shingrix*, GSK); ZVL, recipients of at least 1 dose of the live-attenuated zoster vaccine (*Zostavax*, Merck).

Supplementary table 22 | Post-matching cohort characteristics for main comparisons, Overlap Weights (OW)

Covariate	Multiple vs single HZ episode			ZVL vs PPSV23			RZV (2+ doses) vs PPSV23		
	HZ multiple N=74,624	HZ single N=74,542	p	ZVL N=508,561	PPSV23 N=508,822	p	RZV (2+ doses) N=171,957	PPSV23 N=172,772	p
**Mean age at exposure, years**	66.5	66.3	<0.001	66.5	66.4	<0.001	65.3	65.3	<0.001
**Mean BMI, kg/m^2**	30.4	27.4	NR	25.8	24.1	NR	28.4	36.4	NR
**Female gender, %**	66.2	65.7	<0.001	56.9	57.0	<0.001	55.6	55.7	<0.001
**Race, %**									
Caucasian	86.9	86.0	<0.001	89.7	89.8	<0.001	84.1	84.1	<0.001
African American	6.6	6.4	0.645	4.2	4.1	<0.001	6.3	6.2	<0.001
Asian	1.7	1.8	<0.001	1.4	1.4	<0.001	2.7	2.8	<0.001
Other/Unknown	4.8	5.7	<0.001	4.7	4.7	<0.001	6.9	6.8	0.019
**Comorbidity diagnosis, %**									
Chickenpox	0.2	0.2	0.057	0.1	0.1	0.632	0.1	0.1	0.168
Diabetes	19.5	19.8	<0.001	13.0	12.9	<0.001	21.1	21.1	<0.001

Depression	16.6	16.6	<0.001	8.9	8.9	<0.001	17.9	18.1	<0.001
Stroke	2.7	2.9	<0.001	1.2	1.2	<0.001	2.6	2.7	<0.001
Hypertension	50.7	50.5	0.103	35.1	35.0	<0.001	52.9	53.1	<0.001
Atherosclerosis	4.6	4.6	0.504	1.9	1.9	<0.001	3.9	4.0	<0.001
Ischemic heart disease	16.6	16.5	0.077	9.3	9.3	<0.001	14.7	14.7	<0.001
Chronic obstructive pulmonary disease	11.6	11.6	<0.001	4.8	4.7	<0.001	7.8	7.8	<0.001
Obesity	15.9	15.9	<0.001	9.5	9.5	<0.001	28.4	28.4	<0.001
Herpes simplex	3.1	2.7	<0.001	1.1	1.2	<0.001	3.0	3.0	<0.001
Herpes zoster	NA	NA	NA	2.0	2.0	<0.001	3.9	3.9	<0.001
**Prescribed medication, %**									
Non-steroidal anti-inflammatory drugs	26.4	26.6	<0.001	16.2	16.2	<0.001	33.2	33.2	<0.001
Antivirals	18.2	17.9	0.691	9.7	9.7	<0.001	22.4	22.3	<0.001
Glucocorticoids	27.8	26.9	<0.001	13.3	13.3	<0.001	31.5	31.7	<0.001

%, percentage of individuals with a given characteristic; BMI, body mass index; HZ (multiple) and HZ (single), individuals experiencing at least 2 (multiple) and 1 (single) episodes of herpes zoster (HZ); N, number of individuals included in the cohorts; NA, not applicable; NR, not reported; OW, overlap weighting; PPSV23, recipients of at least 1 dose of a 23-valent pneumococcal polysaccharide vaccine; RZV (2+ doses), recipients of at least 2 doses of the recombinant zoster vaccine (*Shingrix*, GSK); ZVL, recipients of at least 1 dose of the live-attenuated zoster vaccine (*Zostavax*, Merck).

Supplementary table 23 | Post-matching cohort characteristics for primary comparisons

Covariate	Multiple vs single HZ N (per cohort)=97,342			ZVL vs PPSV23 N (per cohort)=703,263			RZV (2+ doses) vs PPSV23 N (per cohort)=234,309		
	HZ multiple	HZ single	p	ZVL	PPSV23	p	RZV (2+ doses)	PPSV23	p
**Median age at exposure (IQR), years**	67 (58, 75)	67 (59, 75)	0.004	65 (61, 72)	65 (61, 72)	<0.001	65 (59, 71)	65 (59, 70)	<0.001
**Median BMI (IQR), kg/m^2**	28.3 (24.7, 32.6)	28.3 (24.7, 32.8)	0.379	28.8 (25.4, 33.1)	28.8 (25.4, 33.0)	0.196	29.1 (25.5, 33.6)	29.0 (25.5, 33.6)	0.147
**Female gender, %**	66.7	66.3	0.039	57.1	57.1	0.544	55.5	55.7	0.172
**Race, %**									
Caucasian	87.1	85.9	<0.001	90.1	90.4	<0.001	84.0	84.1	0.349
African American	6.6	6.7	0.439	3.9	3.7	<0.001	6.2	6.2	0.168
Asian	1.7	1.9	0.002	1.4	1.4	0.942	2.7	2.9	0.007
Other/Unknown	4.6	5.5	<0.001	4.6	4.6	0.180	7.0	6.9	0.071
**Comorbidity diagnosis, %**									
Chickenpox	0.2	0.2	0.100	0.1	0.1	0.323	0.1	0.1	0.592

Diabetes	19.3	19.7	0.013	12.7	12.3	<0.001	21.5	20.8	<0.001
Depression	16.2	16.4	0.239	8.7	8.6	0.010	17.8	17.8	0.593
Stroke	2.7	2.9	0.001	1.2	1.1	0.025	2.6	2.6	0.056
Hypertension	50.4	50.8	0.087	34.6	34.1	<0.001	52.9	52.3	<0.001
Atherosclerosis	4.5	4.7	0.041	1.8	1.7	0.013	4.0	3.8	0.003
Ischemic heart disease	16.5	16.7	0.240	9.2	8.9	<0.001	14.8	14.4	<0.001
Chronic obstructive pulmonary disease	11.6	12.0	0.010	4.5	4.3	<0.001	7.8	7.4	<0.001
Obesity	15.2	15.2	0.749	9.3	9.2	0.018	28.3	28.3	0.858
Herpes simplex	3.1	2.7	<0.001	1.1	1.2	0.099	2.9	3.0	0.115
Herpes zoster	NA	NA	NA	2.0	2.0	0.501	3.9	3.9	0.545
**Prescribed medication, %**									
Non-steroidal anti-inflammatory drugs	25.5	25.9	0.076	16.0	16.0	0.366	32.8	33.1	0.087
Antivirals	18.0	18.2	0.182	9.5	9.7	<0.001	22.2	22.2	0.874
Glucocorticoids	26.9	26.3	0.007	13.1	13.0	0.421	31.1	31.5	0.008

%, percentage of individuals with a given characteristic; BMI, body mass index; N, number of individuals included in the cohorts; HZ, herpes zoster; IQR, interquartile range (25th percentile, 75th percentile); NA, not applicable; PPSV23, recipients of at least 1 dose of a 23-valent pneumococcal polysaccharide vaccine; RZV (2+ doses), recipients of at least 2 doses of the recombinant zoster vaccine (*Shingrix*, GSK); ZVL, recipients of at least 1 dose of the live-attenuated zoster vaccine (*Zostavax*, Merck).

Supplementary table 24 | Post-matching cohort characteristics for RZV (2+ doses) and comparator cohorts

Covariate	RZV (2+ doses) vs ZVL N (per cohort)=56,320			RZV (2+ doses) vs RZV (1 dose) N (per cohort)=416,765		
	RZV (2+ doses)	ZVL	p	RZV (2+ doses)	RZV (1 dose)	p
**Median age at exposure (IQR), years**	63 (60, 69)	64 (59, 70)	<0.001	64 (58, 71)	64 (58, 71)	0.381
**Median BMI (IQR), kg/m^2**	28.8 (25.3, 33.1)	28.7 (25.2, 33.0)	0.305	28.9 (25.3, 33.3)	28.8 (25.3, 33.3)	0.183
**Female gender, %**	56.1	56.1	0.995	56.4	56.4	0.808
**Race, %**						
Caucasian	85.9	82.4	<0.001	82.8	83.1	<0.001
African American	5.2	5.6	0.001	7.7	7.4	<0.001
Asian	2.0	2.5	<0.001	2.8	2.9	0.022
Other/Unknown	7.0	9.5	<0.001	6.7	6.7	0.166
**Comorbidity diagnosis, %**						
Chickenpox	0.1	0.1	0.264	0.2	0.2	0.488
Diabetes	16.3	17.0	0.002	21.6	21.6	0.537
Depression	14.1	15.0	<0.001	21.9	21.7	0.189
Stroke	1.9	2.0	0.130	3.3	3.2	0.129
Hypertension	45.4	45.4	0.919	55.9	55.8	0.449
Atherosclerosis	2.9	3.3	<0.001	5.3	5.2	0.181
Ischemic heart disease	11.1	11.3	0.252	16.6	16.6	0.899
Chronic obstructive pulmonary disease	6.7	7.0	0.108	9.9	9.6	<0.001
Obesity	20.6	23.2	<0.001	32.4	32.3	0.541

Herpes simplex	2.5	2.7	0.023	3.9	4.0	0.744
Herpes zoster	3.7	3.9	0.046	5.2	5.1	0.200
**Prescribed medication, %**						
Non-steroidal anti-inflammatory drugs	26.8	27.6	0.004	40.0	39.9	0.384
Antivirals	22.0	24.3	<0.001	31.3	31.4	0.605
Glucocorticoids	24.3	26.2	<0.001	37.6	37.5	0.389

%, percentage of individuals with a given characteristic; BMI, body mass index; IQR, interquartile range (the 25th and the 75th percentile); N, number of individuals included in the cohorts; RZV (2+ doses), recipients of at least 2 doses of the recombinant zoster vaccine (*Shingrix*, GSK); RZV (1 dose), recipients of only 1 dose of RZV; ZVL, recipients of at least 1 dose of the live-attenuated zoster vaccine (*Zostavax*, Merck).

Supplementary table 25 | Post-matching cohort characteristics for comparisons in women

Covariate	ZVL vs PPSV23 (women ≥50 years) N (per cohort)=401,303			RZV (2+ doses) vs PPSV23 (women 80–89 years) N (per cohort)=12,352		
	ZVL	PPSV23	p	RZV (2+ doses)	PPSV23	p
**Median age at exposure (IQR), years**	65 (61, 73)	65 (61, 72)	<0.001	84 (81, 85)	84 (81, 85)	0.779
**Median BMI (IQR), kg/m^2**	28.5 (24.5, 33.5)	28.4 (24.6, 33.3)	0.349	26.5 (23.3, 30.3)	26.5 (23.3, 30.2)	0.533
**Race, %**						
Caucasian	89.7	90.0	<0.001	88.7	89.0	0.384
African American	4.5	4.2	<0.001	4.2	3.9	0.220
Asian	1.5	1.4	0.613	2.1	2.0	0.468
Other/Unknown	4.4	4.4	0.587	5.0	5.1	0.749
**Comorbidity diagnosis, %**						
Chickenpox	0.1	0.1	0.408	0.1	0.1	1.000
Diabetes	11.3	11.1	0.001	20.2	20.9	0.144
Depression	10.9	10.8	0.026	17.0	16.7	0.505
Stroke	1.0	1.0	0.162	6.1	6.1	0.895
Hypertension	33.0	32.6	<0.001	69.1	69.9	0.179
Atherosclerosis	1.5	1.5	0.884	10.1	9.7	0.313
Ischemic heart disease	6.1	5.9	0.004	22.7	23.2	0.316
Chronic obstructive pulmonary disease	4.3	4.1	<0.001	12.7	12.5	0.675
Obesity	9.4	9.2	0.004	17.2	16.8	0.453
Herpes simplex	1.4	1.4	0.108	1.7	1.6	0.841

Herpes zoster	2.2	2.3	0.046	6.9	6.5	0.254
**Prescribed medication, %**						
Non-steroidal anti-inflammatory drugs	16.7	16.8	0.260	27.5	27.1	0.432
Antivirals	10.1	10.3	0.066	25.4	23.5	0.001
Glucocorticoids	13.8	13.9	0.520	29.5	28.3	0.032

%, percentage of individuals with a given characteristic; BMI, body mass index; IQR, interquartile range (the 25th and the 75th percentile); N, number of individuals included in the cohorts; PPSV23, recipients of at least 1 dose of a 23-valent pneumococcal polysaccharide vaccine; RZV (2+ doses), recipients of at least 2 doses of the recombinant zoster vaccine (*Shingrix*, GSK); ZVL, recipients of at least 1 dose of the live-attenuated zoster vaccine (*Zostavax*, Merck).

Supplementary table 26 | Post-matching cohort characteristics for comparisons with the Not exposed cohorts

Covariate	ZVL vs Not exposed N (per cohort)=531,312			RZV (2+ doses) vs Not exposed N (per cohort)=490,910		
	ZVL	Not exposed	p	RZV (2+ doses)	Not exposed	p
**Median age at exposure (IQR), years**	65 (61, 72)	65 (61, 73)	<0.001	62 (57, 69)	61 (56, 69)	<0.001
**Median BMI (IQR), kg/m^2**	29.1 (25.5, 33.6)	29.3 (25.6, 33.9)	<0.001	29.1 (25.5, 33.8)	29.4 (25.6, 34.2)	<0.001
**Female gender, %**	59.6	59.8	0.051	59.4	60.7	<0.001
**Race, %**						
Caucasian	90.9	89.8	<0.001	86.1	85.3	<0.001
African American	5.3	6.3	<0.001	6.9	7.9	<0.001
Asian	1.3	1.3	0.026	2.7	2.3	<0.001
Other/Unknown	2.6	2.6	0.010	4.3	4.6	<0.001
**Comorbidity diagnosis, %**						
Chickenpox	0.2	0.2	<0.001	0.3	0.2	0.365
Diabetes	27.2	29.5	<0.001	25.2	27.2	<0.001
Depression	22.3	24.1	<0.001	26.5	29.1	<0.001
Stroke	3.2	3.7	<0.001	3.6	4.3	<0.001
Hypertension	68.2	70.1	<0.001	63.0	64.6	<0.001
Atherosclerosis	5.5	6.4	<0.001	6.0	6.8	<0.001
Ischemic heart disease	20.9	23.2	<0.001	18.7	20.6	<0.001
Chronic obstructive pulmonary disease	11.5	13.1	<0.001	10.7	12.0	<0.001
Obesity	23.9	24.5	<0.001	40.7	42.6	<0.001
Herpes simplex	3.0	3.5	<0.001	5.2	5.6	<0.001
Herpes zoster	5.4	5.8	<0.001	6.6	7.0	<0.001
**Prescribed medication, %**						
Non-steroidal anti-inflammatory drugs	42.0	42.6	<0.001	50.7	54.1	<0.001
Antivirals	30.3	30.7	<0.001	35.7	36.9	<0.001

Glucocorticoids	36.1	38.7	<0.001	48.2	51.7	<0.001

%, percentage of individuals with a given characteristic; BMI, body mass index; IQR, interquartile range (the 25th and the 75th percentile); N, number of individuals included in the cohorts; Not exposed, individuals not exposed to the comparator vaccine; RZV (2+ doses), recipients of at least 2 doses of the recombinant zoster vaccine (*Shingrix*, GSK); ZVL, recipients of at least 1 dose of the live-attenuated zoster vaccine (*Zostavax*, Merck).

Supplementary table 27 | Post-matching characteristics of cohorts from different dementia definitions

Covariate	Code[a] AND Medication						Code[a] OR Medication					
	ZVL vs PPSV23 N (per cohort)=703,221			RZV (2+ doses) vs PPSV23 N (per cohort)=233,179			ZVL vs PPSV23 N (per cohort)=699,594			RZV (2+ doses) vs PPSV23 N (per cohort)=236,003		
	ZVL	PPSV23	p	RZV (2+ doses)	PPSV23	p	ZVL	PPSV23	p	RZV (2+ doses)	PPSV23	p
**Median age at exposure (IQR), years**	65 (61, 72)	65 (61, 72)	<0.001	65 (59, 71)	65 (59, 70)	<0.001	65 (61, 72)	65 (61, 72)	<0.001	65 (59, 71)	65 (59, 70)	<0.001
**Median BMI (IQR), kg/m^2**	28.8 (25.3, 33.1)	28.8 (25.3, 33.1)	0.103	29.1 (25.5, 33.6)	29.0 (25.4, 33.5)	0.159	28.8 (25.4, 33.1)	28.8 (25.4, 33.1)	0.791	29.1 (25.5, 33.6)	29.0 (25.5, 33.5)	0.109
**Female gender, %**	57.0	57.2	0.088	55.6	55.5	0.837	56.9	57.2	<0.001	55.4	55.4	0.970
**Race, %**												
Caucasian	90.1	90.4	<0.001	84.1	84.3	0.020	90.1	90.5	<0.001	84.1	84.2	0.228
African American	3.9	3.7	<0.001	6.2	6.0	0.045	3.9	3.7	<0.001	6.2	6.1	0.021
Asian	1.4	1.4	0.728	2.8	2.9	0.121	1.4	1.4	0.015	2.8	2.9	0.094

Other/Unknown	4.6	4.5	0.070	7.0	6.8	0.014	4.6	4.5	0.001	6.9	6.9	0.516
**Comorbidity diagnosis, %**												
Chickenpox	0.1	0.1	0.634	0.1	0.1	0.902	0.1	0.1	0.410	0.1	0.1	0.342
Diabetes	12.7	12.4	<0.001	21.5	20.6	<0.001	12.7	12.3	<0.001	21.5	20.8	<0.001
Depression	8.7	8.7	0.195	17.8	17.7	0.296	8.6	8.6	0.496	17.7	17.6	0.525
Stroke	1.2	1.1	0.042	2.6	2.5	0.015	1.2	1.1	0.004	2.6	2.5	0.298
Hypertension	34.6	34.3	<0.001	52.8	52.1	<0.001	34.5	34.1	<0.001	52.8	52.3	<0.001
Atherosclerosis	1.8	1.8	0.156	4.0	3.8	0.005	1.8	1.7	0.025	3.9	3.8	0.001
Ischemic heart disease	9.2	9.0	<0.001	14.8	14.4	0.002	9.1	8.9	<0.001	14.7	14.4	0.014
Chronic obstructive pulmonary disease	4.5	4.4	<0.001	7.7	7.4	<0.001	4.5	4.3	<0.001	7.7	7.4	<0.001
Obesity	9.3	9.2	0.353	28.1	28.1	0.749	9.3	9.2	0.010	28.4	28.1	0.012
Herpes simplex	1.1	1.1	0.689	2.9	3.0	0.002	1.1	1.2	0.062	3.0	3.1	0.063
Herpes zoster	2.0	2.0	0.022	3.9	3.9	0.820	2.0	2.0	0.067	3.9	3.9	0.626
**Prescribed medication, %**												

Non-steroidal anti-inflammatory drugs	16.0	16.0	0.846	32.8	32.8	0.958	15.9	15.8	0.018	32.9	32.9	0.811
Antivirals	9.6	9.7	0.001	21.8	22.1	0.075	9.5	9.7	<0.001	22.1	22.1	0.883
Glucocorticoids	13.0	13.1	0.420	31.1	31.3	0.137	13.0	12.9	0.112	31.2	31.5	0.046

[a]Dementia diagnosis code based on International Classification of Disease 9th and 10th revision (see Supplementary Table 2 for details); %, percentage of individuals with a given characteristic; BMI, body mass index; IQR, interquartile range (the 25th and the 75th percentile); N, number of individuals included in the cohorts; PPSV23, recipients of at least 1 dose of a 23-valent pneumococcal polysaccharide vaccine; RZV (2+ doses), recipients of at least 2 doses of the recombinant zoster vaccine (*Shingrix*, GSK); ZVL, recipients of at least 1 dose of the live-attenuated zoster vaccine (*Zostavax*, Merck).

Supplementary Table 28 | Post-matching characteristics of cohorts for different dementia subtypes

Covariate	Alzheimer's disease						Vascular dementia					
	ZVL vs PPSV23 N (per cohort)=704,999			RZV (2+ doses) vs PPSV23 N (per cohort)=237,740			ZVL vs PPSV23 N (per cohort)=705,419			RZV (2+ doses) vs PPSV23 N (per cohort)=238,681		
	ZVL	PPSV23	p	RZV (2+ doses)	PPSV23	p	ZVL	PPSV23	p	RZV (2+ doses)	PPSV23	p
**Median age at exposure (IQR), years**	65 (61, 72)	65 (61, 72)	<0.001	65 (59, 71)	65 (59, 70)	<0.001	65 (61, 72)	65 (61, 72)	<0.001	65 (59, 71)	65 (59, 70)	<0.001
**Median BMI (IQR), kg/m^2**	28.8 (25.3, 33.1)	28.8 (25.3, 33.1)	0.875	29.1 (25.5, 33.6)	29.1 (25.5, 33.6)	0.052	28.8 (25.3, 33.1)	28.8 (25.4, 33.1)	0.979	29.1 (25.5, 33.6)	29.0 (25.4, 33.5)	0.220
**Female gender, %**	57.1	57.2	0.463	55.4	55.5	0.240	57.2	57.1	0.413	55.6	55.8	0.266
**Race, %**												
Caucasian	90.1	90.3	<0.001	84.2	84.2	0.583	90.1	90.2	0.023	84.1	84.2	0.178
African American	3.9	3.7	<0.001	6.2	6.2	0.223	3.9	3.8	<0.001	6.2	6.1	0.013
Asian	1.4	1.4	0.443	2.7	2.8	0.001	1.4	1.4	0.324	2.7	2.8	0.038

Other/Unknown	4.6	4.6	0.568	6.9	6.8	0.068	4.6	4.6	0.847	6.9	6.9	0.355
**Comorbidity diagnosis, %**												
Chickenpox	0.1	0.1	0.676	0.1	0.1	0.488	0.1	0.1	0.892	0.1	0.1	0.842
Diabetes	12.8	12.4	<0.001	21.6	20.8	<0.001	12.7	12.5	<0.001	21.6	20.7	<0.001
Depression	8.8	8.6	0.010	17.9	17.9	0.691	8.7	8.7	0.120	17.9	17.9	0.461
Stroke	1.2	1.2	0.180	2.7	2.6	0.083	1.2	1.1	0.017	2.7	2.5	0.016
Hypertension	34.8	34.2	<0.001	53.1	52.4	<0.001	34.6	34.5	0.027	53.0	52.4	<0.001
Atherosclerosis	1.8	1.7	<0.001	4.0	3.9	0.498	1.8	1.8	0.101	4.0	3.8	0.001
Ischemic heart disease	9.2	8.9	<0.001	14.8	14.6	0.023	9.2	9.1	0.306	14.9	14.5	<0.001
Chronic obstructive pulmonary disease	4.5	4.3	<0.001	7.8	7.4	<0.001	4.5	4.4	<0.001	7.8	7.5	<0.001
Obesity	9.3	9.2	0.004	28.3	28.3	0.571	9.2	9.2	0.791	28.4	28.1	0.042
Herpes simplex	1.1	1.2	0.006	2.9	3.1	0.025	1.1	1.2	0.010	3.0	3.0	0.583
Herpes zoster	2.0	2.0	0.616	3.9	3.9	0.374	2.0	2.0	0.183	4.0	3.9	0.067
**Prescribed medication, %**												
Non-steroidal anti-inflammatory drugs	16.1	15.9	0.024	33.1	33.1	0.566	15.9	16.0	0.200	33.0	33.1	0.685

Antivirals	9.5	9.7	<0.001	22.1	22.4	0.050	9.5	9.8	<0.001	22.3	22.2	0.405
Glucocorticoids	13.1	13.0	0.072	31.4	31.5	0.750	13.0	13.0	0.279	31.5	31.4	0.488

%, percentage of individuals with a given characteristic; BMI, body mass index; IQR, interquartile range (25th percentile, 75th percentile); N, number of included individuals; PPSV23, recipients of at least 1 dose of the 23-valent pneumococcal polysaccharide vaccine; RZV (2+ doses), recipients of at least 2 doses of the recombinant zoster vaccine (*Shingrix*, GSK); ZVL, recipients of at least 1 dose of the live-attenuated zoster vaccine (*Zostavax*, Merck).

(a) Dementia: HZ (multiple) vs HZ (single) (all)

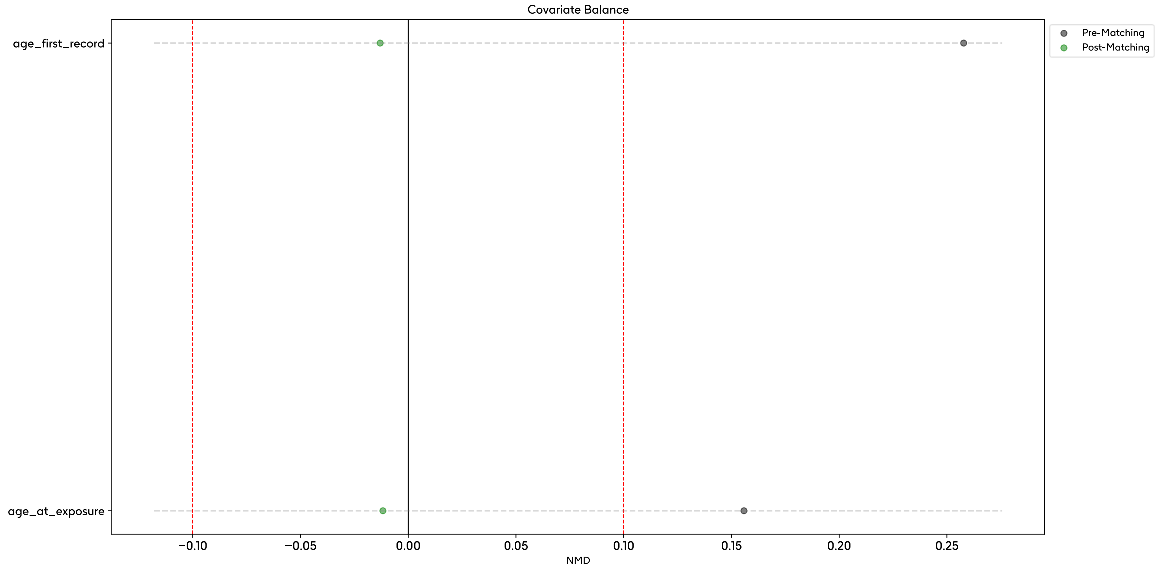

(b) Dementia: ZVL vs PPSV23 (all)

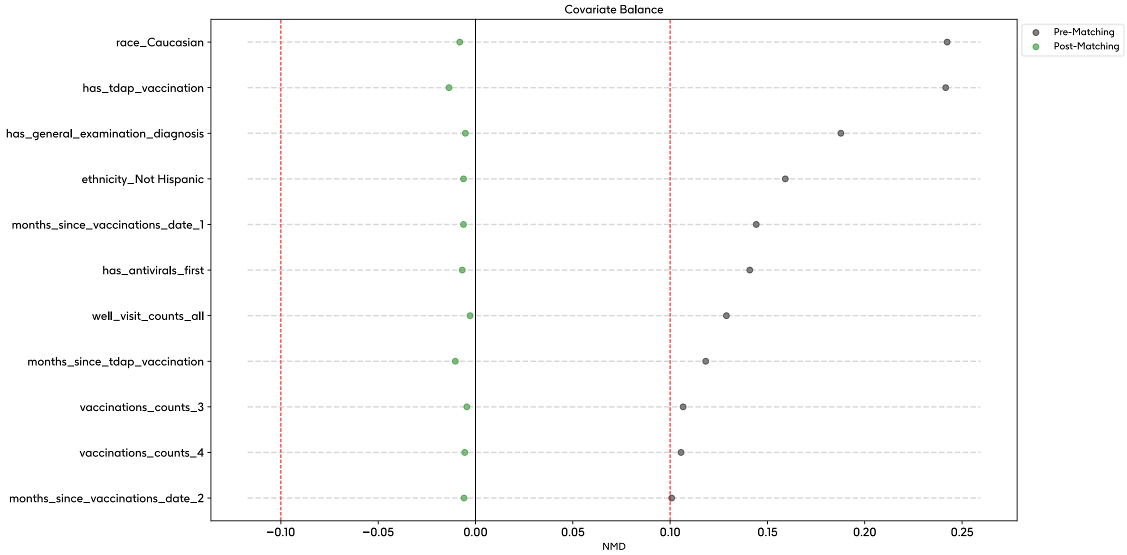

(c) Dementia: RZV (2+ doses) vs PPSV23 (all)

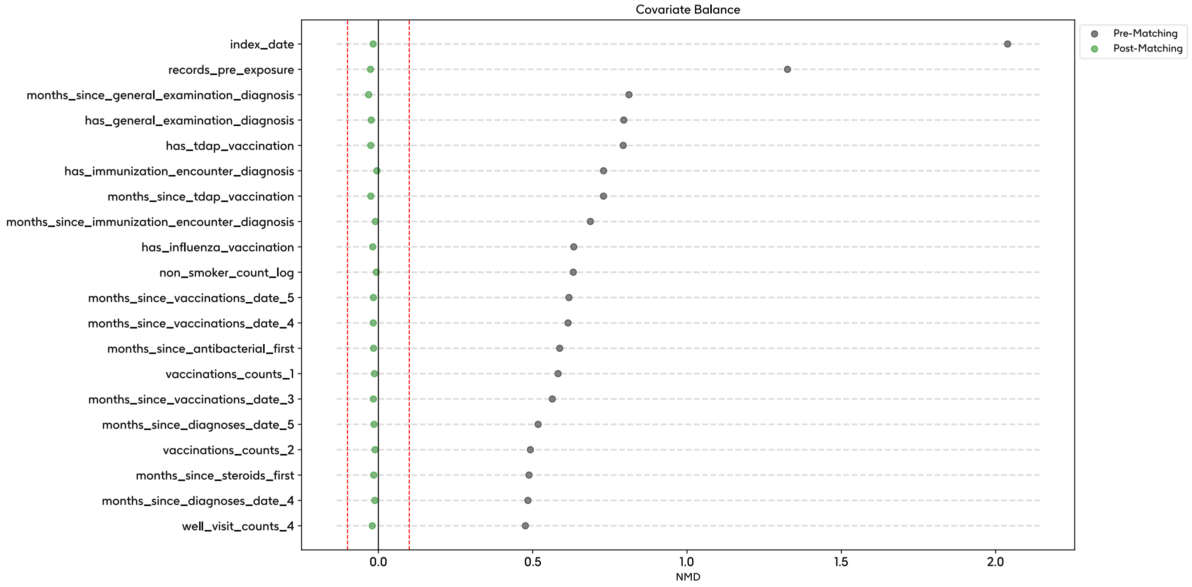

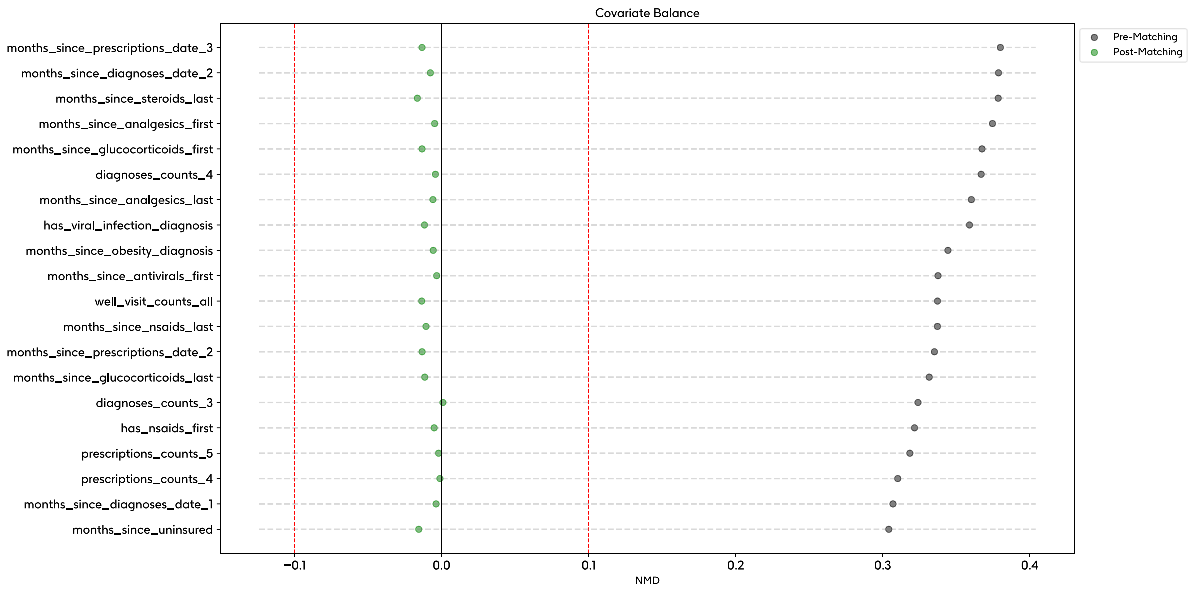
Covariate Balance
months_since_prescriptions_date_3
months_since_diagnoses_date_2
months_since_steroids_last
months_since_analgesics_first
months_since_glucocorticoids_first
diagnoses_counts_4
months_since_analgesics_last
has_viral_infection_diagnosis
months_since_obesity_diagnosis
months_since_antivirals_first
well_visit_counts_all
months_since_nsaids_last
months_since_prescriptions_date_2
months_since_glucocorticoids_last
diagnoses_counts_3
has_nsaids_first
prescriptions_counts_5
prescriptions_counts_4
months_since_diagnoses_date_1
months_since_uninsured
−0.1
0.0
0.1
0.2
0.3
0.4
NMD
Pre-Matching
Post-Matching

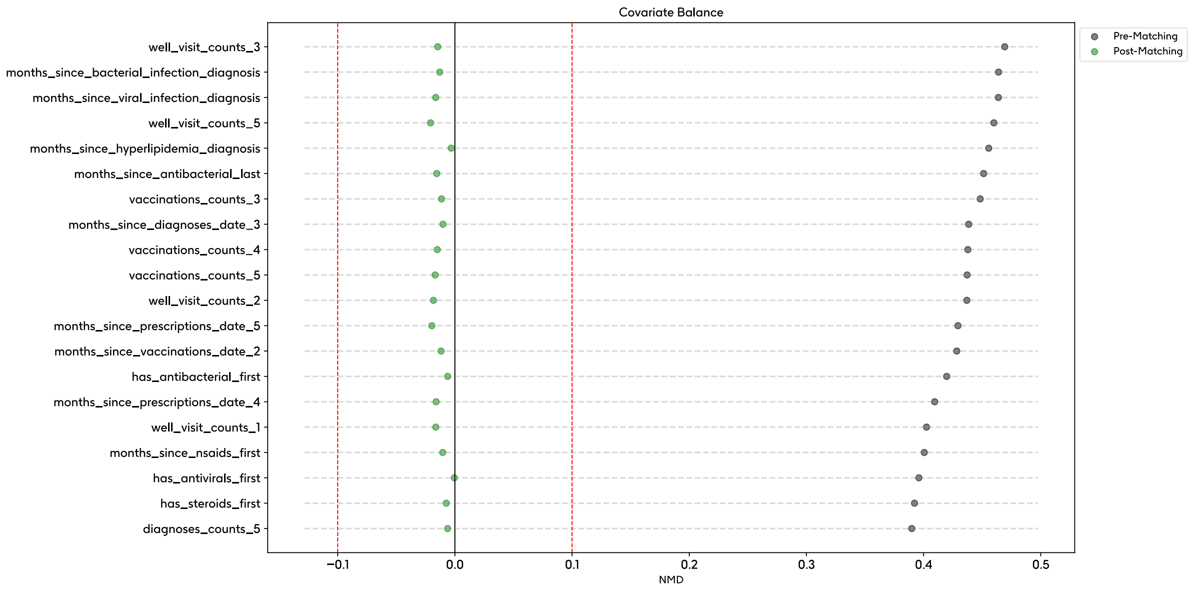
Covariate Balance
well_visit_counts_3
months_since_bacterial_infection_diagnosis
months_since_viral_infection_diagnosis
well_visit_counts_5
months_since_hyperlipidemia_diagnosis
months_since_antibacterial_last
vaccinations_counts_3
months_since_diagnoses_date_3
vaccinations_counts_4
vaccinations_counts_5
well_visit_counts_2
months_since_prescriptions_date_5
months_since_vaccinations_date_2
has_antibacterial_first
months_since_prescriptions_date_4
well_visit_counts_1
months_since_nsaids_first
has_antivirals_first
has_steroids_first
diagnoses_counts_5
−0.1
0.0
0.1
0.2
0.3
0.4
0.5
NMD
Pre-Matching
Post-Matching

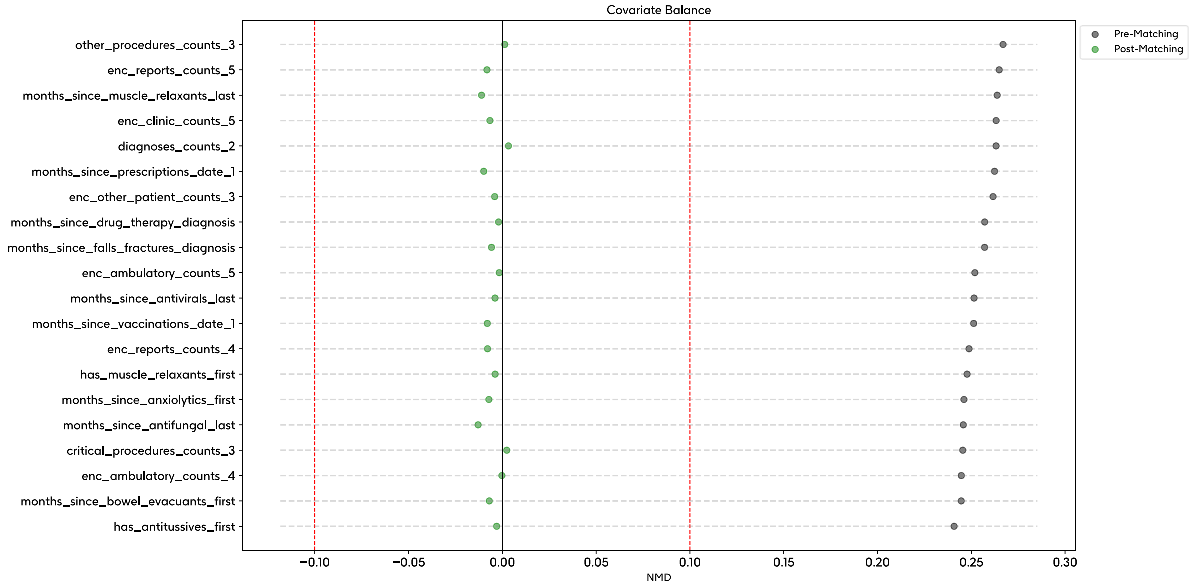
Covariate Balance
Pre-Matching
Post-Matching
other_procedures_counts_3
enc_reports_counts_5
months_since_muscle_relaxants_last
enc_clinic_counts_5
diagnoses_counts_2
months_since_prescriptions_date_1
enc_other_patient_counts_3
months_since_drug_therapy_diagnosis
months_since_falls_fractures_diagnosis
enc_ambulatory_counts_5
months_since_antivirals_last
months_since_vaccinations_date_1
enc_reports_counts_4
has_muscle_relaxants_first
months_since_anxiolytics_first
months_since_antifungal_last
critical_procedures_counts_3
enc_ambulatory_counts_4
months_since_bowel_evacuants_first
has_antitussives_first
−0.10
−0.05
0.00
0.05
0.10
0.15
0.20
0.25
0.30
NMD

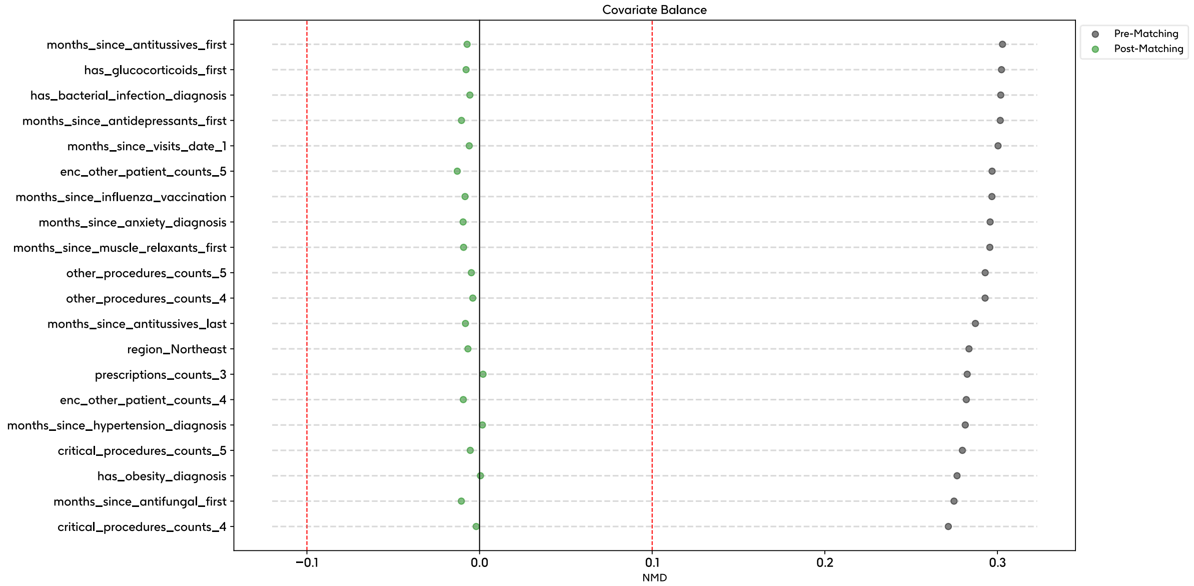
Covariate Balance
Pre-Matching
Post-Matching
months_since_antitussives_first
has_glucocorticoids_first
has_bacterial_infection_diagnosis
months_since_antidepressants_first
months_since_visits_date_1
enc_other_patient_counts_5
months_since_influenza_vaccination
months_since_anxiety_diagnosis
months_since_muscle_relaxants_first
other_procedures_counts_5
other_procedures_counts_4
months_since_antitussives_last
region_Northeast
prescriptions_counts_3
enc_other_patient_counts_4
months_since_hypertension_diagnosis
critical_procedures_counts_5
has_obesity_diagnosis
months_since_antifungal_first
critical_procedures_counts_4
−0.1
0.0
0.1
0.2
0.3
NMD

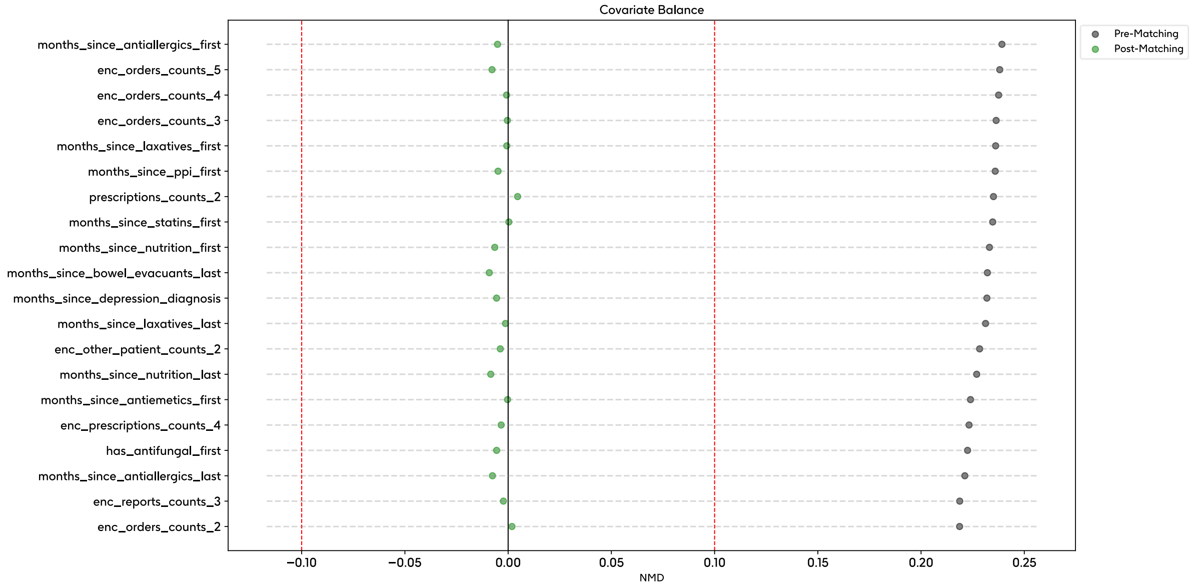
Covariate Balance
Pre-Matching
Post-Matching
months_since_antiallergics_first
enc_orders_counts_5
enc_orders_counts_4
enc_orders_counts_3
months_since_laxatives_first
months_since_ppi_first
prescriptions_counts_2
months_since_statins_first
months_since_nutrition_first
months_since_bowel_evacuants_last
months_since_depression_diagnosis
months_since_laxatives_last
enc_other_patient_counts_2
months_since_nutrition_last
months_since_antiemetics_first
enc_prescriptions_counts_4
has_antifungal_first
months_since_antiallergics_last
enc_reports_counts_3
enc_orders_counts_2
−0.10
−0.05
0.00
0.05
0.10
0.15
0.20
0.25
NMD

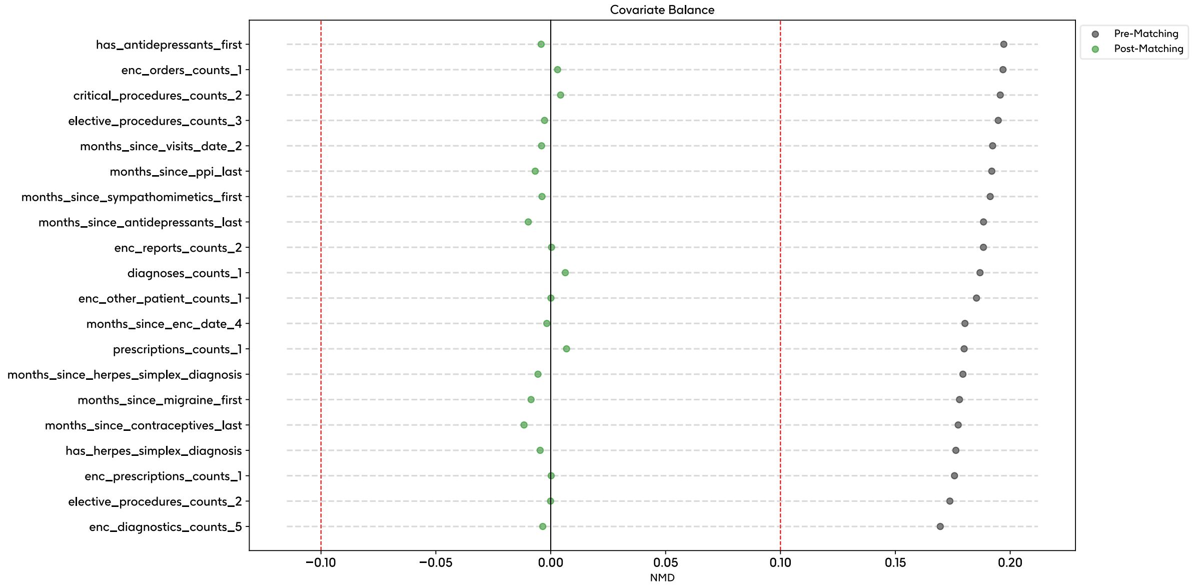
Covariate Balance
has_antidepressants_first
enc_orders_counts_1
critical_procedures_counts_2
elective_procedures_counts_3
months_since_visits_date_2
months_since_ppi_last
months_since_sympathomimetics_first
months_since_antidepressants_last
enc_reports_counts_2
diagnoses_counts_1
enc_other_patient_counts_1
months_since_enc_date_4
prescriptions_counts_1
months_since_herpes_simplex_diagnosis
months_since_migraine_first
months_since_contraceptives_last
has_herpes_simplex_diagnosis
enc_prescriptions_counts_1
elective_procedures_counts_2
enc_diagnostics_counts_5
Pre-Matching
Post-Matching
−0.10
−0.05
0.00
0.05
0.10
0.15
0.20
NMD

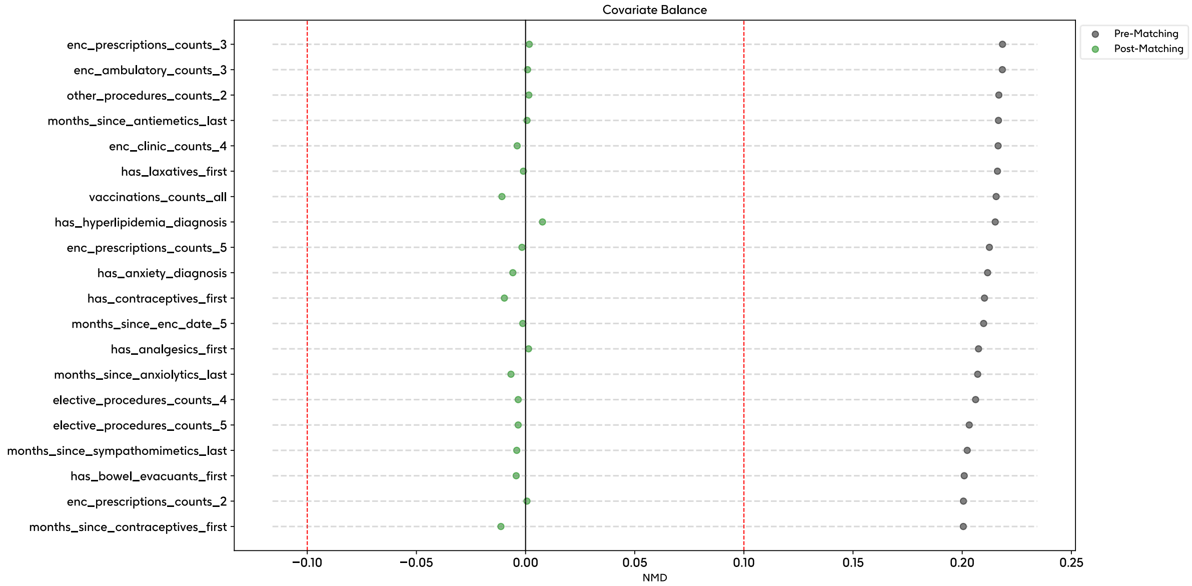
Covariate Balance
enc_prescriptions_counts_3
enc_ambulatory_counts_3
other_procedures_counts_2
months_since_antiemetics_last
enc_clinic_counts_4
has_laxatives_first
vaccinations_counts_all
has_hyperlipidemia_diagnosis
enc_prescriptions_counts_5
has_anxiety_diagnosis
has_contraceptives_first
months_since_enc_date_5
has_analgesics_first
months_since_anxiolytics_last
elective_procedures_counts_4
elective_procedures_counts_5
months_since_sympathomimetics_last
has_bowel_evacuants_first
enc_prescriptions_counts_2
months_since_contraceptives_first
Pre-Matching
Post-Matching
−0.10
−0.05
0.00
0.05
0.10
0.15
0.20
0.25
NMD

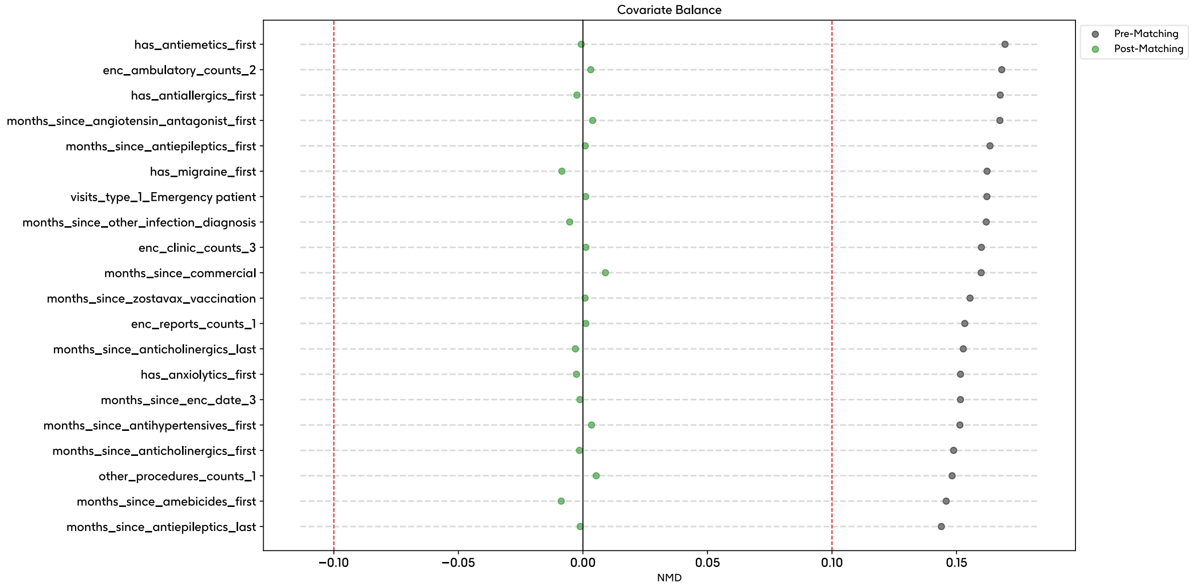
Covariate Balance
has_antiemetics_first
enc_ambulatory_counts_2
has_antiallergics_first
months_since_angiotensin_antagonist_first
months_since_antiepileptics_first
has_migraine_first
visits_type_1_Emergency patient
months_since_other_infection_diagnosis
enc_clinic_counts_3
months_since_commercial
months_since_zostavax_vaccination
enc_reports_counts_1
months_since_anticholinergics_last
has_anxiolytics_first
months_since_enc_date_3
months_since_antihypertensives_first
months_since_anticholinergics_first
other_procedures_counts_1
months_since_amebicides_first
months_since_antiepileptics_last
Pre-Matching
Post-Matching
−0.10
−0.05
0.00
0.05
0.10
0.15
NMD

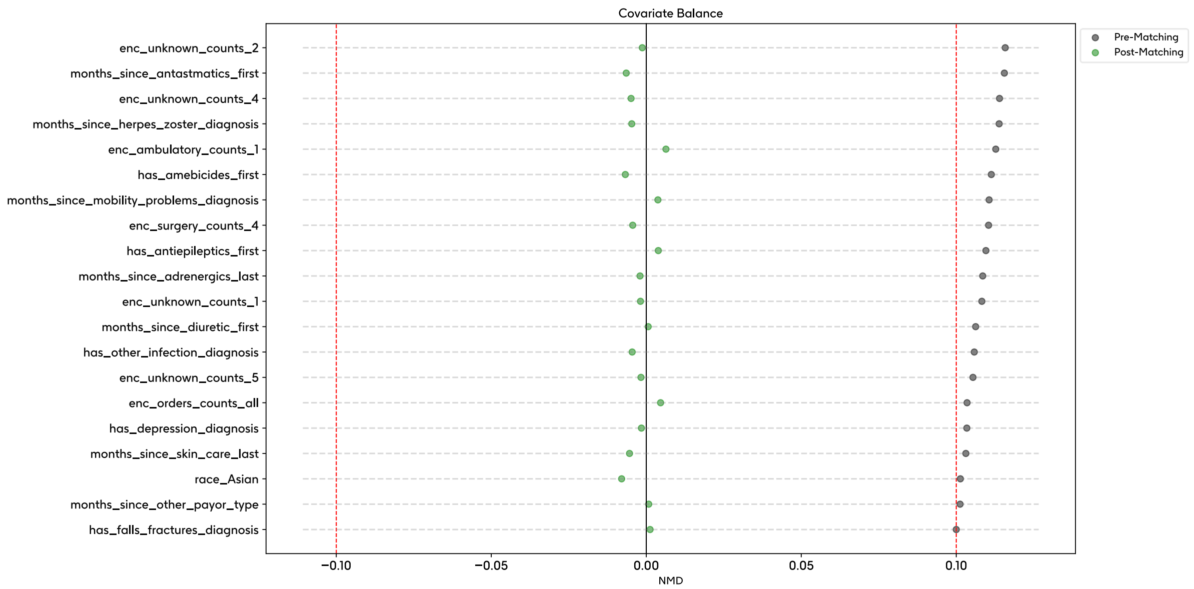

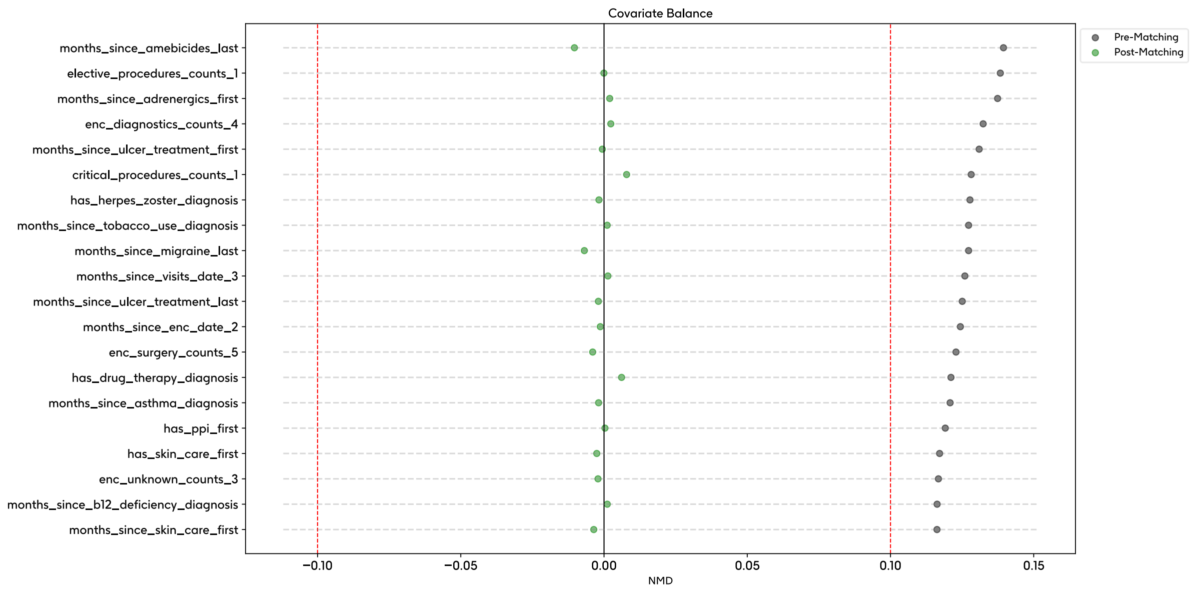

Supplementary Fig. S1 | Pre- and post-matching cohort balance for primary comparisons.
The values on the y-axis are the names of covariates. The dots indicate pre- and post-matching normalized mean distance (NMD) for the corresponding covariate. The red vertical lines indicate the bounds of what is considered a good balance (-0.1 < NMD < 0.1). Post-matching dots within these bounds indicate that the corresponding covariate's mean is well balanced after matching. The covariates are sorted in descending order of their pre-matching NMD.
Note for interpretation of covariate names: For covariates named "months_since_i" (where i=1, 2, 3, 4, 5), the name indicates months between ith last intervention pre-exposure and the exposure date. For "months_since_vaccination", the term "vaccination" refers to any vaccination except the exposure vaccination). For "months_since_diagnosis" covariate, the term "diagnosis" refers to any diagnosis in the Diagnosis table with a certain status. For covariates named "intervention_counts_i", the name indicates the number of interventions in the ith pre-exposure year. The covariate named "well_visits_counts_all" indicates the total count of routine medical and gynecological examinations.
HZ (multiple) and HZ (single), individuals experiencing at least 2 (multiple) and 1 (single) episodes of herpes zoster (HZ); PPSV23, recipients of at least 1 dose of a 23-valent

pneumococcal polysaccharide vaccine; RZV (2+ doses), recipients of at least 2 doses of the recombinant zoster vaccine (*Shingrix*, GSK); ZVL, recipients of at least 1 dose of the live-attenuated zoster vaccine (*Zostavax*, Merck).

(a) Dementia: RZV (2+ doses) vs ZVL (all)

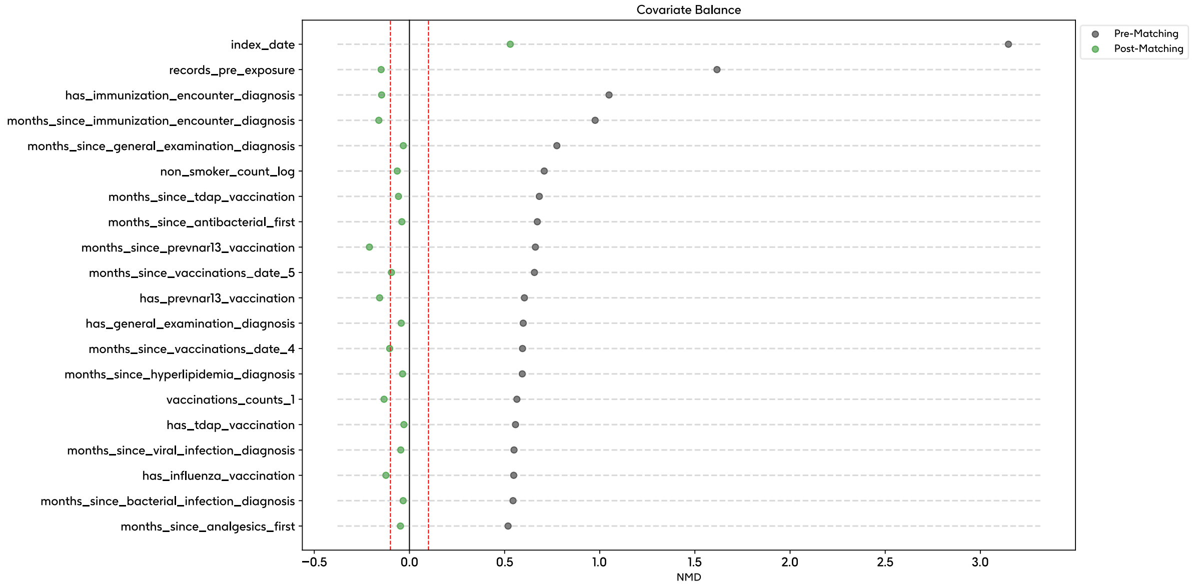

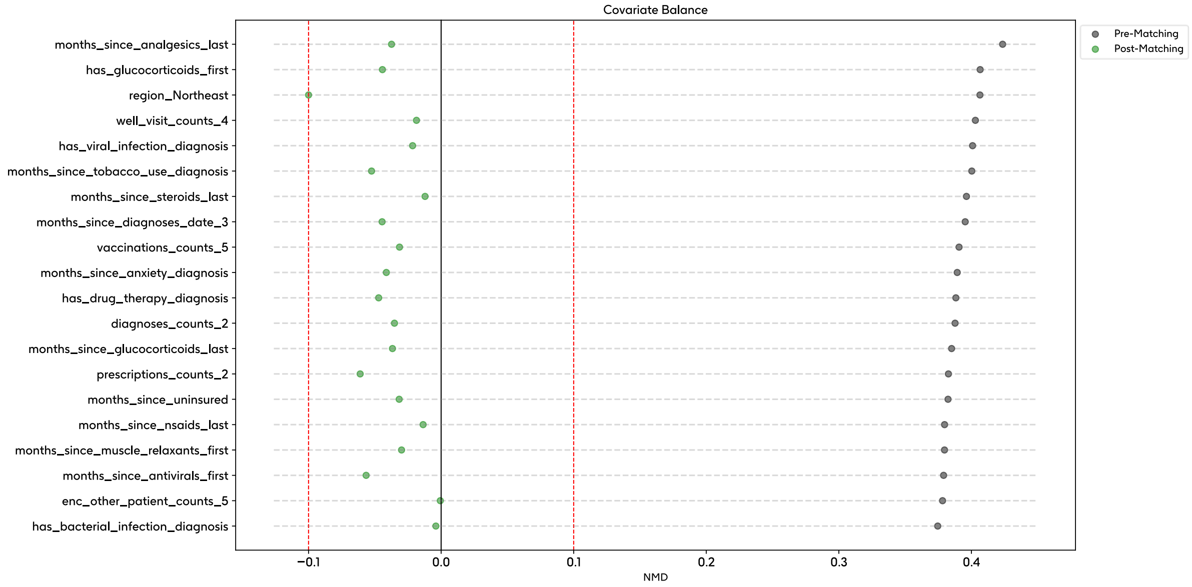

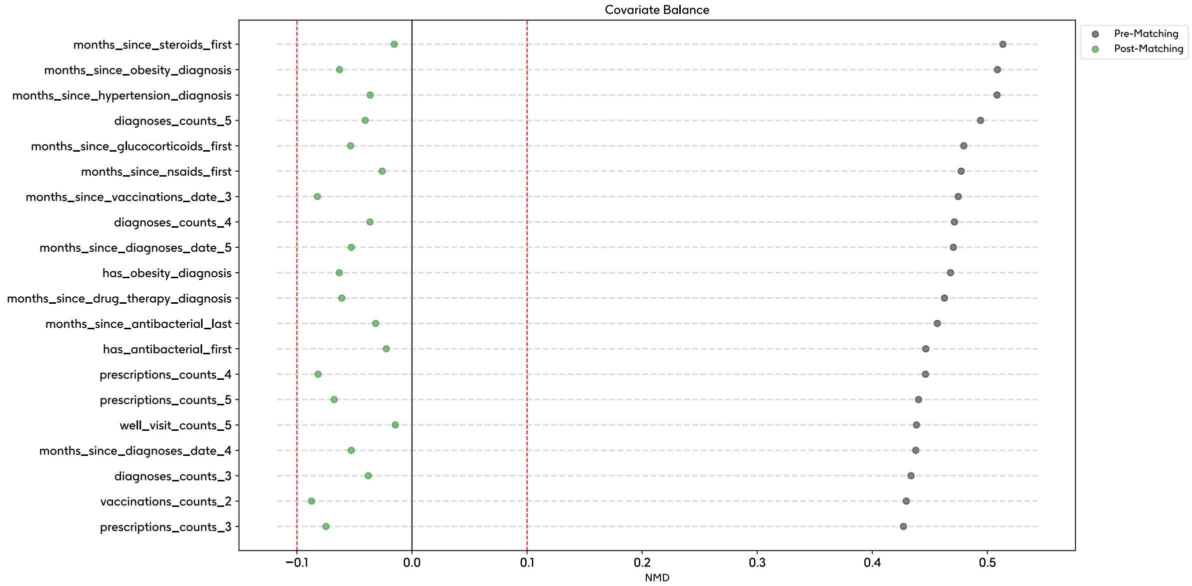
Covariate Balance
months_since_steroids_first
months_since_obesity_diagnosis
months_since_hypertension_diagnosis
diagnoses_counts_5
months_since_glucocorticoids_first
months_since_nsaids_first
months_since_vaccinations_date_3
diagnoses_counts_4
months_since_diagnoses_date_5
has_obesity_diagnosis
months_since_drug_therapy_diagnosis
months_since_antibacterial_last
has_antibacterial_first
prescriptions_counts_4
prescriptions_counts_5
well_visit_counts_5
months_since_diagnoses_date_4
diagnoses_counts_3
vaccinations_counts_2
prescriptions_counts_3
Pre-Matching
Post-Matching
−0.1
0.0
0.1
0.2
0.3
0.4
0.5
NMD

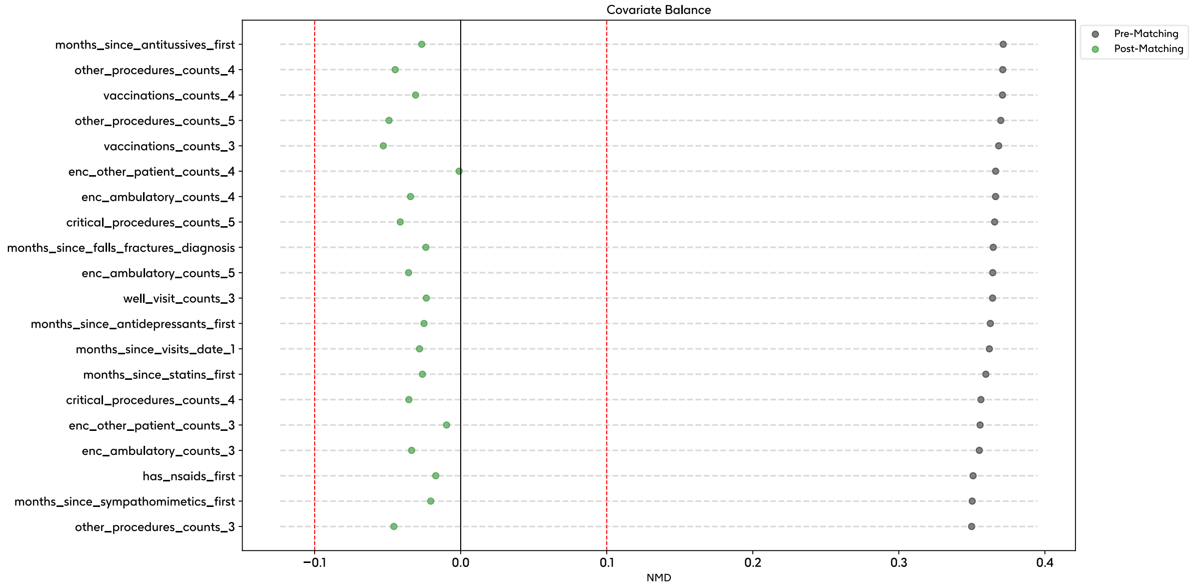
Covariate Balance
months_since_antitussives_first
other_procedures_counts_4
vaccinations_counts_4
other_procedures_counts_5
vaccinations_counts_3
enc_other_patient_counts_4
enc_ambulatory_counts_4
critical_procedures_counts_5
months_since_falls_fractures_diagnosis
enc_ambulatory_counts_5
well_visit_counts_3
months_since_antidepressants_first
months_since_visits_date_1
months_since_statins_first
critical_procedures_counts_4
enc_other_patient_counts_3
enc_ambulatory_counts_3
has_nsaids_first
months_since_sympathomimetics_first
other_procedures_counts_3
Pre-Matching
Post-Matching
−0.1
0.0
0.1
0.2
0.3
0.4
NMD

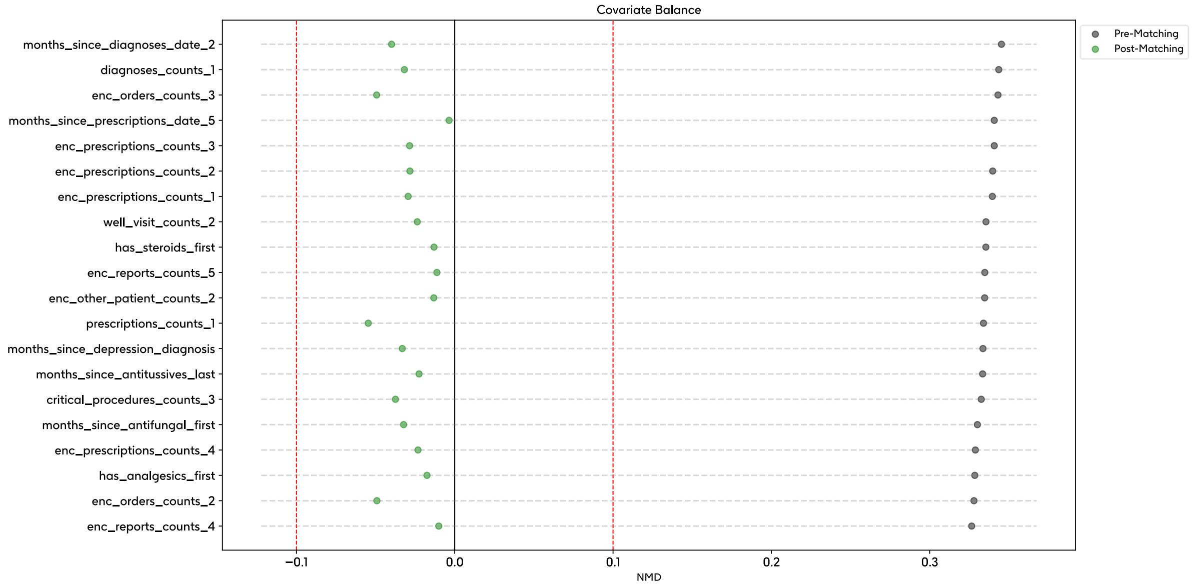
Covariate Balance
months_since_diagnoses_date_2
diagnoses_counts_1
enc_orders_counts_3
months_since_prescriptions_date_5
enc_prescriptions_counts_3
enc_prescriptions_counts_2
enc_prescriptions_counts_1
well_visit_counts_2
has_steroids_first
enc_reports_counts_5
enc_other_patient_counts_2
prescriptions_counts_1
months_since_depression_diagnosis
months_since_antitussives_last
critical_procedures_counts_3
months_since_antifungal_first
enc_prescriptions_counts_4
has_analgesics_first
enc_orders_counts_2
enc_reports_counts_4
Pre-Matching
Post-Matching
−0.1
0.0
0.1
0.2
0.3
NMD

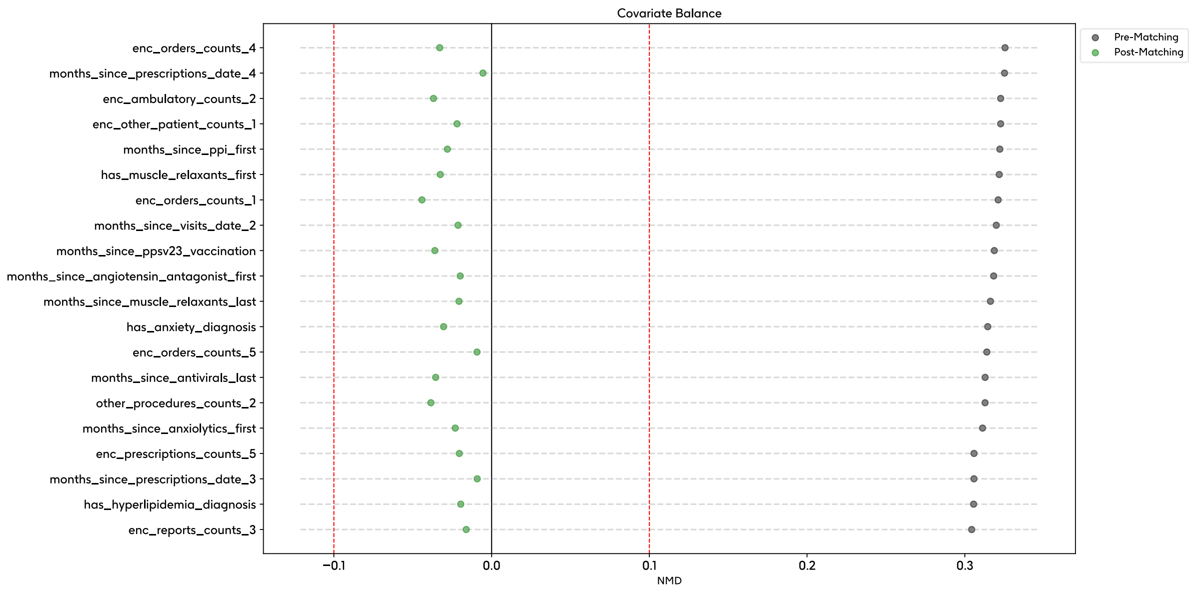

Covariate Balance
enc_orders_counts_4
months_since_prescriptions_date_4
enc_ambulatory_counts_2
enc_other_patient_counts_1
months_since_ppi_first
has_muscle_relaxants_first
enc_orders_counts_1
months_since_visits_date_2
months_since_ppsv23_vaccination
months_since_angiotensin_antagonist_first
months_since_muscle_relaxants_last
has_anxiety_diagnosis
enc_orders_counts_5
months_since_antivirals_last
other_procedures_counts_2
months_since_anxiolytics_first
enc_prescriptions_counts_5
months_since_prescriptions_date_3
has_hyperlipidemia_diagnosis
enc_reports_counts_3
Pre-Matching
Post-Matching
−0.1
0.0
0.1
0.2
0.3
NMD

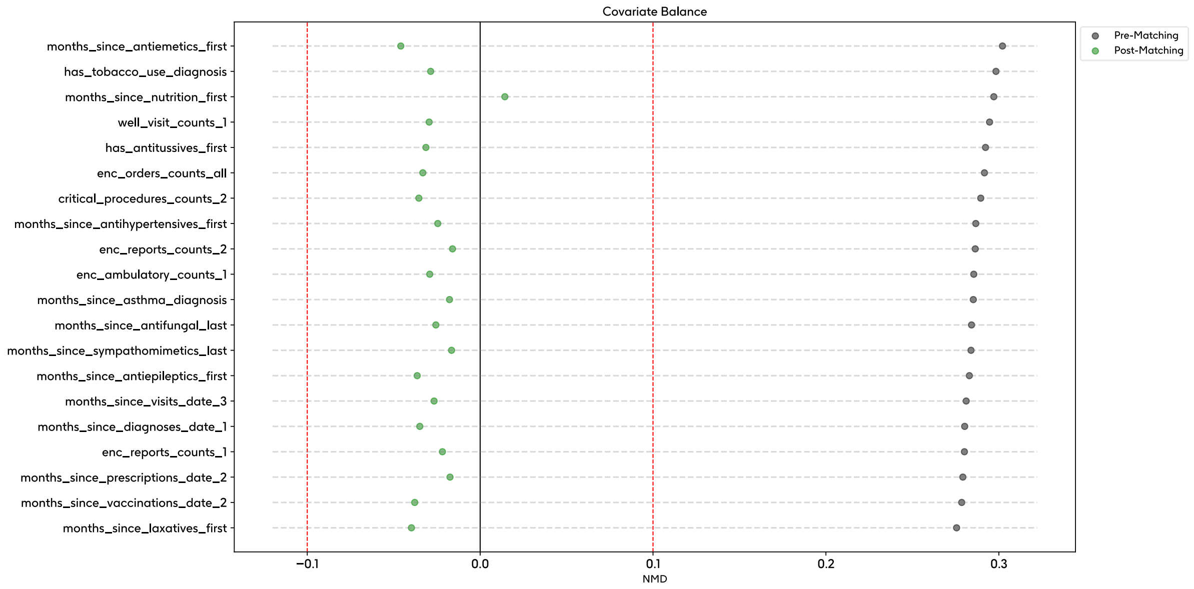

Covariate Balance
months_since_antiemetics_first
has_tobacco_use_diagnosis
months_since_nutrition_first
well_visit_counts_1
has_antitussives_first
enc_orders_counts_all
critical_procedures_counts_2
months_since_antihypertensives_first
enc_reports_counts_2
enc_ambulatory_counts_1
months_since_asthma_diagnosis
months_since_antifungal_last
months_since_sympathomimetics_last
months_since_antiepileptics_first
months_since_visits_date_3
months_since_diagnoses_date_1
enc_reports_counts_1
months_since_prescriptions_date_2
months_since_vaccinations_date_2
months_since_laxatives_first
Pre-Matching
Post-Matching
−0.1
0.0
0.1
0.2
0.3
NMD

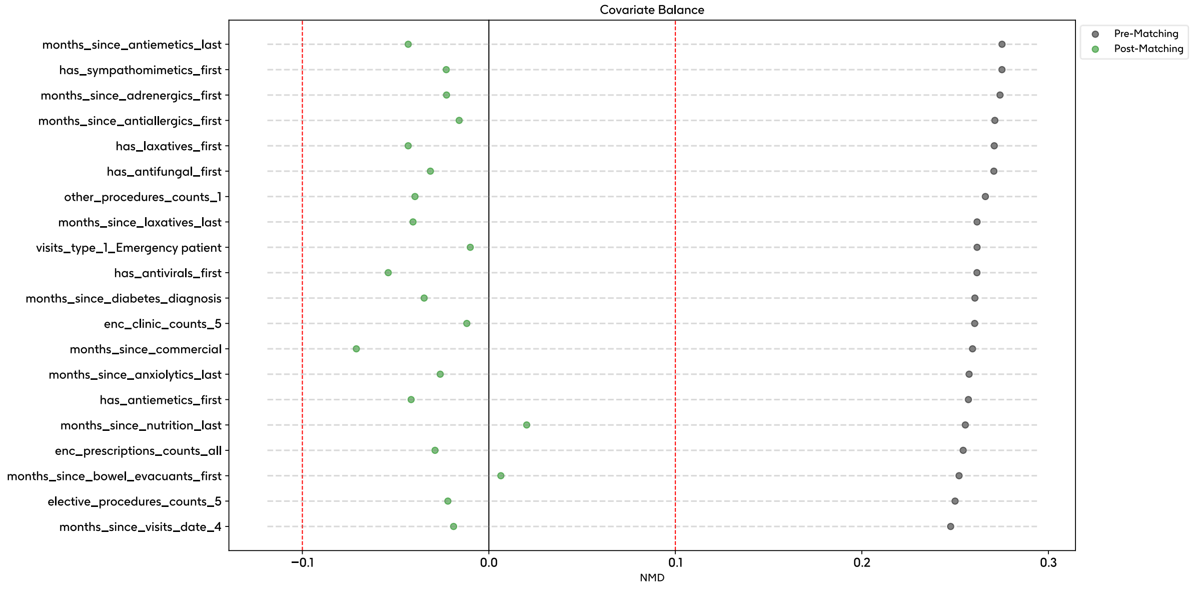
Covariate Balance
months_since_antiemetics_last
has_sympathomimetics_first
months_since_adrenergics_first
months_since_antiallergics_first
has_laxatives_first
has_antifungal_first
other_procedures_counts_1
months_since_laxatives_last
visits_type_1_Emergency patient
has_antivirals_first
months_since_diabetes_diagnosis
enc_clinic_counts_5
months_since_commercial
months_since_anxiolytics_last
has_antiemetics_first
months_since_nutrition_last
enc_prescriptions_counts_all
months_since_bowel_evacuants_first
elective_procedures_counts_5
months_since_visits_date_4
Pre-Matching
Post-Matching
−0.1
0.0
0.1
0.2
0.3
NMD

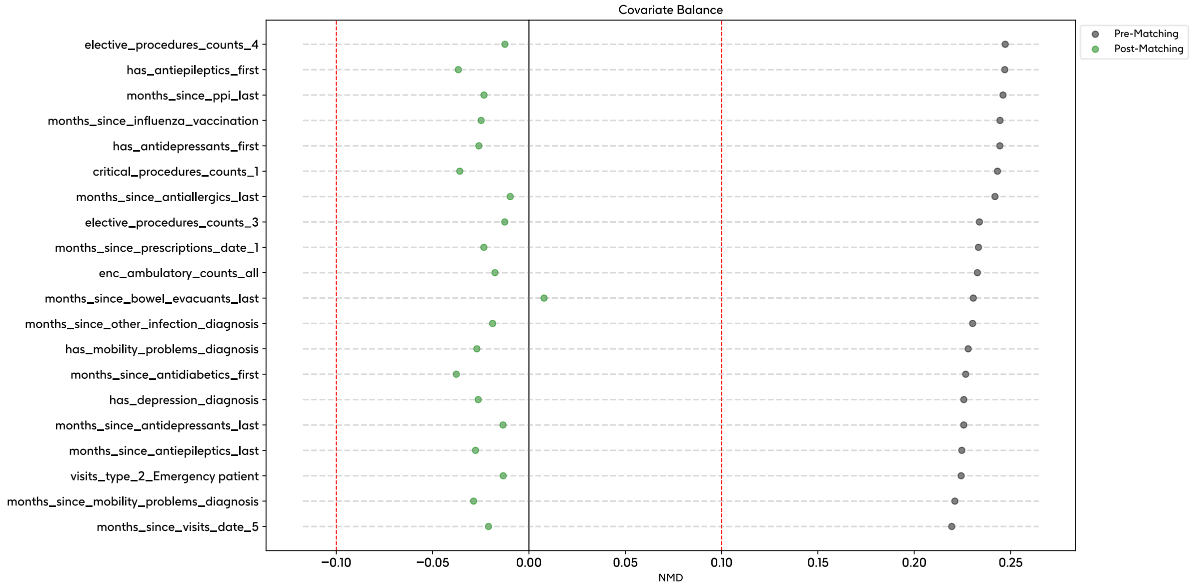
Covariate Balance
elective_procedures_counts_4
has_antiepileptics_first
months_since_ppi_last
months_since_influenza_vaccination
has_antidepressants_first
critical_procedures_counts_1
months_since_antiallergics_last
elective_procedures_counts_3
months_since_prescriptions_date_1
enc_ambulatory_counts_all
months_since_bowel_evacuants_last
months_since_other_infection_diagnosis
has_mobility_problems_diagnosis
months_since_antidiabetics_first
has_depression_diagnosis
months_since_antidepressants_last
months_since_antiepileptics_last
visits_type_2_Emergency patient
months_since_mobility_problems_diagnosis
months_since_visits_date_5
Pre-Matching
Post-Matching
−0.10
−0.05
0.00
0.05
0.10
0.15
0.20
0.25
NMD

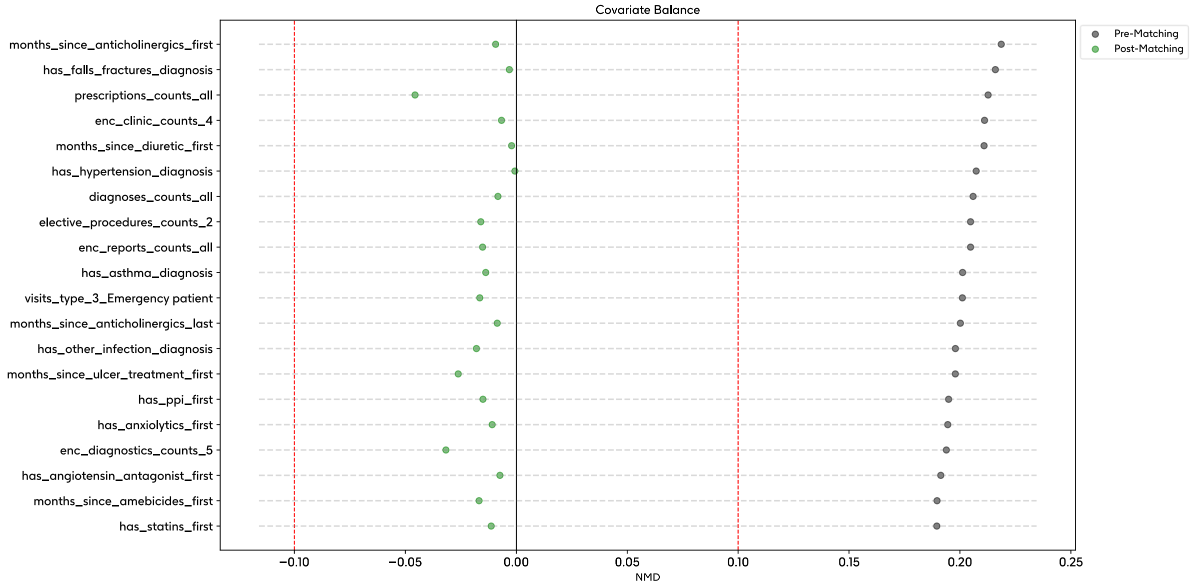
Covariate Balance
months_since_anticholinergics_first
has_falls_fractures_diagnosis
prescriptions_counts_all
enc_clinic_counts_4
months_since_diuretic_first
has_hypertension_diagnosis
diagnoses_counts_all
elective_procedures_counts_2
enc_reports_counts_all
has_asthma_diagnosis
visits_type_3_Emergency patient
months_since_anticholinergics_last
has_other_infection_diagnosis
months_since_ulcer_treatment_first
has_ppi_first
has_anxiolytics_first
enc_diagnostics_counts_5
has_angiotensin_antagonist_first
months_since_amebicides_first
has_statins_first
Pre-Matching
Post-Matching
−0.10
−0.05
0.00
0.05
0.10
0.15
0.20
0.25
NMD

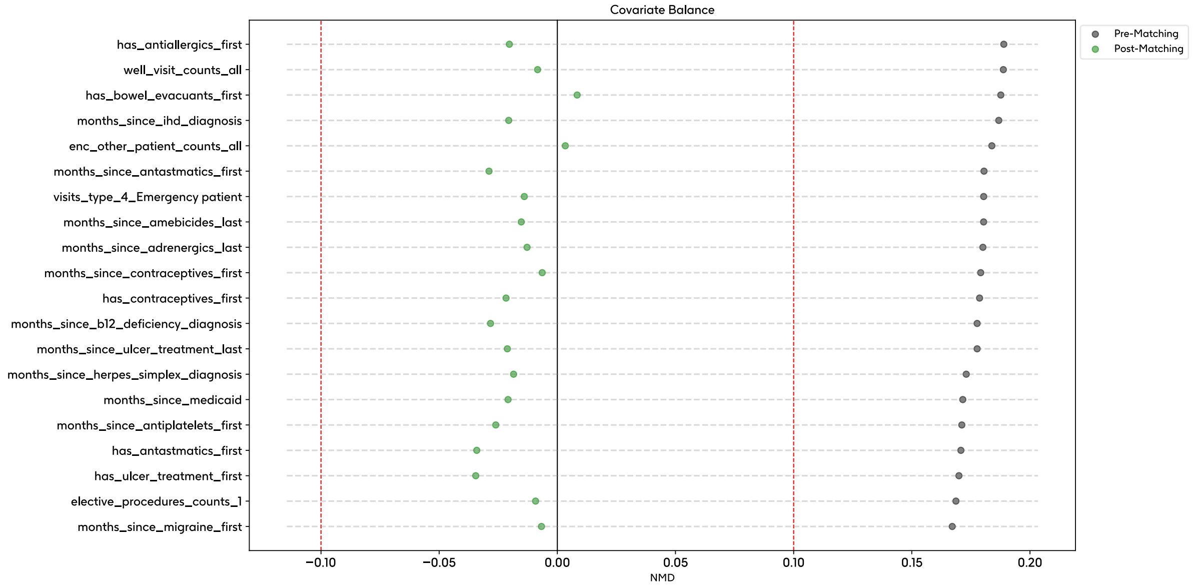
Covariate Balance
has_antiallergics_first
well_visit_counts_all
has_bowel_evacuants_first
months_since_ihd_diagnosis
enc_other_patient_counts_all
months_since_antastmatics_first
visits_type_4_Emergency patient
months_since_amebicides_last
months_since_adrenergics_last
months_since_contraceptives_first
has_contraceptives_first
months_since_b12_deficiency_diagnosis
months_since_ulcer_treatment_last
months_since_herpes_simplex_diagnosis
months_since_medicaid
months_since_antiplatelets_first
has_antastmatics_first
has_ulcer_treatment_first
elective_procedures_counts_1
months_since_migraine_first
Pre-Matching
Post-Matching
−0.10
−0.05
0.00
0.05
0.10
0.15
0.20
NMD

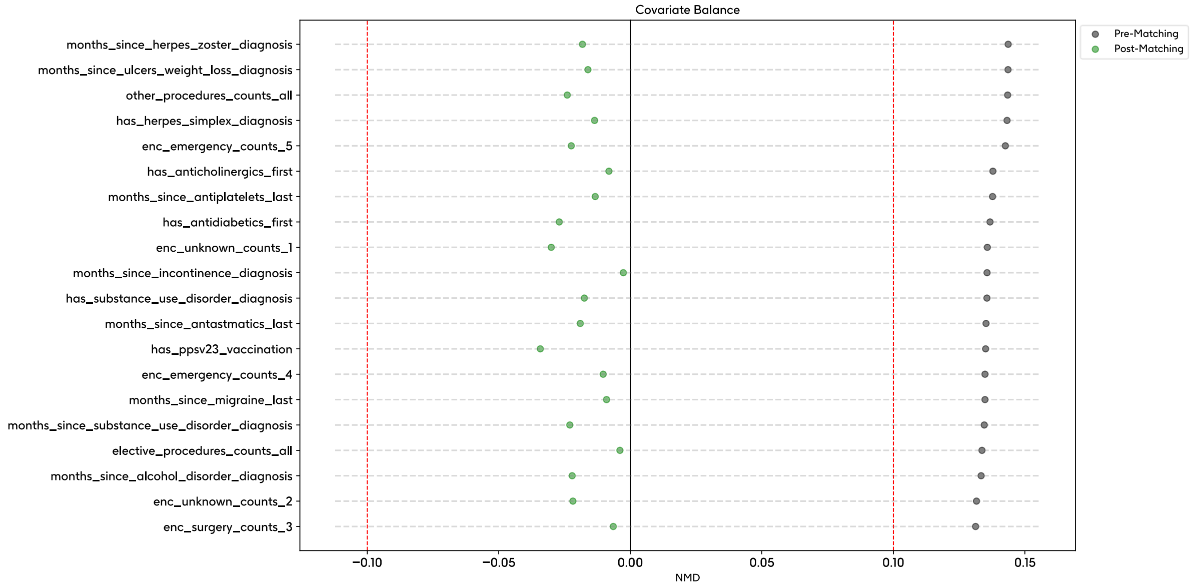
Covariate Balance
months_since_herpes_zoster_diagnosis
months_since_ulcers_weight_loss_diagnosis
other_procedures_counts_all
has_herpes_simplex_diagnosis
enc_emergency_counts_5
has_anticholinergics_first
months_since_antiplatelets_last
has_antidiabetics_first
enc_unknown_counts_1
months_since_incontinence_diagnosis
has_substance_use_disorder_diagnosis
months_since_antastmatics_last
has_ppsv23_vaccination
enc_emergency_counts_4
months_since_migraine_last
months_since_substance_use_disorder_diagnosis
elective_procedures_counts_all
months_since_alcohol_disorder_diagnosis
enc_unknown_counts_2
enc_surgery_counts_3
Pre-Matching
Post-Matching
−0.10
−0.05
0.00
0.05
0.10
0.15
NMD

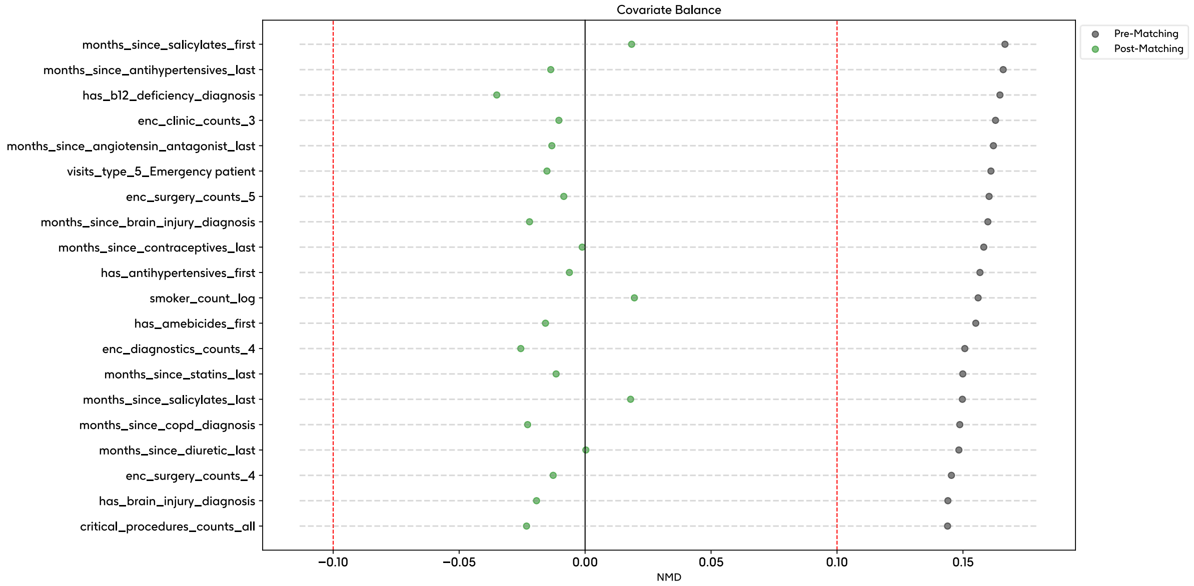
Covariate Balance
months_since_salicylates_first
months_since_antihypertensives_last
has_b12_deficiency_diagnosis
enc_clinic_counts_3
months_since_angiotensin_antagonist_last
visits_type_5_Emergency patient
enc_surgery_counts_5
months_since_brain_injury_diagnosis
months_since_contraceptives_last
has_antihypertensives_first
smoker_count_log
has_amebicides_first
enc_diagnostics_counts_4
months_since_statins_last
months_since_salicylates_last
months_since_copd_diagnosis
months_since_diuretic_last
enc_surgery_counts_4
has_brain_injury_diagnosis
critical_procedures_counts_all
Pre-Matching
Post-Matching
−0.10
−0.05
0.00
0.05
0.10
0.15
NMD

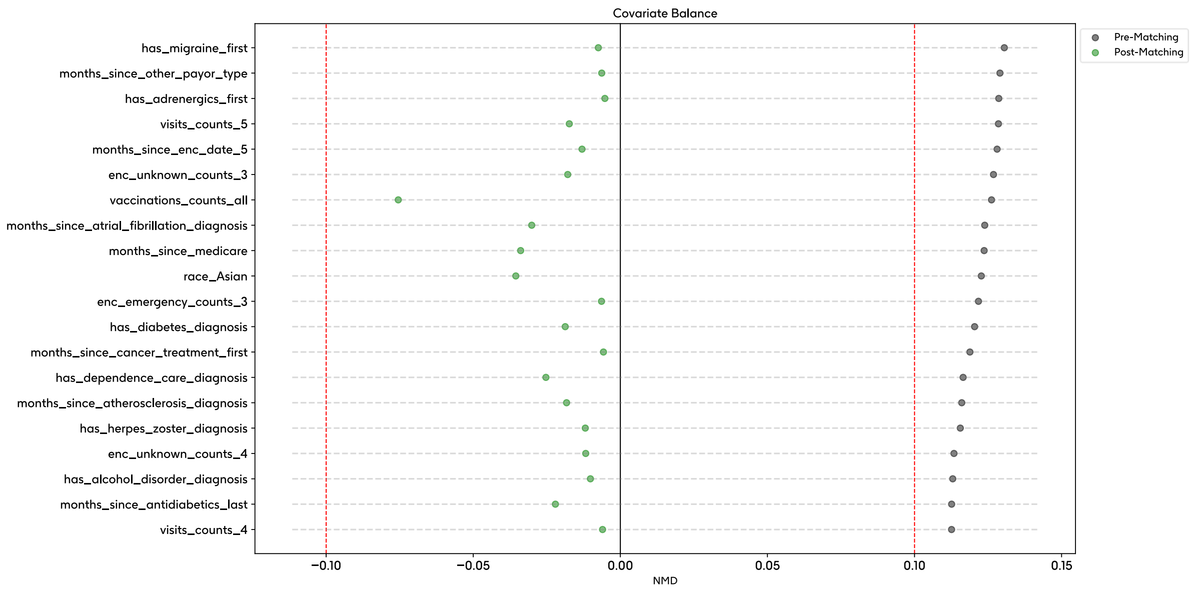
Covariate Balance
Pre-Matching
Post-Matching
has_migraine_first
months_since_other_payor_type
has_adrenergics_first
visits_counts_5
months_since_enc_date_5
enc_unknown_counts_3
vaccinations_counts_all
months_since_atrial_fibrillation_diagnosis
months_since_medicare
race_Asian
enc_emergency_counts_3
has_diabetes_diagnosis
months_since_cancer_treatment_first
has_dependence_care_diagnosis
months_since_atherosclerosis_diagnosis
has_herpes_zoster_diagnosis
enc_unknown_counts_4
has_alcohol_disorder_diagnosis
months_since_antidiabetics_last
visits_counts_4
−0.10
−0.05
0.00
0.05
0.10
0.15
NMD

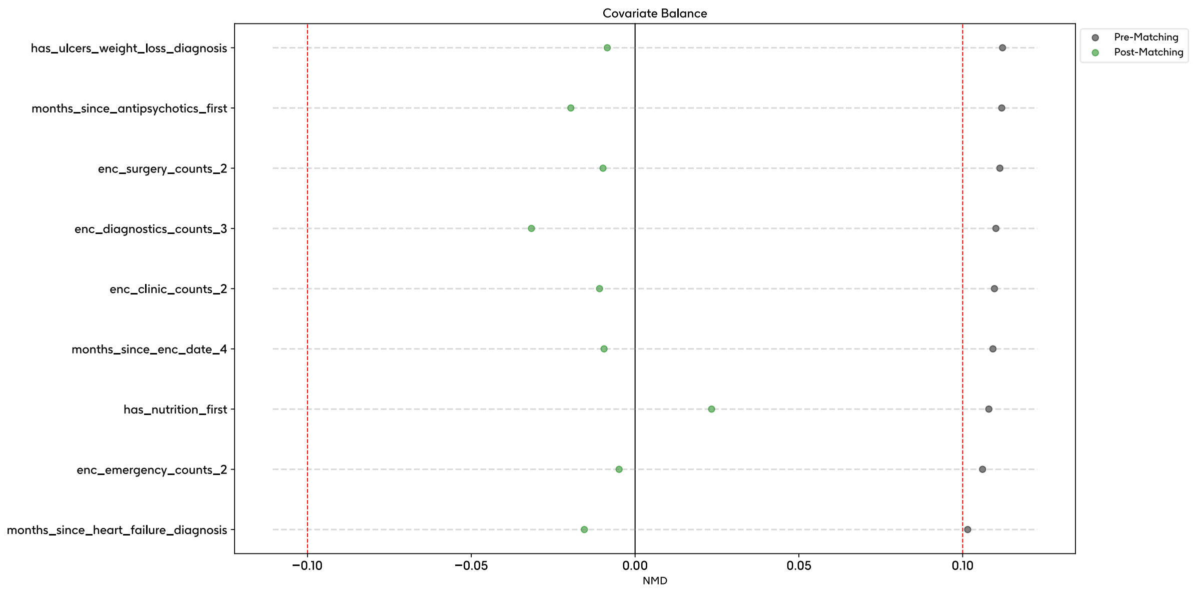
Covariate Balance
Pre-Matching
Post-Matching
has_ulcers_weight_loss_diagnosis
months_since_antipsychotics_first
enc_surgery_counts_2
enc_diagnostics_counts_3
enc_clinic_counts_2
months_since_enc_date_4
has_nutrition_first
enc_emergency_counts_2
months_since_heart_failure_diagnosis
−0.10
−0.05
0.00
0.05
0.10
NMD

(b) Dementia: RZV (2+ doses) vs RZV (1 dose)

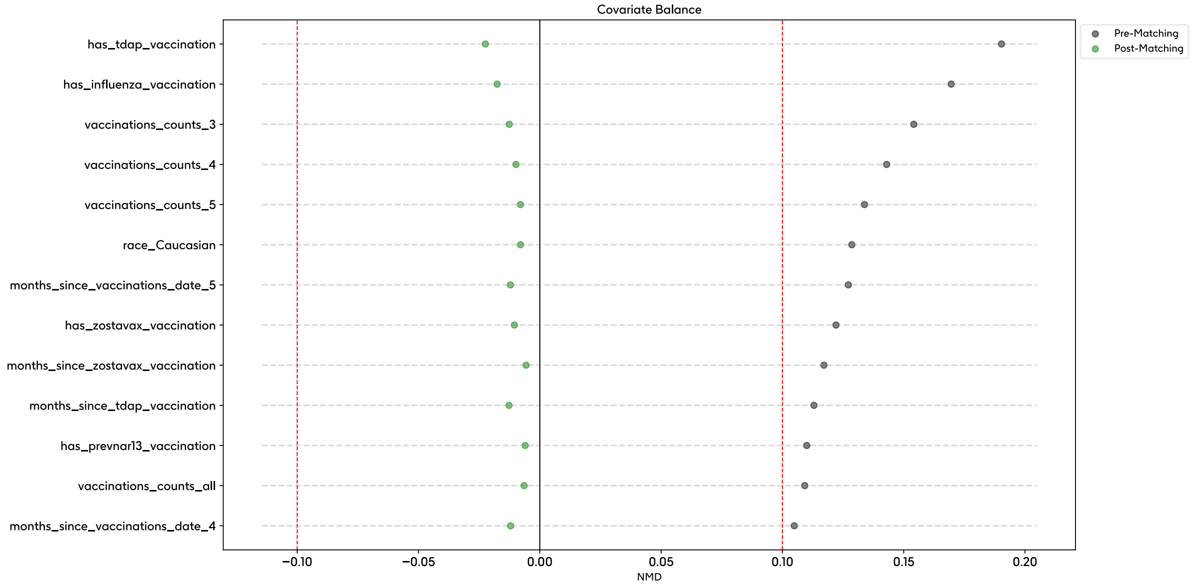

Supplementary Fig. S2 | Pre- and post-matching cohort balance for comparison between RZV (2+ doses) and RZV (1 dose) or ZVL cohorts.

The values on the y-axis are the names of covariates. The dots indicate pre- and post-matching normalized mean distance (NMD) for the corresponding covariate. The red vertical lines indicate the bounds of what is considered a good balance (-0.1 < NMD < 0.1). Post-matching dots within these bounds indicate that the corresponding covariate's mean is well balanced after matching. The covariates are sorted in descending order of their pre-matching NMD.

Note for interpretation of covariate names: For covariates named "months_since_i" (where i=1, 2, 3, 4, 5), the name indicates months between ith last intervention pre-exposure and the exposure date. For "months_since_vaccination", the term "vaccination" refers to any vaccination except the exposure vaccination). For "months_since_diagnosis" covariate, the term "diagnosis" refers to any diagnosis in the Diagnosis table with a certain status. For covariates named "intervention_counts_i", the name indicates the number of interventions in the ith pre-exposure year. The covariate named "well_visits_counts_all" indicates the total count of routine medical and gynecological examinations.

RZV (2+ doses) and RZV (1 dose), recipients of at least 2 doses or 1 dose of the recombinant zoster vaccine (*Shingrix*, GSK); ZVL, recipients of at least 1 dose of the live-attenuated zoster vaccine (*Zostavax*, Merck).

(a) Dementia: ZVL vs PPSV23 (women)

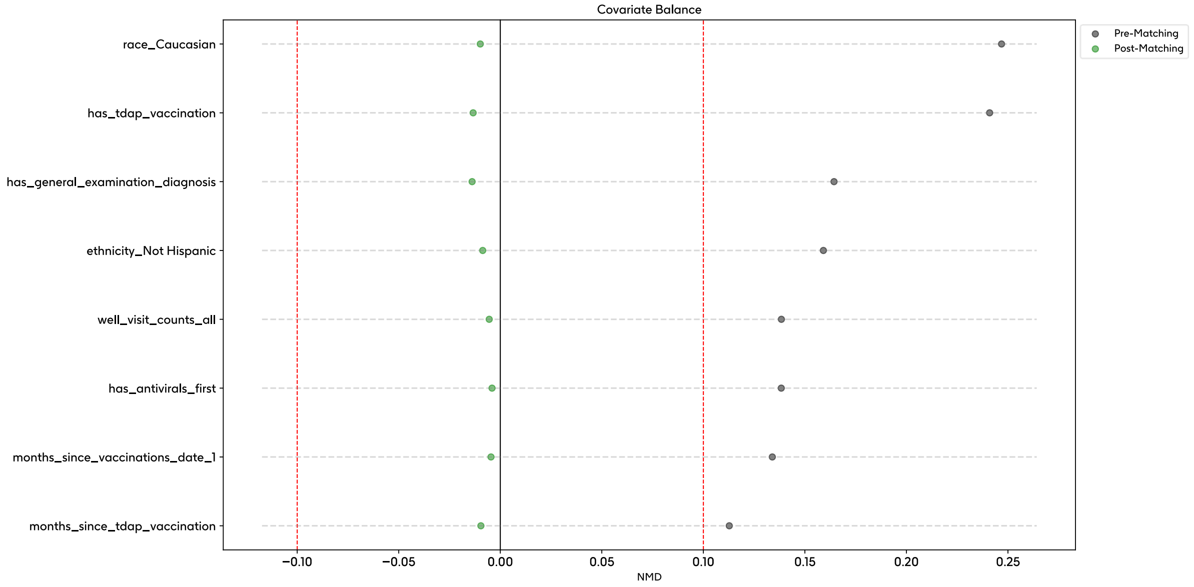

(b) Dementia: RZV (2+ doses) vs PPSV23 (women, 80–89 years)

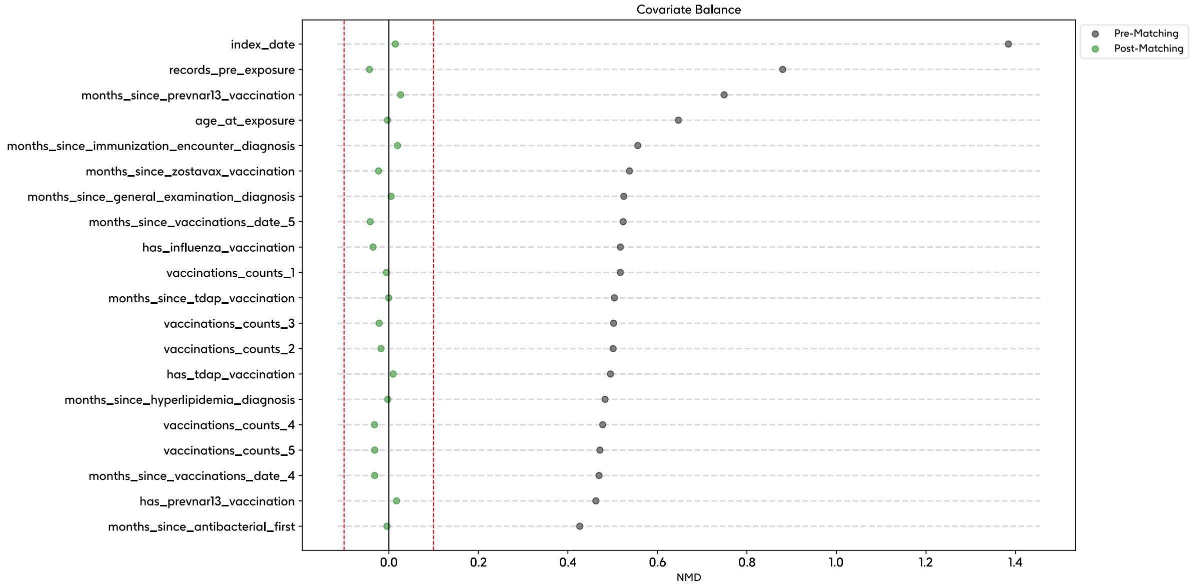

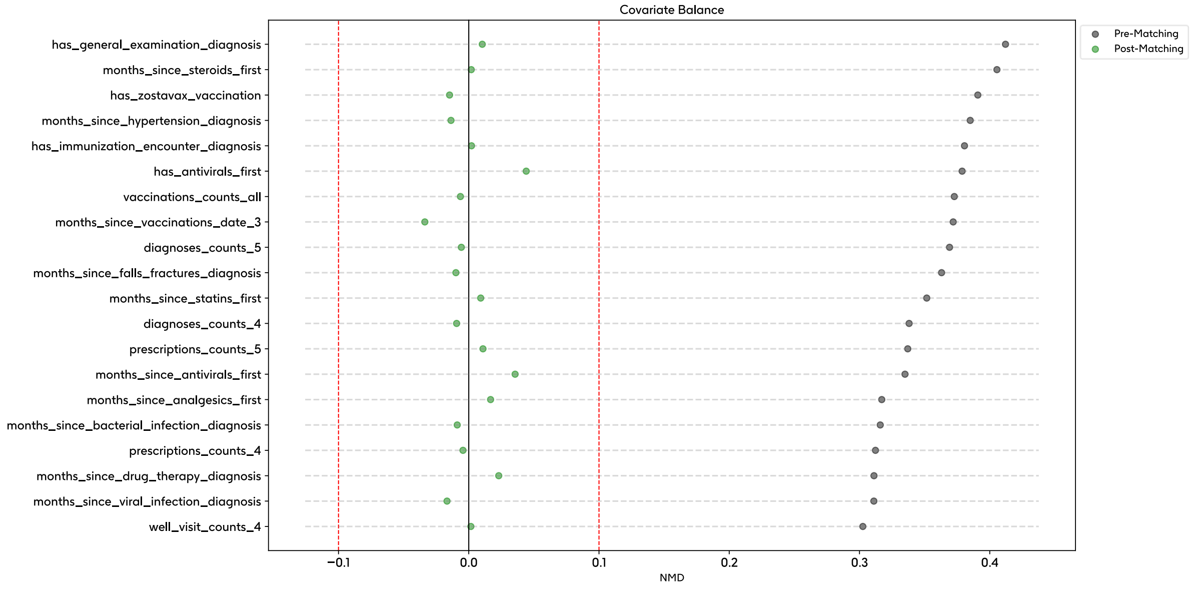
Covariate Balance
Pre-Matching
Post-Matching
has_general_examination_diagnosis
months_since_steroids_first
has_zostavax_vaccination
months_since_hypertension_diagnosis
has_immunization_encounter_diagnosis
has_antivirals_first
vaccinations_counts_all
months_since_vaccinations_date_3
diagnoses_counts_5
months_since_falls_fractures_diagnosis
months_since_statins_first
diagnoses_counts_4
prescriptions_counts_5
months_since_antivirals_first
months_since_analgesics_first
months_since_bacterial_infection_diagnosis
prescriptions_counts_4
months_since_drug_therapy_diagnosis
months_since_viral_infection_diagnosis
well_visit_counts_4
−0.1
0.0
0.1
0.2
0.3
0.4
NMD

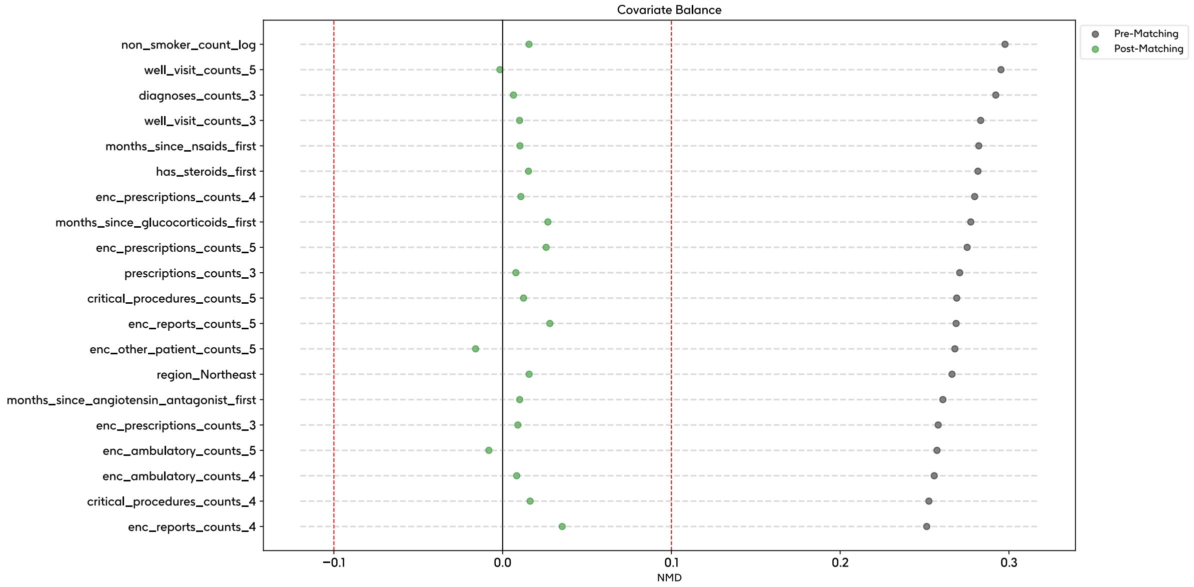
Covariate Balance
Pre-Matching
Post-Matching
non_smoker_count_log
well_visit_counts_5
diagnoses_counts_3
well_visit_counts_3
months_since_nsaids_first
has_steroids_first
enc_prescriptions_counts_4
months_since_glucocorticoids_first
enc_prescriptions_counts_5
prescriptions_counts_3
critical_procedures_counts_5
enc_reports_counts_5
enc_other_patient_counts_5
region_Northeast
months_since_angiotensin_antagonist_first
enc_prescriptions_counts_3
enc_ambulatory_counts_5
enc_ambulatory_counts_4
critical_procedures_counts_4
enc_reports_counts_4
−0.1
0.0
0.1
0.2
0.3
NMD

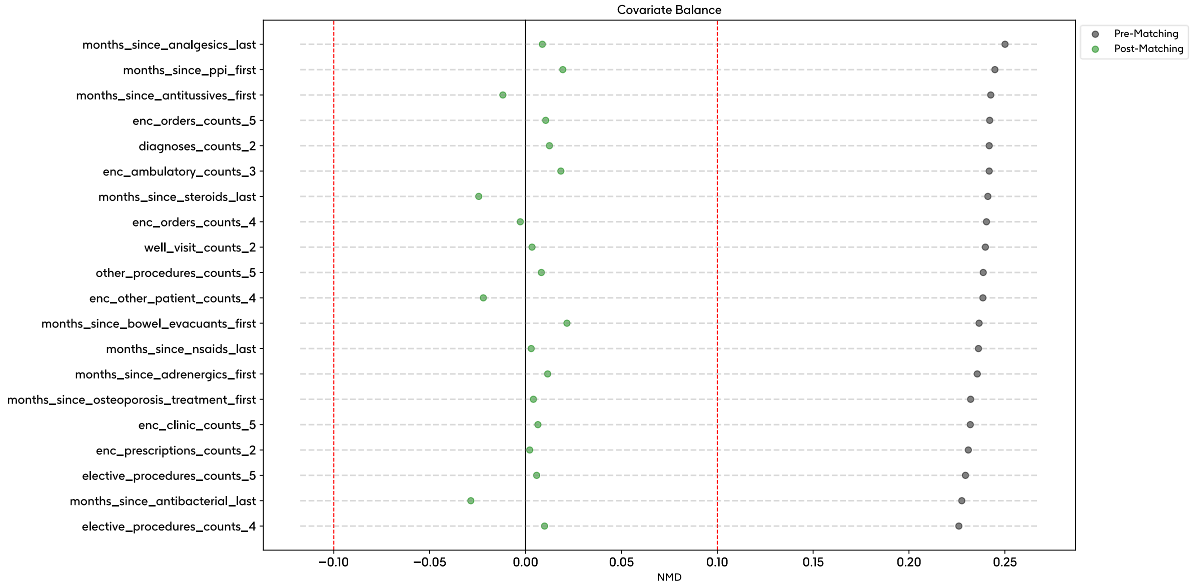
Covariate Balance
Pre-Matching
Post-Matching
months_since_analgesics_last
months_since_ppi_first
months_since_antitussives_first
enc_orders_counts_5
diagnoses_counts_2
enc_ambulatory_counts_3
months_since_steroids_last
enc_orders_counts_4
well_visit_counts_2
other_procedures_counts_5
enc_other_patient_counts_4
months_since_bowel_evacuants_first
months_since_nsaids_last
months_since_adrenergics_first
months_since_osteoporosis_treatment_first
enc_clinic_counts_5
enc_prescriptions_counts_2
elective_procedures_counts_5
months_since_antibacterial_last
elective_procedures_counts_4
−0.10
−0.05
0.00
0.05
0.10
0.15
0.20
0.25
NMD

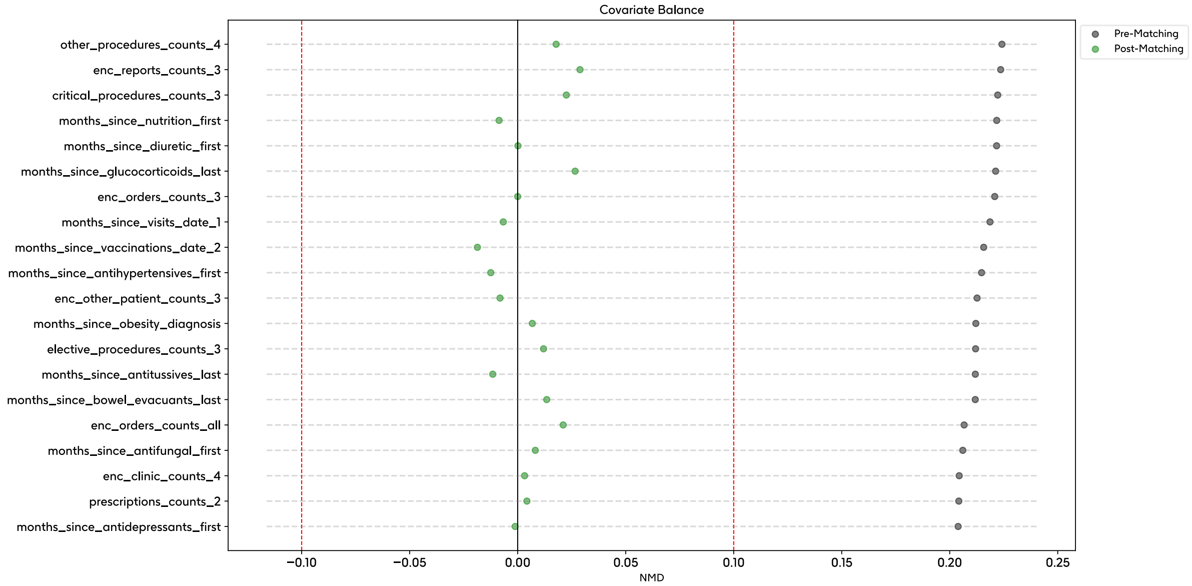
Covariate Balance
other_procedures_counts_4
enc_reports_counts_3
critical_procedures_counts_3
months_since_nutrition_first
months_since_diuretic_first
months_since_glucocorticoids_last
enc_orders_counts_3
months_since_visits_date_1
months_since_vaccinations_date_2
months_since_antihypertensives_first
enc_other_patient_counts_3
months_since_obesity_diagnosis
elective_procedures_counts_3
months_since_antitussives_last
months_since_bowel_evacuants_last
enc_orders_counts_all
months_since_antifungal_first
enc_clinic_counts_4
prescriptions_counts_2
months_since_antidepressants_first
Pre-Matching
Post-Matching
−0.10
−0.05
0.00
0.05
0.10
0.15
0.20
0.25
NMD

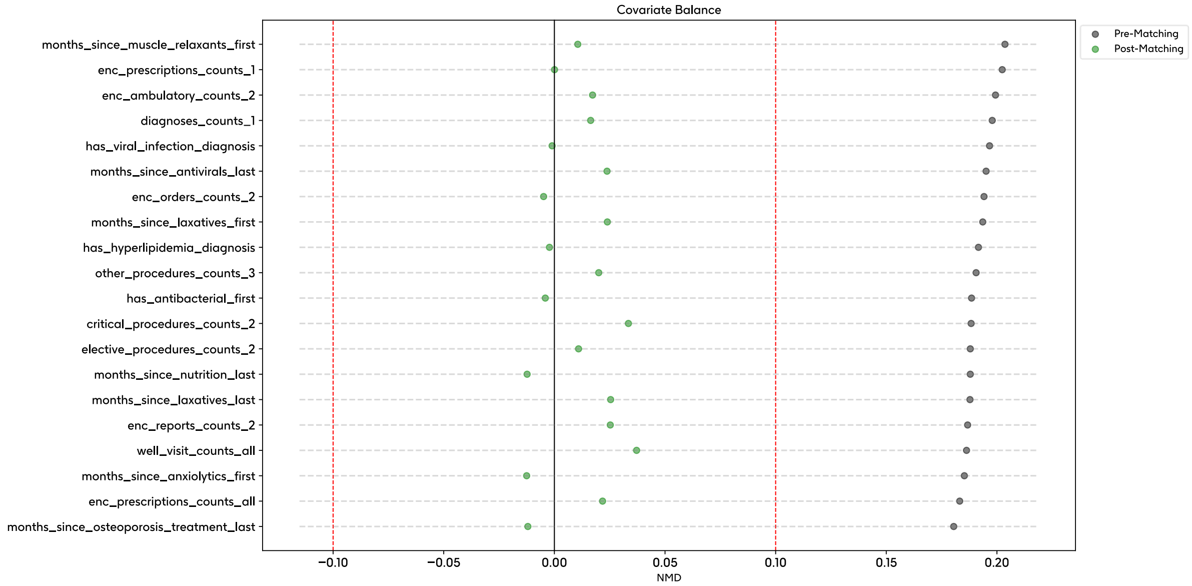
Covariate Balance
months_since_muscle_relaxants_first
enc_prescriptions_counts_1
enc_ambulatory_counts_2
diagnoses_counts_1
has_viral_infection_diagnosis
months_since_antivirals_last
enc_orders_counts_2
months_since_laxatives_first
has_hyperlipidemia_diagnosis
other_procedures_counts_3
has_antibacterial_first
critical_procedures_counts_2
elective_procedures_counts_2
months_since_nutrition_last
months_since_laxatives_last
enc_reports_counts_2
well_visit_counts_all
months_since_anxiolytics_first
enc_prescriptions_counts_all
months_since_osteoporosis_treatment_last
Pre-Matching
Post-Matching
−0.10
−0.05
0.00
0.05
0.10
0.15
0.20
NMD

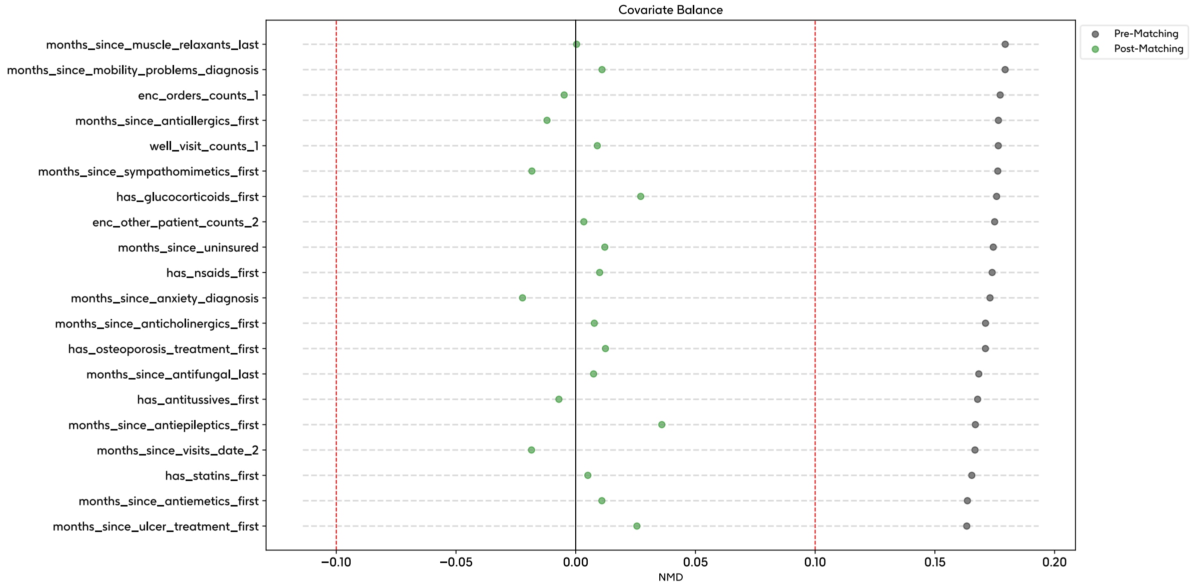
Covariate Balance
months_since_muscle_relaxants_last
months_since_mobility_problems_diagnosis
enc_orders_counts_1
months_since_antiallergics_first
well_visit_counts_1
months_since_sympathomimetics_first
has_glucocorticoids_first
enc_other_patient_counts_2
months_since_uninsured
has_nsaids_first
months_since_anxiety_diagnosis
months_since_anticholinergics_first
has_osteoporosis_treatment_first
months_since_antifungal_last
has_antitussives_first
months_since_antiepileptics_first
months_since_visits_date_2
has_statins_first
months_since_antiemetics_first
months_since_ulcer_treatment_first
Pre-Matching
Post-Matching
−0.10
−0.05
0.00
0.05
0.10
0.15
0.20
NMD

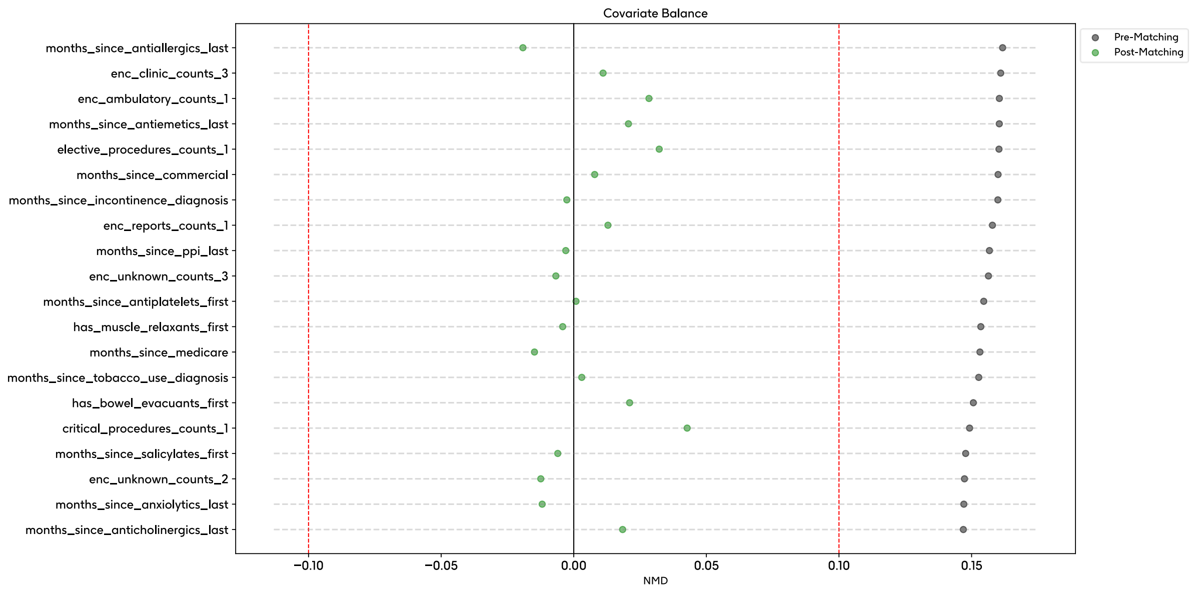
Covariate Balance
Pre-Matching
Post-Matching
months_since_antiallergics_last
enc_clinic_counts_3
enc_ambulatory_counts_1
months_since_antiemetics_last
elective_procedures_counts_1
months_since_commercial
months_since_incontinence_diagnosis
enc_reports_counts_1
months_since_ppi_last
enc_unknown_counts_3
months_since_antiplatelets_first
has_muscle_relaxants_first
months_since_medicare
months_since_tobacco_use_diagnosis
has_bowel_evacuants_first
critical_procedures_counts_1
months_since_salicylates_first
enc_unknown_counts_2
months_since_anxiolytics_last
months_since_anticholinergics_last
−0.10
−0.05
0.00
0.05
0.10
0.15
NMD

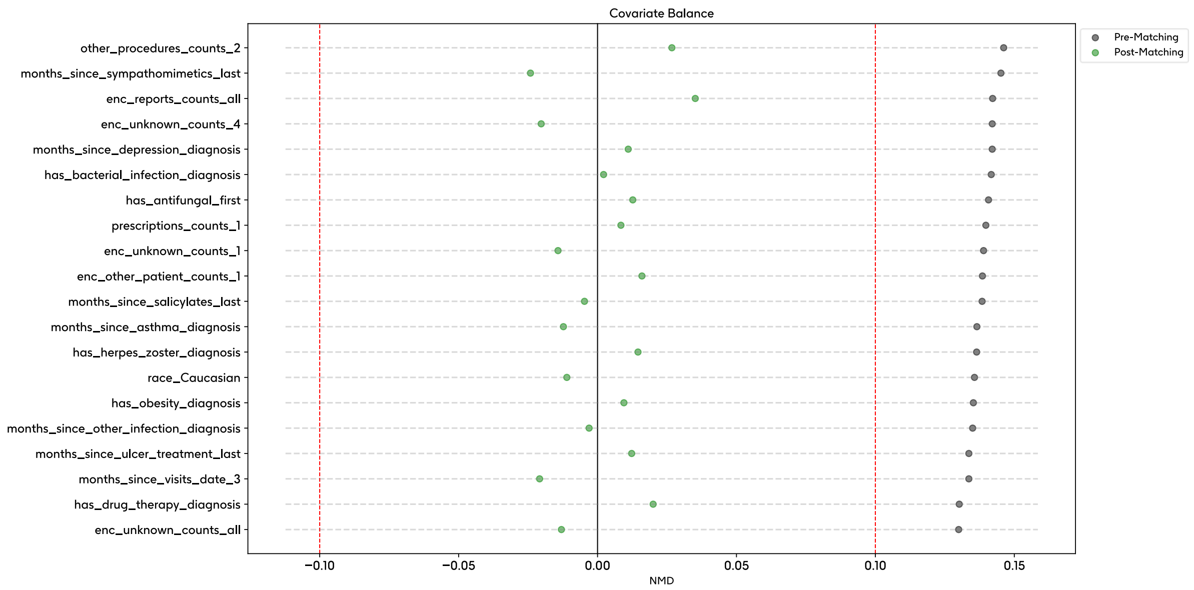
Covariate Balance
Pre-Matching
Post-Matching
other_procedures_counts_2
months_since_sympathomimetics_last
enc_reports_counts_all
enc_unknown_counts_4
months_since_depression_diagnosis
has_bacterial_infection_diagnosis
has_antifungal_first
prescriptions_counts_1
enc_unknown_counts_1
enc_other_patient_counts_1
months_since_salicylates_last
months_since_asthma_diagnosis
has_herpes_zoster_diagnosis
race_Caucasian
has_obesity_diagnosis
months_since_other_infection_diagnosis
months_since_ulcer_treatment_last
months_since_visits_date_3
has_drug_therapy_diagnosis
enc_unknown_counts_all
−0.10
−0.05
0.00
0.05
0.10
0.15
NMD

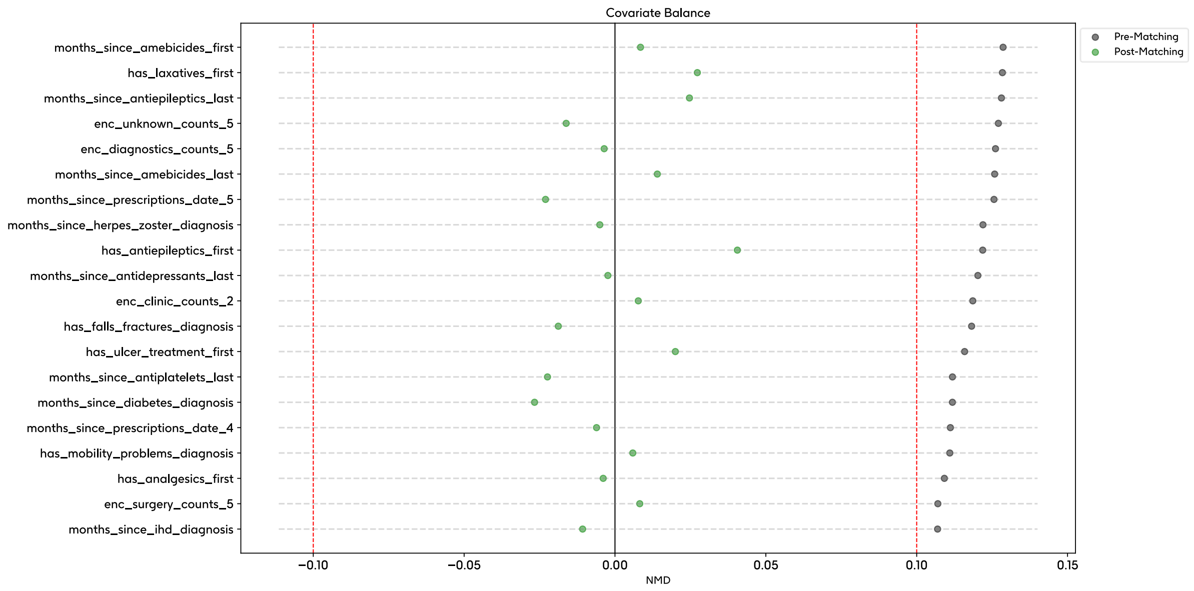
Covariate Balance
Pre-Matching
Post-Matching
months_since_amebicides_first
has_laxatives_first
months_since_antiepileptics_last
enc_unknown_counts_5
enc_diagnostics_counts_5
months_since_amebicides_last
months_since_prescriptions_date_5
months_since_herpes_zoster_diagnosis
has_antiepileptics_first
months_since_antidepressants_last
enc_clinic_counts_2
has_falls_fractures_diagnosis
has_ulcer_treatment_first
months_since_antiplatelets_last
months_since_diabetes_diagnosis
months_since_prescriptions_date_4
has_mobility_problems_diagnosis
has_analgesics_first
enc_surgery_counts_5
months_since_ihd_diagnosis
−0.10
−0.05
0.00
0.05
0.10
0.15
NMD

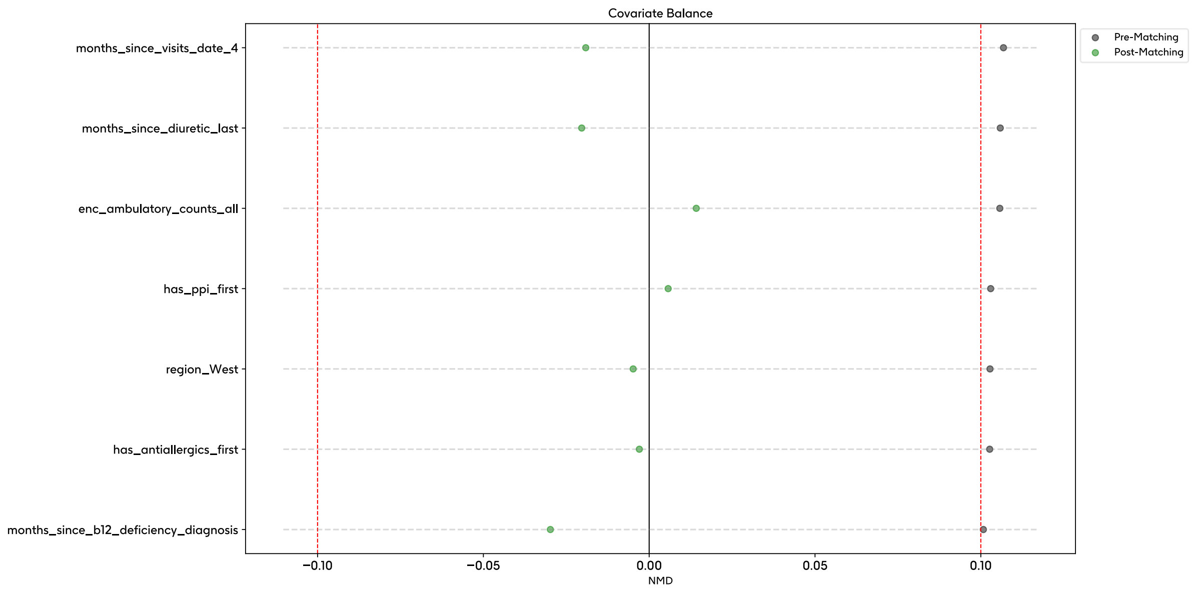

Supplementary Fig. S3 | Pre- and post-matching cohort balance for comparisons in subpopulations of women.

The values on the y-axis are the names of covariates. The dots indicate pre- and post-matching normalized mean distance (NMD) for the corresponding covariate. The red vertical lines indicate the bounds of what is considered a good balance (-0.1 < NMD < 0.1). Post-matching dots within these bounds indicate that the corresponding covariate's mean is well balanced after matching. The covariates are sorted in descending order of their pre-matching NMD.

Note for interpretation of covariate names: For covariates named "months_since_i" (where i=1, 2, 3, 4, 5), the name indicates months between ith last intervention pre-exposure and the exposure date. For "months_since_vaccination", the term "vaccination" refers to any vaccination except the exposure vaccination). For "months_since_diagnosis" covariate, the term "diagnosis" refers to any diagnosis in the Diagnosis table with a certain status. For covariates named "intervention_counts_i", the name indicates the number of interventions in the ith pre-exposure year. The covariate named "well_visits_counts_all" indicates the total count of routine medical and gynecological examinations.

PPSV23, recipients of at least 1 dose of a 23-valent pneumococcal polysaccharide vaccine; RZV (2+ doses), recipients of at least 2 doses of the recombinant zoster vaccine (*Shingrix*, GSK); ZVL, recipients of at least 1 dose of the live-attenuated zoster vaccine (*Zostavax*, Merck).

(a) Dementia: ZVL vs Not exposed (all)

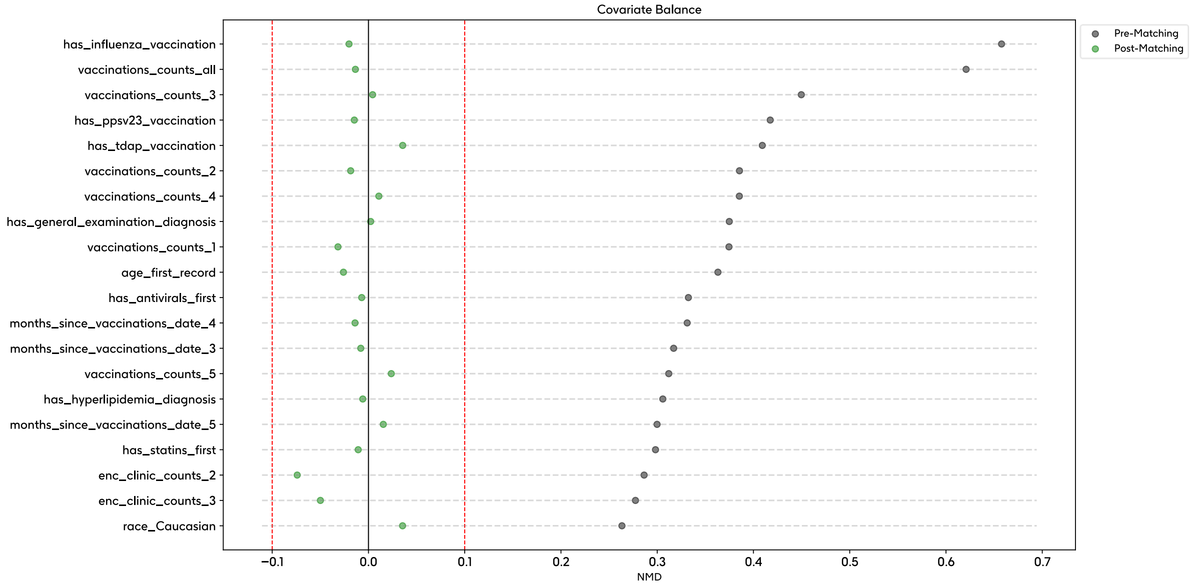

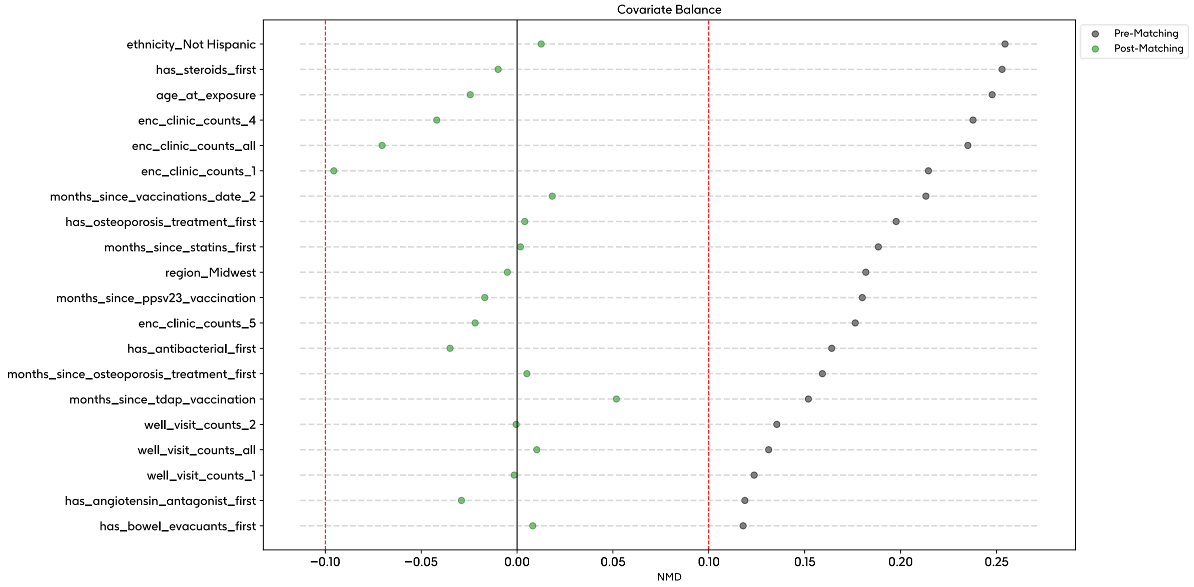

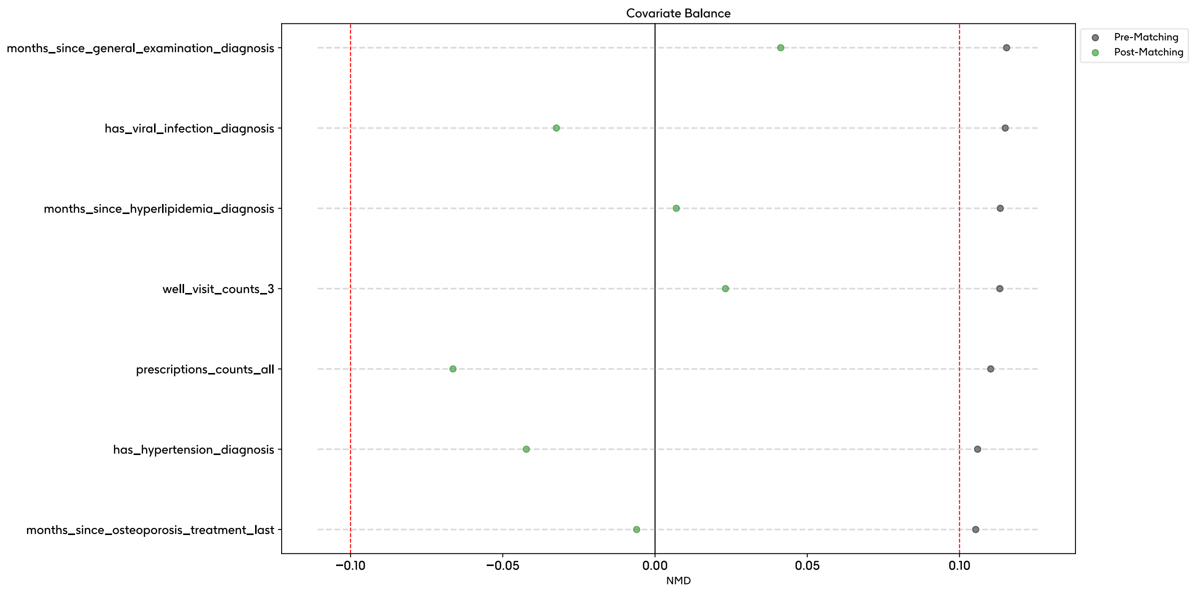

(b) Dementia: RZV (2+ doses) vs Not exposed (all)

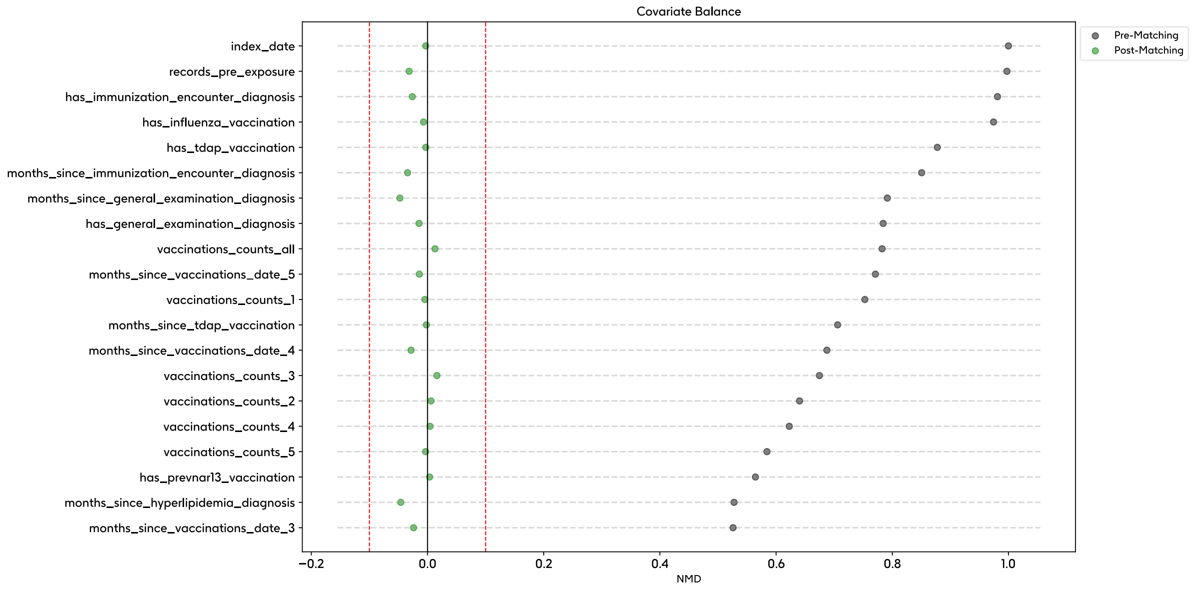

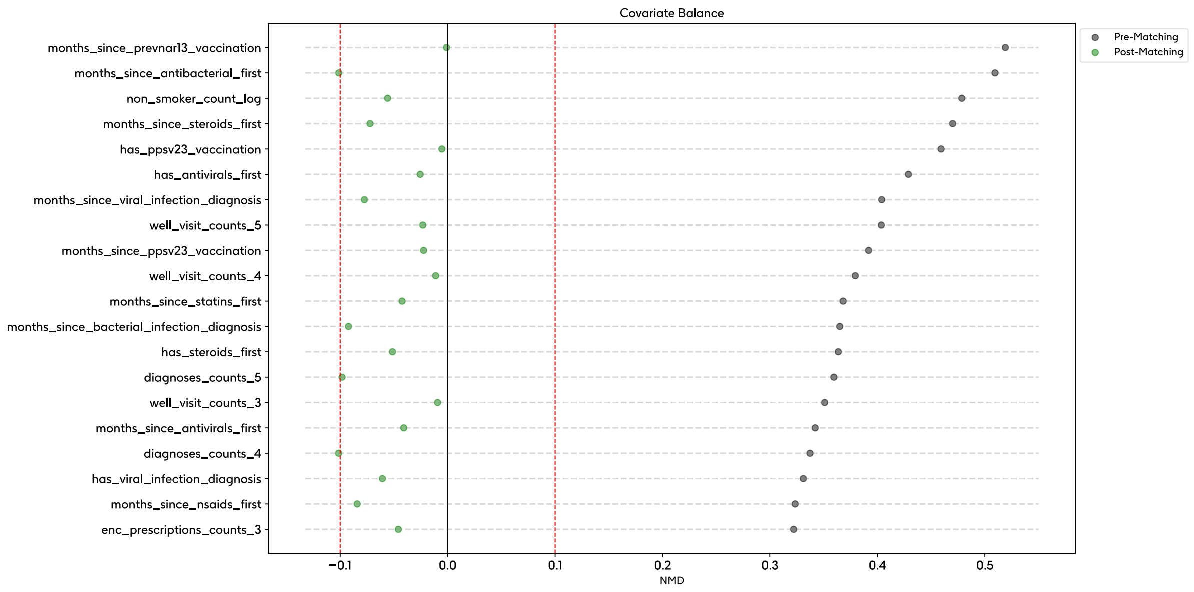
Covariate Balance
Pre-Matching
Post-Matching
months_since_prevnar13_vaccination
months_since_antibacterial_first
non_smoker_count_log
months_since_steroids_first
has_ppsv23_vaccination
has_antivirals_first
months_since_viral_infection_diagnosis
well_visit_counts_5
months_since_ppsv23_vaccination
well_visit_counts_4
months_since_statins_first
months_since_bacterial_infection_diagnosis
has_steroids_first
diagnoses_counts_5
well_visit_counts_3
months_since_antivirals_first
diagnoses_counts_4
has_viral_infection_diagnosis
months_since_nsaids_first
enc_prescriptions_counts_3
−0.1
0.0
0.1
0.2
0.3
0.4
0.5
NMD

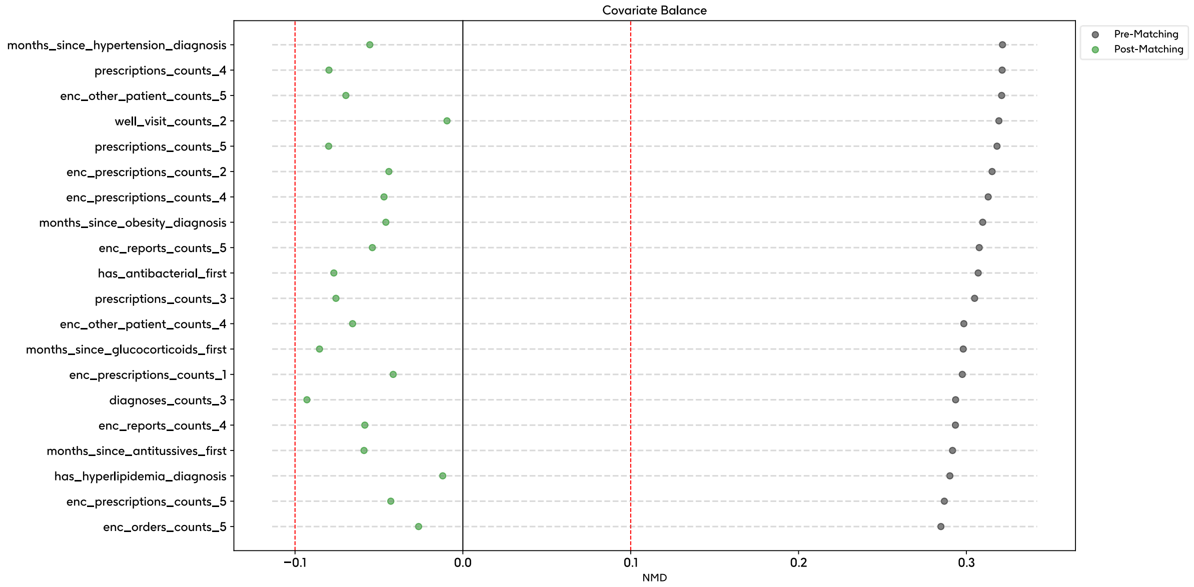
Covariate Balance
Pre-Matching
Post-Matching
months_since_hypertension_diagnosis
prescriptions_counts_4
enc_other_patient_counts_5
well_visit_counts_2
prescriptions_counts_5
enc_prescriptions_counts_2
enc_prescriptions_counts_4
months_since_obesity_diagnosis
enc_reports_counts_5
has_antibacterial_first
prescriptions_counts_3
enc_other_patient_counts_4
months_since_glucocorticoids_first
enc_prescriptions_counts_1
diagnoses_counts_3
enc_reports_counts_4
months_since_antitussives_first
has_hyperlipidemia_diagnosis
enc_prescriptions_counts_5
enc_orders_counts_5
−0.1
0.0
0.1
0.2
0.3
NMD

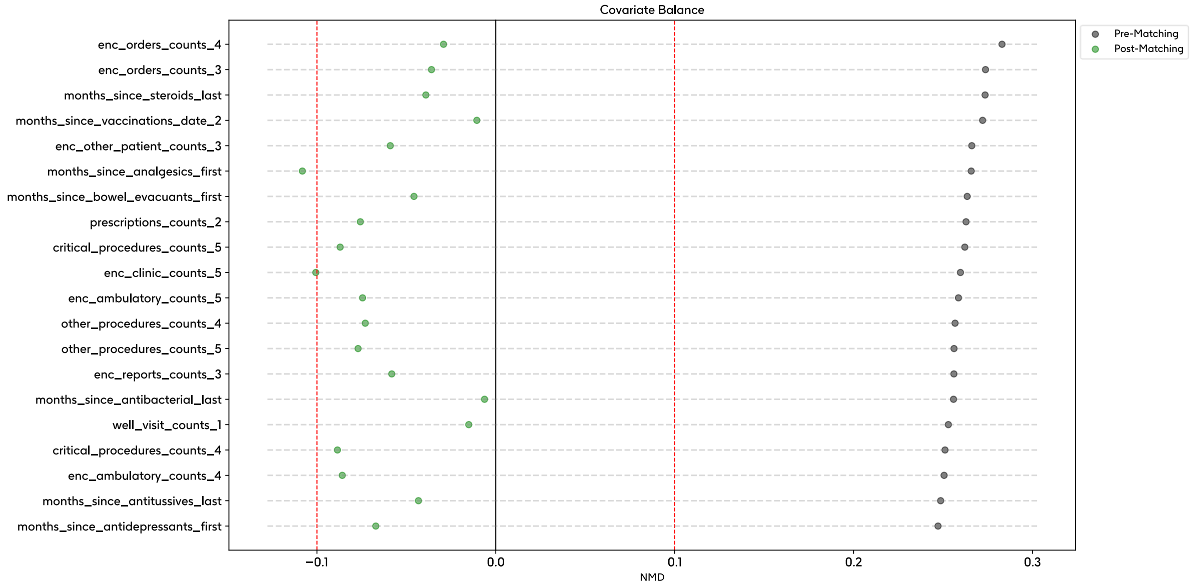
Covariate Balance
Pre-Matching
Post-Matching
enc_orders_counts_4
enc_orders_counts_3
months_since_steroids_last
months_since_vaccinations_date_2
enc_other_patient_counts_3
months_since_analgesics_first
months_since_bowel_evacuants_first
prescriptions_counts_2
critical_procedures_counts_5
enc_clinic_counts_5
enc_ambulatory_counts_5
other_procedures_counts_4
other_procedures_counts_5
enc_reports_counts_3
months_since_antibacterial_last
well_visit_counts_1
critical_procedures_counts_4
enc_ambulatory_counts_4
months_since_antitussives_last
months_since_antidepressants_first
−0.1
0.0
0.1
0.2
0.3
NMD

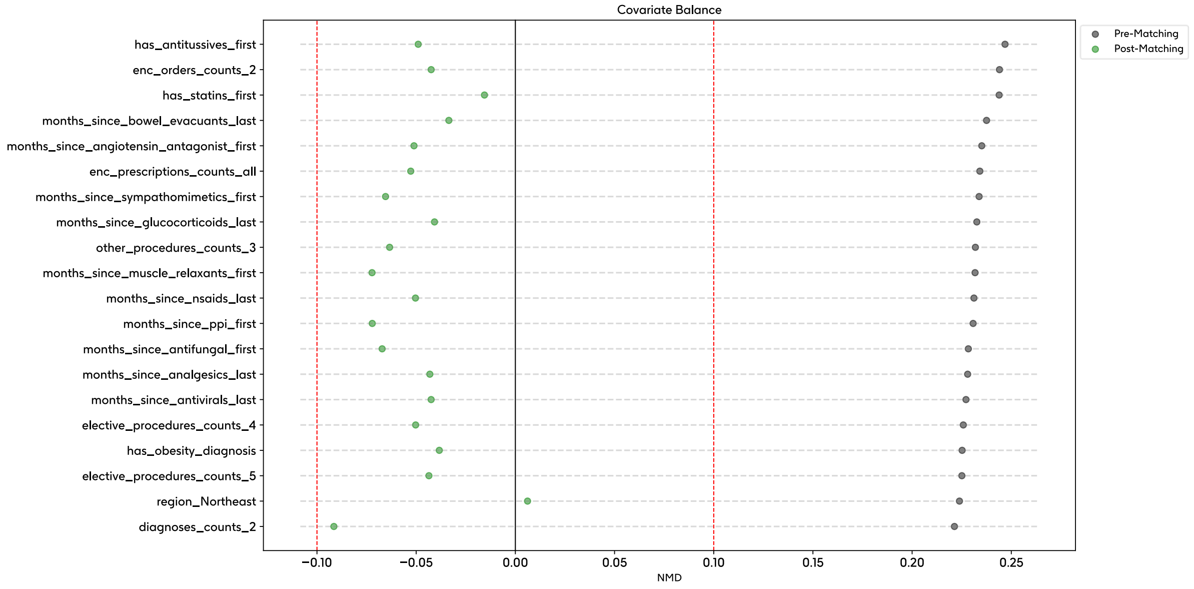
Covariate Balance
has_antitussives_first
enc_orders_counts_2
has_statins_first
months_since_bowel_evacuants_last
months_since_angiotensin_antagonist_first
enc_prescriptions_counts_all
months_since_sympathomimetics_first
months_since_glucocorticoids_last
other_procedures_counts_3
months_since_muscle_relaxants_first
months_since_nsaids_last
months_since_ppi_first
months_since_antifungal_first
months_since_analgesics_last
months_since_antivirals_last
elective_procedures_counts_4
has_obesity_diagnosis
elective_procedures_counts_5
region_Northeast
diagnoses_counts_2
−0.10
−0.05
0.00
0.05
0.10
0.15
0.20
0.25
NMD
Pre-Matching
Post-Matching

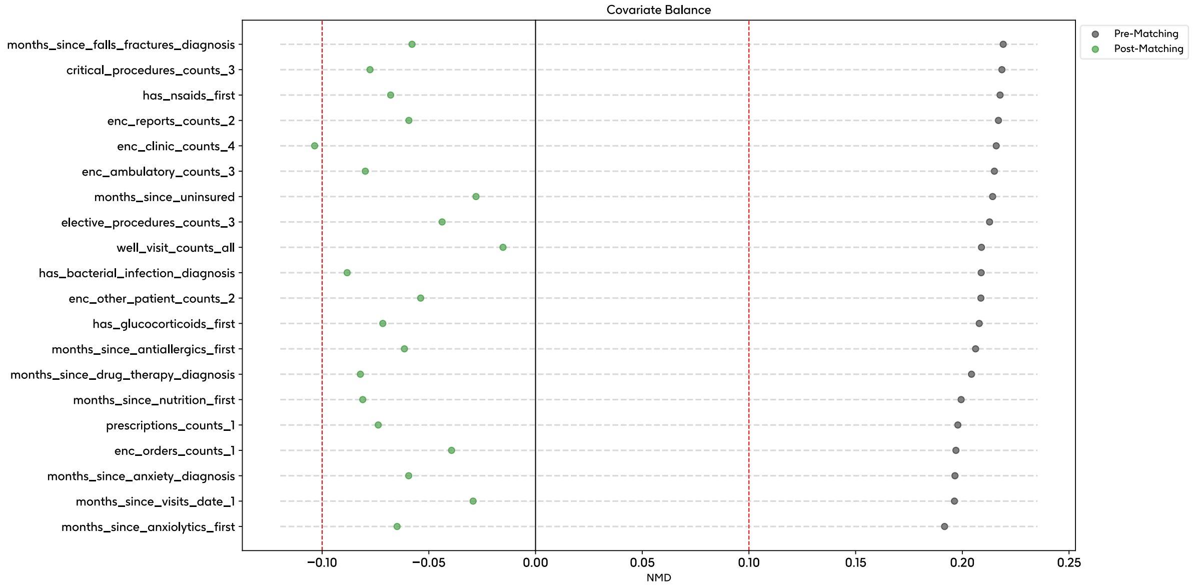
Covariate Balance
months_since_falls_fractures_diagnosis
critical_procedures_counts_3
has_nsaids_first
enc_reports_counts_2
enc_clinic_counts_4
enc_ambulatory_counts_3
months_since_uninsured
elective_procedures_counts_3
well_visit_counts_all
has_bacterial_infection_diagnosis
enc_other_patient_counts_2
has_glucocorticoids_first
months_since_antiallergics_first
months_since_drug_therapy_diagnosis
months_since_nutrition_first
prescriptions_counts_1
enc_orders_counts_1
months_since_anxiety_diagnosis
months_since_visits_date_1
months_since_anxiolytics_first
−0.10
−0.05
0.00
0.05
0.10
0.15
0.20
0.25
NMD
Pre-Matching
Post-Matching

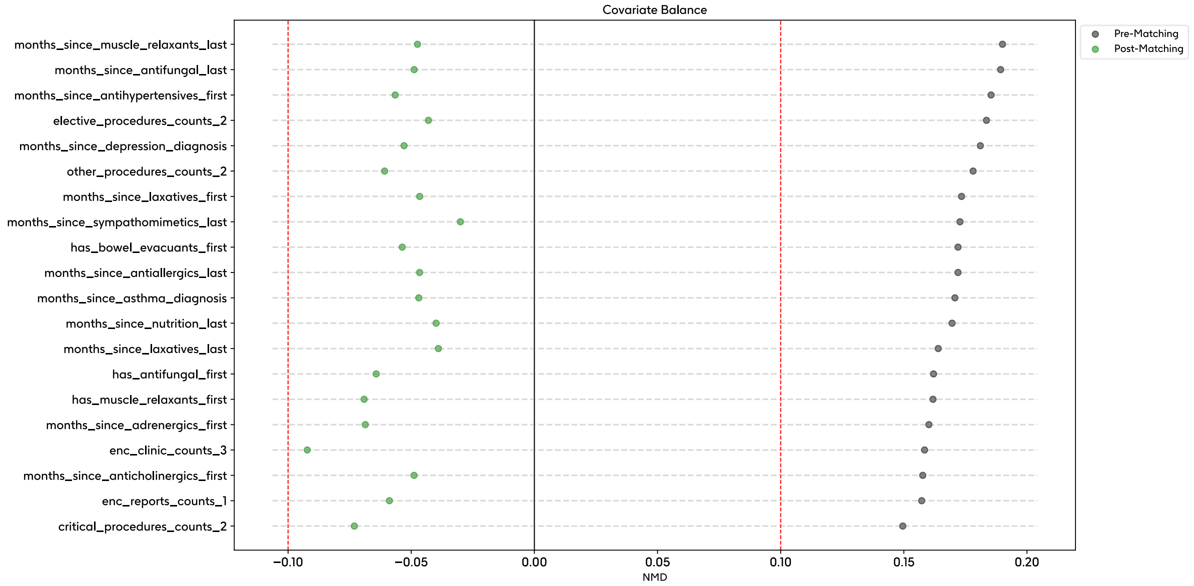
Covariate Balance
months_since_muscle_relaxants_last
months_since_antifungal_last
months_since_antihypertensives_first
elective_procedures_counts_2
months_since_depression_diagnosis
other_procedures_counts_2
months_since_laxatives_first
months_since_sympathomimetics_last
has_bowel_evacuants_first
months_since_antiallergics_last
months_since_asthma_diagnosis
months_since_nutrition_last
months_since_laxatives_last
has_antifungal_first
has_muscle_relaxants_first
months_since_adrenergics_first
enc_clinic_counts_3
months_since_anticholinergics_first
enc_reports_counts_1
critical_procedures_counts_2
−0.10
−0.05
0.00
0.05
0.10
0.15
0.20
NMD
Pre-Matching
Post-Matching

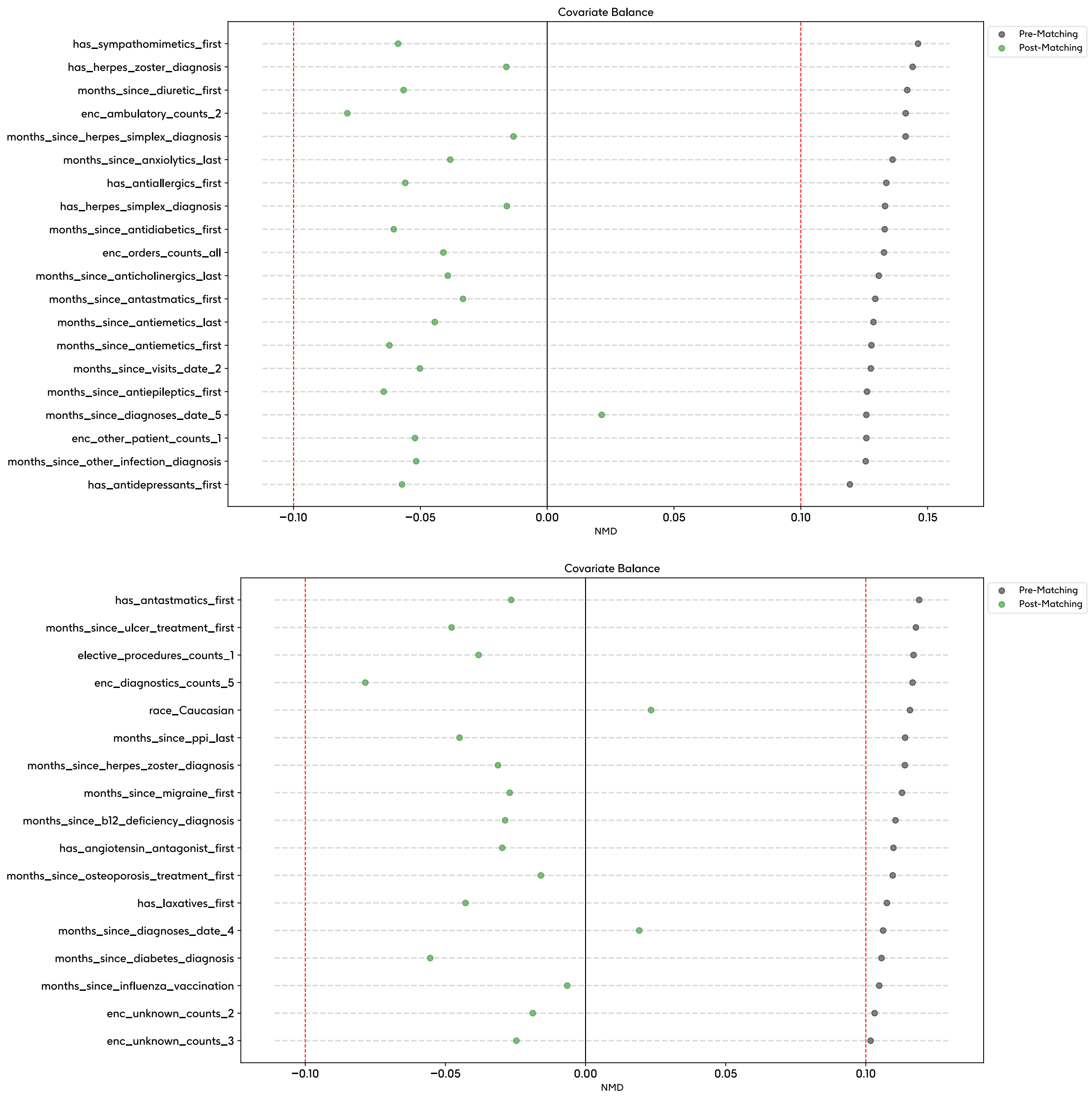

Supplementary Fig. S4 | Pre- and post-matching cohort balance for comparisons with the Note exposed cohorts.

The values on the y-axis are the names of covariates. The dots indicate pre- and post-matching normalized mean distance (NMD) for the corresponding covariate. The red vertical lines indicate the bounds of what is considered a good balance (-0.1 < NMD < 0.1). Post-matching dots within these bounds indicate that the corresponding covariate's mean is well balanced after matching. The covariates are sorted in descending order of their pre-matching NMD.

Note for interpretation of covariate names: For covariates named "months_since_i" (where i=1, 2, 3, 4, 5), the name indicates months between ith last intervention pre-exposure and the exposure date. For "months_since_vaccination", the term "vaccination" refers to any vaccination except the exposure vaccination). For "months_since_diagnosis" covariate, the term "diagnosis" refers to any diagnosis in the Diagnosis table with a certain status. For covariates named "intervention_counts_i", the name indicates the number of interventions in the ith pre-exposure year. The covariate named "well_visits_counts_all" indicates the total count of routine medical and gynecological examinations.

Not exposed, individuals who had not received a shingles vaccine; RZV (2+ doses), recipients of at least 2 doses of the recombinant zoster vaccine (*Shingrix*, GSK); ZVL, recipients of at least 1 dose of the live-attenuated zoster vaccine (*Zostavax*, Merck).

(a) Dementia (code AND medication): ZVL vs PPSV23 (all)

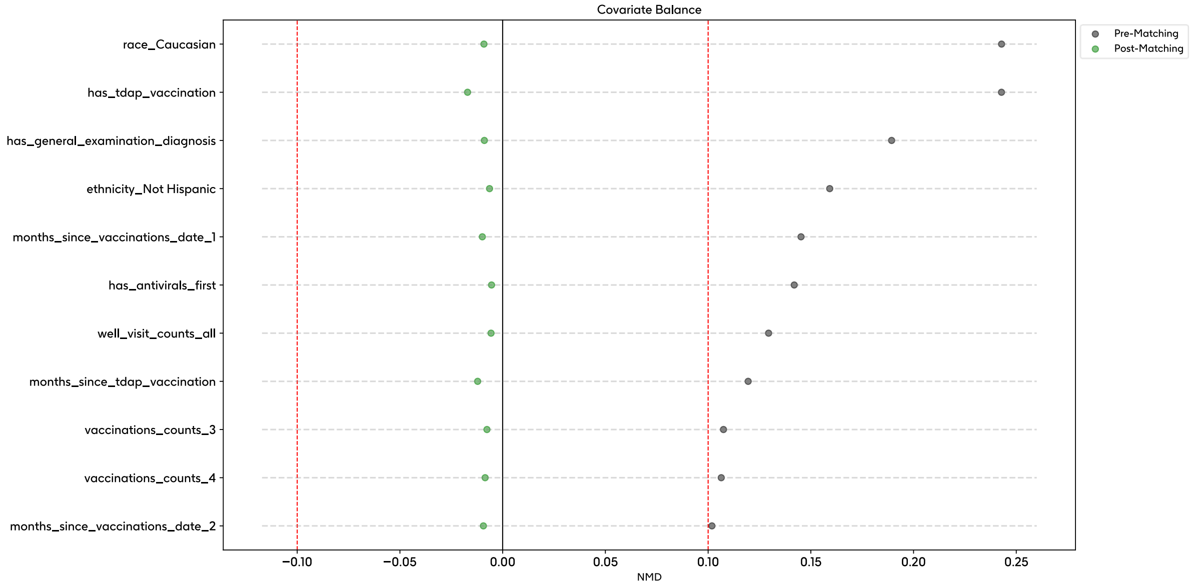

(b) Dementia (code AND medication): RZV (2+ doses) vs PPSV23 (all)

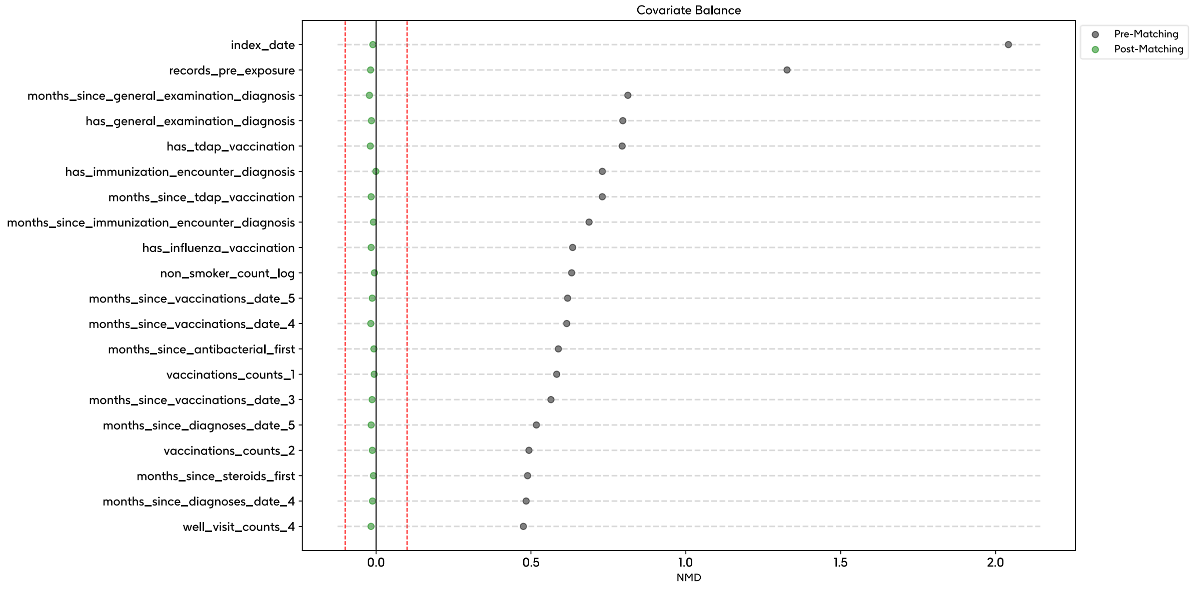

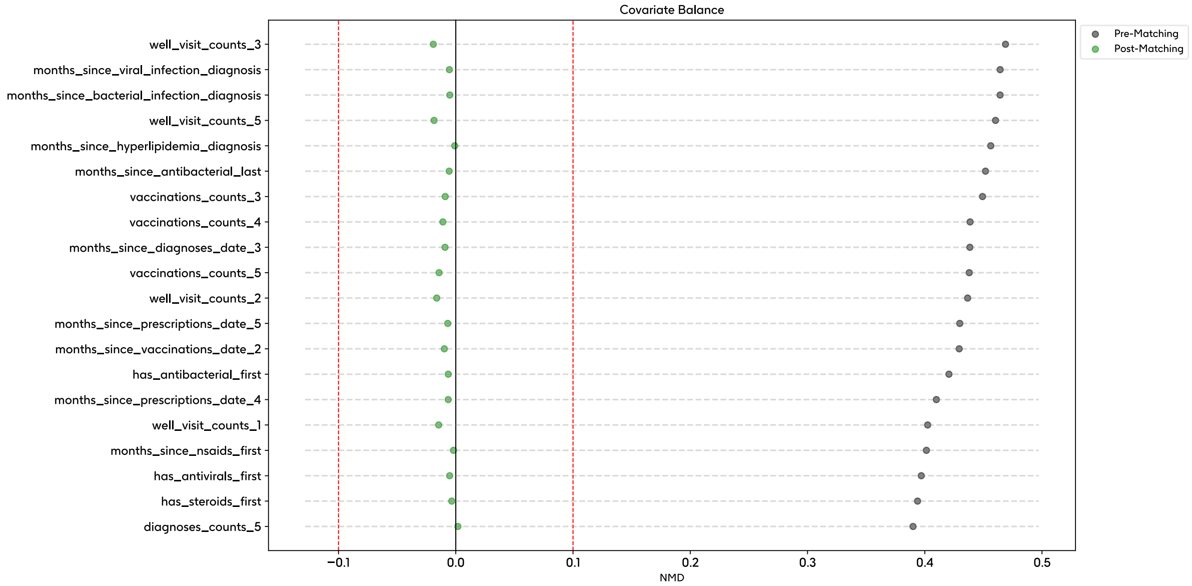
Covariate Balance
well_visit_counts_3
months_since_viral_infection_diagnosis
months_since_bacterial_infection_diagnosis
well_visit_counts_5
months_since_hyperlipidemia_diagnosis
months_since_antibacterial_last
vaccinations_counts_3
vaccinations_counts_4
months_since_diagnoses_date_3
vaccinations_counts_5
well_visit_counts_2
months_since_prescriptions_date_5
months_since_vaccinations_date_2
has_antibacterial_first
months_since_prescriptions_date_4
well_visit_counts_1
months_since_nsaids_first
has_antivirals_first
has_steroids_first
diagnoses_counts_5
−0.1
0.0
0.1
0.2
0.3
0.4
0.5
NMD
Pre-Matching
Post-Matching

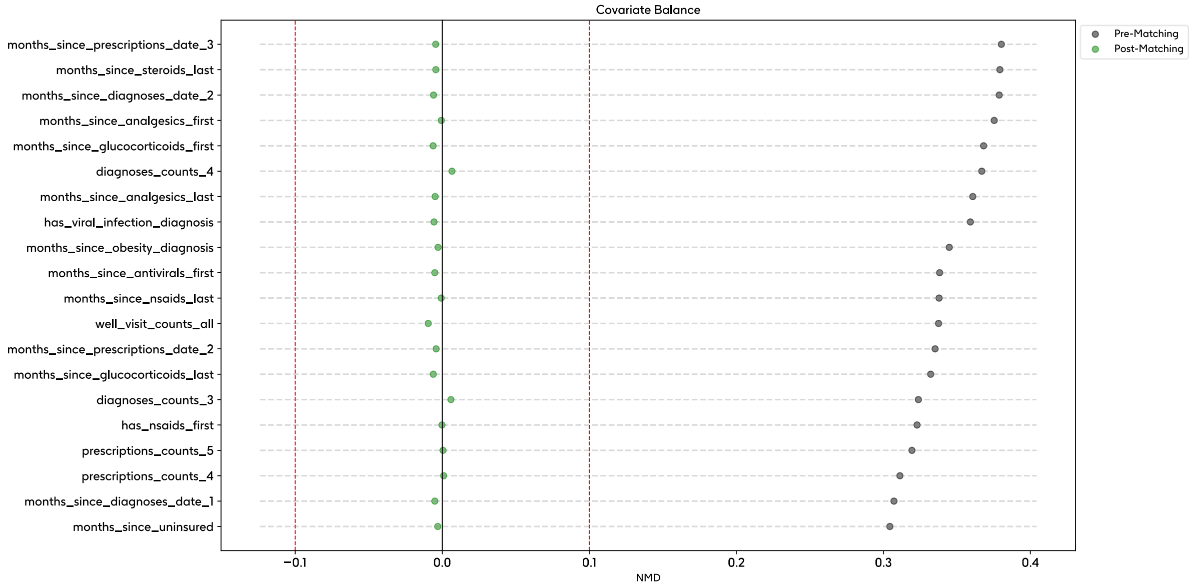
Covariate Balance
months_since_prescriptions_date_3
months_since_steroids_last
months_since_diagnoses_date_2
months_since_analgesics_first
months_since_glucocorticoids_first
diagnoses_counts_4
months_since_analgesics_last
has_viral_infection_diagnosis
months_since_obesity_diagnosis
months_since_antivirals_first
months_since_nsaids_last
well_visit_counts_all
months_since_prescriptions_date_2
months_since_glucocorticoids_last
diagnoses_counts_3
has_nsaids_first
prescriptions_counts_5
prescriptions_counts_4
months_since_diagnoses_date_1
months_since_uninsured
−0.1
0.0
0.1
0.2
0.3
0.4
NMD
Pre-Matching
Post-Matching

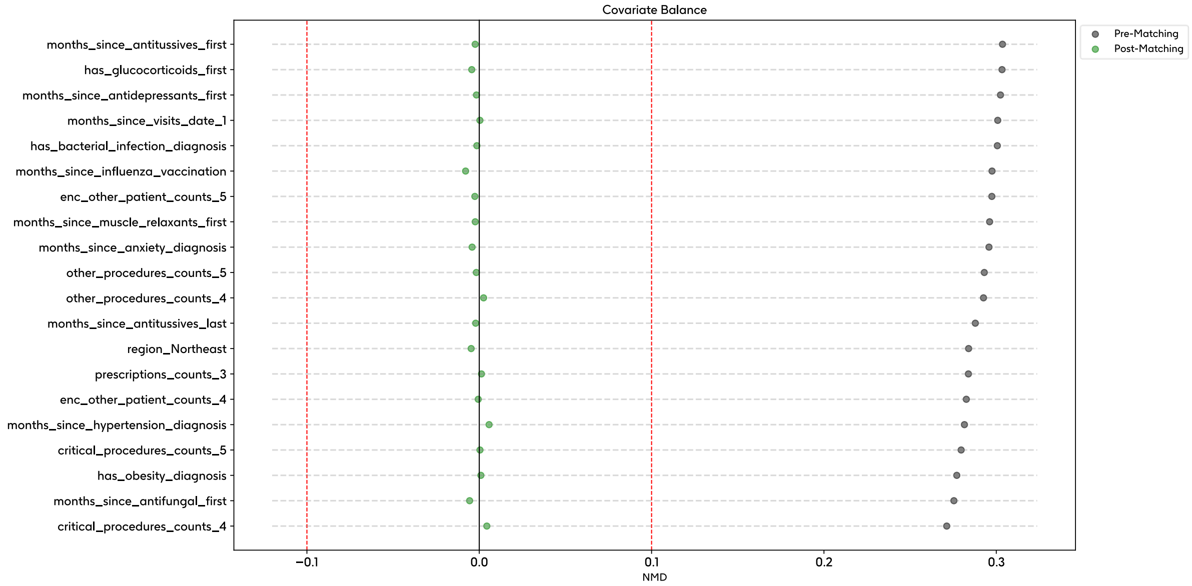
Covariate Balance
months_since_antitussives_first
has_glucocorticoids_first
months_since_antidepressants_first
months_since_visits_date_1
has_bacterial_infection_diagnosis
months_since_influenza_vaccination
enc_other_patient_counts_5
months_since_muscle_relaxants_first
months_since_anxiety_diagnosis
other_procedures_counts_5
other_procedures_counts_4
months_since_antitussives_last
region_Northeast
prescriptions_counts_3
enc_other_patient_counts_4
months_since_hypertension_diagnosis
critical_procedures_counts_5
has_obesity_diagnosis
months_since_antifungal_first
critical_procedures_counts_4
−0.1
0.0
0.1
0.2
0.3
NMD
Pre-Matching
Post-Matching

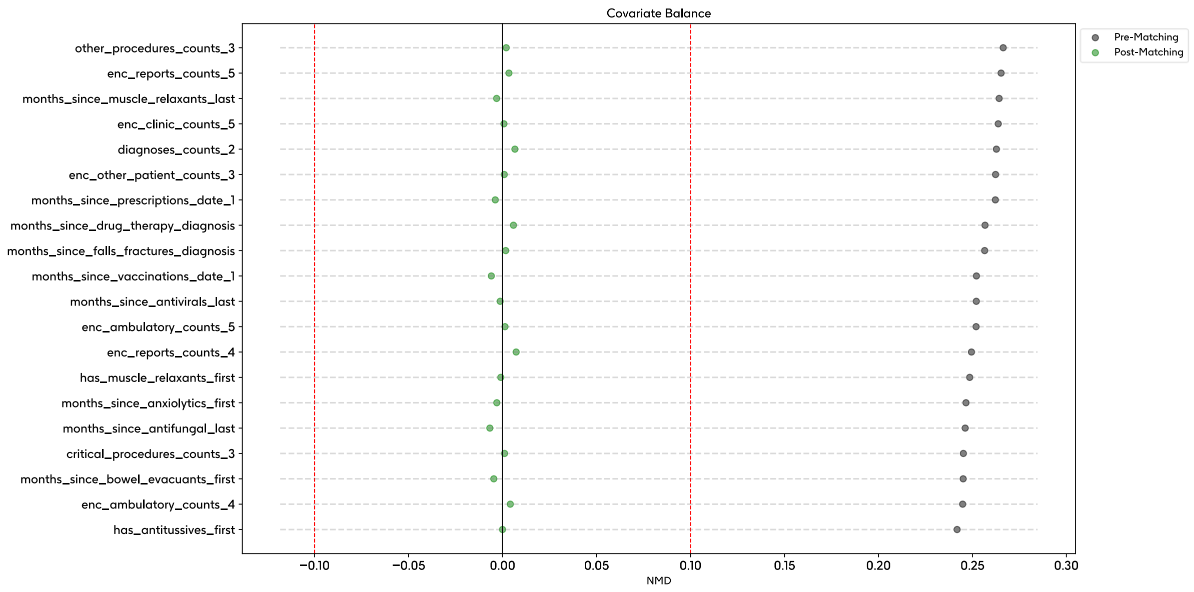
Covariate Balance
Pre-Matching
Post-Matching
other_procedures_counts_3
enc_reports_counts_5
months_since_muscle_relaxants_last
enc_clinic_counts_5
diagnoses_counts_2
enc_other_patient_counts_3
months_since_prescriptions_date_1
months_since_drug_therapy_diagnosis
months_since_falls_fractures_diagnosis
months_since_vaccinations_date_1
months_since_antivirals_last
enc_ambulatory_counts_5
enc_reports_counts_4
has_muscle_relaxants_first
months_since_anxiolytics_first
months_since_antifungal_last
critical_procedures_counts_3
months_since_bowel_evacuants_first
enc_ambulatory_counts_4
has_antitussives_first
−0.10 −0.05 0.00 0.05 0.10 0.15 0.20 0.25 0.30
NMD

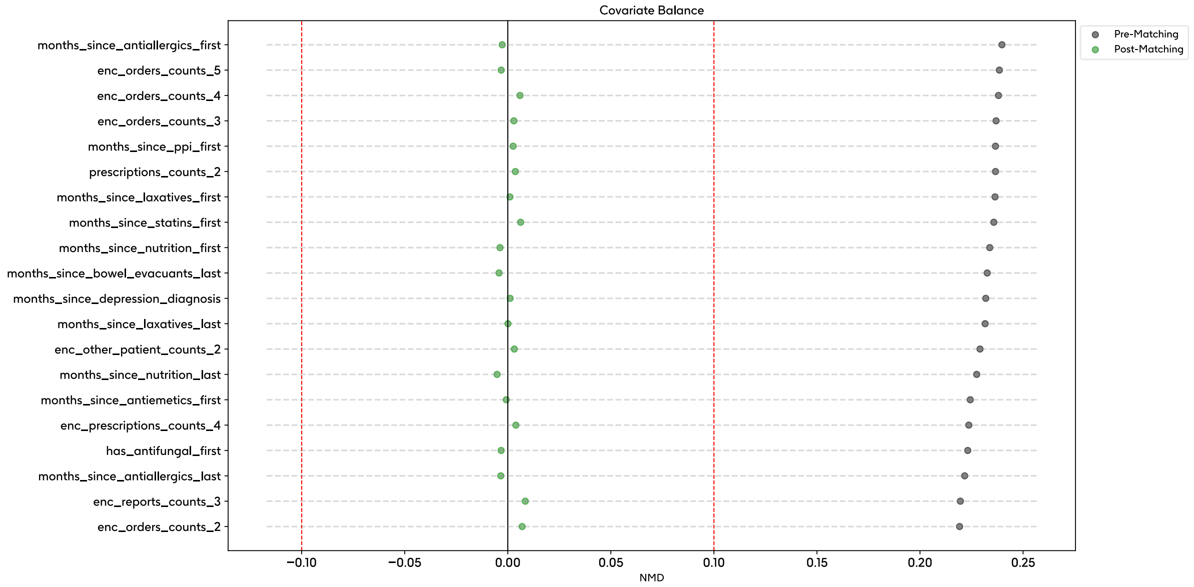
Covariate Balance
Pre-Matching
Post-Matching
months_since_antiallergics_first
enc_orders_counts_5
enc_orders_counts_4
enc_orders_counts_3
months_since_ppi_first
prescriptions_counts_2
months_since_laxatives_first
months_since_statins_first
months_since_nutrition_first
months_since_bowel_evacuants_last
months_since_depression_diagnosis
months_since_laxatives_last
enc_other_patient_counts_2
months_since_nutrition_last
months_since_antiemetics_first
enc_prescriptions_counts_4
has_antifungal_first
months_since_antiallergics_last
enc_reports_counts_3
enc_orders_counts_2
−0.10 −0.05 0.00 0.05 0.10 0.15 0.20 0.25
NMD

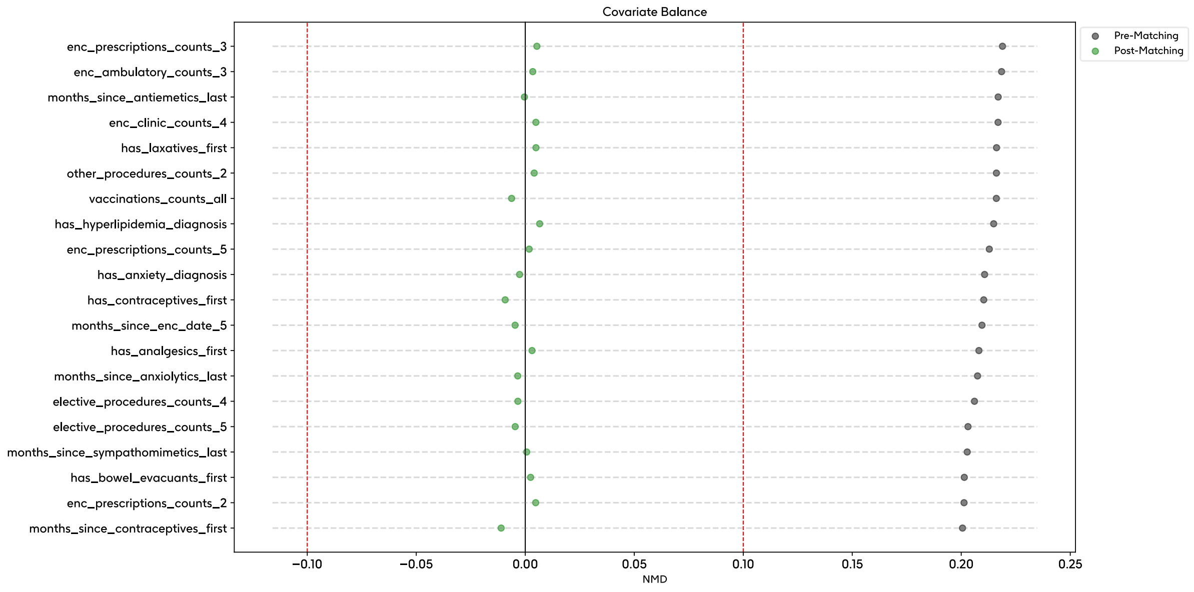
Covariate Balance
Pre-Matching
Post-Matching
enc_prescriptions_counts_3
enc_ambulatory_counts_3
months_since_antiemetics_last
enc_clinic_counts_4
has_laxatives_first
other_procedures_counts_2
vaccinations_counts_all
has_hyperlipidemia_diagnosis
enc_prescriptions_counts_5
has_anxiety_diagnosis
has_contraceptives_first
months_since_enc_date_5
has_analgesics_first
months_since_anxiolytics_last
elective_procedures_counts_4
elective_procedures_counts_5
months_since_sympathomimetics_last
has_bowel_evacuants_first
enc_prescriptions_counts_2
months_since_contraceptives_first
−0.10 −0.05 0.00 0.05 0.10 0.15 0.20 0.25
NMD

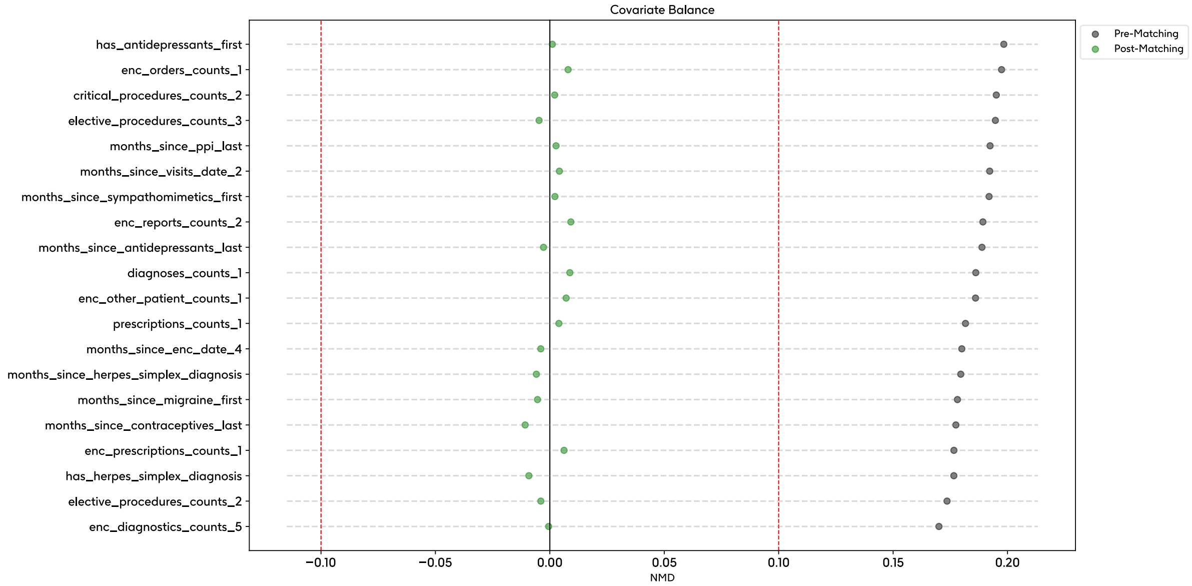
Covariate Balance
Pre-Matching
Post-Matching
has_antidepressants_first
enc_orders_counts_1
critical_procedures_counts_2
elective_procedures_counts_3
months_since_ppi_last
months_since_visits_date_2
months_since_sympathomimetics_first
enc_reports_counts_2
months_since_antidepressants_last
diagnoses_counts_1
enc_other_patient_counts_1
prescriptions_counts_1
months_since_enc_date_4
months_since_herpes_simplex_diagnosis
months_since_migraine_first
months_since_contraceptives_last
enc_prescriptions_counts_1
has_herpes_simplex_diagnosis
elective_procedures_counts_2
enc_diagnostics_counts_5
−0.10
−0.05
0.00
0.05
0.10
0.15
0.20
NMD

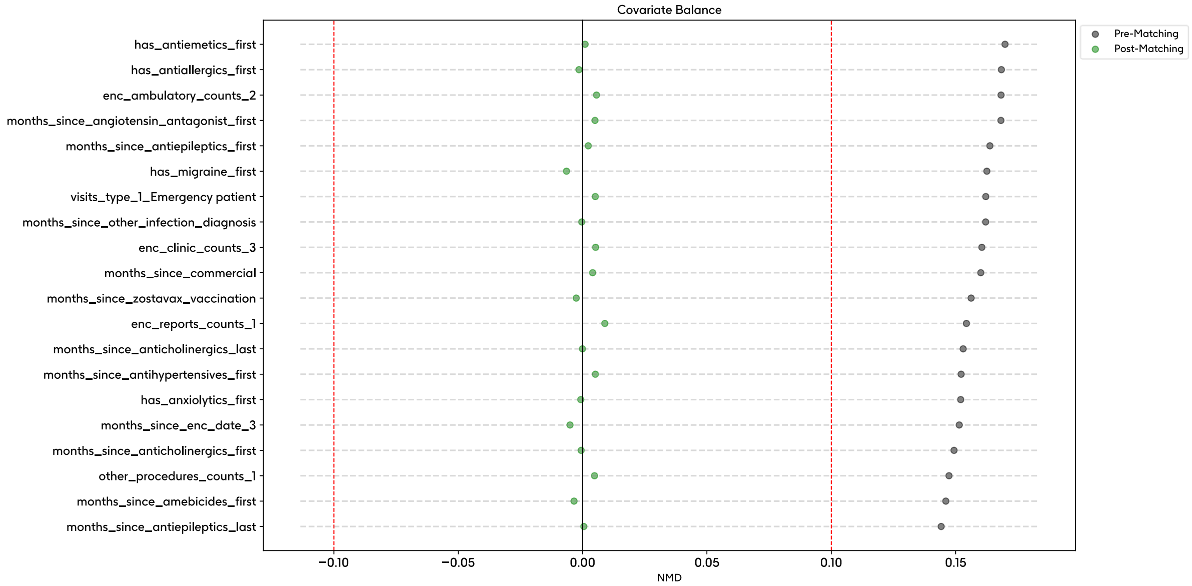
Covariate Balance
Pre-Matching
Post-Matching
has_antiemetics_first
has_antiallergics_first
enc_ambulatory_counts_2
months_since_angiotensin_antagonist_first
months_since_antiepileptics_first
has_migraine_first
visits_type_1_Emergency patient
months_since_other_infection_diagnosis
enc_clinic_counts_3
months_since_commercial
months_since_zostavax_vaccination
enc_reports_counts_1
months_since_anticholinergics_last
months_since_antihypertensives_first
has_anxiolytics_first
months_since_enc_date_3
months_since_anticholinergics_first
other_procedures_counts_1
months_since_amebicides_first
months_since_antiepileptics_last
−0.10
−0.05
0.00
0.05
0.10
0.15
NMD

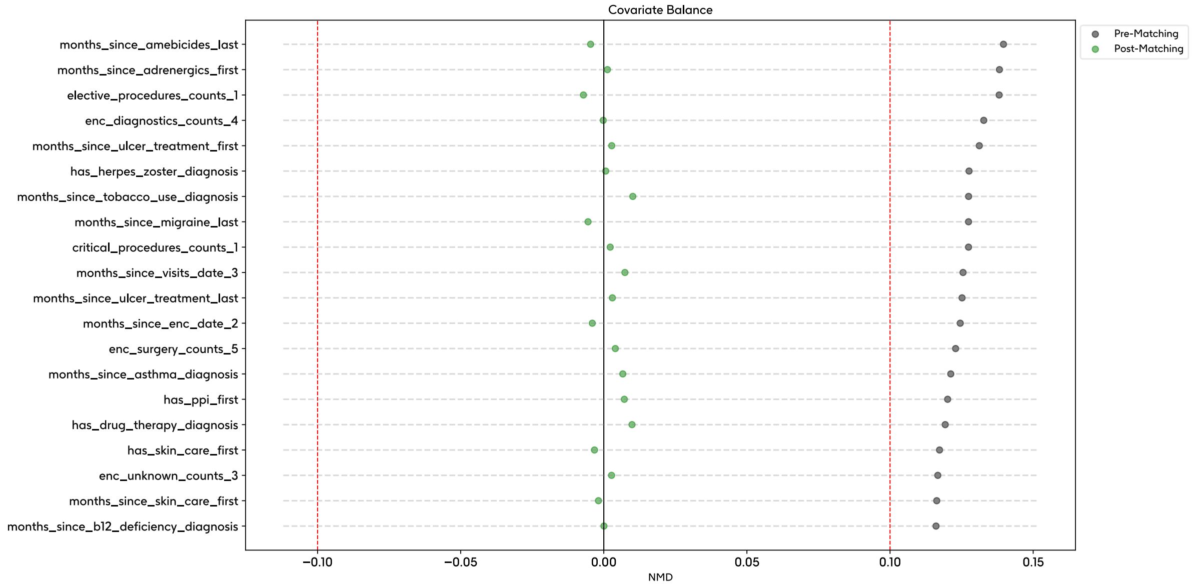
Covariate Balance
Pre-Matching
Post-Matching
months_since_amebicides_last
months_since_adrenergics_first
elective_procedures_counts_1
enc_diagnostics_counts_4
months_since_ulcer_treatment_first
has_herpes_zoster_diagnosis
months_since_tobacco_use_diagnosis
months_since_migraine_last
critical_procedures_counts_1
months_since_visits_date_3
months_since_ulcer_treatment_last
months_since_enc_date_2
enc_surgery_counts_5
months_since_asthma_diagnosis
has_ppi_first
has_drug_therapy_diagnosis
has_skin_care_first
enc_unknown_counts_3
months_since_skin_care_first
months_since_b12_deficiency_diagnosis
−0.10
−0.05
0.00
0.05
0.10
0.15
NMD

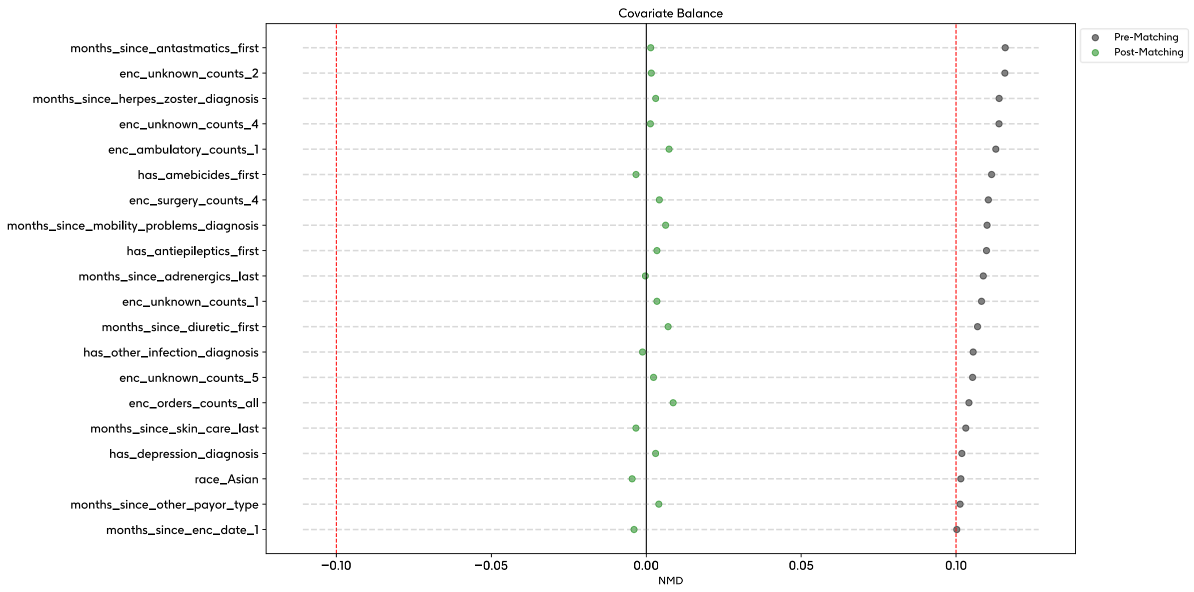

(c) Dementia (code OR medication): ZVL vs PPSV23 (all)

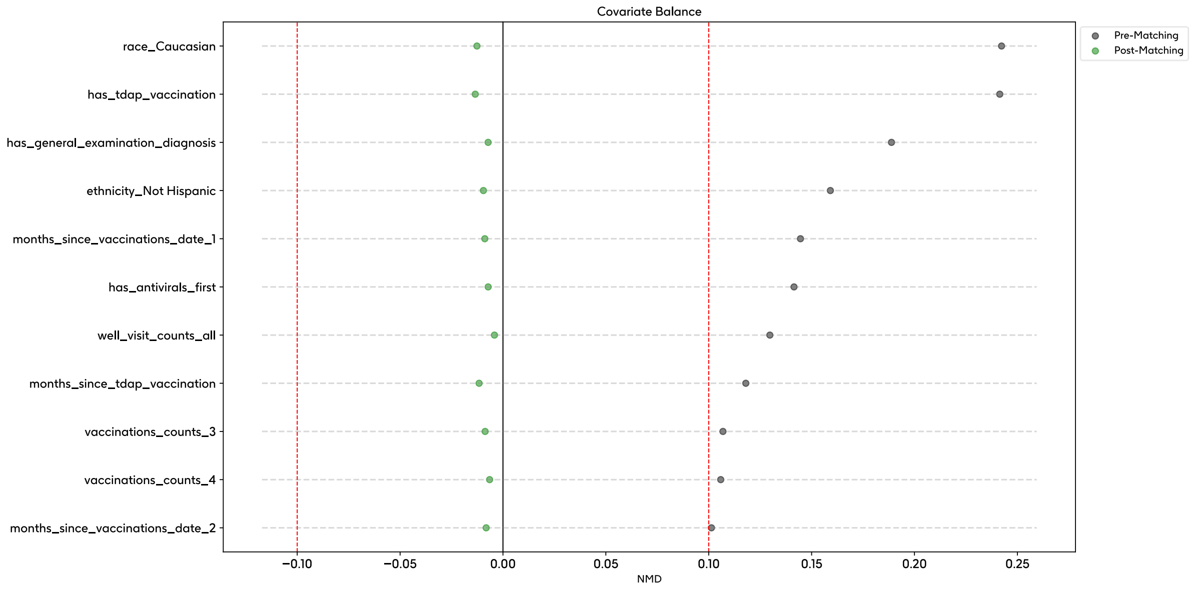

(d) Dementia (code OR medication): RZV (2+ doses) vs PPSV23 (all)

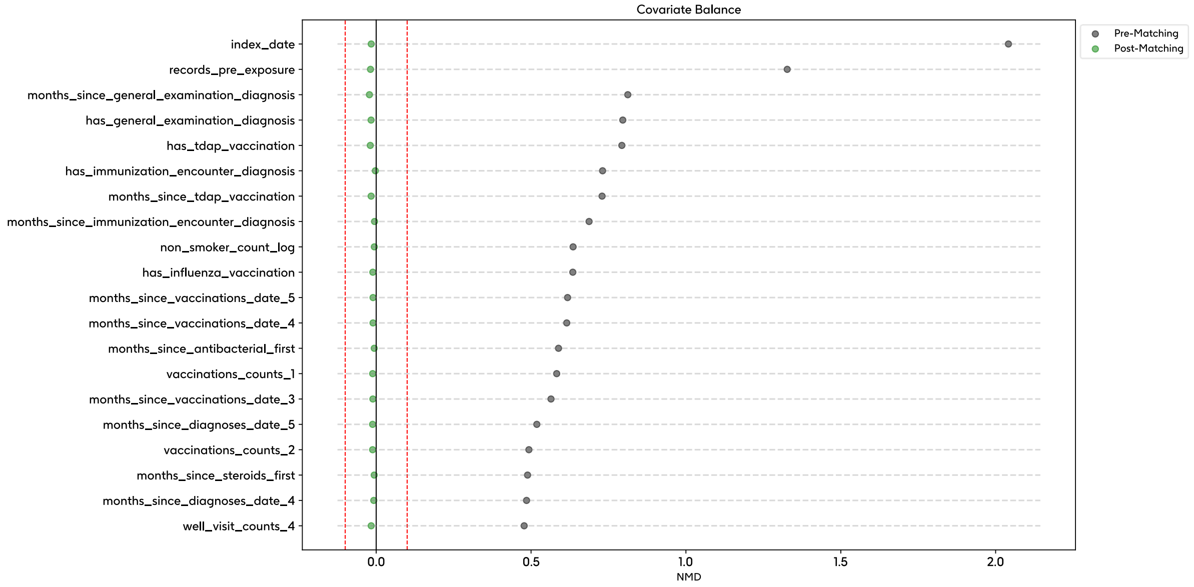

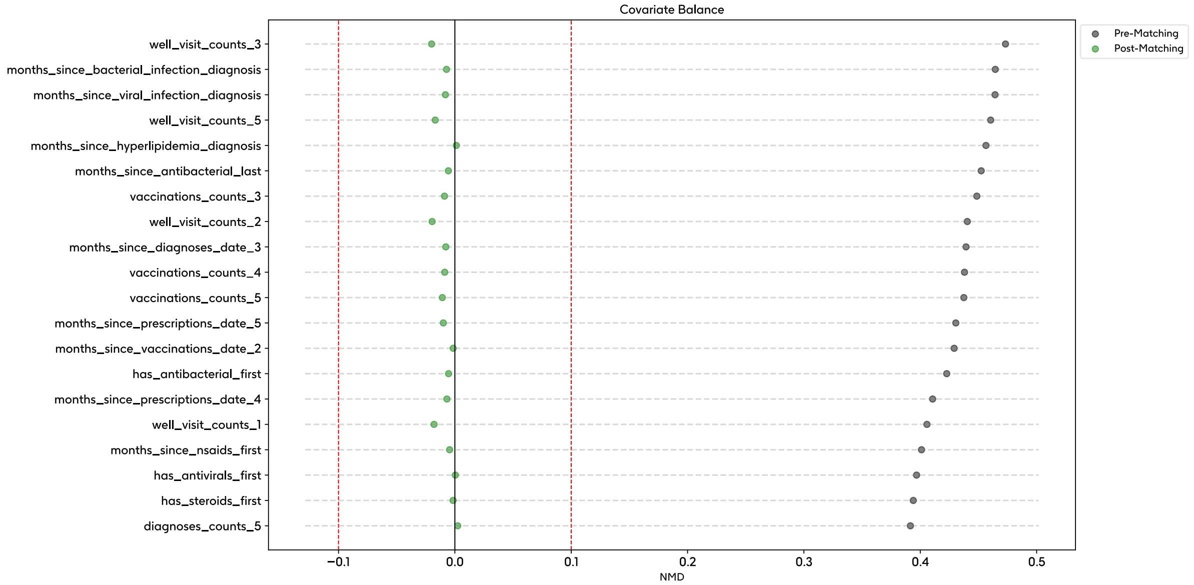

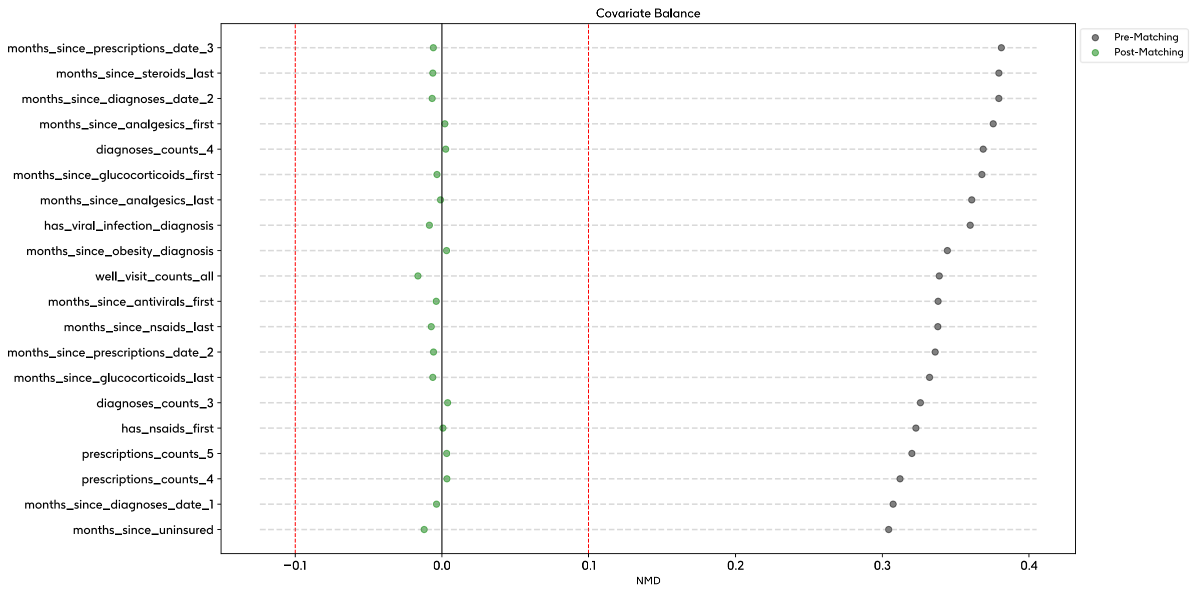
Covariate Balance
months_since_prescriptions_date_3
months_since_steroids_last
months_since_diagnoses_date_2
months_since_analgesics_first
diagnoses_counts_4
months_since_glucocorticoids_first
months_since_analgesics_last
has_viral_infection_diagnosis
months_since_obesity_diagnosis
well_visit_counts_all
months_since_antivirals_first
months_since_nsaids_last
months_since_prescriptions_date_2
months_since_glucocorticoids_last
diagnoses_counts_3
has_nsaids_first
prescriptions_counts_5
prescriptions_counts_4
months_since_diagnoses_date_1
months_since_uninsured
−0.1
0.0
0.1
0.2
0.3
0.4
NMD
Pre-Matching
Post-Matching

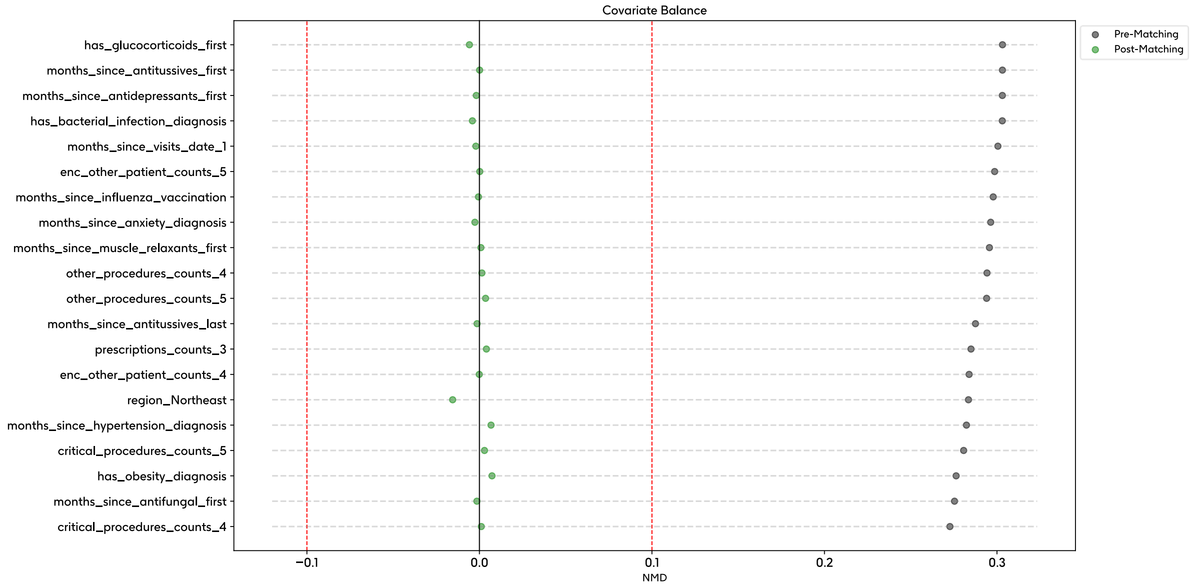
Covariate Balance
has_glucocorticoids_first
months_since_antitussives_first
months_since_antidepressants_first
has_bacterial_infection_diagnosis
months_since_visits_date_1
enc_other_patient_counts_5
months_since_influenza_vaccination
months_since_anxiety_diagnosis
months_since_muscle_relaxants_first
other_procedures_counts_4
other_procedures_counts_5
months_since_antitussives_last
prescriptions_counts_3
enc_other_patient_counts_4
region_Northeast
months_since_hypertension_diagnosis
critical_procedures_counts_5
has_obesity_diagnosis
months_since_antifungal_first
critical_procedures_counts_4
−0.1
0.0
0.1
0.2
0.3
NMD
Pre-Matching
Post-Matching

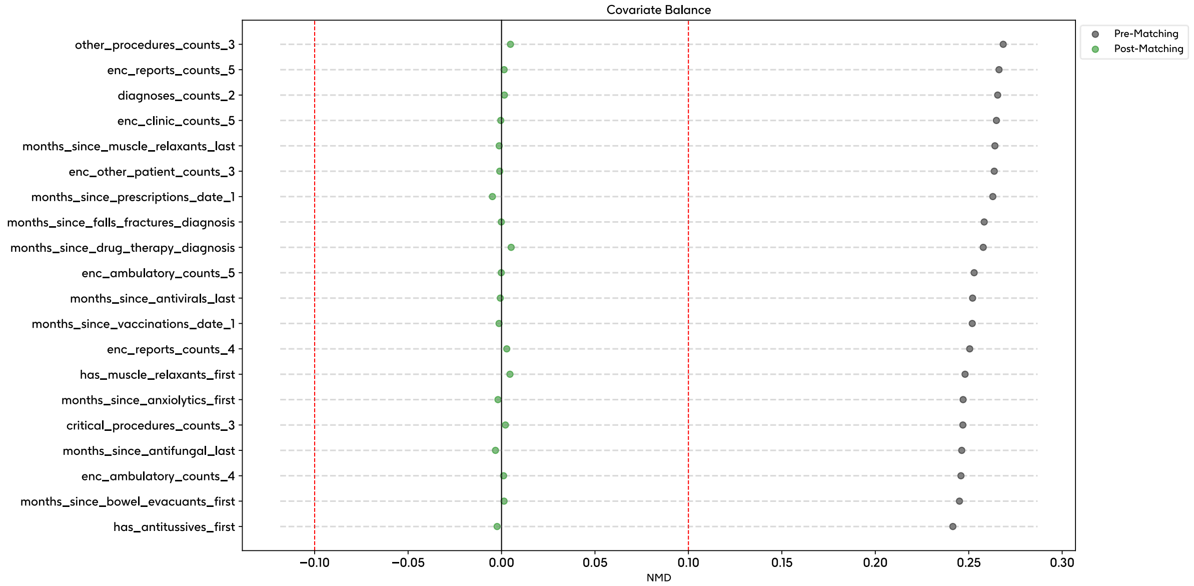
Covariate Balance
other_procedures_counts_3
enc_reports_counts_5
diagnoses_counts_2
enc_clinic_counts_5
months_since_muscle_relaxants_last
enc_other_patient_counts_3
months_since_prescriptions_date_1
months_since_falls_fractures_diagnosis
months_since_drug_therapy_diagnosis
enc_ambulatory_counts_5
months_since_antivirals_last
months_since_vaccinations_date_1
enc_reports_counts_4
has_muscle_relaxants_first
months_since_anxiolytics_first
critical_procedures_counts_3
months_since_antifungal_last
enc_ambulatory_counts_4
months_since_bowel_evacuants_first
has_antitussives_first
−0.10
−0.05
0.00
0.05
0.10
0.15
0.20
0.25
0.30
NMD
Pre-Matching
Post-Matching

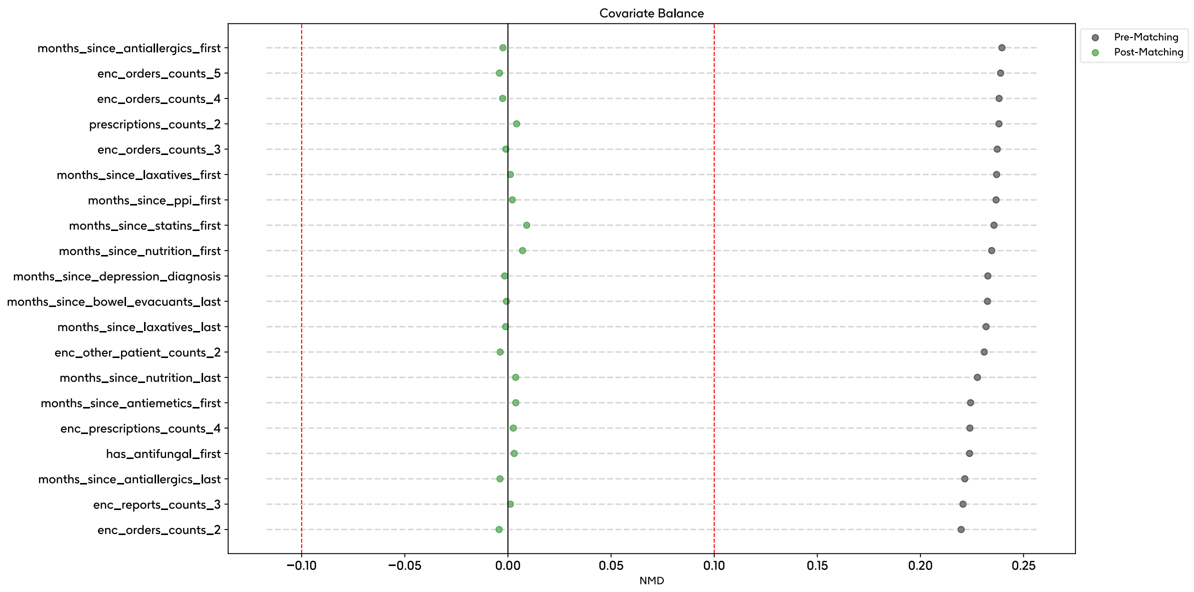
Covariate Balance
months_since_antiallergics_first
enc_orders_counts_5
enc_orders_counts_4
prescriptions_counts_2
enc_orders_counts_3
months_since_laxatives_first
months_since_ppi_first
months_since_statins_first
months_since_nutrition_first
months_since_depression_diagnosis
months_since_bowel_evacuants_last
months_since_laxatives_last
enc_other_patient_counts_2
months_since_nutrition_last
months_since_antiemetics_first
enc_prescriptions_counts_4
has_antifungal_first
months_since_antiallergics_last
enc_reports_counts_3
enc_orders_counts_2
Pre-Matching
Post-Matching
−0.10
−0.05
0.00
0.05
0.10
0.15
0.20
0.25
NMD

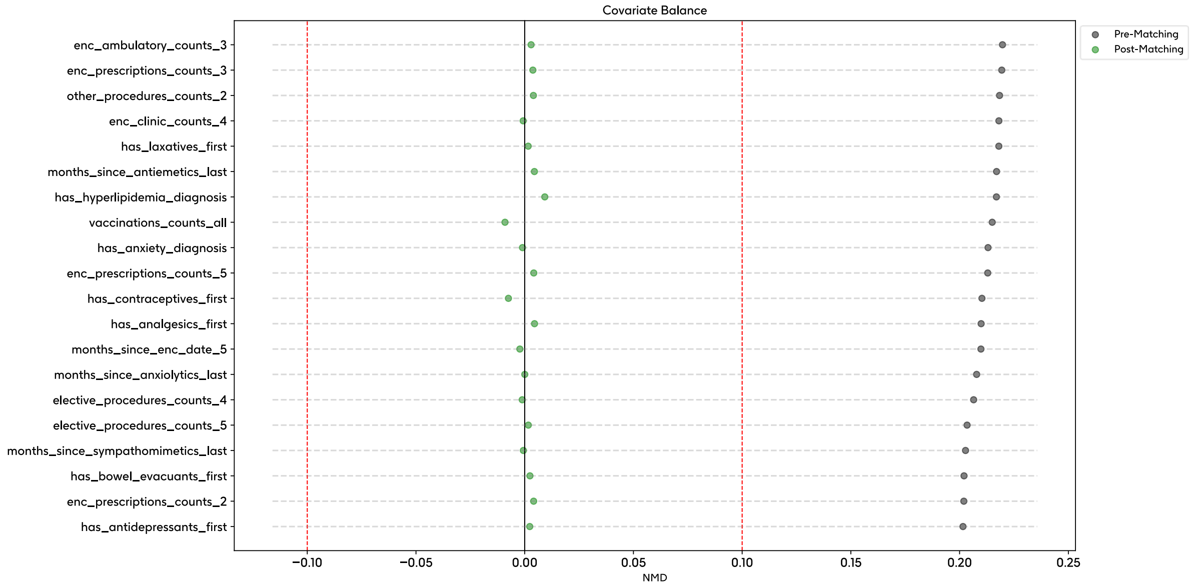
Covariate Balance
enc_ambulatory_counts_3
enc_prescriptions_counts_3
other_procedures_counts_2
enc_clinic_counts_4
has_laxatives_first
months_since_antiemetics_last
has_hyperlipidemia_diagnosis
vaccinations_counts_all
has_anxiety_diagnosis
enc_prescriptions_counts_5
has_contraceptives_first
has_analgesics_first
months_since_enc_date_5
months_since_anxiolytics_last
elective_procedures_counts_4
elective_procedures_counts_5
months_since_sympathomimetics_last
has_bowel_evacuants_first
enc_prescriptions_counts_2
has_antidepressants_first
Pre-Matching
Post-Matching
−0.10
−0.05
0.00
0.05
0.10
0.15
0.20
0.25
NMD

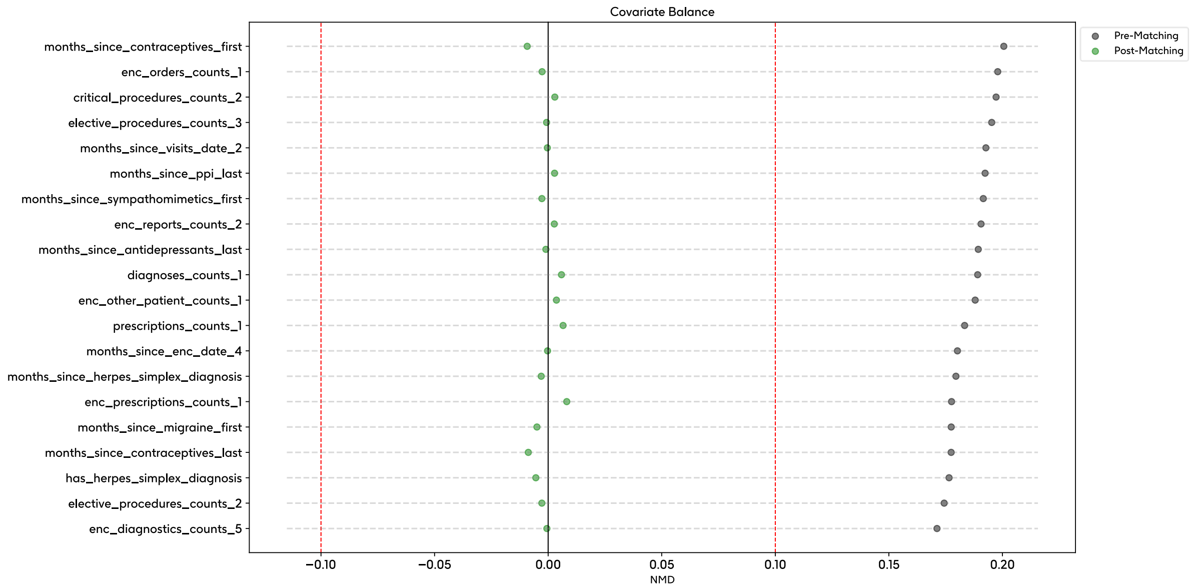
Covariate Balance
months_since_contraceptives_first
enc_orders_counts_1
critical_procedures_counts_2
elective_procedures_counts_3
months_since_visits_date_2
months_since_ppi_last
months_since_sympathomimetics_first
enc_reports_counts_2
months_since_antidepressants_last
diagnoses_counts_1
enc_other_patient_counts_1
prescriptions_counts_1
months_since_enc_date_4
months_since_herpes_simplex_diagnosis
enc_prescriptions_counts_1
months_since_migraine_first
months_since_contraceptives_last
has_herpes_simplex_diagnosis
elective_procedures_counts_2
enc_diagnostics_counts_5
Pre-Matching
Post-Matching
−0.10
−0.05
0.00
0.05
0.10
0.15
0.20
NMD

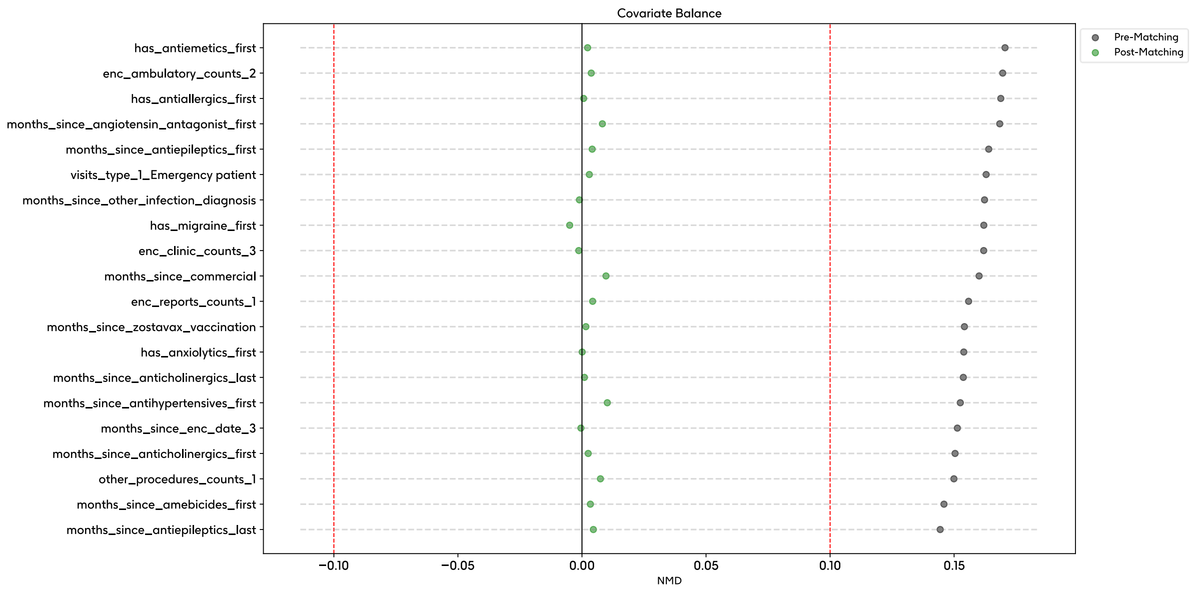

Covariate Balance
Pre-Matching
Post-Matching
has_antiemetics_first
enc_ambulatory_counts_2
has_antiallergics_first
months_since_angiotensin_antagonist_first
months_since_antiepileptics_first
visits_type_1_Emergency patient
months_since_other_infection_diagnosis
has_migraine_first
enc_clinic_counts_3
months_since_commercial
enc_reports_counts_1
months_since_zostavax_vaccination
has_anxiolytics_first
months_since_anticholinergics_last
months_since_antihypertensives_first
months_since_enc_date_3
months_since_anticholinergics_first
other_procedures_counts_1
months_since_amebicides_first
months_since_antiepileptics_last
−0.10
−0.05
0.00
0.05
0.10
0.15
NMD

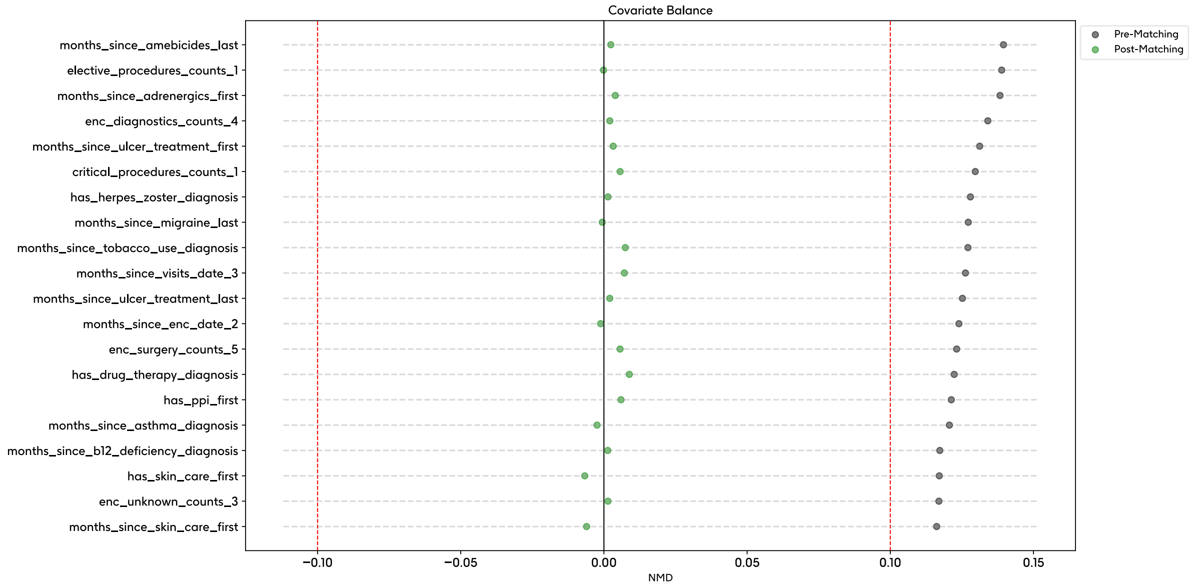

Covariate Balance
Pre-Matching
Post-Matching
months_since_amebicides_last
elective_procedures_counts_1
months_since_adrenergics_first
enc_diagnostics_counts_4
months_since_ulcer_treatment_first
critical_procedures_counts_1
has_herpes_zoster_diagnosis
months_since_migraine_last
months_since_tobacco_use_diagnosis
months_since_visits_date_3
months_since_ulcer_treatment_last
months_since_enc_date_2
enc_surgery_counts_5
has_drug_therapy_diagnosis
has_ppi_first
months_since_asthma_diagnosis
months_since_b12_deficiency_diagnosis
has_skin_care_first
enc_unknown_counts_3
months_since_skin_care_first
−0.10
−0.05
0.00
0.05
0.10
0.15
NMD

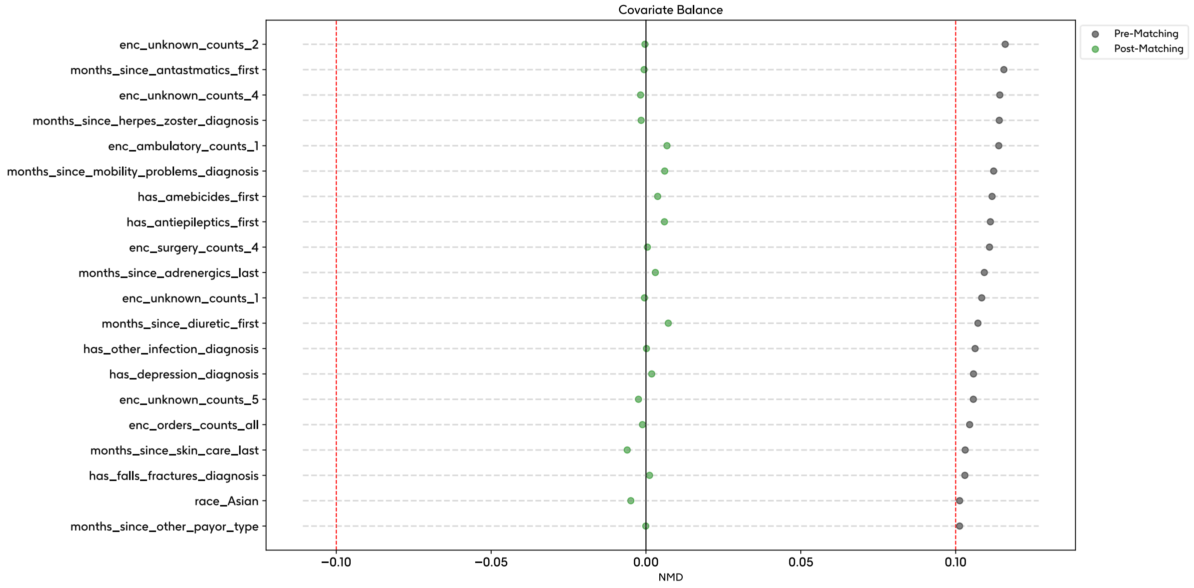

Covariate Balance
Pre-Matching
Post-Matching
enc_unknown_counts_2
months_since_antastmatics_first
enc_unknown_counts_4
months_since_herpes_zoster_diagnosis
enc_ambulatory_counts_1
months_since_mobility_problems_diagnosis
has_amebicides_first
has_antiepileptics_first
enc_surgery_counts_4
months_since_adrenergics_last
enc_unknown_counts_1
months_since_diuretic_first
has_other_infection_diagnosis
has_depression_diagnosis
enc_unknown_counts_5
enc_orders_counts_all
months_since_skin_care_last
has_falls_fractures_diagnosis
race_Asian
months_since_other_payor_type
−0.10
−0.05
0.00
0.05
0.10
NMD

Supplementary Fig. S5 | Pre- and post-matching cohort balance for comparisons of cohorts using different dementia definitions.
The values on the y-axis are the names of covariates. The dots indicate pre- and post-matching normalized mean distance (NMD) for the corresponding covariate. The red vertical lines indicate the bounds of what is considered a good balance (-0.1 < NMD < 0.1). Post-matching dots within these bounds indicate that the corresponding covariate's mean is well balanced after matching. The covariates are sorted in descending order of their pre-matching NMD.
Note for interpretation of covariate names: For covariates named "months_since_i" (where i=1, 2, 3, 4, 5), the name indicates months between ith last intervention pre-exposure and the exposure date. For "months_since_vaccination", the term "vaccination" refers to any vaccination except the exposure vaccination). For "months_since_diagnosis" covariate, the term "diagnosis" refers to any diagnosis in the Diagnosis table with a certain status. For covariates named "intervention_counts_i", the name indicates the number of interventions in the ith pre-exposure year. The covariate named "well_visits_counts_all" indicates the total count of routine medical and gynecological examinations.

Code AND medication, dementia defined by presence of both a diagnostic code and record of prescribed medication; Code OR mediation, dementia defined by either a presence of a diagnostic code or record of prescribed medication; PPSV23, recipients of at least 1 dose of a 23-valent pneumococcal polysaccharide vaccine; RZV (2+ doses), recipients of at least 2 doses of the recombinant zoster vaccine (*Shingrix*, GSK); ZVL, recipients of at least 1 dose of the live-attenuated zoster vaccine (*Zostavax*, Merck).

(a) Alzheimer's disease: ZVL vs PPSV23 (all)

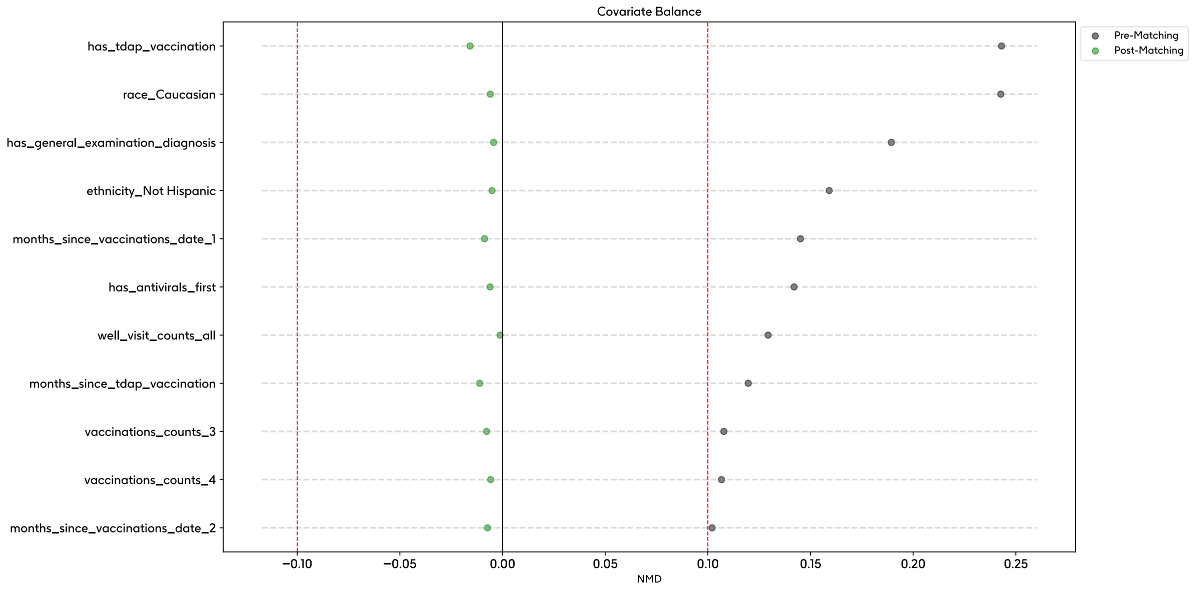

(b) Alzheimer's disease: RZV (2+ doses) vs PPSV23 (all)

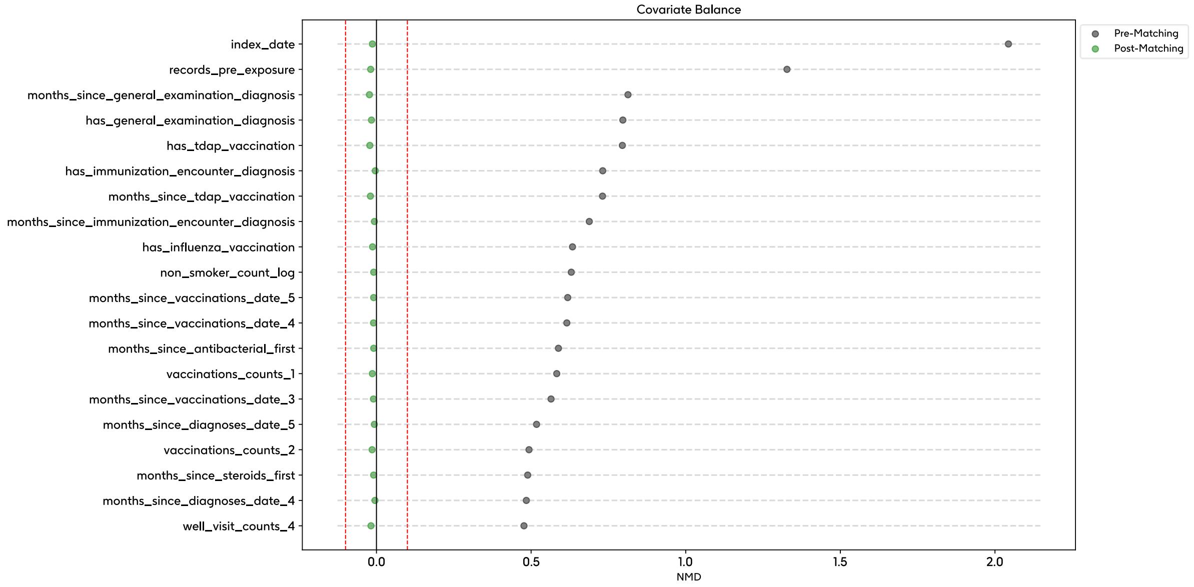

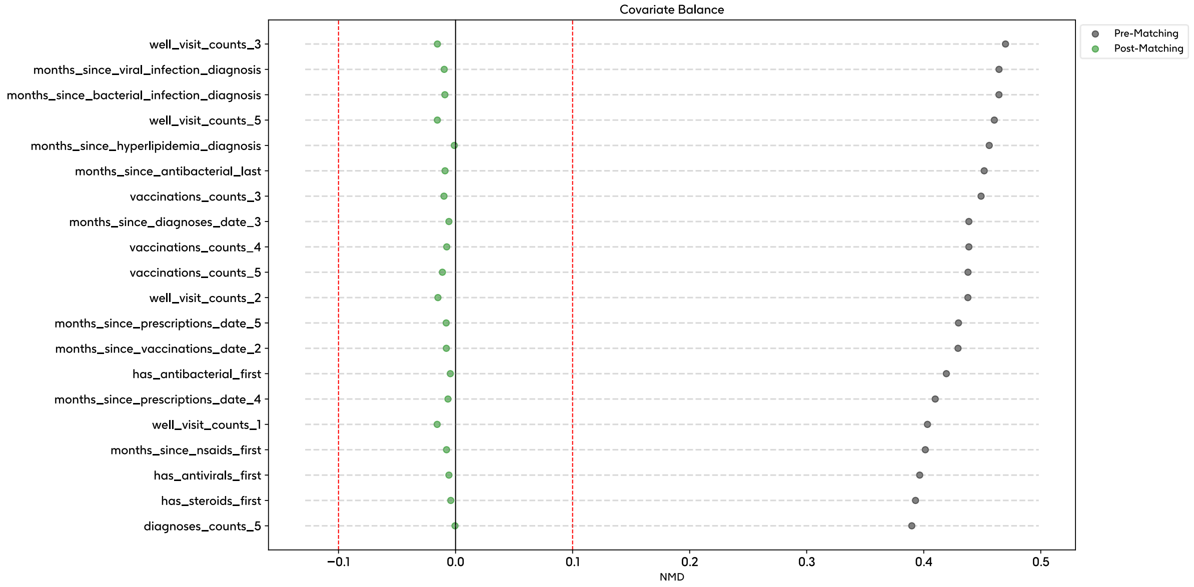

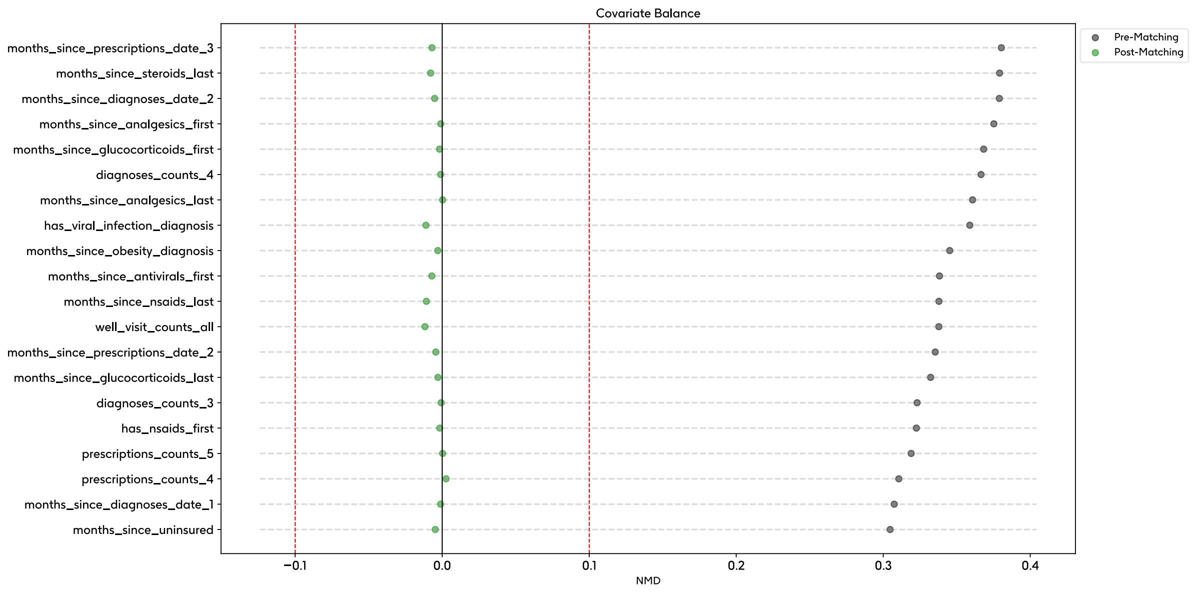
Covariate Balance
months_since_prescriptions_date_3
months_since_steroids_last
months_since_diagnoses_date_2
months_since_analgesics_first
months_since_glucocorticoids_first
diagnoses_counts_4
months_since_analgesics_last
has_viral_infection_diagnosis
months_since_obesity_diagnosis
months_since_antivirals_first
months_since_nsaids_last
well_visit_counts_all
months_since_prescriptions_date_2
months_since_glucocorticoids_last
diagnoses_counts_3
has_nsaids_first
prescriptions_counts_5
prescriptions_counts_4
months_since_diagnoses_date_1
months_since_uninsured
−0.1
0.0
0.1
0.2
0.3
0.4
NMD
Pre-Matching
Post-Matching

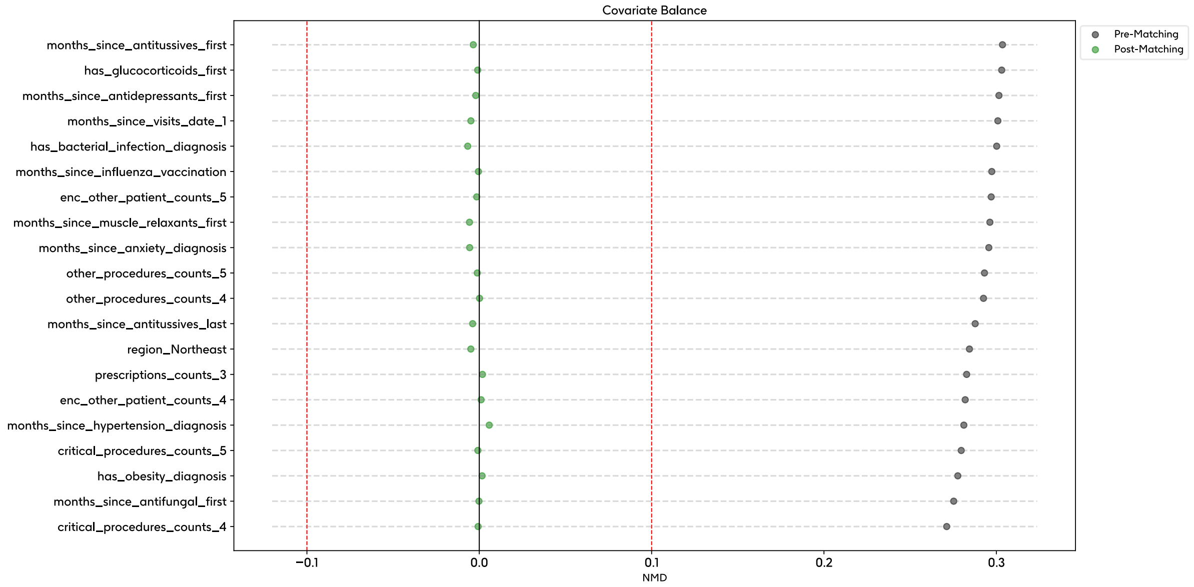
Covariate Balance
months_since_antitussives_first
has_glucocorticoids_first
months_since_antidepressants_first
months_since_visits_date_1
has_bacterial_infection_diagnosis
months_since_influenza_vaccination
enc_other_patient_counts_5
months_since_muscle_relaxants_first
months_since_anxiety_diagnosis
other_procedures_counts_5
other_procedures_counts_4
months_since_antitussives_last
region_Northeast
prescriptions_counts_3
enc_other_patient_counts_4
months_since_hypertension_diagnosis
critical_procedures_counts_5
has_obesity_diagnosis
months_since_antifungal_first
critical_procedures_counts_4
−0.1
0.0
0.1
0.2
0.3
NMD
Pre-Matching
Post-Matching

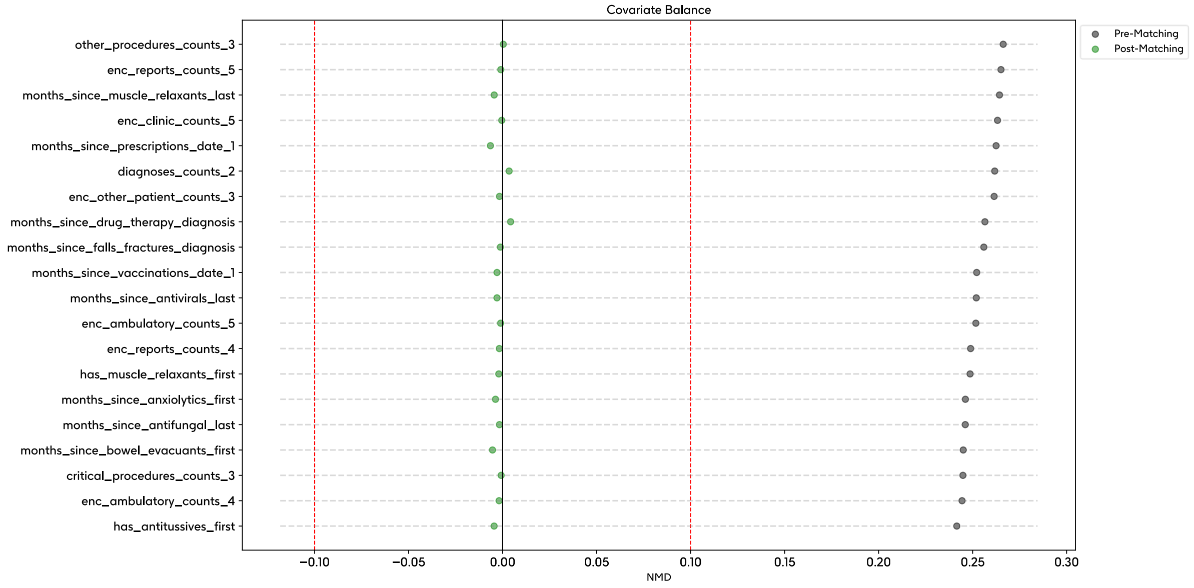
Covariate Balance
other_procedures_counts_3
enc_reports_counts_5
months_since_muscle_relaxants_last
enc_clinic_counts_5
months_since_prescriptions_date_1
diagnoses_counts_2
enc_other_patient_counts_3
months_since_drug_therapy_diagnosis
months_since_falls_fractures_diagnosis
months_since_vaccinations_date_1
months_since_antivirals_last
enc_ambulatory_counts_5
enc_reports_counts_4
has_muscle_relaxants_first
months_since_anxiolytics_first
months_since_antifungal_last
months_since_bowel_evacuants_first
critical_procedures_counts_3
enc_ambulatory_counts_4
has_antitussives_first
−0.10
−0.05
0.00
0.05
0.10
0.15
0.20
0.25
0.30
NMD
Pre-Matching
Post-Matching

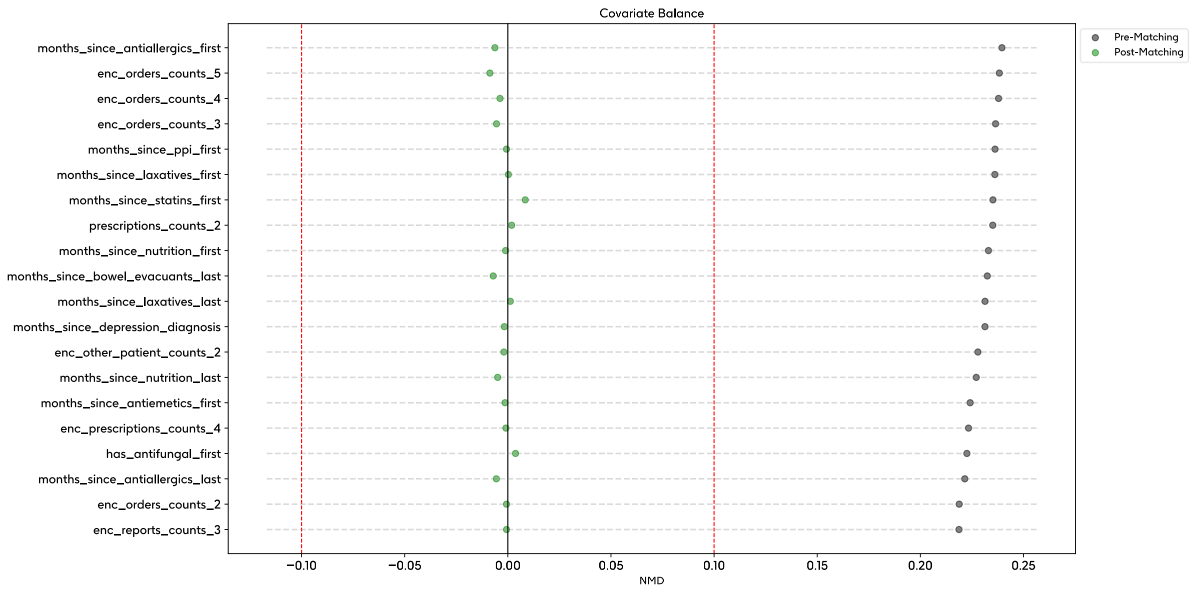
Covariate Balance
months_since_antiallergics_first
enc_orders_counts_5
enc_orders_counts_4
enc_orders_counts_3
months_since_ppi_first
months_since_laxatives_first
months_since_statins_first
prescriptions_counts_2
months_since_nutrition_first
months_since_bowel_evacuants_last
months_since_laxatives_last
months_since_depression_diagnosis
enc_other_patient_counts_2
months_since_nutrition_last
months_since_antiemetics_first
enc_prescriptions_counts_4
has_antifungal_first
months_since_antiallergics_last
enc_orders_counts_2
enc_reports_counts_3
Pre-Matching
Post-Matching
−0.10 −0.05 0.00 0.05 0.10 0.15 0.20 0.25
NMD

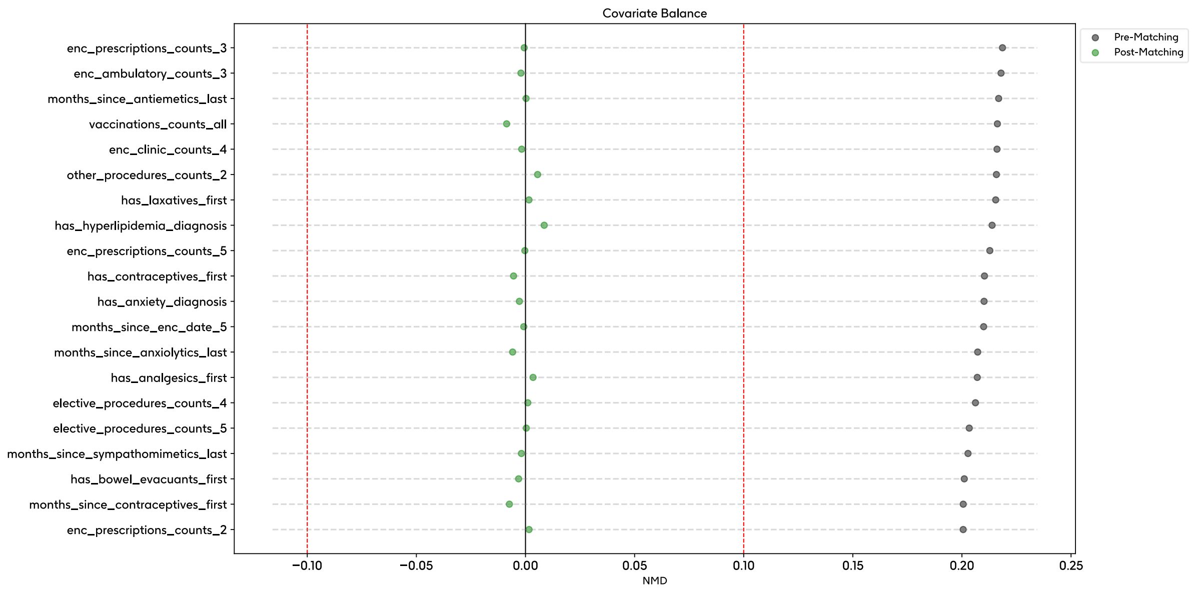
Covariate Balance
enc_prescriptions_counts_3
enc_ambulatory_counts_3
months_since_antiemetics_last
vaccinations_counts_all
enc_clinic_counts_4
other_procedures_counts_2
has_laxatives_first
has_hyperlipidemia_diagnosis
enc_prescriptions_counts_5
has_contraceptives_first
has_anxiety_diagnosis
months_since_enc_date_5
months_since_anxiolytics_last
has_analgesics_first
elective_procedures_counts_4
elective_procedures_counts_5
months_since_sympathomimetics_last
has_bowel_evacuants_first
months_since_contraceptives_first
enc_prescriptions_counts_2
Pre-Matching
Post-Matching
−0.10 −0.05 0.00 0.05 0.10 0.15 0.20 0.25
NMD

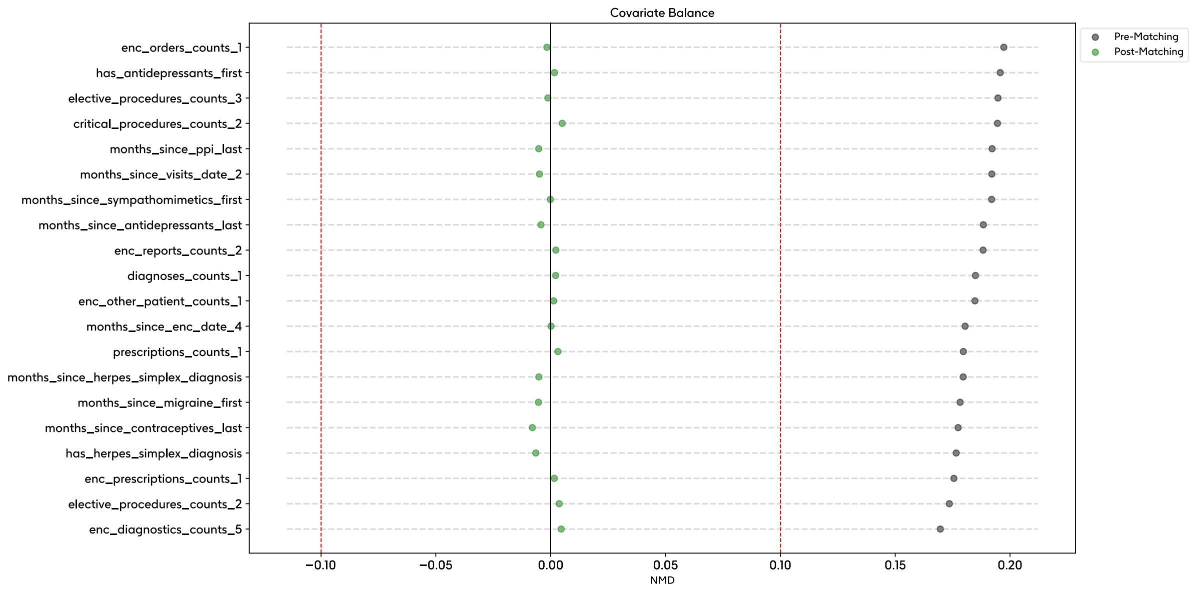
Covariate Balance
enc_orders_counts_1
has_antidepressants_first
elective_procedures_counts_3
critical_procedures_counts_2
months_since_ppi_last
months_since_visits_date_2
months_since_sympathomimetics_first
months_since_antidepressants_last
enc_reports_counts_2
diagnoses_counts_1
enc_other_patient_counts_1
months_since_enc_date_4
prescriptions_counts_1
months_since_herpes_simplex_diagnosis
months_since_migraine_first
months_since_contraceptives_last
has_herpes_simplex_diagnosis
enc_prescriptions_counts_1
elective_procedures_counts_2
enc_diagnostics_counts_5
Pre-Matching
Post-Matching
−0.10 −0.05 0.00 0.05 0.10 0.15 0.20
NMD

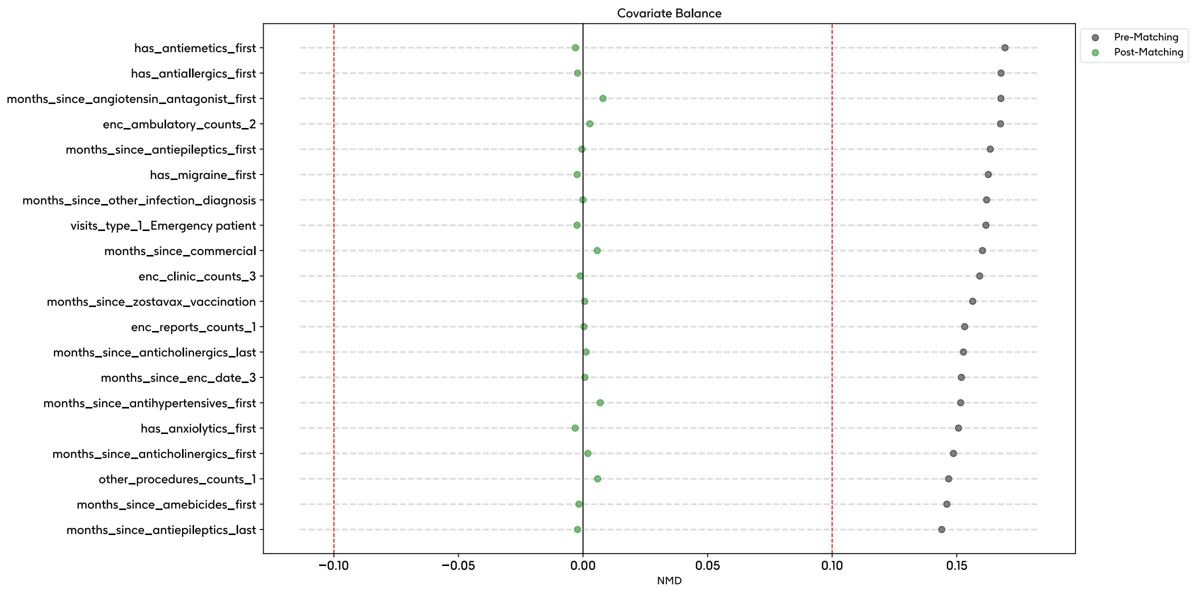

Covariate Balance
has_antiemetics_first
has_antiallergics_first
months_since_angiotensin_antagonist_first
enc_ambulatory_counts_2
months_since_antiepileptics_first
has_migraine_first
months_since_other_infection_diagnosis
visits_type_1_Emergency patient
months_since_commercial
enc_clinic_counts_3
months_since_zostavax_vaccination
enc_reports_counts_1
months_since_anticholinergics_last
months_since_enc_date_3
months_since_antihypertensives_first
has_anxiolytics_first
months_since_anticholinergics_first
other_procedures_counts_1
months_since_amebicides_first
months_since_antiepileptics_last
−0.10
−0.05
0.00
0.05
0.10
0.15
NMD
Pre-Matching
Post-Matching

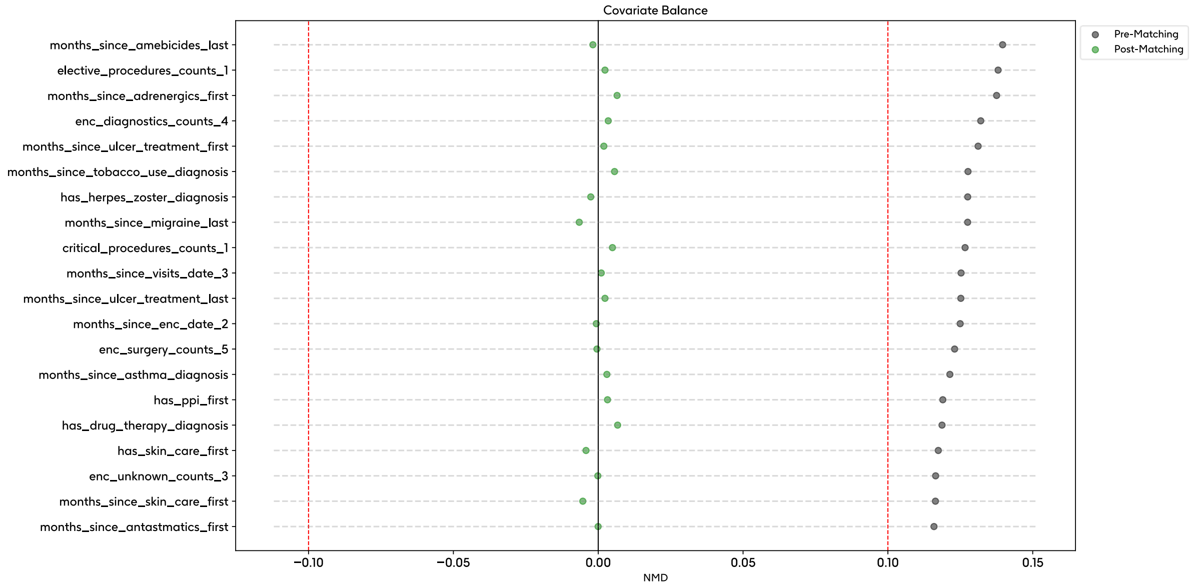

Covariate Balance
months_since_amebicides_last
elective_procedures_counts_1
months_since_adrenergics_first
enc_diagnostics_counts_4
months_since_ulcer_treatment_first
months_since_tobacco_use_diagnosis
has_herpes_zoster_diagnosis
months_since_migraine_last
critical_procedures_counts_1
months_since_visits_date_3
months_since_ulcer_treatment_last
months_since_enc_date_2
enc_surgery_counts_5
months_since_asthma_diagnosis
has_ppi_first
has_drug_therapy_diagnosis
has_skin_care_first
enc_unknown_counts_3
months_since_skin_care_first
months_since_antastmatics_first
−0.10
−0.05
0.00
0.05
0.10
0.15
NMD
Pre-Matching
Post-Matching

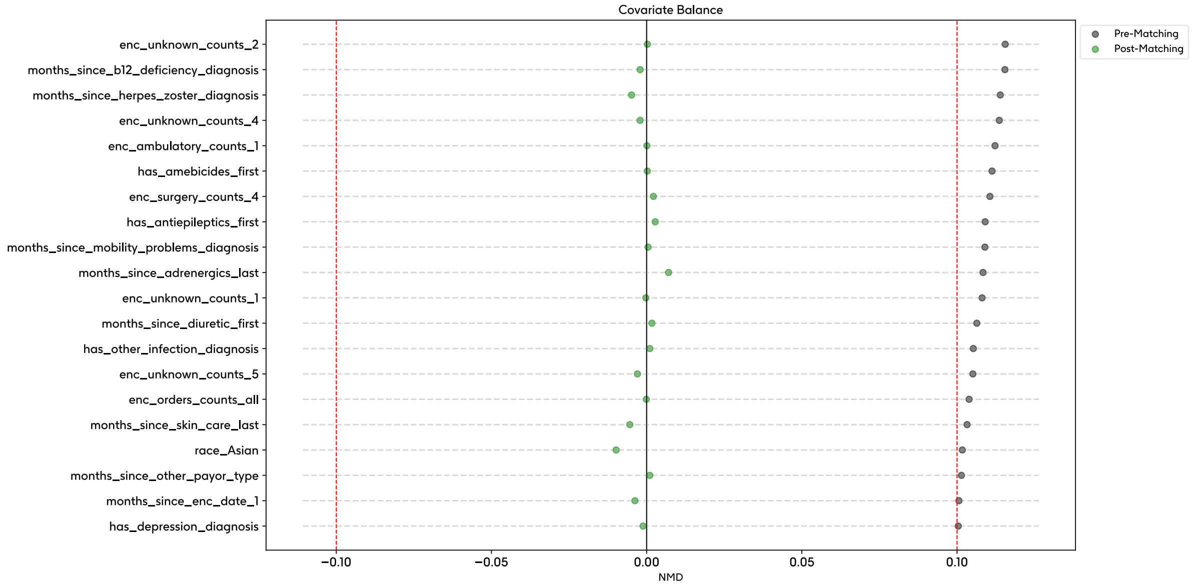

Covariate Balance
enc_unknown_counts_2
months_since_b12_deficiency_diagnosis
months_since_herpes_zoster_diagnosis
enc_unknown_counts_4
enc_ambulatory_counts_1
has_amebicides_first
enc_surgery_counts_4
has_antiepileptics_first
months_since_mobility_problems_diagnosis
months_since_adrenergics_last
enc_unknown_counts_1
months_since_diuretic_first
has_other_infection_diagnosis
enc_unknown_counts_5
enc_orders_counts_all
months_since_skin_care_last
race_Asian
months_since_other_payor_type
months_since_enc_date_1
has_depression_diagnosis
−0.10
−0.05
0.00
0.05
0.10
NMD
Pre-Matching
Post-Matching

(c) Vascular dementia: ZVL vs PPSV23 (all)

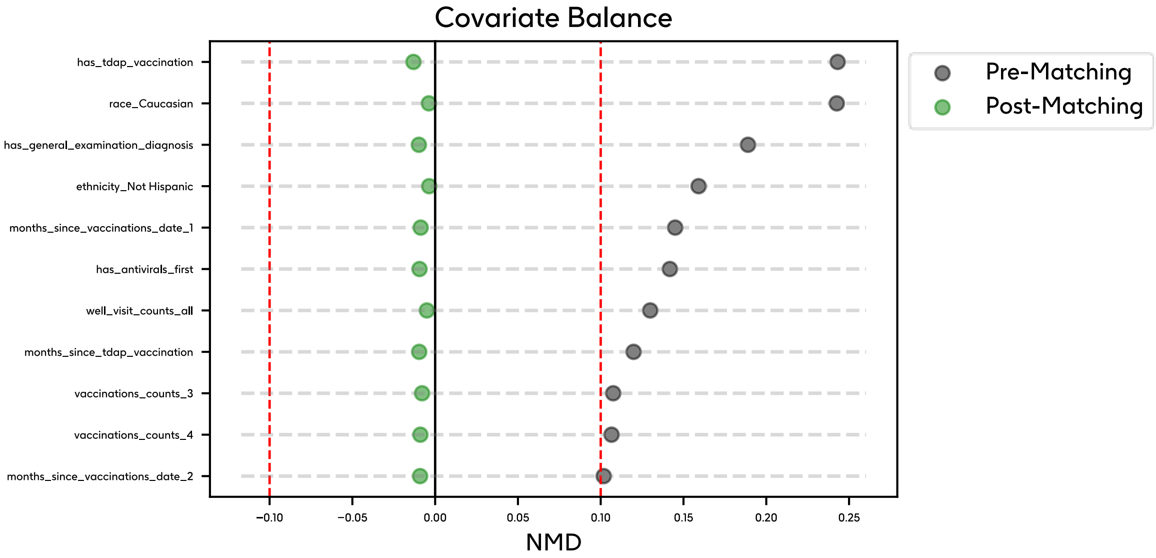

(d) Vascular dementia: RZV (2+ doses) vs PPSV23 (all)

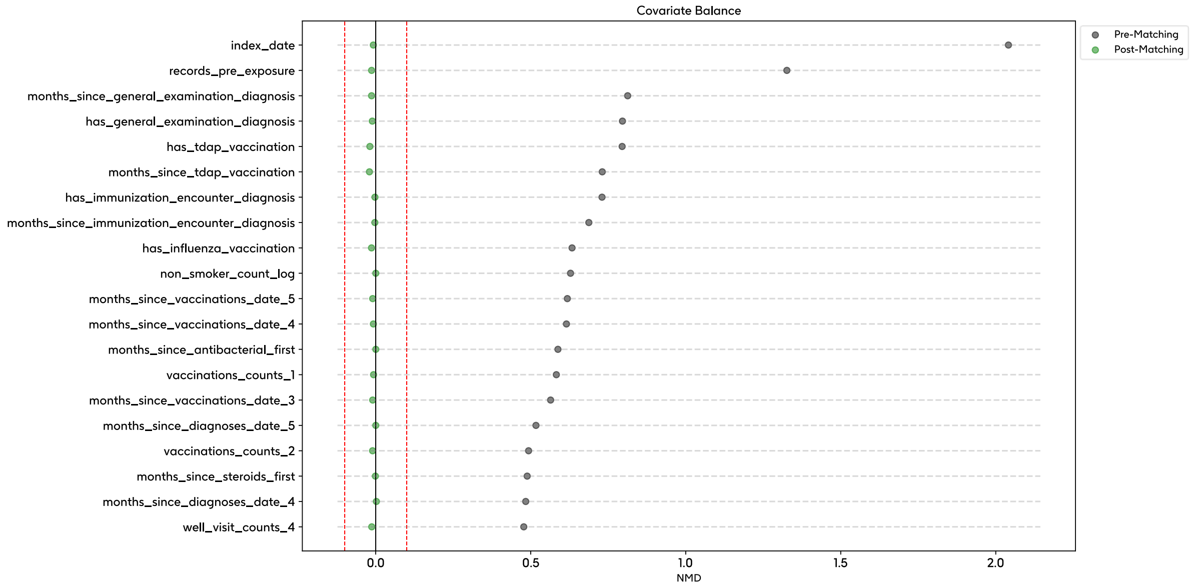

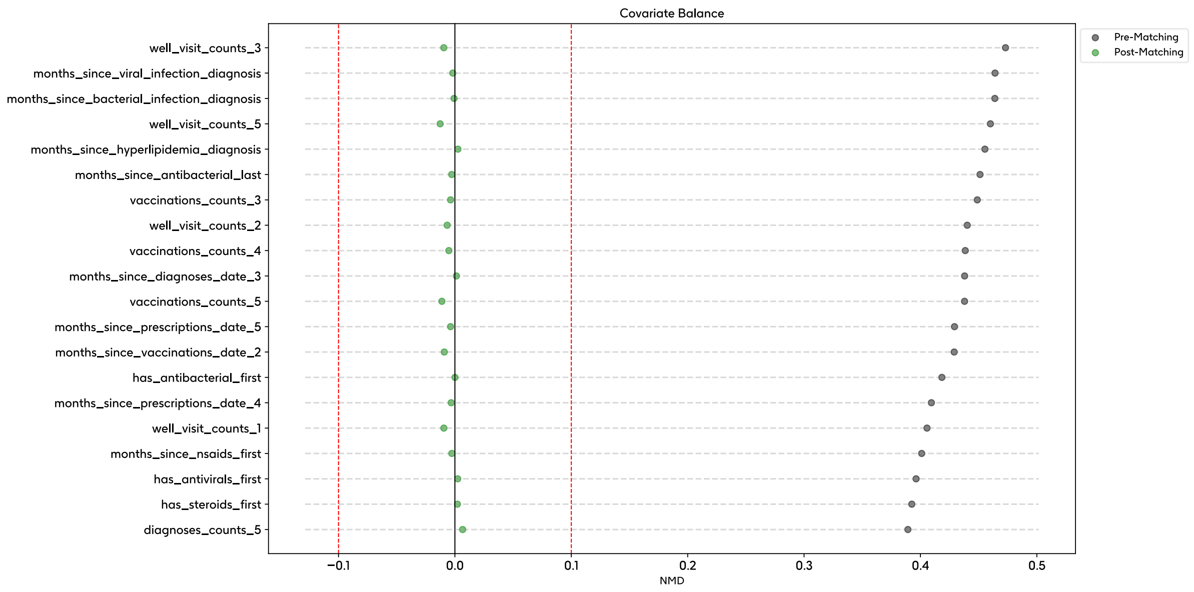
Covariate Balance
well_visit_counts_3
months_since_viral_infection_diagnosis
months_since_bacterial_infection_diagnosis
well_visit_counts_5
months_since_hyperlipidemia_diagnosis
months_since_antibacterial_last
vaccinations_counts_3
well_visit_counts_2
vaccinations_counts_4
months_since_diagnoses_date_3
vaccinations_counts_5
months_since_prescriptions_date_5
months_since_vaccinations_date_2
has_antibacterial_first
months_since_prescriptions_date_4
well_visit_counts_1
months_since_nsaids_first
has_antivirals_first
has_steroids_first
diagnoses_counts_5
Pre-Matching
Post-Matching
−0.1
0.0
0.1
0.2
0.3
0.4
0.5
NMD

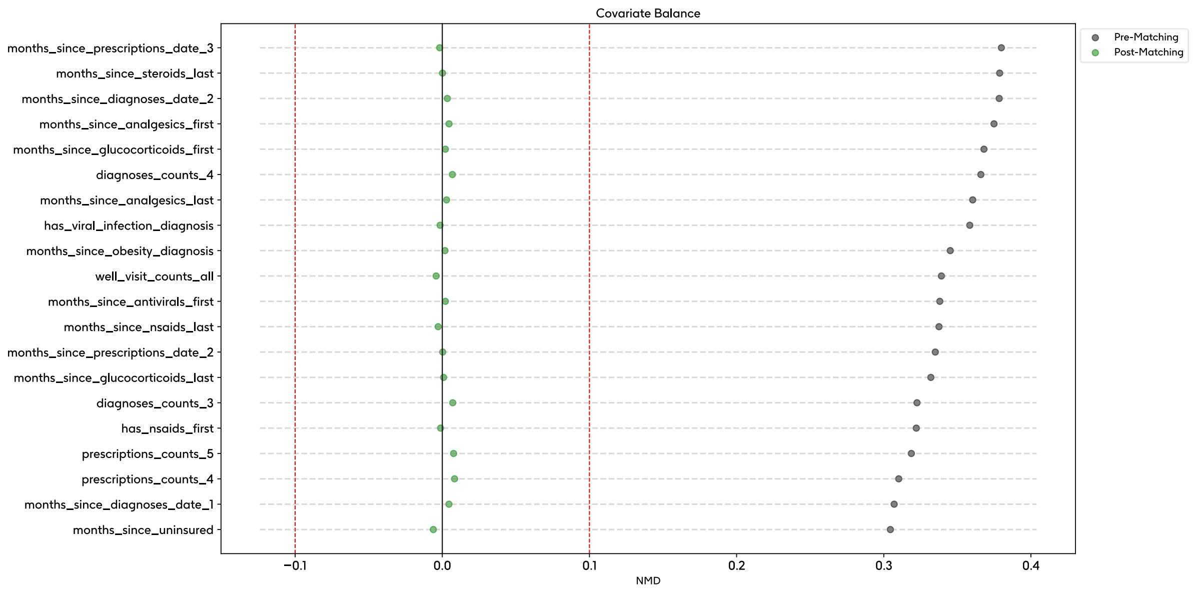
Covariate Balance
months_since_prescriptions_date_3
months_since_steroids_last
months_since_diagnoses_date_2
months_since_analgesics_first
months_since_glucocorticoids_first
diagnoses_counts_4
months_since_analgesics_last
has_viral_infection_diagnosis
months_since_obesity_diagnosis
well_visit_counts_all
months_since_antivirals_first
months_since_nsaids_last
months_since_prescriptions_date_2
months_since_glucocorticoids_last
diagnoses_counts_3
has_nsaids_first
prescriptions_counts_5
prescriptions_counts_4
months_since_diagnoses_date_1
months_since_uninsured
Pre-Matching
Post-Matching
−0.1
0.0
0.1
0.2
0.3
0.4
NMD

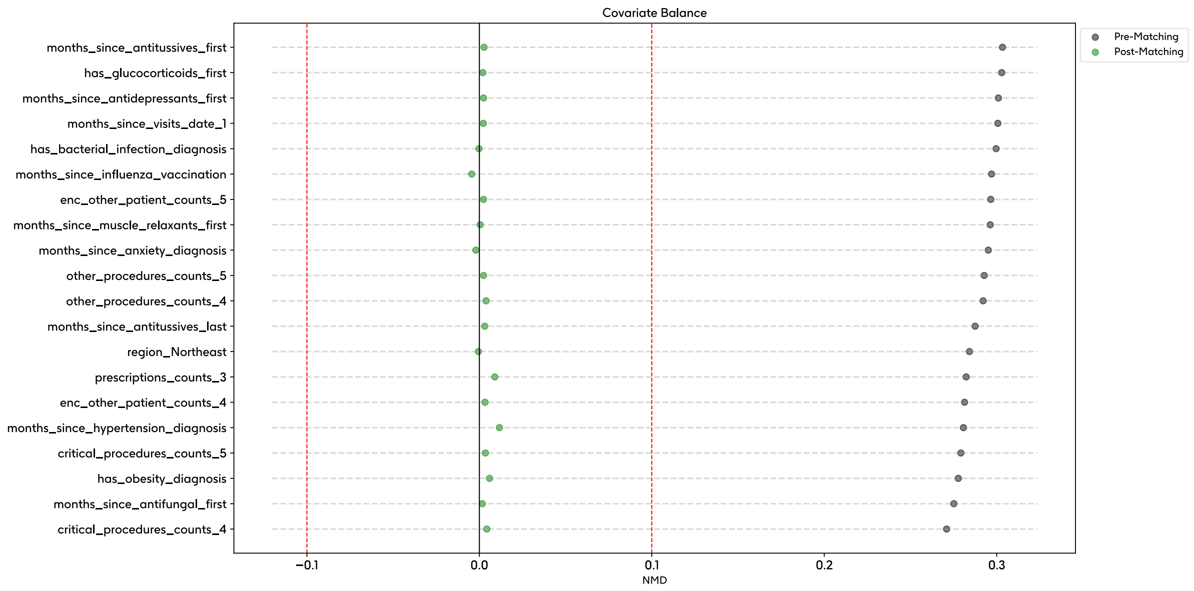
Covariate Balance
months_since_antitussives_first
has_glucocorticoids_first
months_since_antidepressants_first
months_since_visits_date_1
has_bacterial_infection_diagnosis
months_since_influenza_vaccination
enc_other_patient_counts_5
months_since_muscle_relaxants_first
months_since_anxiety_diagnosis
other_procedures_counts_5
other_procedures_counts_4
months_since_antitussives_last
region_Northeast
prescriptions_counts_3
enc_other_patient_counts_4
months_since_hypertension_diagnosis
critical_procedures_counts_5
has_obesity_diagnosis
months_since_antifungal_first
critical_procedures_counts_4
Pre-Matching
Post-Matching
−0.1
0.0
0.1
0.2
0.3
NMD

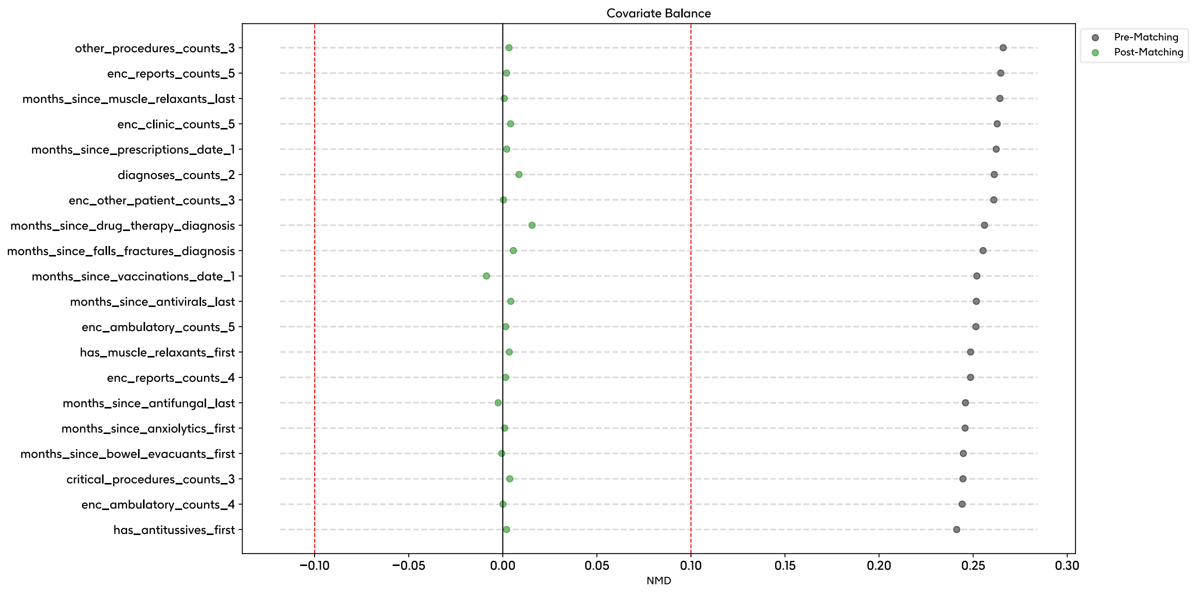
Covariate Balance
Pre-Matching
Post-Matching
other_procedures_counts_3
enc_reports_counts_5
months_since_muscle_relaxants_last
enc_clinic_counts_5
months_since_prescriptions_date_1
diagnoses_counts_2
enc_other_patient_counts_3
months_since_drug_therapy_diagnosis
months_since_falls_fractures_diagnosis
months_since_vaccinations_date_1
months_since_antivirals_last
enc_ambulatory_counts_5
has_muscle_relaxants_first
enc_reports_counts_4
months_since_antifungal_last
months_since_anxiolytics_first
months_since_bowel_evacuants_first
critical_procedures_counts_3
enc_ambulatory_counts_4
has_antitussives_first
−0.10
−0.05
0.00
0.05
0.10
0.15
0.20
0.25
0.30
NMD

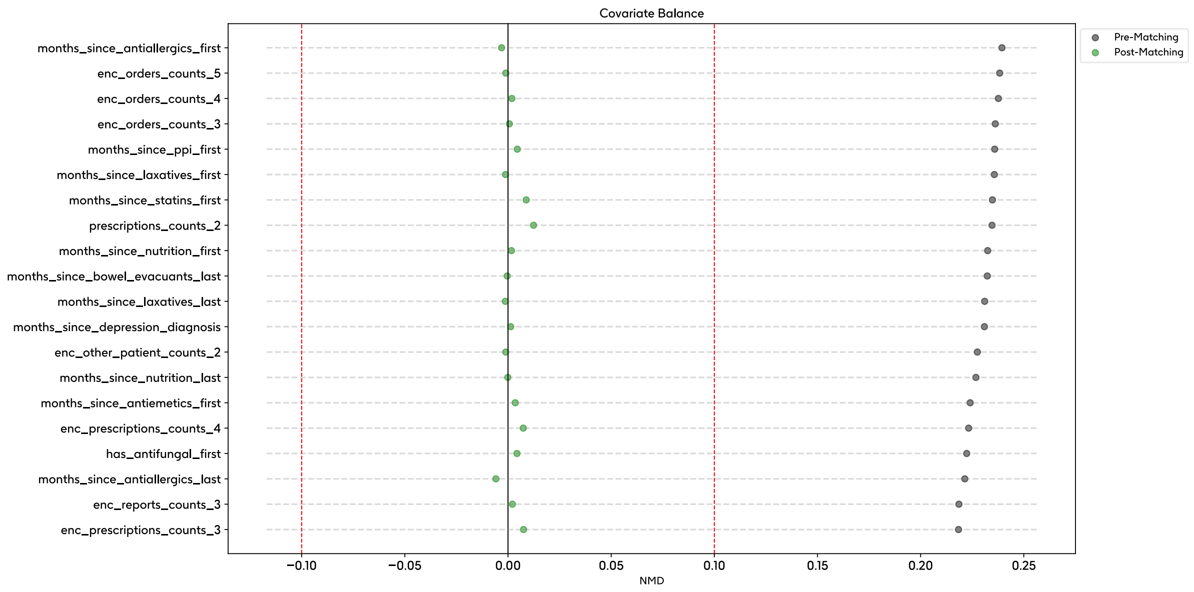
Covariate Balance
Pre-Matching
Post-Matching
months_since_antiallergics_first
enc_orders_counts_5
enc_orders_counts_4
enc_orders_counts_3
months_since_ppi_first
months_since_laxatives_first
months_since_statins_first
prescriptions_counts_2
months_since_nutrition_first
months_since_bowel_evacuants_last
months_since_laxatives_last
months_since_depression_diagnosis
enc_other_patient_counts_2
months_since_nutrition_last
months_since_antiemetics_first
enc_prescriptions_counts_4
has_antifungal_first
months_since_antiallergics_last
enc_reports_counts_3
enc_prescriptions_counts_3
−0.10
−0.05
0.00
0.05
0.10
0.15
0.20
0.25
NMD

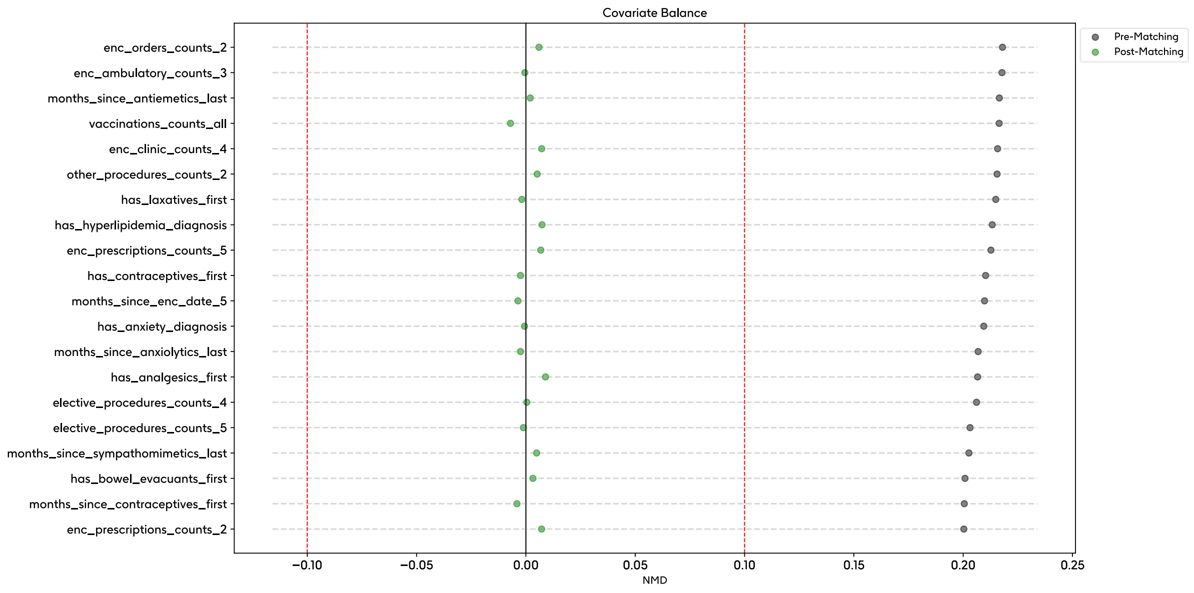
Covariate Balance
Pre-Matching
Post-Matching
enc_orders_counts_2
enc_ambulatory_counts_3
months_since_antiemetics_last
vaccinations_counts_all
enc_clinic_counts_4
other_procedures_counts_2
has_laxatives_first
has_hyperlipidemia_diagnosis
enc_prescriptions_counts_5
has_contraceptives_first
months_since_enc_date_5
has_anxiety_diagnosis
months_since_anxiolytics_last
has_analgesics_first
elective_procedures_counts_4
elective_procedures_counts_5
months_since_sympathomimetics_last
has_bowel_evacuants_first
months_since_contraceptives_first
enc_prescriptions_counts_2
−0.10
−0.05
0.00
0.05
0.10
0.15
0.20
0.25
NMD

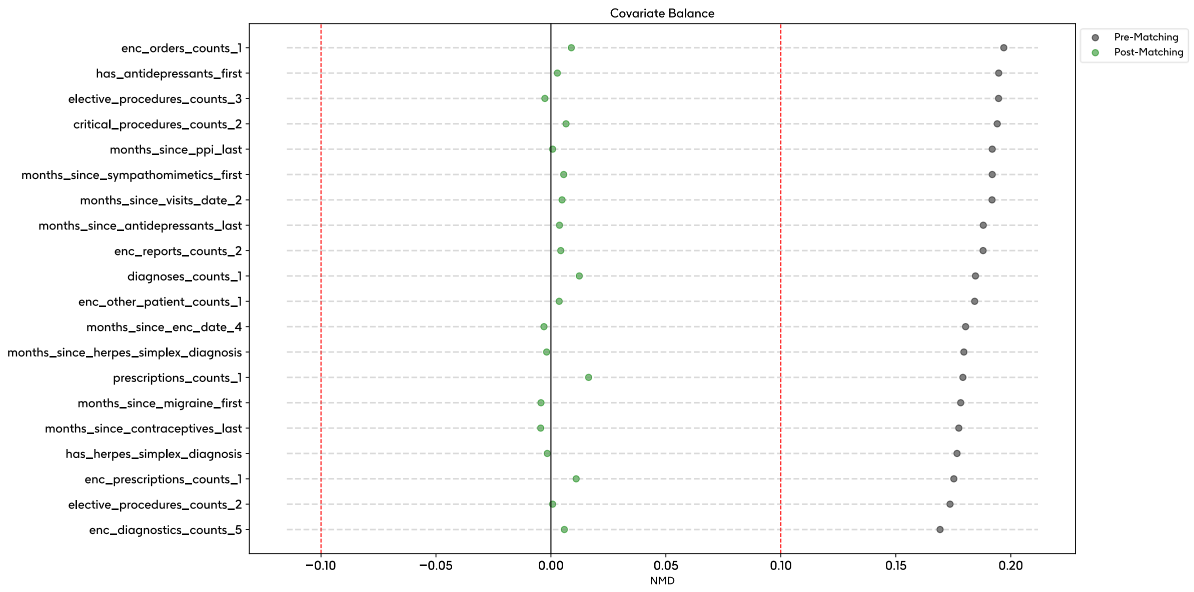
Covariate Balance
enc_orders_counts_1
has_antidepressants_first
elective_procedures_counts_3
critical_procedures_counts_2
months_since_ppi_last
months_since_sympathomimetics_first
months_since_visits_date_2
months_since_antidepressants_last
enc_reports_counts_2
diagnoses_counts_1
enc_other_patient_counts_1
months_since_enc_date_4
months_since_herpes_simplex_diagnosis
prescriptions_counts_1
months_since_migraine_first
months_since_contraceptives_last
has_herpes_simplex_diagnosis
enc_prescriptions_counts_1
elective_procedures_counts_2
enc_diagnostics_counts_5
Pre-Matching
Post-Matching
−0.10
−0.05
0.00
0.05
0.10
0.15
0.20
NMD

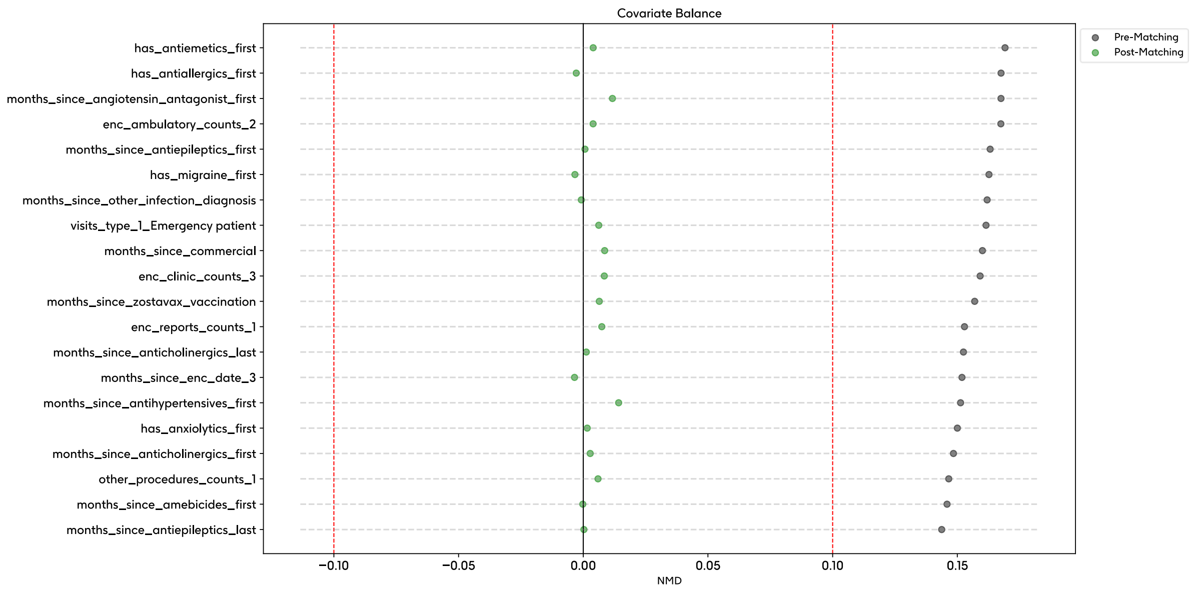
Covariate Balance
has_antiemetics_first
has_antiallergics_first
months_since_angiotensin_antagonist_first
enc_ambulatory_counts_2
months_since_antiepileptics_first
has_migraine_first
months_since_other_infection_diagnosis
visits_type_1_Emergency patient
months_since_commercial
enc_clinic_counts_3
months_since_zostavax_vaccination
enc_reports_counts_1
months_since_anticholinergics_last
months_since_enc_date_3
months_since_antihypertensives_first
has_anxiolytics_first
months_since_anticholinergics_first
other_procedures_counts_1
months_since_amebicides_first
months_since_antiepileptics_last
Pre-Matching
Post-Matching
−0.10
−0.05
0.00
0.05
0.10
0.15
NMD

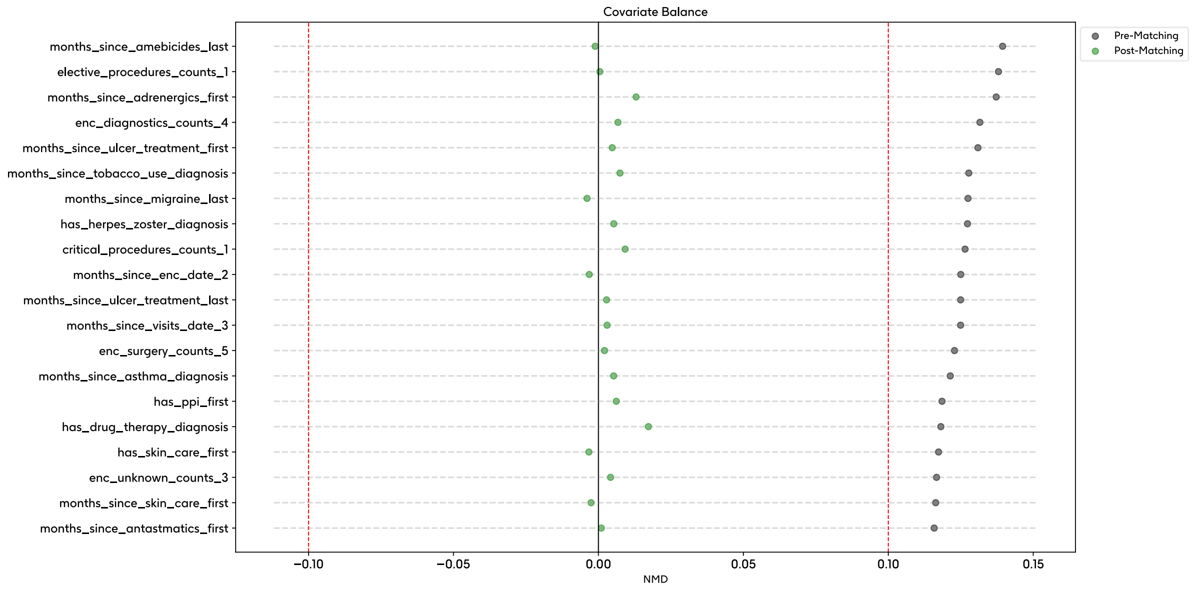
Covariate Balance
months_since_amebicides_last
elective_procedures_counts_1
months_since_adrenergics_first
enc_diagnostics_counts_4
months_since_ulcer_treatment_first
months_since_tobacco_use_diagnosis
months_since_migraine_last
has_herpes_zoster_diagnosis
critical_procedures_counts_1
months_since_enc_date_2
months_since_ulcer_treatment_last
months_since_visits_date_3
enc_surgery_counts_5
months_since_asthma_diagnosis
has_ppi_first
has_drug_therapy_diagnosis
has_skin_care_first
enc_unknown_counts_3
months_since_skin_care_first
months_since_antastmatics_first
Pre-Matching
Post-Matching
−0.10
−0.05
0.00
0.05
0.10
0.15
NMD

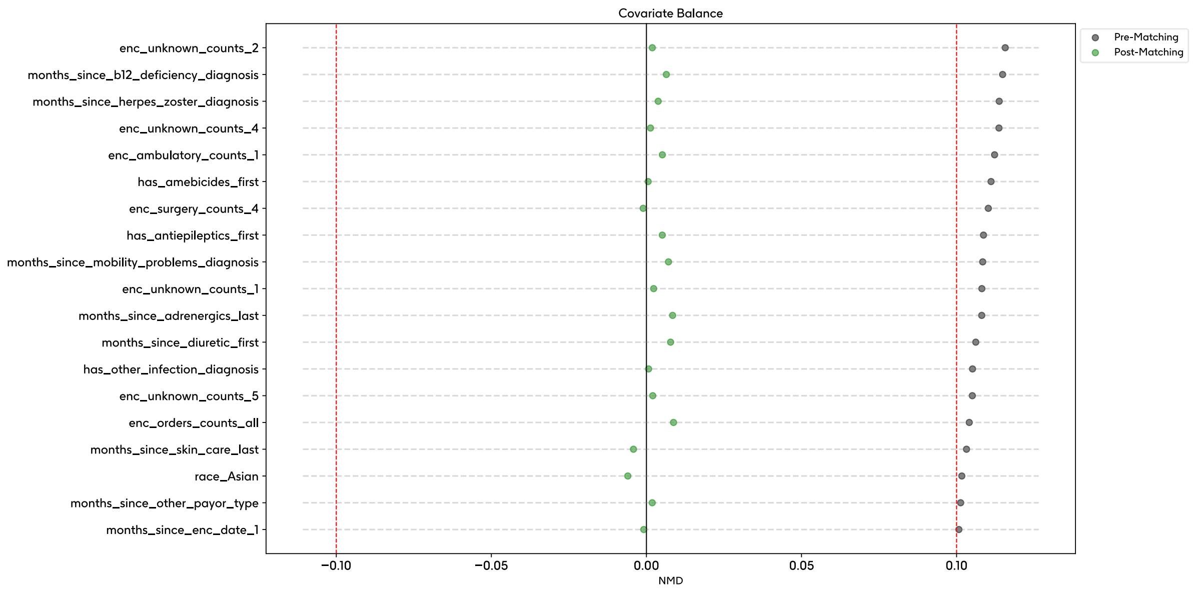

Supplementary Fig. S6 | Pre- and post-matching cohort balance for comparisons of cohorts for different dementia subtypes.

The values on the y-axis are the names of covariates. The dots indicate pre- and post-matching normalized mean distance (NMD) for the corresponding covariate. The red vertical lines indicate the bounds of what is considered a good balance (-0.1 < NMD < 0.1). Post-matching dots within these bounds indicate that the corresponding covariate's mean is well balanced after matching. The covariates are sorted in descending order of their pre-matching NMD.

Note for interpretation of covariate names: For covariates named "months_since_i" (where i=1, 2, 3, 4, 5), the name indicates months between ith last intervention pre-exposure and the exposure date. For "months_since_vaccination", the term "vaccination" refers to any vaccination except the exposure vaccination). For "months_since_diagnosis" covariate, the term "diagnosis" refers to any diagnosis in the Diagnosis table with a certain status. For covariates named "intervention_counts_i", the name indicates the number of interventions in the ith pre-exposure year. The covariate named "well_visits_counts_all" indicates the total count of routine medical and gynecological examinations.

PPSV23, recipients of at least 1 dose of a 23-valent pneumococcal polysaccharide vaccine; RZV (2+ doses), recipients of at least 2 doses of the recombinant zoster vaccine (*Shingrix*, GSK); ZVL, recipients of at least 1 dose of the live-attenuated zoster vaccine (*Zostavax*, Merck).

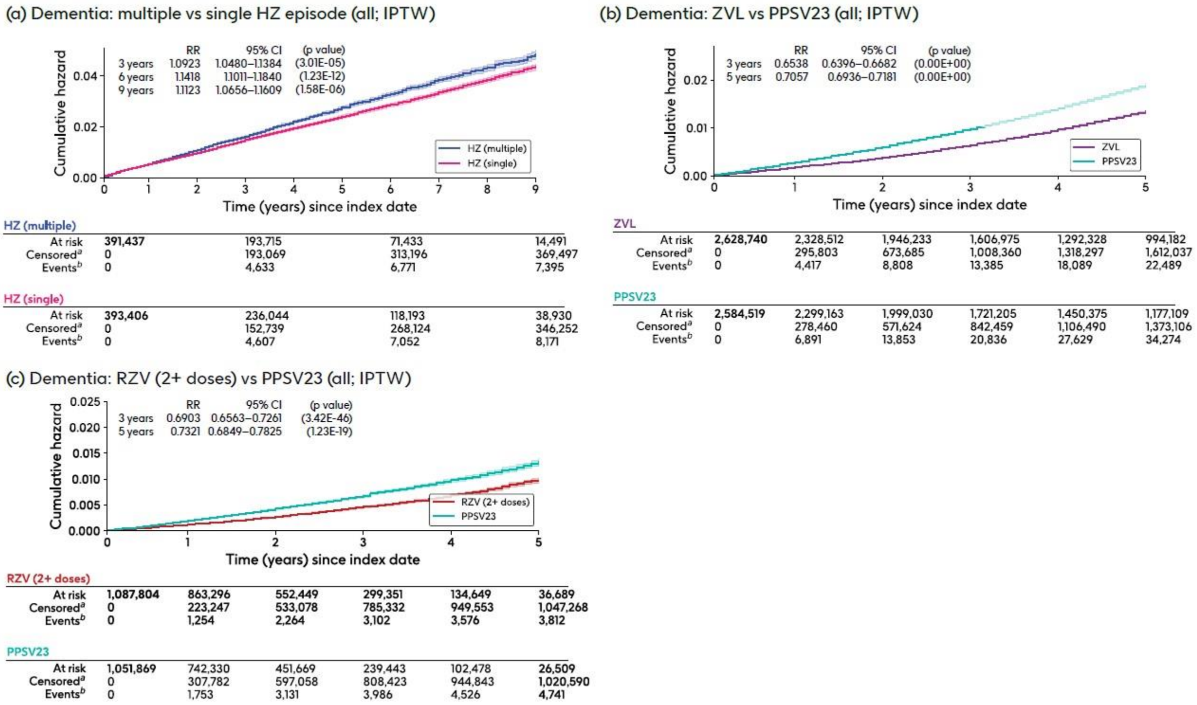

Supplementary Fig. S7 | Comparisons based on IPTW

The curves show the Nelson-Aalen estimates of the cumulative hazard function (y-axis) over the follow-up period (x-axis) with a 95% confidence interval (CI) band (shaded areas around each curve) for each cohort being compared. Cumulative hazards at specific timepoints are compared between the cohorts based on relative risk (RR) using a two-sided chi^2 statistic with 1 degree of freedom without adjustments for multiple comparisons. The sample size (n) for each cohort is indicated as the IPTW-weighted counts of patients at risk in the “At risk” row in the event table.

HZ (multiple) and HZ (single), individuals experiencing at least 2 (multiple) and 1 (single) episodes of herpes zoster (HZ); IPTW, inverse probability of treatment weighting; PPSV23, recipients of at least 1 dose of a 23-valent pneumococcal polysaccharide vaccine; RZV (2+ doses), recipients of at least 2 doses of the recombinant zoster vaccine (*Shingrix*, GSK); ZVL, recipients of at least 1 dose of the live-attenuated zoster vaccine (*Zostavax*, Merck).

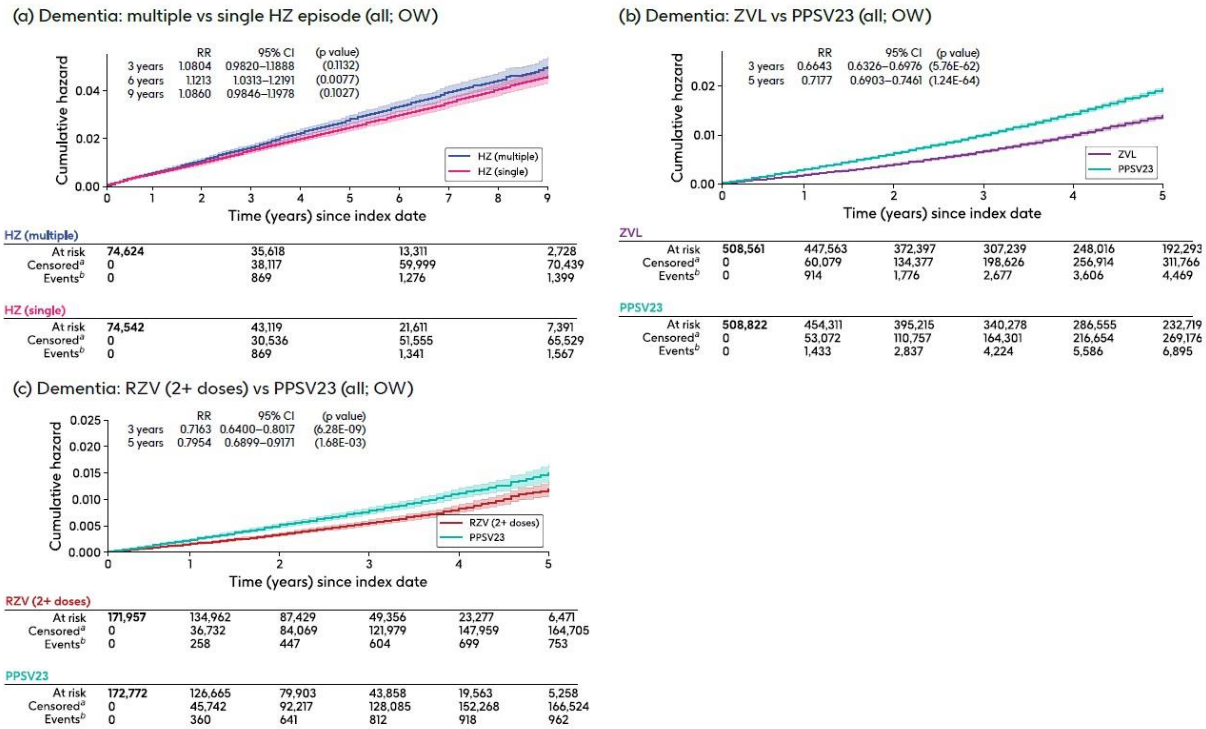

Supplementary Fig. S8 | Comparisons based on OW

The curves show the Nelson-Aalen estimates of the cumulative hazard function (y-axis) over the follow-up period (x-axis) with a 95% confidence interval (CI) band (shaded areas around each curve) for each cohort being compared. Cumulative hazards at specific timepoints are compared between the cohorts based on relative risk (RR) using a two-sided chi^2 statistic with 1 degree of freedom without adjustments for multiple comparisons. The sample size (n) for each cohort is indicated as the OW-weighted counts of patients at risk in the "At risk" row in the event table.

HZ (multiple) and HZ (single), individuals experiencing at least 2 (multiple) and 1 (single) episodes of herpes zoster (HZ); OW, overlap weighting; PPSV23, recipients of at least 1 dose of a 23-valent pneumococcal polysaccharide vaccine; RZV (2+ doses), recipients of at least 2 doses of the recombinant zoster vaccine (*Shingrix*, GSK); ZVL, recipients of at least 1 dose of the live-attenuated zoster vaccine (*Zostavax*, Merck).
